# Serplulimab Plus Chemotherapy, with or without HLX04, versus Chemotherapy as First-Line Treatment for Nonsquamous NSCLC: Final Survival Analysis of the Phase III ASTRUM-002 Study

**DOI:** 10.34133/cancomm.0034

**Published:** 2026-06-10

**Authors:** Xuezhi Hao, Lin Wang, Yanrong Hao, Yanping Hu, Chun Chen, Bi Chen, Yunchao Huang, Aimin Zang, Yan Wang, Zhendong Chen, Wu Zhuang, Jinsheng Shi, Xiubao Ren, Ligong Nie, Guohua Yu, Feng Luo, Yimin Mao, Xiang Wang, Baolan Li, Yuansong Bai, Jianhua Shi, Hongyan Ni, Xiaoli Hou, Haoyu Yu, Jing Li, Qingyu Wang, Jun Zhu, Yuankai Shi, on behalf of the ASTRUM-002 Study Group

**Affiliations:** ^1^ Department of Medical Oncology, Beijing Key Laboratory of Key Technologies for Early Clinical Trial Evaluation of Innovative Drugs for Major Diseases, National Cancer Center/National Clinical Research Center for Cancer/Cancer Hospital, Chinese Academy of Medical Sciences & Peking Union Medical College, Beijing, P. R. China.; ^2^ Department of Medical Oncology, The People’s Hospital of Guangxi Zhuang Autonomous Region, Nanning, Guangxi Zhuang Autonomous Region, P. R. China.; ^3^ Department of Medical Oncology, Hubei Cancer Hospital, Wuhan, Hubei, P. R. China.; ^4^ Department of Thoracic Surgery, Fujian Medical University Union Hospital, Fuzhou, Fujian, P. R. China.; ^5^ Department of Respiratory and Critical Care Medicine, The Affiliated Hospital of Xuzhou Medical University, Xuzhou, Jiangsu, P. R. China.; ^6^ Department of Thoracic Surgery I, Yunnan Cancer Hospital, Kunming, Yunnan, P. R. China.; ^7^ Department of Medical Oncology, Affiliated Hospital of Hebei University, Baoding, Hebei, P. R. China.; ^8^ Department of Respiratory Medicine, Harbin Medical University Cancer Hospital, Harbin, Heilongjiang, P. R. China.; ^9^ Department of Medical Oncology, The Second Affiliated Hospital of Anhui Medical University, Hefei, Anhui, P. R. China.; ^10^ Department of Thoracic Oncology, Fujian Cancer Hospital, Fuzhou, Fujian, P. R. China.; ^11^ Department of Oncology, Cangzhou People’s Hospital, Cangzhou, Hebei, P. R. China.; ^12^ Department of Biotherapy, Tianjin Medical University Cancer Institute & Hospital, Tianjin, P. R. China.; ^13^ Department of Respiratory and Critical Care Medicine, Peking University First Hospital, Beijing, P. R. China.; ^14^ Department of Medical Oncology, Weifang People’s Hospital, Weifang, Shandong, P. R. China.; ^15^ Lung Cancer Center, West China Hospital of Sichuan University, Chengdu, Sichuan, P. R. China.; ^16^ Department of Respiratory and Critical Care Medicine, The First Affiliated Hospital of Henan University of Science and Technology, Luoyang, Henan, P. R. China.; ^17^ Department of Medical Oncology, Xuzhou Central Hospital, Xuzhou, Jiangsu, P. R. China.; ^18^ Department of Medical Oncology, Beijing Chest Hospital, Capital Medical University, Beijing, P. R. China.; ^19^ Department of Oncology and Hematology, China-Japan Union Hospital of Jilin University, Changchun, Jilin, P. R. China.; ^20^ Department of Medical Oncology, Linyi Cancer Hospital, Linyi, Shandong, P. R. China.; ^21^ Global Product Development, Shanghai Henlius Biotech, Inc., Shanghai, P. R. China.

## Abstract

**Background:** ASTRUM-002 met the primary endpoint of progression-free survival (PFS) with the combination of serplulimab plus HLX04 (bevacizumab biosimilar) and chemotherapy at interim analysis. Here, we report the results of the final survival analysis. **Methods:** A total of 636 patients with treatment-naïve, locally advanced or metastatic nonsquamous non-small cell lung cancer (nsq-NSCLC) without epidermal growth factor receptor (*EGFR*) or anaplastic lymphoma kinase (*ALK*)*/*ROS proto-oncogene 1 receptor tyrosine kinase (*ROS1*) genetic alterations were randomized 1:1:1 to receive serplulimab plus HLX04 and chemotherapy (group A), serplulimab plus HLX04 placebo and chemotherapy (group B), or double placebo plus chemotherapy (group C). Patients and the investigators were blinded to the group assignments. The primary endpoint was blinded independent central review-assessed PFS. Overall survival (OS) was the key secondary endpoint. **Results:** At the final analysis, median OS was 23.7 (95% confidence interval [CI] 20.5 to 27.5) months, 26.8 (95% CI 21.2 to 30.9) months, and 20.3 (95% CI 16.2 to 24.6) months in groups A (*n* = 212), B (*n* = 214), and C (*n* = 210), respectively. A significant reduction in risk of death for group B compared to group C was observed (hazard ratio [HR] = 0.66, 95% CI 0.52 to 0.83; *P* < 0.001). A total of 79 (37.6%) patients in group C had crossed over to serplulimab plus HLX04 treatment. Median OS in group C adjusted by the 2-stage model was 14.2 months (95% CI 11.9 to 17.0), corresponding to an adjusted HR of 0.53 (95% CI 0.42 to 0.68; *P* < 0.001) for group B versus group C. Using the rank-preserving structural failure time model, the adjusted median OS in group C was 17.9 months (95% CI 14.2 to 20.3), with a corresponding adjusted HR of 0.65 (95% CI 0.51 to 0.83; *P* < 0.001). No statistical difference in median OS for group A compared to group B (HR = 1.12, 95% CI 0.88 to 1.42; *P* = 0.363) was found. **Conclusions:** Serplulimab plus chemotherapy significantly prolonged OS and maintained PFS benefit compared to chemotherapy; however, the addition of bevacizumab biosimilar HLX04 did not yield further improvement for the first-line treatment of nsq-NSCLC without *EGFR* or *ALK/ROS1* genetic alterations. **Trial registration:** This trial was registered at ClinicalTrials.gov (NCT03952403, date of registration: 2019 May 14).

## Background

Nonsquamous (nsq) histology accounts for over 75% of all non-small cell lung cancer (NSCLC) [1]. For most patients with advanced nsq-NSCLC without targetable driver alterations, platinum-based chemotherapy combined with immune checkpoint inhibitors (ICIs) such as pembrolizumab, atezolizumab, and cemiplimab has shown significant improvements in efficacy and is currently the standard first-line treatment [2–4].

Anti-angiogenic agents, such as bevacizumab, targeting the vascular endothelial growth factor (VEGF) pathway, are also used in combination with ICIs and chemotherapy in selected patient populations [5] based on the findings from the IMpower150 trial. In that study, bevacizumab plus atezolizumab, the programmed cell death ligand 1 (PD-L1) monoclonal antibody (mAb), and chemotherapy showed significant clinical benefits compared to bevacizumab plus chemotherapy [6]. However, the subsequent IMpower151 study revealed no significant survival advantage for atezolizumab plus bevacizumab and chemotherapy compared to bevacizumab plus chemotherapy in Chinese patients [7]. Notably, in both trials, bevacizumab plus chemotherapy served as the control arm for statistical comparison, rather than atezolizumab plus chemotherapy. Further evidence from the APPLE study, which directly evaluated the incremental benefit of adding bevacizumab to atezolizumab and chemotherapy, showed no improvement in efficacy compared with atezolizumab plus chemotherapy [8]. Interpretation of these findings is complicated by the inclusion of heterogeneous patient populations in these studies, comprising both patients without epidermal growth factor receptor (*EGFR*), anaplastic lymphoma kinase (*ALK*), or ROS proto-oncogene 1 (*ROS1*) oncogenic alterations and those with driver gene alterations previously treated with tyrosine kinase inhibitors. In addition, these studies only investigated a PD-L1 mAb and thus do not provide definitive evidence of a synergistic effect of the triplet regimen compared with programmed cell death protein 1 (PD-1) mAb plus chemotherapy. More recently, the HARMONi-6 trial has provided supportive evidence for potential synergism between VEGF and PD-1 blockade. In the HARMONi-6 study, ivonescimab, a bispecific antibody targeting both PD-1 and VEGF, in combination with chemotherapy, significantly improved progression-free survival (PFS) compared with tislelizumab, a PD-1 mAb, plus chemotherapy, irrespective of PD-L1 expression, in the first-line treatment of squamous NSCLC [9]. Nevertheless, direct evidence evaluating the combination of VEGF mAb and PD-1 mAb in patients with nsq-NSCLC remains lacking.

Serplulimab, a recombinant mAb with a higher binding affinity to the human PD-1 receptor than pembrolizumab and nivolumab [10,11], has shown efficacy in different tumor types [12–14]. HLX04 is an approved biosimilar of bevacizumab with comparable efficacy, safety, immunogenicity, and pharmacokinetics [15,16]. Although the pivotal trial of HLX04 was conducted in colorectal cancer, established biosimilar principles allow indication extrapolation once biosimilarity is demonstrated [17]. Accordingly, the China National Medical Products Administration approved HLX04 for all bevacizumab indications, including both colorectal cancer and nsq-NSCLC, at the time of marketing authorization. Besides, previous studies have demonstrated the efficacy and tolerability of serplulimab plus HLX04 in patients with previously treated advanced hepatocellular carcinoma and metastatic colorectal cancer [18,19].

ASTRUM-002 is a phase III study of serplulimab plus chemotherapy, with or without HLX04, versus chemotherapy in patients with previously untreated locally advanced or metastatic nsq-NSCLC without *EGFR*, *ALK*/*ROS1* alterations (NCT03952403). Interim analysis results demonstrated a superior PFS with serplulimab plus chemotherapy compared to chemotherapy alone (hazard ratio [HR] = 0.55, 95% confidence interval [CI] 0.43 to 0.69; *P* < 0.001) that met the protocol-specified superiority criterion [20]. The addition of HLX04 to serplulimab plus chemotherapy did not confer a further improvement in PFS when compared to serplulimab plus chemotherapy (HR = 0.86, 95% CI 0.67 to 1.11; *P* = 0.253) [20]. Overall survival (OS) was a key secondary endpoint; however, the data were not mature at the time of the interim analysis. Here, we present the OS results along with updated efficacy and safety outcomes.

## Materials and Methods

### Patients

Eligible patients were aged 18 to 75 years; had histologically or cytologically diagnosed stage IIIB, IIIC, or IV (American Joint Committee on Cancer 8th edition) nsq-NSCLC that cannot be treated with surgery or radiotherapy; and had no *EGFR* sensitizing mutation or *ALK*/*ROS1* rearrangements. Patients with NSCLC of other histopathological types, or other active malignancies concurrently or within 5 years, and those who were not eligible for HLX04 administration were excluded. A full account of these study eligibility criteria can be found in the study protocol (Data S1).

### Study design and treatment

ASTRUM-002 was a 3-arm, randomized, double-blind, multicenter phase III study conducted across 75 participating hospitals in China, of which 72 enrolled patients; the full list of the ASTRUM-002 Study Group members is listed in Table S1. The first 6 patients were enrolled into a single-arm safety run-in phase and received serplulimab (4.5 mg/kg) plus HLX04 (15 mg/kg) and chemotherapy (pemetrexed [500 mg/m^2^] and carboplatin [area under the curve = 5; for up to 4 cycles]). The study would advance to the randomization phase if no protocol-defined safety events were reported. Patients were randomized 1:1:1 to receive one of the following regimens intravenously once every 3 weeks: group A: serplulimab (4.5 mg/kg) plus HLX04 (15 mg/kg) and chemotherapy (pemetrexed [500 mg/m^2^] and carboplatin [area under the curve = 5 for up to 4 cycles]); group B: serplulimab plus HLX04 placebo and chemotherapy; group C: serplulimab placebo plus HLX04 placebo and chemotherapy. Study drugs were given until the loss of clinical benefit, intolerable toxicity, death, withdrawal of informed consent, or other reasons defined in the protocol, of which the intended duration of serplulimab, HLX04, and pemetrexed treatment is 2 years, whichever occurs first. Patients were allowed to continue with the administration of all drugs apart from carboplatin after 2 years of treatment based on the investigator’s decision. For group C, patients would discontinue chemotherapy and change to serplulimab and HLX04 after the first progressive disease (PD); treatment would continue based on the investigator’s assessment.

The study was conducted in accordance with the principles of the Declaration of Helsinki, International Council for Harmonization Guideline for Good Clinical Practice, and local regulatory requirements. The study protocol, any amendments, and informed consent were approved by the central or independent institutional review board/ethics committee at each participating hospital/site (Table S1).

### Randomization and masking

Eligible patients were randomized using an Interactive Web Response System, with a permuted block randomization algorithm according to the following stratification factors: PD-L1 expression level (based on combined positive score [CPS], negative [CPS <1] vs. positive [CPS ≥1] vs. indeterminable), smoking history (yes vs. no), and brain metastasis (yes vs. no). Patients, investigators, and the study team were masked to the group assignments.

### Endpoints

The primary endpoint was PFS assessed by the blinded independent central review (BICR). Key secondary endpoints included OS, PFS assessed by the investigator, objective response rate (ORR), duration of response (DoR), safety, and potential predictive and prognostic biomarkers. Other secondary endpoints, such as immunogenicity, pharmacokinetic parameters, and quality of life, will be reported separately. All subgroup analyses were exploratory and had no statistical assumptions.

### Assessments

Tumor imaging by computed tomography or magnetic resonance imaging was scheduled at baseline, then every 6 weeks within 48 weeks from the first dose of study drugs, and every 12 weeks thereafter. Brain imaging and bone scans were mandatory at baseline and then based on the investigator’s judgment. Tumor response was assessed by BICR and investigators per the Response Evaluation Criteria in Solid Tumors version 1.1. All adverse events (AEs) were monitored from the time of the first dose of study drugs up to 90 days after the last dose of study drugs. AEs were coded using the Medical Dictionary for Regulatory Activities version 23.0 and graded for severity using the National Cancer Institute Common Terminology Criteria for Adverse Events version 5.0.

Archival tumor samples collected within 6 months before the start of study treatment, or fresh biopsy samples collected during the screening period, were used to analyze PD-L1 expression, *EGFR* sensitizing mutations, and *ALK*/*ROS1* rearrangements. PD-L1 expression was evaluated by immunohistochemistry (IHC) using the PD-L1 IHC 22C3 pharmDx assay (catalog# SK006, Agilent Technologies). Viable cells were defined as PD-L1-positive if they showed partial or complete membrane staining at any intensity. CPS was derived for tumor PD-L1 expression by dividing the number of PD-L1-positive cells by the total number of viable tumor cells, then multiplying by 100. Tumor proportion score (TPS) was derived by dividing the number of PD-L1-positive tumor cells by the total number of viable tumor cells, then multiplying by 100%. *EGFR* sensitizing mutations and *ALK*/*ROS1* rearrangements were detected using next-generation sequencing or real-time amplification refractory mutation polymerase chain reaction (PCR) with the AmoyDx *EGFR/ALK/ROS1* Mutations Detection Kit (catalog# 8.01.0079, Amoy Diagnostics) on the Mx3000P system (catalog# 401400, Agilent Technologies) or SLAN-96S (Shanghai Hongshi Medical Technology) platform according to the manufacturer’s protocol. Results were defined as positive if the Ct (sample) − Ct (control) < Ct (cutoff) in accordance with the criteria provided in the manufacturer’s instructions. *EGFR* mutation testing using blood samples was not acceptable for enrolment evaluation.

### Statistical analysis

The sample size for the randomization phase was determined based on the primary endpoint, PFS. Assuming a median PFS of 6 months in group C and a hazard ratio of 0.69 for group B versus group C, with an accrual period of 24 months and a total study duration of 30 months, and using a 2-sided type I error rate (α) of 0.05, a minimum of 264 PFS events is required to achieve 85% power. Allowing for a 15% dropout rate, approximately 400 patients in total were required for enrolment in groups B and C (200 in each group). For the comparison between group A and group B, assuming a median PFS of 8.7 months in group B and a hazard ratio of 0.67 for group A versus group B, with all other parameters identical to those used in the B versus C comparison, a total of 404 patients were needed for these 2 groups (202 in each group). Overall, the study planned to enroll 606 patients (approximately 202 in each group), with at least 396 PFS events required for the final analysis.

Efficacy analyses were performed in the intent-to-treat (ITT) population that included all randomized patients and the per-protocol set (PPS) that comprised all randomized patients who underwent at least one posttreatment tumor assessment and had no major protocol deviations that significantly impacted the primary efficacy outcome. Safety data were summarized from the safety set (SS) that included all patients who received any dose of study drugs. Comparison of the primary and secondary efficacy endpoints between groups B and C, and between groups A and B, was evaluated using a hierarchical log-rank test with stratification factors. The hierarchical Cox proportional risk model was used to estimate PFS and OS, and their 95% confidence intervals (CIs); the median and its 95% CI were estimated by the Kaplan–Meier method. The superiority criteria for a significant statistical difference between groups B and C, or between groups A and B, are indicated by a *P* value of <0.05. The stratified Cochran–Mantel–Haenszel method was used to test the between-group variation in the ORR and to estimate the odds ratio (OR) and its 95% CI. The median DoR and its 95% CI were estimated using the Kaplan–Meier method.

A post hoc supportive analysis using a 2-stage model [21] and the rank-preserving structural failure time model (RPSFTM) [22] were performed to estimate the effect of in-study crossover on OS with data from the patients in group C who progressed per BICR assessment and changed to serplulimab plus HLX04 prior to any subsequent antitumor therapy. All data analyses were produced using Statistical Analysis Software (SAS Institute) version 9.4 or later. Additional details on the statistical analysis are provided in the statistical analysis plan (Data S2).

## Results

### Patients’ disposition and baseline characteristics

**Fig. 1.**
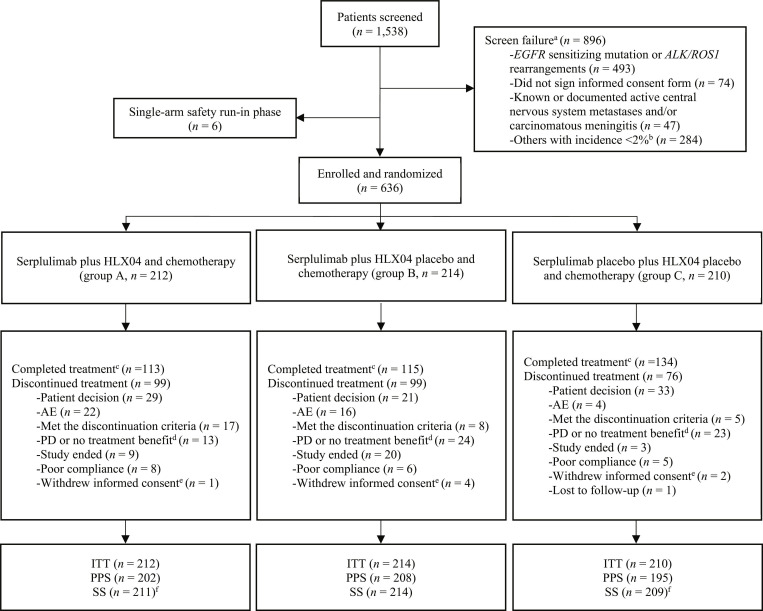
Study profile. ^a^ Two screened patients had *EGFR* sensitizing mutation or *ALK/ROS1* rearrangements and known or documented active central nervous system metastases and/or carcinomatous meningitis. ^b^ Included any eligibility criteria that led to failure for enrolment in 2% or less of the screened patients. ^c^ Defined as patients who ended the treatment after progressive disease as confirmed by the BICR, died, or completed the 2-year treatment (35 dosing cycles) and would not continue to receive the investigational product as evaluated by the investigator. ^d^ Assessed by the investigator. ^e^ Patients who withdrew informed consent did not undergo subsequent survival follow-up. ^f^ One patient each in group A and group C did not receive any study treatment and were therefore excluded from safety analyses. Between 2019 November 25 and 2022 June 15, a total of 1,538 patients were screened, of whom 636 were randomized. The ITT population consisted of all randomized patients (*n* = 636), with 212 in group A, 214 in group B, and 210 in group C; the PPS consisted of all randomized subjects who have received at least one posttreatment tumor assessment without any major protocol deviation that can significantly affect the primary efficacy (*n* = 605), with 202 in group A, 208 in group B, and 195 in group C; the SS consisted of all subjects who have received at least one dose of study treatment (*n* = 634), with 211 in group A, 214 in group B, and 209 in group C. Abbreviations: AE, adverse event; *ALK*, anaplastic lymphoma kinase; BICR, blinded independent central review; *EGFR*, epidermal growth factor receptor; ITT, intent-to-treat; PPS, per-protocol set; *ROS1*, *ROS* proto-oncogene 1 receptor tyrosine kinase; SS, safety set.

Between 2019 November 25 and 2022 June 15, 636 eligible patients were randomized to group A (*n* = 212), group B (*n* = 214), and group C (*n* = 210) (Fig. 1). Two randomized patients (one in group A and one in group C) did not receive any dose of study drugs and thus were included in the ITT analysis population but not in the SS. By the time of the final survival analysis data cutoff date (2025 August 7), all patients had either completed or discontinued treatment. Treatment was completed by 113 (53.3%) patients in group A, 115 (53.7%) in group B, and 134 (63.8%) in group C. Treatment completion was defined as discontinuation after PD confirmation by BICR, death, or completion of the planned 2-year treatment period (35 dosing cycles), after which patients no longer received the investigational product at the investigator’s discretion. Overall, 274 (43.1%) patients discontinued study treatment, most commonly due to patient decision (*n* = 83, 13.1%), AEs (*n* = 42, 6.6%), or delayed dosing meeting the end-of-treatment criteria (*n* = 30, 4.7%) (Fig. 1). A total of 79 patients (37.6%) in group C crossed over to serplulimab plus HLX04 treatment at the final survival analysis. In group A, 21 (9.9%) patients continued the assigned treatment regimen, while 27 (12.6%) in group B continued serplulimab and pemetrexed beyond PD by the time of the final survival analysis. Subsequent antitumor therapies received by the patients are listed in Table S2. Specifically, 32 (15.1%), 42 (19.6%), and 43 (20.5%) of the patients in groups A, B, and C, respectively, have received subsequent antitumor immunotherapy after first disease progression. Demographic and disease baseline characteristics were generally well balanced between the treatment groups except for minor imbalances in terms of clinical stage and PD-L1 TPS score (Table 1). A total of 39 (18.4%), 41 (19.2%), and 39 (18.6%) patients in groups A, B, and C had brain metastases, respectively. Overall, 495 (77.8%) patients had a PD-L1 CPS ≥1, while 374 (58.8%) patients had PD-L1 TPS ≥1%. A slightly higher proportion of patients in group A had a PD-L1 TPS <1% (44.3% vs. 39.3% vs. 32.4% in groups A, B, and C, respectively), and were diagnosed with stage III nsq-NSCLC (18.9% vs. 14.0% vs. 14.8% in groups A, B, and C, respectively).

**Table 1.** Demographics and disease baseline characteristics of patients in the ITT population

Characteristics	Group A (*n* = 212)	Group B (*n* = 214)	Group C (*n* = 210)
Median age (range), years	62 (27–74)	62 (29–75)	61 (33–75)
Sex, *n* (%)			
Male	152 (71.7)	157 (73.4)	156 (74.3)
Female	60 (28.3)	57 (26.6)	54 (25.7)
Han ethnicity, *n* (%)	199 (93.9)	200 (93.5)	200 (95.2)
ECOG performance status, *n* (%)			
0	53 (25.0)	60 (28.0)	57 (27.1)
1	158 (74.5)	154 (72.0)	153 (72.9)
Missing ^a^	1 (0.5)	0 (0.0)	0 (0.0)
History of tobacco use, *n* (%)			
Yes	142 (67.0)	143 (66.8)	140 (66.7)
No	70 (33.0)	71 (33.2)	70 (33.3)
History of brain metastasis, *n* (%)			
Yes	39 (18.4)	41 (19.2)	39 (18.6)
No	173 (81.6)	173 (80.8)	171 (81.4)
PD-L1 expression by CPS, *n* (%)			
CPS < 1	43 (20.3)	44 (20.6)	44 (21.0)
CPS ≥ 1	166 (78.3)	166 (77.6)	163 (77.6)
Not evaluable	3 (1.4)	4 (1.9)	3 (1.4)
PD-L1 expression by TPS, *n* (%)			
TPS < 1%	94 (44.3)	84 (39.3)	68 (32.4)
1% ≤ TPS < 50%	58 (27.4)	64 (29.9)	73 (34.8)
TPS ≥ 50%	55 (25.9)	62 (29.0)	62 (29.5)
Not evaluable	5 (2.4)	4 (1.9)	7 (3.3)
Nonsquamous histologic subtype, *n* (%)			
Adenocarcinoma	210 (99.1)	208 (97.2)	208 (99.0)
Large cell carcinoma	0 (0.0)	3 (1.4)	0 (0.0)
Others ^b^	2 (0.9)	3 (1.4)	2 (1.0)
Clinical stage, *n* (%)			
Stage IIIB/IIIC	40 (18.9)	30 (14.0)	31 (14.8)
Stage IV	172 (81.1)	184 (86.0)	179 (85.2)

Group A, serplulimab plus HLX04 and chemotherapy; Group B, serplulimab plus HLX04 placebo and chemotherapy; Group C, serplulimab placebo plus HLX04 placebo and chemotherapy; CPS, combined positive score; ECOG, Eastern Cooperative Oncology Group; ITT, intent-to-treat; PD-L1, programmed cell death ligand-1; TPS, tumor proportion score

^a^ One patient in group A had an ECOG performance status score of “not checked” recorded during the screening period.

^b^ Two patients in group C had a mucinous adenocarcinoma, one patient each in group A and B had a lympho-epithelioma-like carcinoma, one patient each in group A and B had a sarcomatoid carcinoma, and one patient in group B had a spindle cell neoplasm.

### Efficacy

#### Progression-free survival

**Fig. 2.**
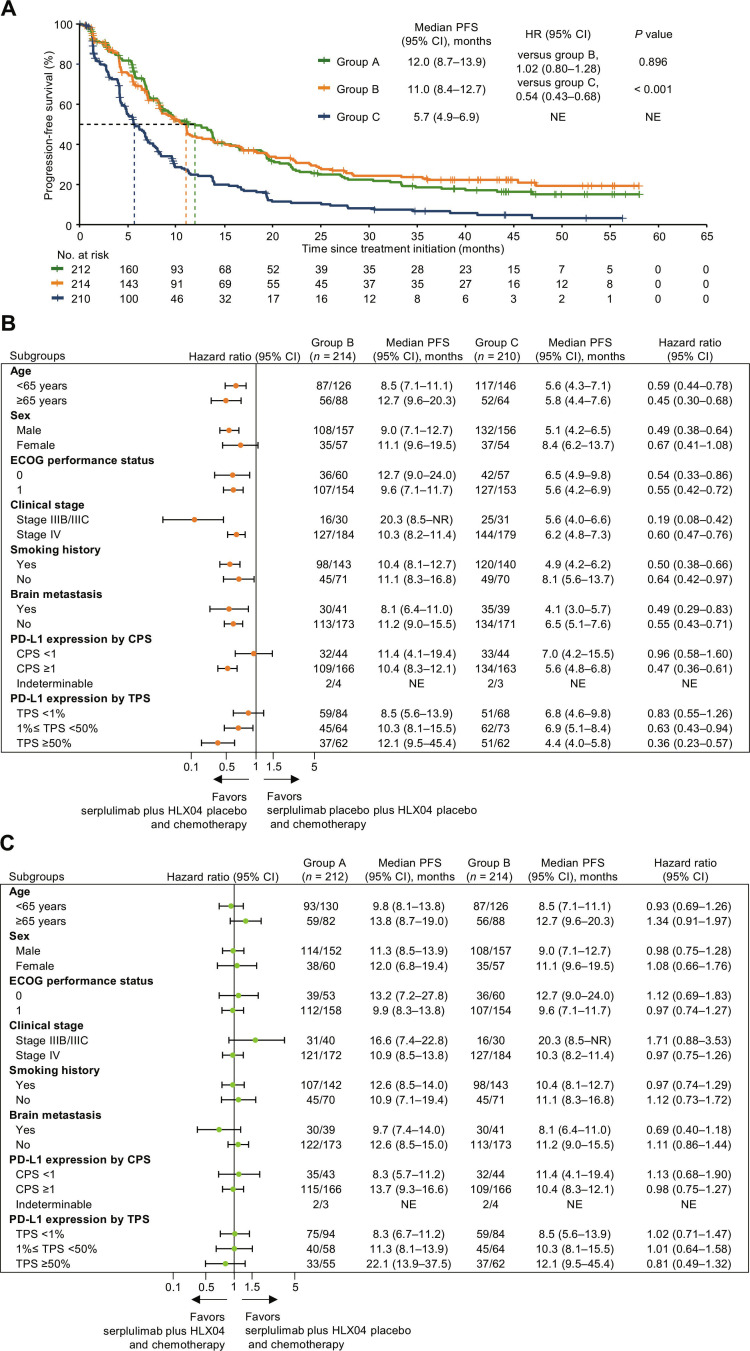
Kaplan–Meier survival curve and subgroup analysis of BICR-assessed progression-free survival, showing the progression-free survival benefits with serplulimab plus chemotherapy compared to chemotherapy alone in the intent-to-treat population and across subgroups. (A) In the ITT population. The median PFS was 12.0 (95% CI 8.7 to 13.9) months in group A, 11.0 (95% CI 8.4 to 12.7) months in group B, and 5.7 (95% CI 4.9 to 6.9) in group C. Serplulimab plus chemotherapy significantly improved PFS compared with chemotherapy alone with an HR of 0.54 (95% CI 0.43 to 0.68; *P* < 0.001). The addition of HLX04 did not translate to an improvement in PFS compared with serplulimab plus chemotherapy (HR = 1.02, 95% CI 0.80 to 1.28; *P* = 0.896). (B) Exploratory subgroup analysis of PFS in group B vs. group C, suggesting a PFS benefit with serplulimab plus chemotherapy over chemotherapy alone across the various patient subgroups. (C) Exploratory subgroup analysis of PFS in group A vs. group B, implying a lack of PFS benefit with HLX04 and serplulimab plus chemotherapy over serplulimab plus chemotherapy across the various patient subgroups. Group A, serplulimab plus HLX04 and chemotherapy; Group B, serplulimab plus HLX04 placebo and chemotherapy; Group C, serplulimab placebo plus HLX04 placebo and chemotherapy. Abbreviations: BICR, blinded independent central review; CI, confidence interval; CPS, combined positive score; ECOG, Eastern Cooperative Oncology Group; HR, hazard ratio; ITT, intent-to-treat; NE, not evaluable; NR, not reached; PD-L1, programmed cell death ligand 1; PFS, progression-free survival; TPS, tumor proportion score.

Patients received a median of 11 (range, 1 to 80) cycles of serplulimab and 11 (range, 1 to 70) cycles of HLX04 in group A. In group B, patients received a median of 12 (range, 1 to 80) cycles of serplulimab. Patients in groups A, B, and C received a median of 10 (range, 1 to 76), 12 (range, 1 to 80), and 6 (range, 1 to 66) cycles of pemetrexed, respectively; median cycles of carboplatin were 4 (range, 1 to 4) for each group. At the final survival analysis, BICR-assessed PD or death was observed in 152 (71.7%) patients in group A, 143 (66.8%) patients in group B, and 169 (80.5%) patients in group C. Consistent with the findings in the interim analysis [20], median PFS was significantly prolonged in group B compared to group C (11.0 [95% CI, 8.4 to 12.7] months vs. 5.7 [95% CI, 4.9 to 6.9] months), reducing the risk of PD or death by 46% (HR = 0.54, 95% CI 0.43 to 0.68, *P* < 0.001; Fig. 2A). The PFS rate at 36 months was 23.0% (95% CI, 16.9 to 29.6) in group B and 6.7% (95% CI, 3.5 to 11.4) in group C (Fig. 2A). Similarly, subgroup analysis of PFS showed various extents of benefit for group B compared to group C across all exploratory subgroups, particularly for those with brain metastasis (HR = 0.49, 95% CI 0.29 to 0.83) and PD-L1 TPS ≥50% (HR = 0.36, 95% CI 0.23 to 0.57; Fig. 2B).

Consistent with the data from the interim analysis [20], no further benefit in median PFS was observed in group A, when compared to group B (12.0 months vs. 11.0 months, HR = 1.02, 95% CI 0.80 to 1.28; *P* = 0.896). The 36-month PFS rate was 18.5% (95% CI, 13.1 to 24.8) in group A compared to 23.0% (95% CI, 16.9 to 29.6) in group B (Fig. 2A). Similarly, subgroup analysis revealed no statistical improvement in median PFS for group A compared to group B across all the exploratory subgroups (Fig. 2C). The results assessed by investigators were in line with those assessed by BICR (Fig. S1).

#### Overall survival

**Fig. 3.**
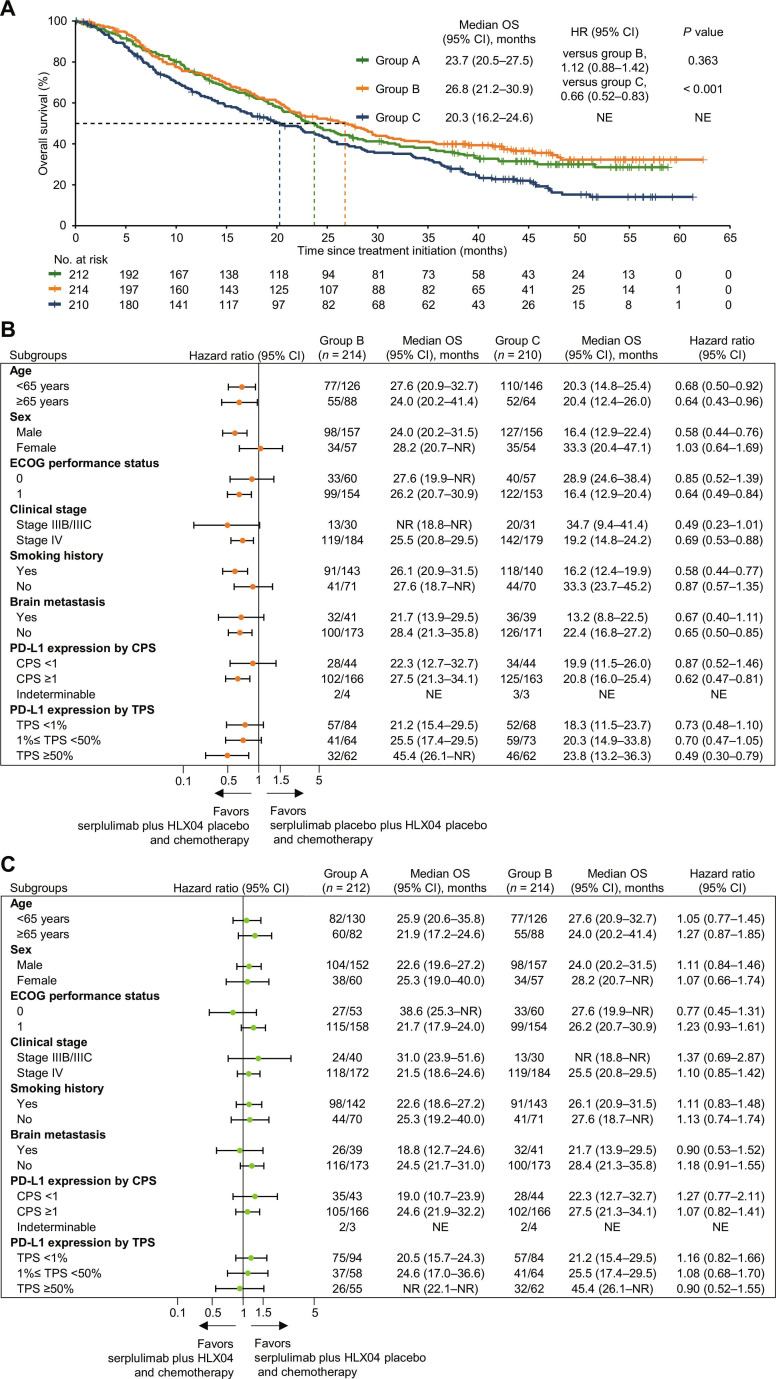
Kaplan–Meier survival curve and subgroup analysis of overall survival, showing the overall survival benefits with serplulimab plus chemotherapy compared to chemotherapy alone in the intent-to-treat population and across subgroups. (A) In the ITT population. The median OS was 23.7 (95% CI 20.5 to 27.5) months in group A, 26.8 (95% CI 21.2 to 30.9) months in group B, and 20.3 (95% CI 16.2 to 24.6) in group C. Serplulimab plus chemotherapy significantly improved OS compared with chemotherapy alone with an HR of 0.66 (95% CI 0.52 to 0.83; *P* < 0.001). The addition of HLX04 did not translate to an OS benefit compared with serplulimab plus chemotherapy (HR = 1.12, 95% CI 0.88 to 1.42; *P* = 0.363). (B) Exploratory subgroup analysis of OS in group B vs. group C, suggesting an OS benefit with serplulimab plus chemotherapy over chemotherapy alone across the various patient subgroups. (C) Exploratory subgroup analysis of OS in group A vs. group B, implying a lack of OS benefit with HLX04 and serplulimab plus chemotherapy over serplulimab plus chemotherapy across the various patient subgroups. Group A, serplulimab plus HLX04 and chemotherapy; Group B, serplulimab plus HLX04 placebo and chemotherapy; Group C, serplulimab placebo plus HLX04 placebo and chemotherapy. Abbreviations: CI, confidence interval; CPS, combined positive score; ECOG, Eastern Cooperative Oncology Group; HR, hazard ratio; ITT, intent-to-treat; NE, not evaluable; NR, not reached; OS, overall survival; PD-L1, programmed cell death ligand 1; TPS, tumor proportion score.

OS data were not mature as of the date of the interim analysis on 2023 June 15. At the data cutoff date for the final survival analysis on 2025 August 7, the median follow-up duration was 48.4 (95% CI 45.9 to 49.9) months in group A, 45.4 (95% CI 43.3 to 49.1) months in group B, and 45.7 (95% CI 43.6 to 51.7) months in group C. A total of 142 (67.0%) patients in group A, 132 (61.7%) in group B, and 162 (77.1%) in group C had died. In respective groups, 70 (33.0%), 82 (38.3%), and 48 (22.9%) were censored due to being alive (57 [26.9%] vs. 71 [33.2%] vs. 36 [17.1%]), withdrawal of informed consent (2 [0.9%] vs. 4 [1.9%] vs. 3 [1.4%]), and lost to follow-up (11 [5.2%] vs. 7 [3.3%] vs. 9 [4.3%]). The median OS was significantly prolonged in group B compared to group C (26.8 [95% CI 21.2 to 30.9] months vs. 20.3 [95% CI 16.2 to 24.6] months), reducing the risk of death by 34% (HR = 0.66, 95% CI 0.52 to 0.83; *P* < 0.001). The estimated OS rate at 36 months was 39.9% (95% CI 33.1 to 46.5) in group B and 30.9% (95% CI 24.6 to 37.4) in group C, while OS rate at 48 months was 34.0% (95% CI 27.0% to 41.1%) in group B and 16.2% (95% CI 10.8% to 22.5%) in group C (Fig. 3A). By the time of the final survival analysis, 79 (37.6%) patients in group C had crossed over to serplulimab plus HLX04 treatment. The median OS in group C adjusted by the 2-stage estimation model was 14.2 (95% CI 11.9 to 17.0) months, resulting in an adjusted HR of 0.53 (95% CI 0.42 to 0.68; *P* < 0.001) for group B versus group C, while that adjusted by the RPSFTM was 17.9 (95% CI 14.2 to 20.3) months, resulting in an adjusted HR of 0.65 (95% CI 0.51 to 0.83; *P* < 0.001) for group B compared to group C (Table S3). An additional 43 (20.5%) patients in group C received subsequent immunotherapy, excluding serplulimab, following their first disease progression. Median OS in group C adjusted for crossing over and subsequent immunotherapy using the RPSFTM model was 16.4 (95% CI 13.2 to 19.4) months, resulting in an adjusted HR of 0.62 (95% CI 0.49 to 0.80; *P* < 0.001) for group B compared to group C (Table S3). Subgroup analysis of OS showed different extents of benefit for group B compared with group C across most exploratory subgroups, including those with PD-L1 TPS ≥ 50% (HR = 0.49, 95% CI 0.30 to 0.79). A trend toward improved median OS was also observed in patients with brain metastasis (HR = 0.67, 95% CI 0.40 to 1.11) (Fig. 3B).

There was no further improvement in median OS in group A compared with group B (23.7 months vs. 26.8 months, HR = 1.12, 95% CI 0.88 to 1.42; *P* = 0.363) (Fig. 3A). The 48-month OS rate was 30.0% (95% CI 23.6% to 36.5%) in group A and 34.0% (95% CI 27.0% to 41.1%) in group B. Subgroup analysis revealed no statistically significant improvement in median OS in group A compared with group B across all exploratory subgroups (Fig. 3C).

#### Tumor response

At the final survival analysis, ORR assessed by BICR in group B was higher than that in group C (52.8% vs. 27.6%; OR = 2.85, 95% CI 1.91 to 4.26; *P* < 0.001), with 111 (51.9%) patients achieving partial response in group B compared to 56 (26.7%) in group C. BICR-assessed median DoR was also prolonged in group B compared to group C (15.4 [95% CI, 11.1 to 23.7] months vs. 8.3 [95% CI, 5.5 to 12.5] months, HR = 0.52, 95% CI 0.35 to 0.78; *P* = 0.001) (Table 2).

**Table 2.** Summary of tumor response assessed by BICR in the ITT population

Tumor response	Group A (*n* = 212)	Group B (*n* = 214)	Group C (*n* = 210)
Best overall response, *n* (%)			
CR	5 (2.4)	2 (0.9)	2 (1.0)
PR	110 (51.9)	111 (51.9)	56 (26.7)
SD	69 (32.5)	72 (33.6)	95 (45.2)
PD	13 (6.1)	19 (8.9)	37 (17.6)
NE	15 (7.1)	10 (4.7)	20 (9.5)
Objective response*, n*	115	113	58
ORR (%, 95% CI)	54.2 (47.3–61.1)	52.8 (45.9–59.7)	27.6 (21.7–34.2)
OR ^a^ (95% CI)	1.05 (0.72–1.55)	2.85 (1.91–4.26)	NE
*P* value ^a^	0.788	< 0.001	NE
DoR, median (95% CI), months	16.0 (11.2–19.5)	15.4 (11.1–23.7)	8.3 (5.5–12.5)
HR ^a^ (95% CI)	1.11 (0.79–1.55)	0.52 (0.35–0.78)	NE
*P* value ^a^	0.553	0.001	NE

Group A, serplulimab plus HLX04 and chemotherapy; Group B, serplulimab plus HLX04 placebo and chemotherapy; Group C, serplulimab placebo plus HLX04 placebo and chemotherapy; BICR, blinded independent central review; CI, confidence interval; CR, complete response; DoR, duration of response; HR, hazard ratio; ITT, intent-to-treat; NE, not evaluable; OR, odds ratio; ORR, objective response rate; PD, progressive disease; PR, partial response; SD, stable disease.

^a^ Group A was statistically compared to Group B, while Group B was compared to Group C.

Consistent with the interim analysis [20], no significant improvement in tumor response was observed in group A compared to group B. The BICR-assessed ORR was 54.2% (95% CI 47.3 to 61.1) and 52.8% (95% CI 45.9 to 59.7) for the respective groups (OR = 1.05, 95% CI 0.72 to 1.55; *P* = 0.788). Median DoR was not significantly improved in group A compared to group B (16.0 [95% CI, 11.2 to 19.5] months vs. 15.4 [95% CI, 11.1 to 23.7] months, HR = 1.11, 95% CI 0.79 to 1.55; *P* = 0.553) (Table 2). Investigator-assessed tumor responses and DoR were consistent with those of the evaluation by BICR (Table S4). Investigator-assessed PFS, OS, and tumor response in the PPS were consistent with those of the ITT population (Fig. S2 and Table S5).

### Safety

The summary of AEs for the SS population (*n* = 634) at the final survival analysis is presented in Table S6. At the final survival analysis, treatment-related adverse events (TRAEs) were reported in 98.6% (*n* = 208) of patients in group A, 99.1% (*n* = 212) in group B, and 98.6% (*n* = 206) in group C. Grade ≥3 TRAEs occurred in 71.6% (*n* = 151), 67.8% (*n* = 145), and 56.9% (*n* = 119) of patients in groups A, B, and C, respectively (Table S6). The most common grade ≥3 TRAEs included neutrophil count decreased (50.2% [*n* = 106] in group A, 44.4% [*n* = 95] in group B, and 35.9% [*n* = 75] in group C), white blood cell decreased (27.0% [*n* = 57] in group A, 22.9% [*n* = 49] in group B, and 19.6% [*n* = 41] in group C), anemia (20.4% [*n* = 43] in group A, 22.4% [*n* = 48] in group B, and 19.6% [*n* = 41] in group C), and platelet count decreased (19.0% [*n* = 40] in group A, 14.5% [*n* = 31] in group B, and 15.3% [*n* = 32] in group C) (Table 3). A total of 41.2% (*n* = 87) of patients in group A, 39.7% (*n* = 85) in group B, and 24.9% (*n* = 52) in group C experienced serious TRAEs (Table S6). The incidence of TRAEs leading to treatment discontinuation was 24.6% (*n* = 52) in group A, 15.9% (*n* = 34) in group B, and 7.2% (*n* = 15) in group C. TRAEs leading to death occurred in 4.7% (*n* = 10), 2.3% (*n* = 5), and 2.9% (*n* = 6) of patients in groups A, B, and C, respectively (Table S6).

**Table 3.** TRAEs in the SS population.

Safety	Group A (*n* = 211)			Group B (*n* = 214)			Group C (*n* = 209)		
Events	Grades 1–2	Grades 3–4	Grade 5	Grades 1–2	Grades 3–4	Grade 5	Grades 1–2	Grades 3–4	Grade 5
Any TRAEs, *n* (%)	57 (27.0)	141 (66.8)	10 (4.7)^a^	67 (31.3)	140 (65.4)	5 (2.3) ^a^	87 (41.6)	112 (53.6)	6 (2.9)^a^
TRAEs with an incidence of ≥15% in any group ^b^, *n* (%)									
Anemia	119 (56.4)	43 (20.4)	0 (0.0)	111 (51.9)	48 (22.4)	0 (0.0)	120 (57.4)	41 (19.6)	0 (0.0)
AST increased	113 (53.6)	5 (2.4)	0 (0.0)	93 (43.5)	7 (3.3)	0 (0.0)	74 (35.4)	3 (1.4)	0 (0.0)
WBC count decreased	107 (50.7)	57 (27.0)	0 (0.0)	121 (56.5)	49 (22.9)	0 (0.0)	113 (54.1)	40 (19.1)	1 (0.5)
ALT increased	100 (47.4)	5 (2.4)	0 (0.0)	103 (48.1)	9 (4.2)	0 (0.0)	77 (36.8)	6 (2.9)	0 (0.0)
Nausea	84 (39.8)	1 (0.5)	0 (0.0)	82 (38.3)	3 (1.4)	0 (0.0)	65 (31.1)	3 (1.4)	0 (0.0)
Platelet count decreased	82 (38.9)	40 (19.0)	0 (0.0)	75 (35.0)	31 (14.5)	0 (0.0)	67 (32.1)	31 (14.8)	1 (0.5)
Decreased appetite	78 (37.0)	6 (2.8)	0 (0.0)	72 (33.6)	3 (1.4)	0 (0.0)	41 (19.6)	3 (1.4)	0 (0.0)
Proteinuria	68 (32.2)	6 (2.8)	0 (0.0)	36 (16.8)	0 (0.0)	0 (0.0)	7 (3.3)	0 (0.0)	0 (0.0)
Neutrophil count decreased	66 (31.3)	106 (50.2)	0 (0.0)	80 (37.4)	95 (44.4)	0 (0.0)	85 (40.7)	74 (35.4)	1 (0.5)
Asthenia	64 (30.3)	11 (5.2)	0 (0.0)	55 (25.7)	5 (2.3)	0 (0.0)	38 (18.2)	4 (1.9)	0 (0.0)
Constipation	59 (28.0)	1 (0.5)	0 (0.0)	51 (23.8)	0 (0.0)	0 (0.0)	40 (19.1)	0 (0.0)	0 (0.0)
Hypoalbuminemia	56 (26.5)	0 (0.0)	0 (0.0)	52 (24.3)	1 (0.5)	0 (0.0)	38 (18.2)	0 (0.0)	0 (0.0)
Vomiting	49 (23.2)	2 (0.9)	0 (0.0)	51 (23.8)	2 (0.9)	0 (0.0)	43 (20.6)	4 (1.9)	0 (0.0)
GGT increased	42 (19.9)	5 (2.4)	0 (0.0)	37 (17.3)	9 (4.2)	0 (0.0)	20 (9.6)	4 (1.9)	0 (0.0)
Blood creatinine increased	40 (19.0)	2 (0.9)	0 (0.0)	29 (13.6)	0 (0.0)	0 (0.0)	14 (6.7)	0 (0.0)	0 (0.0)
Hypercholesterolemia	37 (17.5)	0 (0.0)	0 (0.0)	32 (15.0)	2 (0.9)	0 (0.0)	19 (9.1)	0 (0.0)	0 (0.0)
Weight decreased	34 (16.1)	0 (0.0)	0 (0.0)	22 (10.3)	2 (0.9)	0 (0.0)	17 (8.1)	2 (1.0)	0 (0.0)
Hypokalemia	33 (15.6)	5 (2.4)	0 (0.0)	21 (9.8)	3 (1.4)	0 (0.0)	12 (5.7)	4 (1.9)	0 (0.0)
Hypertension	32 (15.2)	15 (7.1)	0 (0.0)	4 (1.9)	5 (2.3)	0 (0.0)	2 (1.0)	0 (0.0)	0 (0.0)
Diarrhea	27 (12.8)	6 (2.8)	0 (0.0)	13 (6.1)	4 (1.9)	0 (0.0)	13 (6.2)	1 (0.5)	0 (0.0)
Lymphocyte count decreased	26 (12.3)	14 (6.6)	0 (0.0)	24 (11.2)	6 (2.8)	0 (0.0)	23 (11.0)	8 (3.8)	0 (0.0)

Group A, serplulimab plus HLX04 and chemotherapy; Group B, serplulimab plus HLX04 placebo and chemotherapy; Group C, serplulimab placebo plus HLX04 placebo and chemotherapy; ALT, alanine aminotransferase; AST, aspartate aminotransferase; GGT, gamma-glutamyltransferase; SS, safety set; TRAE, treatment-related adverse event; WBC, white blood cell. TRAE refers to any adverse event as assessed by the investigator to be related, likely related, or unknown in relation to the study drugs.

^a^ Ten patients in group A experienced grade 5 adverse events of epilepsy (*n* = 1), myocarditis (*n* = 1), cardiac failure (*n* = 1), pneumonitis (*n* = 1), interstitial lung disease (*n* = 1), myocardial infarction (*n* = 1), completed suicide (*n* = 1), sudden cardiac death (*n* = 1), septic shock (*n* = 1), and multiple organ dysfunction syndrome (*n* = 1). Five patients in group B experienced grade 5 toxicities of pneumonia (*n* = 1), respiratory failure (*n* = 1), plasma cell myeloma (*n* =1), and death (*n* = 2). Six patients in group C experienced grade 5 adverse events of tumor lysis syndrome (*n* = 1), death (*n* = 1), myocardial infarction (*n* = 1), WBC count decreased, neutrophil count decreased, platelet count decreased and gastrointestinal infection (*n* = 1), cardiovascular disorder (*n* =1), and soft tissue infection (*n* = 1).

^b^ For grade 3 to 4 events, TRAEs with an incidence of 5% or higher are listed.

By the time of final survival analysis, 32.2% (*n* = 68) of patients in group A, 31.8% (*n* = 68) in group B, and 12.9% (*n* = 27) in group C reported immune-related adverse events (irAEs) that were predominantly mild in severity (Table S7). Some of the most common irAEs included hypothyroidism (9.5% [*n* = 20] in group A, 9.3% [*n* = 20] in group B, and 2.9% [*n* = 6] in group C), blood thyroid-stimulating hormone increased (4.7% [*n* = 10] in group A, 4.2% [*n* = 9] in group B, and 1.0% [*n* = 2] in group C), and hyperthyroidism (4.3% [*n* = 9] in group A, 7.9% [*n* = 17] in group B, and 1.0% [*n* = 2] in group C) (Table S7). Serious irAEs occurred in 12.3% (*n* = 26), 10.3% (*n* = 22), and 1.4% (*n* = 3) of patients in groups A, B, and C, respectively (Table S6).

## Discussion

This final survival analysis of the phase III ASTRUM-002 study of first-line serplulimab and chemotherapy, with or without bevacizumab biosimilar HLX04, in patients with advanced nsq-NSCLC confirmed the significant improvements in PFS and tumor response with serplulimab plus chemotherapy compared to chemotherapy alone with a longer follow-up duration, as previously reported in the interim analysis [20]. The exploratory subgroup analyses of PFS suggest the long-term benefit with serplulimab plus chemotherapy compared to chemotherapy alone, especially in patients with PD-L1 TPS ≥ 50%, and regardless of the presence of brain metastasis, disease stage, age, sex, tobacco use history, and Eastern Cooperative Oncology Group performance status score at baseline.

OS, the key secondary endpoint, results of ASTRUM-002 were herein reported for the first time. Serplulimab plus chemotherapy conferred a significantly longer median OS compared to chemotherapy alone. Currently, the estimated percentage of patients who were alive at 48 months was 34.0% (95% CI 27.0% to 41.1%) in the serplulimab plus chemotherapy group and 16.2% (95% CI 10.8% to 22.5%) in the chemotherapy alone group, demonstrating a long-term survival benefit with the addition of serplulimab. Exploratory subgroup analyses of OS further demonstrated a consistent improvement across most patient subgroups, including for those with brain metastasis (HR = 0.67, 95% CI 0.40 to 1.11). An indirect comparison with other approved PD-1/PD-L1 inhibitors revealed a comparable OS improvement with serplulimab when combined with pemetrexed and platinum-based chemotherapy for the same indication (serplulimab, HR = 0.66, 95% CI 0.52 to 0.83; pembrolizumab, HR = 0.60, 95% CI 0.50 to 0.72 [23]; cemiplimab, HR = 0.65, 95% CI 0.51 to 0.82 [24]; sugemalimab, HR = 0.68, 95% CI 0.54 to 0.85 [25]; toripalimab, HR = 0.69, 95% CI 0.57 to 0.93 [26]) as well as PFS benefit (serplulimab, HR = 0.54, 95% CI 0.43 to 0.68; pembrolizumab, HR = 0.50, 95% CI 0.42 to 0.60 [23]; cemiplimab, HR = 0.55, 95% CI 0.44 to 0.68 [24]; sugemalimab, HR = 0.49, 95% CI 0.39 to 0.60 [25]; toripalimab, HR = 0.49, 95% CI 0.39 to 0.61 [27]). Notably, the median OS of patients with high PD-L1 expression (PD-L1 TPS ≥50%) treated with serplulimab plus chemotherapy was 45.4 (95% CI 26.1 months to not reached [NR]) months compared with 27.7 (95% CI 20.4 to 38.2) months in patients treated with pembrolizumab plus chemotherapy [23]. At the time of the final survival analysis, 79 (37.6%) patients in the chemotherapy alone group (group C) had crossed over to serplulimab plus HLX04 treatment and another 43 (20.5%) patients had received subsequent antitumor immunotherapy excluding serplulimab; adjusted HR revealed a more pronounced OS benefit in the serplulimab plus chemotherapy group compared to the chemotherapy alone group (adjusted HR by RPSFTM: 0.62 [95% CI 0.49 to 0.80]; *P* < 0.001), thereby confirming the benefit of combining serplulimab with chemotherapy as first-line treatment compared to chemotherapy alone.

The lack of a further improvement in survival benefits, including median PFS, median OS, and tumor response with the addition of HLX04 to serplulimab plus chemotherapy compared to serplulimab plus chemotherapy, was also confirmed in this final survival analysis. ASTRUM-002 is the first randomized study to directly compare the efficacy and safety of PD-1 inhibitor plus chemotherapy with or without an anti-VEGF mAb. In line with the results from the IMpower151 and APPLE trials [7,8], these findings add to the body of evidence suggesting a lack of enhanced efficacy with this tri-combination treatment strategy for advanced nsq-NSCLC, regardless of *EGFR*, *ALK*, or *ROS1* oncogenic alterations compared to doublet chemotherapy plus PD-1/PD-L1 mAb.

Compared with the interim analysis [20], there were no new safety signals and no major changes to the safety profiles with a longer follow-up duration at this final survival analysis. Safety findings for the serplulimab plus chemotherapy group were largely comparable to that of the other investigated first-line PD-1/PD-L1 mAbs plus chemotherapy for this indication [23,28,29]. The increased AEs with HLX04 and serplulimab plus chemotherapy group as compared to the serplulimab plus chemotherapy group clearly indicated a less desirable benefit–risk ratio for this treatment regimen.

Although the 2-stage model and RPSFTM have their limitations, such as the assumption of a constant effect of the investigated treatment regimen across patients and over time for the latter model [30] and difficulty in identifying covariates that were imbalanced between patients who switched treatment and those who did not [21] that could affect the outcome of these approaches, they are widely adopted and well-established methodologies for the analysis of survival data whereby treatment switching occurs for patients. Other limitations of this study included an all-Chinese patient population that could limit the applicability of these findings to patients of different demographics and the selection of PD-L1 CPS as a stratification factor instead of the validated PD-L1 TPS for immuno-oncology in NSCLC. Additionally, the real-time PCR assay employed to detect *EGFR*, *ALK*, and *ROS1* oncogenic alterations in this study was restricted to known fusion variants and, thus, may not capture rare or novel *ALK*/*ROS1* rearrangements. Nonetheless, these uncommon variants constitute only a small subset of *ALK* or *ROS1* rearrangement-positive NSCLC cases [31,32] and are therefore unlikely to have a meaningful impact on the overall study population and the study’s conclusions.

## Conclusions

In summary, the final survival analysis of the ASTRUM-002 study demonstrated the sustained clinical benefits of serplulimab plus chemotherapy compared to chemotherapy alone as first-line treatment for patients with locally advanced or metastatic nsq-NSCLC without *EGFR* or *ALK/ROS1* genetic alterations. The safety profile of the serplulimab plus chemotherapy regimen was manageable with a longer follow-up duration. The updated results also provided clear evidence for the lack of superiority for the addition of bevacizumab biosimilar HLX04 to serplulimab plus chemotherapy over serplulimab plus chemotherapy for this indication.

## Ethical Approval

The study was conducted in accordance with the principles of the Declaration of Helsinki, International Council for Harmonization Guideline for Good Clinical Practice, and local regulatory requirements. The study protocol, any amendments, and informed consent were approved by the central or independent institutional review board/ethics committee at each participating hospital. Written informed consent was obtained from each patient or each patient’s guardian before enrolment. This trial is registered on ClinicalTrials.gov (NCT03952403).

## Data Availability

Individual deidentified patient data (including data dictionaries) from this study are not available. Study protocol and statistical analysis plan are provided as supplementary data. The data supporting the findings of this study are available within the article and its Supplementary Materials. Additional data from this study are available upon reasonable request from the corresponding author.
