# Supplementary material for: Serplulimab Plus Chemotherapy, with or without HLX04, versus Chemotherapy as First-Line Treatment for Nonsquamous NSCLC: Final Survival Analysis of the Phase III ASTRUM-002 Study
Source: Cancer Commun (Lond). 2026 Jun 10;46:0034. doi: 10.34133/cancomm.0034 (PMC13250280; doi:10.34133/cancomm.0034)
Supplement: Supplementary 1 — Figs. S1 and S2 Tables S1 to S7 Data S1 and S2 [file cancomm.0034.f1.zip › CANCOMM-D-26-00023-Supplementary-Final.docx]

**Supplementary material for**

**Serplulimab Plus Chemotherapy, With or Without HLX04, Versus Chemotherapy as First-line Treatment for Non-squamous Non-Small-Cell Lung Cancer: Final Survival Analysis of a Randomized, Double-blind, Multicenter Phase III ASTRUM-002 study**

Running title: Final Survival for Serplulimab and chemotherapy with or without HLX04 in nsq-NSCLC

Xuezhi Hao^1,†^, Lin Wang^1,†^, Yanrong Hao^2^, Yanping Hu^3^, Chun Chen^4^, Bi Chen^5^, Yunchao Huang^6^, Aimin Zang^7^, Yan Wang^8^, Zhendong Chen^9^, Wu Zhuang^10^, Jinsheng Shi^11^, Xiubao Ren^12^, Ligong Nie^13^, Guohua Yu^14^, Feng Luo^15^, Yimin Mao^16^, Xiang Wang^17^, Baolan Li^18^, Yuansong Bai^19^, Jianhua Shi^20^, Hongyan Ni^21^, Xiaoli Hou^21^, Haoyu Yu^21^, Jing Li^21^, Qingyu Wang^21^, Jun Zhu^21^, Yuankai Shi^1,*^, the ASTRUM-002 Study Group

1. Department of Medical Oncology, Beijing Key Laboratory of Key Technologies for Early Clinical Trial Evaluation of Innovative Drugs for Major Diseases, National Cancer Center/National Clinical Research Center for Cancer/Cancer Hospital, Chinese Academy of Medical Sciences & Peking Union Medical College, Beijing, P. R. China
2. Department of Medical Oncology, The People’s Hospital of Guangxi Zhuang Autonomous Region, Nanning, Guangxi Zhuang Autonomous Region, P. R. China
3. Department of Medical Oncology, Hubei Cancer Hospital, Wuhan, Hubei, P. R. China
4. Department of Thoracic Surgery, Fujian Medical University Union Hospital, Fuzhou, Fujian, P. R. China
5. Department of Respiratory and Critical Care Medicine, The Affiliated Hospital of Xuzhou Medical University, Xuzhou, Jiangsu, P. R. China
6. Department of Thoracic Surgery I, Yunnan Cancer Hospital, Kunming, Yunnan, P. R. China
7. Department of Medical Oncology, Affiliated Hospital of Hebei University, Baoding, Hebei, P. R. China
8. Department of Respiratory Medicine, Harbin Medical University Cancer Hospital, Harbin, Heilongjiang, P. R. China
9. Department of Medical Oncology, The Second Affiliated Hospital of Anhui Medical University, Hefei, Anhui, P. R. China
10. Department of Thoracic Oncology, Fujian Cancer Hospital, Fuzhou, Fujian, P. R. China
11. Department of Oncology, Cangzhou People’s Hospital, Cangzhou, Hebei, P. R. China
12. Department of Biotherapy, Tianjin Medical University Cancer Institute & Hospital, Tianjin, P. R. China
13. Department of Respiratory and Critical Care Medicine, Peking University First Hospital, Beijing, P. R. China
14. Department of Medical Oncology, Weifang People’s Hospital, Weifang, Shandong, P. R. China
15. Lung Cancer Center, West China Hospital of Sichuan University, Chengdu, Sichuan, P. R. China
16. Department of Respiratory and Critical Care Medicine, The First Affiliated Hospital of Henan University of Science and Technology, Luoyang, Henan, P. R. China
17. Department of Medical Oncology, Xuzhou Central Hospital, Xuzhou, Jiangsu, P. R. China
18. Department of Medical Oncology, Beijing Chest Hospital, Capital Medical University, Beijing, P. R. China
19. Department of Oncology and Hematology, China-Japan Union Hospital of Jilin University, Changchun, Jilin, P. R. China
20. Department of Medical Oncology, Linyi Cancer Hospital, Linyi, Shandong, P. R. China
21. Global Product Development, Shanghai Henlius Biotech, Inc., Shanghai, P. R. China

**^*^Address correspondence to:** Y.S. (Yuankai Shi), [syuankai@cicams.ac.cn](mailto:syuankai@cicams.ac.cn)

^†^**These authors contributed equally to this work**

Supplementary Table S1. The ASTRUM-002 study group members and the ethics approval numbers

| **Investigators** | **Site** | **Ethics approval number** | **Number of patients enrolled** |
| --- | --- | --- | --- |
| Yanrong Hao | The People's Hospital of Guangxi Zhuang Autonomous Region | Ethics-2019-40 (1) | 30 |
| Yanping Hu | Hubei Cancer Hospital | (2019) No. 38 | 24 |
| Chun Chen | Union Hospital Affiliated to Fujian Medical University | 2019YW067 | 23 |
| Bi Chen | The Affiliated Hospital of Xuzhou Medical University | XYFY2020-YL071-01 | 22 |
| Yunchao Huang | Yunnan Cancer Hospital | YW201938 | 20 |
| Aimin Zang | The Affiliated Hospital of Hebei University | HDFY-LL-2020-055 | 18 |
| Yan Wang | Harbin Medical University - Tumor Hospital (The Third Affiliated Hospital) | 2020-76 | 18 |
| Zhendong Chen | The Second Affiliated Hospital of Anhui Medical University | Ethics No. PJ-YW2019-066(F1) | 17 |
| Wu Zhuang | Fujian Cancer Hospital | 2019-088 | 17 |
| Jinsheng Shi | Cangzhou People's Hospital | 2019 Approval-16 | 17 |
| Xiubao Ren | Tianjin Medical University Cancer Institute and Hospital | E2019465 | 17 |
| Ligong Nie | Peking University First Hospital | (2019) Drug Registration No. (60) | 16 |
| Guohua Yu | Weifang People's Hospital | 2020-005 | 16 |
| Feng Luo | West China Hospital of Sichuan University | 2019 Clinical Trial (Western Med) No. 146 | 16 |
| Yimin Mao | The First Affiliated Hospital of Henan University of Science and Technology | 2019-0058 | 15 |
| Xiang Wang | Xuzhou Central Hospital | XZXY-YL-2019089-048 | 15 |
| Baolan Li | Beijing Chest Hospital, Capital Medical University | (2019) Clinical Review No. (61) | 14 |
| Yuansong Bai | China-Japan Friendship Hospital of Jilin University | (2019) Clinical Review No. 2019061806 | 13 |
| Jianhua Shi | Linyi Cancer Hospital | XY1944 | 13 |
| Kejing Ying | SIR RUN RUN SHAW HOSPITAL | Drug Clinical Trial 20190806-7 | 12 |
| Guoping Sun | The First Affiliated Hospital of China University of Science and Technology | Ethics No. YJ 2020-02-01 | 11 |
| Zhengguo Li  Dongji Chen | Gansu Wuwei Tumour Hospital | 2019 Ethics Review-09 | 11 |
| Jinsheng Wu | The First Affiliated Hospital of Hainan Medical University | 2019 (Drug) No. 27 | 11 |
| Yuankai Shi (principal investigator), Xuezhi Hao (sub-investigator), Lin Wang (sub-investigator) | National Cancer Center/National Clinical Research Center for Cancer/Cancer Hospital, Chinese Academy of Medical Sciences & Peking Union Medical College | 19/067-1852 | 10 |
| Chuan Jin | The Affiliated Cancer Hospital and Institute of Guangzhou Medical University | Initial Ethics Review [2019] No. 21 | 10 |
| Haifeng Lin | The Second Affiliated Hospital of Hainan Medical University | 2019D010-F01 | 10 |
| Xingya Li | The First Affiliated Hospital of Zhengzhou University | Drug-2019-101 | 10 |
| Hongmei Sun | Jiamusi Cancer Hospital | 2020 Ethics Opinion-02 | 10 |
| Peiguo Cao  Xi Zhang | The Third Xiangya Hospital of Central South University | 20056 | 9 |
| Liangming Zhang | Yantai Yuhuangding Hospital | Ethics Review for Drugs/Devices [2019] No. 22 | 9 |
| Xicheng Wang | The First Affiliated Hospital of Guangdong Pharmaceutical University | Ethics Review [2020] No. 106 | 9 |
| Xueli Yuan | The Fourth affiliated hospital of Harbin Medical University | SY2019-031 | 8 |
| Tangfeng Lv | Chinese People's Liberation Army Eastern Theater Command General Hospital | 2020NZYW-006-01 | 8 |
| Yinghua Ji | The First Affiliated Hospital of Xinxiang Medical University | [2021] Ethics Drug Review No. 19 | 8 |
| Jian Fang | Beijing Cancer Hospital | 2019YW132 | 7 |
| Guangyu An | Beijing Chao-yang Hospital, Capital Medical University | 2019-Drug-12-6 | 7 |
| Yueyin Pan | The First Affiliated Hospital of Anhui Medical University | 2019 Ethics Review No. 287 | 7 |
| Lin Wu | Hunan Cancer Hospital | Hunan Cancer Hospital Ethics Committee 2019 Drug Review No. 199 | 7 |
| Jiuwei Cui | The First Hospital of Jilin University | 19Y125-001 | 7 |
| Wei Zhang | The First Affiliated Hospital of Nanchang University | [2019] Clinical Ethics Review No. 095 | 7 |
| Jianying Zhou | The First Affiliated Hospital Zhejiang University School of Medicine | 2019 Ethics Review No. 235 | 7 |
| Xiaohong Wu | The Affiliated Hospital of Jiangnan University | (2020) Ethics Review No. 15 | 7 |
| Qingshan Li | The Affiliated Hospital of Chengde Medical University | LL2021 (Drug) 012 | 7 |
| Jun Liang | Peking University International Hospital | 2019-0018 | 6 |
| Hui Luo | Jiangxi Cancer Hospital | 2019015-YW015 | 6 |
| Huiwen Ma | Chongqing University Cancer Hospital | CZLS2021123-A | 6 |
| Bangwei Cao | Beijing Friendship Hospital, Capital Medical University | 2019-P1-Drug034 | 5 |
| Jingchang Li | Liuzhou General Hospital | 2020-006-01 | 5 |
| Rui Meng | Wuhan Union Hospital | [2021] Ethics Review No. 0193 | 5 |
| Xi Chen | 900TH Hospital of Joint Logistics Support Force | IEC-2019-086 | 4 |
| Fen Wang | Peking University Shenzhen Hospital | Ethics Review [2019] No. (009) | 4 |
| Yong Li | The First Affiliated Hospital of Nanchang University | [2019] Clinical Ethics Review No. 094 | 4 |
| Yiping Zhang | Zhejiang Cancer Hospital | IRB-[2020]500 | 4 |
| Xiaoping Huang | Chongqing University Three Gorges Hospital | (2019) Ethics Review No. 023 | 4 |
| Rui Ma | Liaoning Cancer Hospital | 20200827 | 4 |
| Zhiyong Ma | Henan Cancer Hospital | 2019091202 | 3 |
| Yan Tan | Jilin Province People's Hospital | 2019-Y-018 | 3 |
| Wen Li | The Second Affiliated Hospital of Zhejiang University School of Medicine | (2019) Ethics Drug Review No. 491 | 3 |
| Youlun Li | The First Affiliated Hospital of Chongqing Medical University | 2021 Ethics Review (CY20216401) | 3 |
| Na Li | Suining Central Hospital | 2021 Ethics Opinion-sy009-01 | 3 |
| Hui Zhao | The Second Affiliated Hospital of Anhui Medical University | Ethics No. PJ-YW2019-065(F1) | 2 |
| Haixin Huang | Liuzhou Worker's Hospital | PJ201919 | 2 |
| Zhongyao Jia | Linyi People's Hospital | 2019 Ethics Review-16 | 2 |
| Jun Chen | Tianjin Medical University General Hospital | IRB2019-121-01 | 2 |
| Jianhua Chang | Cancer Hospital Chinese Academy of Medical Sciences, Shenzhen Center | YW2021-13 | 2 |
| Meili Sun | Jinan Central Hospital | Clinical Ethics Review 2021-055-01 | 2 |
| Youxin Ji | Qingdao Central Hospital | [Y]SY202101801 | 2 |
| Zhitu Zhu | The First Affiliated Hospital of Jinzhou Medical University | 202108-01 | 2 |
| Aihong Zhong | Fuzhou Tuberculosis Control Hospital of Fujian Province | 2019-007 (Drug Research)-01 | 1 |
| Yarong Li | The Second Hospital of Jilin University | (2020) Clinical Review No. (04) | 1 |
| Liyan Jiang | Shanghai Chest Hospital | LS2041 | 1 |
| Dongqing Lv | Taizhou Hospital of Zhejiang Province | 202127-01 | 1 |

Supplementary Table S2. Subsequent systemic anti-tumor treatment

| **Subsequent therapy^a^** | **Group A**  **(*n* = 212)** | **Group B**  **(*n* = 214)** | **Group C**  **(*n* = 210)** |
| --- | --- | --- | --- |
| Patients with anti-tumor immunotherapy after first disease progression, *n* (%) | 95 (44.8) | 102 (47.7) | 137 (65.2) |
| Crossed over to serplulimab plus HLX04, *n* (%) | NA | NA | 79 (37.6) |
| Subsequent immunotherapy following the end of study drug treatment, *n* (%) | 32 (15.1) | 42 (19.6) | 43 (20.5) |
| Therapy type, *n* (%) | | | |
| Single-agent chemotherapy | 11 (5.2) | 8 (3.7) | 3 (1.4) |
| Multiple chemotherapy | 5 (2.4) | 5 (2.3) | 5 (2.4) |
| Single-agent ICI | 4 (1.9) | 2 (0.9) | 4 (1.9) |
| ICI and chemotherapy | 10 (4.7) | 7 (3.3) | 5 (2.4) |
| ICI and anti-angiogenesis therapy | 4 (1.9) | 4 (1.9) | 47 (22.4) |
| ICI and targeted therapy^b^ | 0 (0.0) | 1 (0.5) | 0 (0.0) |
| ICI and anti-angiogenesis therapy and chemotherapy | 9 (4.2) | 26 (12.1) | 38 (18.1) |
| ICI and chemotherapy and targeted therapy^b^ | 2 (0.9) | 0 (0.0) | 0 (0.0) |
| ICI and anti-angiogenesis therapy and targeted therapy^b^ | 0 (0.0) | 0 (0.0) | 3 (1.4) |
| ICI and anti-angiogenesis therapy and targeted therapy^b^ and chemotherapy | 3 (1.4) | 2 (0.9) | 7 (3.3) |
| Anti-angiogenesis therapy | 6 (2.8) | 10 (4.7) | 5 (2.4) |
| Anti-angiogenesis therapy and chemotherapy | 22 (10.4) | 27 (12.6) | 9 (4.3) |
| Anti-angiogenesis therapy and targeted therapy^b^ | 0 (0.0) | 2 (0.9) | 2 (1.0) |
| Anti-angiogenesis therapy and targeted therapy^b^ and chemotherapy | 6 (2.8) | 1 (0.5) | 2 (1.0) |
| Targeted therapy^b^ | 8 (3.8) | 2 (0.9) | 6 (2.9) |
| Targeted therapy^b^ and chemotherapy | 2 (0.9) | 2 (0.9) | 1 (0.5) |
| Other^c^ | 3 (1.4) | 3 (1.4) | 0 (0.0) |

^a^Some patients received more than one line of subsequent anti-tumor therapies (crossed over or not).

^b^Targeted therapy included inhibitors for less common gene mutations such as *KRAS*, *BRAF*, and *MEK* alterations.

^c^Included traditional Chinese medicine.

Group A, serplulimab plus HLX04 and chemotherapy; Group B, serplulimab plus HLX04 placebo and chemotherapy; Group C, serplulimab placebo plus HLX04 placebo and chemotherapy. **Abbreviations:** ICI, immune checkpoint inhibitors; NA, not applicable.

Supplementary Table S3. Overall survival after adjusting for patient cross over and having received subsequent immunotherapy

| **Overall survival** | **Group A (*n* = 212)** | **Group B (*n* = 214)** | **Group C (*n* = 210)** |
| --- | --- | --- | --- |
| Adjusting for patient cross over with the TSE model | | | |
| OS, median (95% CI), months | 23.7 (20.5–27.5) | 26.8 (21.2–30.9) | 14.2 (11.9–17.0) |
| HR^a^ (95% CI) | 1.12 (0.88–1.42) | 0.53 (0.42–0.68) | NE |
| *P*-value^a^ | 0.363 | < 0.001 | NE |
| Adjusting for patient cross over with the RPSFTM model | | | |
| OS, median (95% CI), months | 23.7 (20.5–27.5) | 26.8 (21.2–30.9) | 17.9 (14.2–20.3) |
| HR^a^ (95% CI) | 1.12 (0.88–1.42) | 0.65 (0.51–0.83) | NE |
| *P*-value^a^ | 0.363 | < 0.001 | NE |
| Adjusting for patient cross over and subsequent immunotherapy with the RPSFTM model | | | |
| OS, median (95% CI), months | 23.7 (20.5–27.5) | 26.8 (21.2–30.9) | 16.4 (13.2–19.4) |
| HR^a^ (95% CI) | 1.12 (0.88–1.42) | 0.62 (0.49–0.80) | NE |
| *P*-value^a^ | 0.363 | < 0.001 | NE |

^a^ Group A was statistically compared to Group B, while Group B was compared to Group C.

Group A, serplulimab plus HLX04 and chemotherapy; Group B, serplulimab plus HLX04 placebo and chemotherapy; Group C, serplulimab placebo plus HLX04 placebo and chemotherapy. **Abbreviations:** CI, confidence interval; HR, hazard ratio; NE, not evaluable; OS, overall survival; RPSFTM, rank-preserving structural failure time model; TSE, two-stage estimation.

Supplementary Table S4. Summary of tumor response assessed by the investigators in the ITT population

| **Tumor response** | **Group A (*n* = 212)** | **Group B (*n* = 214)** | **Group C (*n* = 210)** |
| --- | --- | --- | --- |
| Best overall response, *n* (%) | | | |
| CR | 0 (0.0) | 0 (0.0) | 1 (0.5) |
| PR | 121 (57.1) | 114 (53.3) | 59 (28.1) |
| SD | 67 (31.6) | 73 (34.1) | 105 (50.0) |
| PD | 11 (5.2) | 19 (8.9) | 27 (12.9) |
| NE | 13 (6.1) | 8 (3.7) | 18 (8.6) |
| Objective response, *n* | 121 | 114 | 60 |
| ORR*,* (%, 95% CI) | 57.1 (50.1–63.8) | 53.3 (46.4–60.1) | 28.6 (22.6–35.2) |
| OR^a^ (95% CI) | 1.17 (0.79–1.74) | 2.85 (1.90–4.26) | NE |
| *P*-value^a^ | 0.437 | < 0.001 | NE |
| DoR, median (95% CI), months | 15.2 (11.1–20.3) | 15.6 (11.3–22.5) | 10.9 (8.3–13.8) |
| HR^a^ (95% CI) | 1.01 (0.74–1.38) | 0.59 (0.40–0.87) | NE |
| *P*-value^a^ | 0.936 | 0.006 | NE |

^a^ Group A was statistically compared to Group B, while Group B was compared to Group C.

Group A, serplulimab plus HLX04 and chemotherapy; Group B, serplulimab plus HLX04 placebo and chemotherapy; Group C, serplulimab placebo plus HLX04 placebo and chemotherapy. **Abbreviations:** CI, confidence interval; CR, complete response; DoR, duration of response; HR, hazard ratio; ITT, intention-to-treat; NE, not evaluable; OR, odds ratio; ORR, objective response rate; PD, progressive disease; PR, partial response; SD, stable disease.

Supplementary Table S5. Summary of tumor response assessed by the BICR and investigators in the PPS population

| **Tumor response** | **BICR-assessed** | | | **Investigator-assessed** | | |
| --- | --- | --- | --- | --- | --- | --- |
|  | **Group A**  **(*n* = 202)** | **Group B**  **(*n* = 208)** | **Group C**  **(*n* = 195)** | **Group A**  **(*n* = 202)** | **Group B**  **(*n* = 208)** | **Group C**  **(*n* = 195)** |
| **Best overall response, *n* (%)** | | | | | | |
| CR | 5 (2.5) | 2 (1.0) | 2 (1.0) | 0 | 0 | 1 (0.5) |
| PR | 110 (54.5) | 111 (53.4) | 56 (28.7) | 121 (59.9) | 114 (54.8) | 59 (30.3) |
| SD | 68 (33.7) | 71 (34.1) | 95 (48.7) | 65 (32.2) | 72 (34.6) | 105 (53.8) |
| PD | 13 (6.4) | 19 (9.1) | 37 (19.0) | 11 (5.4) | 19 (9.1) | 27 (13.8) |
| NE | 6 (3.0) | 5 (2.4) | 5 (2.6) | 5 (2.5) | 3 (1.4) | 3 (1.5) |
| Objective response, *n* | 115 | 113 | 58 | 121 | 114 | 60 |
| ORR*,* (%, 95% CI) | 56.9 (49.8–63.9) | 54.3 (47.3–61.2) | 29.7 (23.4–36.7) | 59.9 (52.8–66.7) | 54.8 (47.8–61.7) | 30.8 (24.4–37.8) |
| OR^a^ (95% CI) | 1.12 (0.75–1.66) | 2.72 (1.81–4.09) | NE | 1.26 (0.84–1.90) | 2.72 (1.81–4.09) | NE |
| *P*-value^a^ | 0.577 | < 0.001 | NE | 0.267 | < 0.001 | NE |
| DoR, median (95% CI), months | 16.0 (11.2–19.5) | 15.4 (11.1–23.7) | 8.3 (5.5–12.5) | 15.2 (11.1–20.3) | 15.6 (11.3–22.5) | 10.9 (8.3–13.8) |
| HR^a^ (95% CI) | 1.11 (0.79–1.55) | 0.52 (0.35–0.78) | NE | 1.01 (0.74–1.38) | 0.59 (0.40–0.87) | NE |
| *P*-value^a^ | 0.553 | 0.001 | NE | 0.936 | 0.006 | NE |

^a^ Group B was statistically compared to Group C, while Group A was compared to Group B.

Group A, serplulimab plus HLX04 and chemotherapy; Group B, serplulimab plus HLX04 placebo and chemotherapy; Group C, serplulimab placebo plus HLX04 placebo and chemotherapy. **Abbreviations:** BICR, blinded independent central review; CI, confidence interval; CR, complete response; DoR, duration of response; HR, hazard ratio; NE, not evaluable; OR, odds ratio; ORR, objective response rate; PD, progressive disease; PPS, per-protocol set; PR, partial response; SD, stable disease.

Supplementary Table S6. Safety summary in the SS population^a^

| **Events** | **Group A**  **(*n* = 211)** | **Group B**  **(*n* = 214)** | **Group C**  **(*n* = 209)** |
| --- | --- | --- | --- |
| Any AEs | 210 (99.5%) | 212 (99.1%) | 208 (99.5%) |
| Grade ≥3 | 170 (80.6%) | 157 (73.4%) | 143 (68.4%) |
| Any TRAEs^b^ | 208 (98.6%) | 212 (99.1%) | 206 (98.6%) |
| Grade ≥3 | 151 (71.6%) | 145 (67.8%) | 119 (56.9%) |
| Serious AEs | 117 (55.5%) | 106 (49.5%) | 88 (42.1%) |
| Serious TRAEs^b^ | 87 (41.2%) | 85 (39.7%) | 52 (24.9%) |
| AEs leading to treatment withdrawal^b^ | 57 (27.0%) | 39 (18.2%) | 26 (12.4%) |
| TRAEs leading to treatment withdrawal | 52 (24.6%) | 34 (15.9%) | 15 (7.2%) |
| AEs leading to death | 33 (15.6%) | 21 (9.8%) | 30 (14.4%) |
| TRAEs leading to death^c^ | 10 (4.7%)^d^ | 5 (2.3%)^d^ | 6 (2.9%)^d^ |
| Any AESIs | 71 (33.6%) | 68 (31.8%) | 29 (13.9%) |
| Any irAEs | 68 (32.2%) | 68 (31.8%) | 27 (12.9%) |
| Serious irAEs | 26 (12.3%) | 22 (10.3%) | 3 (1.4%) |
| Infusion-related reactions | 3 (1.4%) | 0 (0.0%) | 1 (0.5%) |
| Serious infusion-related reactions | 0 (0.0%) | 0 (0.0%) | 1 (0.5%) |

^a^ One patient each in group A and group C did not receive any study treatment and were therefore excluded from safety analyses.

^b^ The incidence rates of TRAEs, serious TRAEs, and AEs leading to treatment withdrawal are for any study drug.

^c^ Excluding death from disease progression.

^d^ Ten patients in group A experienced grade 5 adverse events of epilepsy (*n* = 1), myocarditis (*n* = 1), cardiac failure (*n* = 1), pneumonitis (*n* = 1), interstitial lung disease (*n* = 1), myocardial infarction (*n* = 1), completed suicide (*n* = 1), sudden cardiac death (*n* = 1), septic shock (*n* = 1), and multiple organ dysfunction syndrome (*n* = 1). Five patients in group B experienced grade 5 toxicities of pneumonia (*n* = 1), respiratory failure (*n* = 1), plasma cell myeloma (*n* =1), and death (*n* = 2). Six patients in group C experienced grade 5 adverse events of tumor lysis syndrome (*n* = 1), death (*n* = 1), myocardial infarction (*n* = 1), WBC count decreased, neutrophil count decreased, platelet count decreased and gastrointestinal infection (*n* = 1), cardiovascular disorder (*n* =1), and soft tissue infection (*n* = 1).

Group A, serplulimab plus HLX04 and chemotherapy; Group B, serplulimab plus HLX04 placebo and chemotherapy; Group C, serplulimab placebo plus HLX04 placebo and chemotherapy.

**Abbreviations:** AE, adverse event; AESI, adverse event of special interest; irAE, immune-related adverse event; SS, safety set; TRAE, treatment-related adverse event.

Supplementary Table S7. Summary of irAEs in the SS population

|  | **Group A (*n* = 211)** | | | | **Group B (*n* = 214)** | | | **Group C (*n* = 209)** | | | |
| --- | --- | --- | --- | --- | --- | --- | --- | --- | --- | --- | --- |
| **Events** | Grade 1-2 | Grade 3-4 | Grade 5 | Grade 1-2 | | Grade 3-4 | Grade 5 | | Grade 1-2 | Grade 3-4 | Grade 5 |
| Any irAEs, *n* (%) | 48 (22.7) | 17 (8.1) | 3 (1.4) | 54 (25.2) | | 14 (6.5) | 0 (0.0) | | 26 (12.4) | 1 (0.5) | 0 (0.0) |
| irAEs with an incidence of ≥1% in any group^a^, *n* (%) | | | | | | | | | | | |
| Hypothyroidism | 19 (9.0) | 1 (0.5) | 0 (0.0) | 20 (9.3) | | 0 (0.0) | 0 (0.0) | | 6 (2.9) | 0 (0.0) | 0 (0.0) |
| Blood TSH increased | 10 (4.7) | 0 (0.0) | 0 (0.0) | 9 (4.2) | | 0 (0.0) | 0 (0.0) | | 2 (1.0) | 0 (0.0) | 0 (0.0) |
| Hyperthyroidism | 9 (4.3) | 0 (0.0) | 0 (0.0) | 17 (7.9) | | 0 (0.0) | 0 (0.0) | | 2 (1.0) | 0 (0.0) | 0 (0.0) |
| Rash | 8 (3.8) | 1 (0.5) | 0 (0.0) | 3 (1.4) | | 0 (0.0) | 0 (0.0) | | 5 (2.4) | 0 (0.0) | 0 (0.0) |
| Immune-mediated lung disease | 5 (2.4) | 2 (0.9) | 0 (0.0) | 6 (2.8) | | 4 (1.9) | 0 (0.0) | | 2 (1.0) | 0 (0.0) | 0 (0.0) |
| Pneumonitis | 4 (1.9) | 0 (0.0) | 0 (0.0) | 2 (0.9) | | 0 (0.0) | 0 (0.0) | | 1 (0.5) | 0 (0.0) | 0 (0.0) |
| AST increased | 2 (0.9) | 1 (0.5) | 0 (0.0) | 3 (1.4) | | 0 (0.0) | 0 (0.0) | | 1 (0.5) | 0 (0.0) | 0 (0.0) |
| NT-proBNP increased | 2 (0.9) | 1 (0.5) | 0 (0.0) | 2 (0.9) | | 0 (0.0) | 0 (0.0) | | 1 (0.5) | 0 (0.0) | 0 (0.0) |
| Chronic kidney disease | 2 (0.9) | 1 (0.5) | 0 (0.0) | 0 (0.0) | | 0 (0.0) | 0 (0.0) | | 0 (0.0) | 0 (0.0) | 0 (0.0) |
| Blood TSH decreased | 2 (0.9) | 0 (0.0) | 0 (0.0) | 3 (1.4) | | 0 (0.0) | 0 (0.0) | | 0 (0.0) | 0 (0.0) | 0 (0.0) |
| Immune-mediated myocarditis | 1 (0.5) | 2 (0.9) | 0 (0.0) | 1 (0.5) | | 0 (0.0) | 0 (0.0) | | 0 (0.0) | 0 (0.0) | 0 (0.0) |
| ALT increased | 1 (0.5) | 1 (0.5) | 0 (0.0) | 2 (0.9) | | 1 (0.5) | 0 (0.0) | | 3 (1.4) | 0 (0.0) | 0 (0.0) |
| Interstitial lung disease | 1 (0.5) | 0 (0.0) | 1 (0.5) | 2 (0.9) | | 1 (0.5) | 0 (0.0) | | 0 (0.0) | 0 (0.0) | 0 (0.0) |
| Thyroxine increased | 1 (0.5) | 0 (0.0) | 0 (0.0) | 7 (3.3) | | 0 (0.0) | 0 (0.0) | | 2 (1.0) | 0 (0.0) | 0 (0.0) |
| T3 free increased | 1 (0.5) | 0 (0.0) | 0 (0.0) | 5 (2.3) | | 0 (0.0) | 0 (0.0) | | 1 (0.5) | 0 (0.0) | 0 (0.0) |
| T3 increased | 1 (0.5) | 0 (0.0) | 0 (0.0) | 4 (1.9) | | 0 (0.0) | 0 (0.0) | | 0 (0.0) | 0 (0.0) | 0 (0.0) |
| Myocarditis | 0 (0.0) | 0 (0.0) | 1 (0.5) | 1 (0.5) | | 0 (0.0) | 0 (0.0) | | 0 (0.0) | 0 (0.0) | 0 (0.0) |
| Epilepsy | 0 (0.0) | 0 (0.0) | 1 (0.5) | 0 (0.0) | | 0 (0.0) | 0 (0.0) | | 0 (0.0) | 0 (0.0) | 0 (0.0) |
| Troponin I increased | 0 (0.0) | 0 (0.0) | 0 (0.0) | 4 (1.9) | | 1 (0.5) | 0 (0.0) | | 0 (0.0) | 0 (0.0) | 0 (0.0) |
| Adrenal insufficiency | 0 (0.0) | 0 (0.0) | 0 (0.0) | 3 (1.4) | | 0 (0.0) | 0 (0.0) | | 0 (0.0) | 0 (0.0) | 0 (0.0) |
| Thyroxine free increased | 0 (0.0) | 0 (0.0) | 0 (0.0) | 2 (0.9) | | 0 (0.0) | 0 (0.0) | | 2 (1.0) | 0 (0.0) | 0 (0.0) |
| Supraventricular extrasystoles | 0 (0.0) | 0 (0.0) | 0 (0.0) | 0 (0.0) | | 0 (0.0) | 0 (0.0) | | 2 (1.0) | 0 (0.0) | 0 (0.0) |

^a^For grade 3-4 events, irAEs with an incidence of ≥1% are listed. All grade 5 events are listed.

Group A, serplulimab plus HLX04 and chemotherapy; Group B, serplulimab plus HLX04 placebo and chemotherapy; Group C, serplulimab placebo plus HLX04 placebo and chemotherapy. **Abbreviations:** ALT, alanine aminotransferase; AST, aspartate aminotransferase; irAE, immune-related adverse event; NT-proBNP, N-terminal prohormone brain natriuretic peptide; SS, safety set; TSH, thyroid-stimulating hormone; T3, tri-iodothyronine.


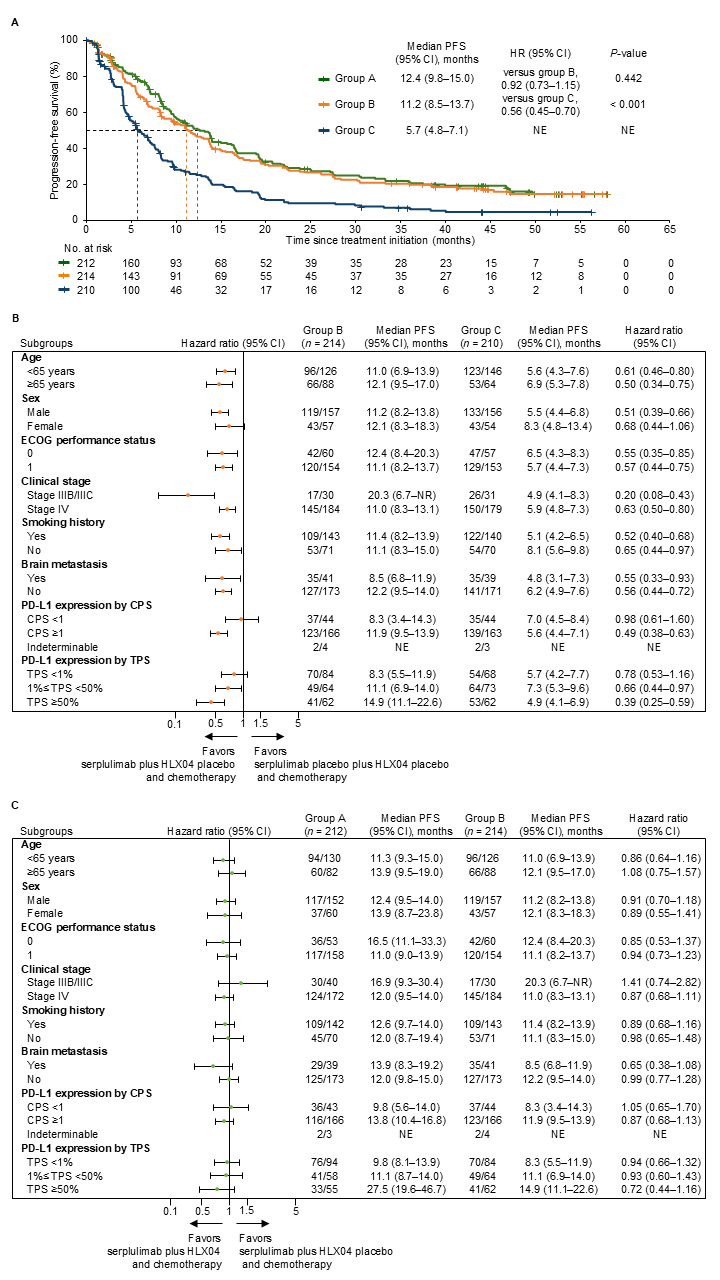


Supplementary Figure S1. Kaplan‐Meier survival curve and subgroup analysis of investigator-assessed progression-free survival, showing the progression-free survival benefits with serplulimab plus chemotherapy compared to chemotherapy alone in the intent-to-treat population and across subgroups.

(**A**) In the ITT population. The median PFS was 12.4 (95% CI 9.8–15.0) months in group A, 11.2 (95% CI 8.5–13.7) months in group B, and 5.7 (95% CI 4.8–7.1) months in group C. Serplulimab plus chemotherapy significantly improved PFS compared with chemotherapy alone, with an HR of 0.56 (95% CI 0.45–0.70; *P* < 0.001). The addition of HLX04 did not translate to an improvement in PFS compared with serplulimab plus chemotherapy (HR = 0.92, 95% CI 0.73–1.15; *P* = 0.442). (**B**) Exploratory subgroup analysis of PFS in group B vs. group C, suggesting a PFS benefit with serplulimab plus chemotherapy over chemotherapy alone across the various patient subgroups.  (**C**) Exploratory subgroup analysis of PFS in group A vs. group B, implying a lack of PFS benefit with HLX04 and serplulimab plus chemotherapy over serplulimab plus chemotherapy across the various patient subgroups. Group A, serplulimab plus HLX04 and chemotherapy; Group B, serplulimab plus HLX04 placebo and chemotherapy; Group C, serplulimab placebo plus HLX04 placebo and chemotherapy. **Abbreviations:** CI, confidence interval; CPS, combined positive score; ECOG, Eastern Cooperative Oncology Group; HR, hazard ratio; ITT, intent-to-treat; NE, not evaluable; NR, not reached; PD–L1, programmed cell death ligand 1; PFS, progression-free survival; TPS, tumor proportion score.


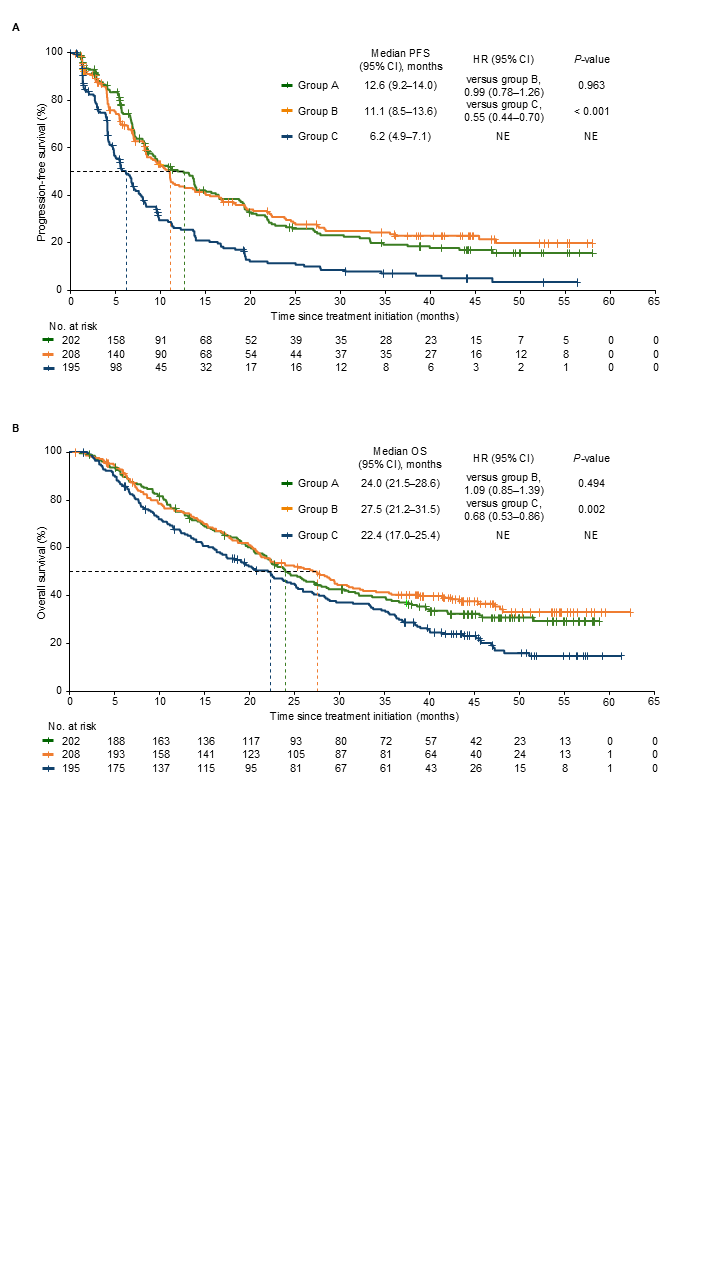


Supplementary Figure S2. Kaplan-Meier survival curve of progression-free survival and overall survival in the per-protocol set.

(**A**) BICR-assessed PFS. The median PFS was 12.6 (95% CI, 9.2–14.0) months in group A, 11.1 (95% CI, 8.5–13.6) months in group B, and 6.2 (95% CI, 4.9–7.1) months in group C. Serplulimab plus chemotherapy significantly improved PFS compared with chemotherapy alone, with an HR of 0.55 (95% CI, 0.44–0.70; *P* < 0.001). The addition of HLX04 did not translate to an improvement in PFS compared with serplulimab plus chemotherapy (HR 0.99, 95% CI, 0.78–1.26; *P* = 0.963). (**B**) OS. The median OS was 24.0 (95% CI, 21.5–28.6) months in group A, 27.5 (95% CI, 21.2–31.5) months in group B, and 22.4 (95% CI, 17.0–25.4) months in group C. Serplulimab plus chemotherapy significantly improved OS compared with chemotherapy alone, with an HR of 0.68 (95% CI, 0.53–0.86; *P* = 0.002). The addition of HLX04 did not translate to an OS benefit compared with serplulimab plus chemotherapy (HR 1.09, 95% CI, 0.85–1.39; *P* = 0.494). Group A, serplulimab plus HLX04 and chemotherapy; Group B, serplulimab plus HLX04 placebo and chemotherapy; Group C, serplulimab placebo plus HLX04 placebo and chemotherapy. **Abbreviations:** CI, confidence interval; HR, hazard ratio; NE, not evaluable; OS, overall survival; PFS, progression-free survival; PPS, per-protocol set.
