# Supplementary material for: Serplulimab Plus Chemotherapy, with or without HLX04, versus Chemotherapy as First-Line Treatment for Nonsquamous NSCLC: Final Survival Analysis of the Phase III ASTRUM-002 Study
Source: Cancer Commun (Lond). 2026 Jun 10;46:0034. doi: 10.34133/cancomm.0034 (PMC13250280; doi:10.34133/cancomm.0034)
Supplement: Supplementary 1 — Figs. S1 and S2 Tables S1 to S7 Data S1 and S2 [file cancomm.0034.f1.zip › Supplementary Data S1-Final.pdf]

A Three-Arm, Randomized, Double-Blind, Multicenter, Phase III Clinical Study to Evaluate HLX10 (Recombinant Humanized Anti-PD-1 Monoclonal Antibody Injection) in Combination with Chemotherapy (Carboplatin-Pemetrexed) Versus HLX10 + HLX04 (Recombinant Anti-VEGF Humanized Monoclonal Antibody Injection) in Combination with Chemotherapy (Carboplatin- Pemetrexed) Versus Chemotherapy (Carboplatin- Pemetrexed) as First-Line Treatment of Advanced Non-Squamous Non-Small Cell Lung Cancer (NSCLC)

PROTOCOL

**Investigational product**	HLX10, HLX04
**NMPA Approval No.**	2018L02201/2018L03039/2018L02998
**Protocol Number**	HLX10-002-NSCLC301
**Sponsor**	Shanghai Henlius Biotech, Inc.
**Leading Site**	Cancer Hospital, Chinese Academy of Medical Sciences
**Principal Investigator**	Professor Yuankai Shi
**Version No.**	V7.0
**Version Date**	28 Nov. 2022

Confidentiality Statement

It is hereby declared that all the contents of this protocol are proprietary to Shanghai Henlius Biotech, Inc. and shall not be forwarded or copied without permission to anyone other than the investigator/Ethics Committee/relevant authorities and personnel authorised by the Sponsor.

Signature Page for Principal Investigator (Leading Site)

I have read and become familiar with this protocol, confirmed the schedule in the protocol and the necessary contents including the implementation, and clearly understood the responsibilities related to this trial protocol. I agree to and will perform my duties in strict accordance with the laws and regulations of China, Declaration of Helsinki, Good Clinical Practice, and this protocol.

Leading site: Cancer Hospital, Chinese Academy of Medical Sciences

______________________________________ ____________________________
Professor Yuankai Shi (signature) Date (dd/mm/yyyy)

Signature Page for Principal Investigator (Participating Site)

I have read and become familiar with this protocol, confirmed the schedule in the protocol and the necessary contents including the implementation, and clearly understood the responsibilities related to this trial protocol. I agree to and will perform my duties in strict accordance with the laws and regulations of China, Declaration of Helsinki, Good Clinical Practice, and this protocol.

____________________ Principal investigator (printed name)	____________________ Principal investigator (signature)
____________________ Study site	____________________ Date (dd/mm/yyyy)

Signature Page for Sponsor

I have read and been familiar with the protocol, confirmed the study plan and such necessary content related to study implementation as included in the protocol, and clarified the responsibilities of the investigators related to the protocol. I agree to and will perform my duties in strict accordance with the laws and regulations of China, Declaration of Helsinki, Good Clinical Practice, and this protocol.

Sponsor: Shanghai Henlius Biotech, Inc.

Kang Wenying Medical Executive Director (signature)	Date (dd/mm/yyyy)
Cheng Jiancheng, Executive Director of Biostatistics (signature)	Date (dd/mm/yyyy)

Table of Contents

Signature Page for Principal Investigator (Leading Site) **2**

Signature Page for Principal Investigator (Participating Site) **3**

Signature Page for Sponsor **4**

List of Tables **10**

List of Figures **10**

Glossary of Abbreviations **11**

Synopsis **15**

Schedule of Study Procedure **32**

1. INTRODUCTION **37**

1.1 Disease Background **37**

1.2 Research Development and Basis for Dose Selection **37**

1.2.1 Overview of HLX10 and HLX04 37

1.2.2 Non-clinical studies on HLX10 38

1.2.3 Non-clinical studies of HLX04 43

1.2.4 Evaluation of anti-tumor efficacy of HLX10 combined with HLX04 in the model of NOD/SCID mice 47

1.2.5 Clinical studies on HLX10 and HLX04 48

1.2.6 Rationale for selection of chemotherapy as control group 49

1.2.7 Rationale for combined treatment 49

1.2.8 Rationale for HLX10 and HLX04 dose selection 50

1.3 Assessment for Risks and Benefits **51**

1.3.1 Potential benefits 51

1.3.2 Identified and potential risks 52

1.3.3 Overall benefits: risk and ethics review 53

2. OBJECTIVES **54**

2.1. Stage I: A Single-Arm Study (Safety Run-In Phase) **54**

2.2. Stage II: Phase III Study **54**

3. STUDY DESIGN **54**

3.1. Overall Study Design **54**

3.2. Endpoints **56**

3.2.1 Stage I endpoints 56

3.2.2 Stage II endpoints 56

3.3. Randomization, Blinding, and Unblinding ... 57
3.3.1 Emergency unblinding ... 57
3.3.2 Unblinding for treatment after disease progression ... 58
3.4. Number of Subjects ... 58
3.5. Selection and Withdrawal of Subjects ... 58
3.5.1 Inclusion criteria ... 58
3.5.2 Exclusion criteria ... 60
3.5.3 Criteria for discontinuation ... 63
3.6. Loss to follow-up ... 64
3.7. End of Study ... 64
3.8. Early Termination of Study/Closure of Study Site ... 65
3.9. Study Treatment ... 65
3.9.1 Study drugs ... 65
3.9.2 Route of administration and dosage ... 66
3.9.3 Dose modification ... 68
3.9.4 Principles for HLX10 or placebo dose modification ... 68
3.9.5 Principles for HLX04 or placebo dose modification ... 69
3.9.6 Principles for chemotherapy dose modifications ... 70
3.9.7 Packaging and labelling ... 73
3.9.8 Storage, management, and dispensing ... 73
3.9.9 Concomitant and prohibited therapies ... 74
3.9.10 Treatment compliance ... 75
4. STUDY PROCEDURES AND VISITS ... 75
4.1. Study Procedures ... 75
4.1.1 Demographics and medical history ... 75
4.1.2 Prior and concomitant medications ... 76
4.1.3 Adverse event ... 76
4.1.4 Quality of life assessment ... 76
4.1.5 EQ-5D-5L ... 76
4.1.6 EORTC QLQ-C30 ... 76
4.1.7 EORTC QLQ-LC13 ... 77
4.1.8 Echocardiography ... 77
4.1.9 12-ECG ... 77

4.1.10 Complete physical examination 77
4.1.11 Symptom-directed physical examination 77
4.1.12 Height, weight and vital signs 78
4.1.13 ECOG score 78
4.1.14 Local laboratory tests 78
4.1.15 PK and ADA blood sampling 79
4.1.16 EGFR/ALK/ROS1 DNA mutation status confirmation 80
4.1.17 Radiological examination 80
4.1.18 Biomarker test 80
4.2. Screening Period (Day -28 to -1) 81
4.3. Treatment Period 82
4.3.1 Treatment visits prior to progressive disease (first PD) 82
4.3.2 Treatment visits after progressive disease (first PD) 86
4.4. End-of-Treatment Visit 88
4.5. Follow-Up Period 89
4.5.1 Safety follow-up period 89
4.5.2 Survival follow-up period 90
4.6. Study Assessments 90
4.6.1 Efficacy assessment 91
4.6.2 Safety assessment 91
4.7. Adverse Event 91
4.7.1 Definition of AE 91
4.7.2 Adverse event of special interest (AESI) 92
4.7.3 Definition of serious adverse event (SAE) 94
4.7.4 Liver function test outliers meeting Hy's Law 96
4.7.5 Documentation of AE 96
4.7.6 Severity evaluation 98
4.7.7 Causality assessment 99
4.7.8 Expectedness Judgement 100
4.7.9 Disease progression 100
4.7.10 Newly developed primary tumor 100
4.7.11 Death 100
4.7.12 Reporting of AE 100

4.7.13 Overdose 101

4.7.14 Pregnancy 101

4.8. Sample Size Estimation 102

4.9. Statistical Analysis Sets 103

4.9.1 Intent-to-treat (ITT) set 103

4.9.2 Per protocol set (PPS) 103

4.9.3 Safety set (SS) 103

4.9.4 Pharmacokinetic set (PKS) 103

4.10. Interim Analysis 103

4.11. Statistical Analysis Methods 104

4.11.1 Demographics, medical history, and baseline characteristics 105

4.11.2 Medication compliance 105

4.11.3 Efficacy analysis 105

4.11.4 Stage I: safety run-in phase 105

4.11.5 Stage II: phase III study 105

4.11.6 Safety Analysis 106

4.11.7 Pharmacokinetics and immunogenicity analysis 106

4.11.8 Biomarker analysis 107

4.11.9 Analysis of patient-reported outcomes 107

5. ETHICS 107

5.1. Ethical Requirements 107

5.2. Informed Consent 108

5.3. Subject Confidentiality 108

6. DATA MANAGEMENT 108

6.1. Database Setup 108

6.2. Data Check 108

6.3. Database Lock 109

7. STUDY MANAGEMENT 109

7.1. Quality Control and Quality Assurance 109

7.1.1 Training 110

7.1.2 Clinical monitoring 110

7.1.3 Audit 110

7.1.4 Data Management/Coding 111

7.1.5 Missing and useless data 111

7.2. Documentation and Retention of Study Data 111

7.3. Follow-ups and Medical Measures After Study Completion 112

8. RESPONSIBILITIES 112

8.1. Responsibilities of the Investigator 112

8.2. Responsibilities of the Sponsor 113

9. CONFIDENTIALITY AND PUBLICATION OF TRIAL RESULTS 114

References 115

10. APPENDICES 117

Appendix 1: Common Terminology Criteria for Adverse Events 117

Appendix 2: Response Evaluation Criteria in Solid Tumors (RECIST 1.1) 118

Appendix 3: Quality of Life Scale EORTC QLQ-C30, EQ-5D-5L, EORTC QLQ-LC13 EORTC QLQ-C30 (3rd Edition) 124

Appendix 4: Eastern Cooperative Oncology Group (ECOG) - Performance Status Scale 129

Appendix 5: Fridericia's Correction Formula 130

Appendix 6: New York Heart Association Classification 131

Appendix 7: Prohibited Traditional Chinese Medicines during the Trial 132

List of Tables

Table 1 Basic principles for HLX10 or placebo dose modification 69
Table 2 Basic principles for HLX04 or placebo dose modification 69
Table 3 Basic principles for dose modifications of carboplatin and pemetrexed 70
Table 4 Principles for dose modifications of carboplatin and pemetrexed for hematological toxicities 71
Table 5 Carboplatin dose modifications based on non-hematological toxicities in previous cycles 72
Table 6 Pemetrexed dose modifications based on non-hematological toxicities in previous cycles 72
Table 7 Local laboratory tests 78
Table 8 PK and ADA blood sampling 79
Table 9 Treatment of infusion reaction 93
Table 10 Liver function test outliers that should be reported as an SAE 96
Table 11 Scale for assessment of causality between AE and study drugs 99

List of Figures

Figure 1 Study design schematic 30
Figure 2 Efficacy of HLX04 combined with HLX10 in the mouse tumor model 48
Figure 3 Schematic of study treatment 84

Glossary of Abbreviations

Abbreviation	Explanation
ADA	Anti-drug antibody
AE	Adverse event
ALT	Alanine aminotransferase
ANC	Neutrophils
APTT	Activated partial thromboplastin time
ASCO	American Society of Clinical Oncology
AST	Aspartate aminotransferase
BUN	Blood urea nitrogen
CHO	Chinese hamster ovary cells
CI	Confidence interval
CMH	Cochran-Mantel-Haenszel test
CNS	Central nervous system
CR	Complete response
Cr	Creatinine
CRA	Clinical research associate
CRO	Contract research organisation
CSCO	Chinese Society of Clinical Oncology, China Anti-Cancer Association
CSF	Colony-stimulating factor
CT	Computerised tomography
CTCAE	Common Terminology Criteria for Adverse Events
CTLA-4	Cytotoxic T-lymphocyte antigen 4
DILI	Drug-induced liver injury
DOR	Duration of response
ECOG	Eastern Cooperative Oncology Group
eCRF	Electronic case report form
EDC	Electronic data collection
EORTC QLQ-C30	European Organization for Research and Treatment of Cancer Quality of Life Questionnaire Core 30
EQ-5D-5L	5-level EQ-5D version
FDA	Food and Drug Administration
FDG-PET	Fluorodeoxyglucose positron emission tomography
FFPE	Formalin-fixed paraffin embedded
FT3	Free triiodothyronine

Abbreviation	Explanation
FT4	Free thyroxine
GCP	Good Clinical Practice
Hb	Haemoglobin
HBcAb	Hepatitis B core antibody
HBsAg	Hepatitis B surface antigen
HBV	Hepatitis B virus
HCV	Hepatitis C virus
HIV	Human immunodeficiency virus
HR	Hazard ratio
HRQoL	Health-related quality of life
ICF	Informed consent form
ICH	International Council for Harmonisation
INR	International normalized ratio
IDMC	Independent Data Monitoring Committee
irAEs	Immune-related adverse events
IRB	Institutional Review Board
IRRC	Independent Radiology Review Committee
ITT	Intent-to-treatment set
IV	Intravenous infusion
IVIG	Intravenous immunoglobulin
IWRS	Interactive web response system
LFT	Liver function test
LVEF	Left ventricular ejection fraction
MedDRA	Medical Dictionary for Regulatory Activities
MRI	Magnetic resonance imaging
MRT	Mean residence time
MSI	Microsatellite instability
MTD	Maximum tolerated dose
NA	Not applicable
NCCN	National comprehensive cancer network
NE	Not evaluable
NMPA	National Medical Products Administration
NSAID	Nonsteroidal anti-inflammatory drug
NSCLC	Non-small cell lung cancer

Abbreviation	Explanation
NYHA	New York Heart Association
ORR	Objective response rate
OS	Overall survival
PCP	Pneumocystis pneumonia
PD	Progressive disease
PD-1	Programmed cell death protein 1
PD-L1	Programmed cell death-ligand 1
PET-CT	Positron emission tomography-computed tomography
PFS	Progression-free survival
PI	Principal investigator
PK	Pharmacokinetics
PKS	PK set
PLT	Platelet
PPS	Per protocol set
PR	Partial response
QC	Quality control
RECIST	Response Evaluation Criteria in Solid Tumors
RO	Receptor occupancy
RSI	Safety information
SAE	Serious adverse event
SAP	Statistical analysis plan
SC	Steering Committee
SD	Stable disease
SOP	Standard operating procedure
SS	Safety set
T3	Triiodothyronine
T4	Thyroxine
TBIL	Total bilirubin
TEAE	Treatment emergent adverse event
TMB	Tumor mutation burden
TNF-α	Tumor necrosis factor α
TSH	Thyroid-stimulating hormone
ULN	Upper limit of normal

Abbreviation	Explanation
US	Ultrasound
VEGF	Vascular Endothelial Growth Factor
WHO-DDE	World Health Organization Drug Dictionary Enhanced

Synopsis

**Investigational product**	HLX10 (recombinant humanized anti-PD-1 monoclonal antibody injection)<br>HLX04 (recombinant anti-VEGF humanized monoclonal antibody injection)
**Title**	A Three-Arm, Randomized, Double-Blind, Multicenter, Phase III Clinical Study to Evaluate HLX10 (Recombinant Humanized Anti-PD-1 Monoclonal Antibody Injection) in Combination with Chemotherapy (Carboplatin-Pemetrexed) Versus HLX10 + HLX04 (Recombinant Anti-VEGF Humanized Monoclonal Antibody Injection) in Combination with Chemotherapy (Carboplatin-Pemetrexed) Versus Chemotherapy (Carboplatin-Pemetrexed) as First-Line Treatment of Advanced Non- Squamous Non-Small Cell Lung Cancer (NSCLC)
**Protocol No.**	HLX10-002-NSCLC301
**Sponsor**	Shanghai Henlius Biotech, Inc.
**Trial phase**	Registered clinical phase III study
**Study sites**	Approximately 80 study sites in China
**Subjects**	Patients with advanced non-squamous non-small cell lung cancer (NSCLC)
**Objectives**	**Stage I: a single-arm study (safety run-in phase)**<br>**Primary objective:**<br>- To evaluate the safety and tolerability of HLX10 + HLX04 combined with chemotherapy as first-line treatment in patients with advanced non-squamous non-small cell lung cancer.<br>**Secondary objective:**<br>- To evaluate the clinical efficacy of HLX10 + HLX04 combined with chemotherapy as first-line treatment in patients with advanced non-squamous non-small cell lung cancer.<br>**Stage II: phase III study**<br>**Primary objective:**<br>- To evaluate the clinical efficacy of HLX10 combined with chemotherapy versus HLX10 + HLX04 combined with chemotherapy as first-line treatment in patients with advanced non-squamous non-small cell lung cancer.<br>**Secondary objective:**<br>- To evaluate the safety and tolerability of HLX10 combined with chemotherapy versus HLX10 + HLX04 combined with chemotherapy as first-line treatment in patients with advanced non-squamous non-small cell lung cancer.

**Study period**	This study includes the following stages: screening (28 days), treatment (until loss of clinical benefit, death, intolerable toxicity, withdrawal of informed consent, or occurrence of other reasons specified in the protocol, whichever occurs first), and follow-up period (including safety follow-up period and survival follow-up period).
**Study design**	This study aims to evaluate the clinical efficacy, safety, and tolerability of HLX10 combined with chemotherapy versus HLX10 + HLX04 combined with chemotherapy versus chemotherapy in subjects who have not previously received systemic treatment for advanced non-squamous NSCLC, collect PK parameters, and explore the biomarkers related to efficacy.<br>This study has two stages:<br>***The first stage is a single-arm study and a safety run-in phase.***<br>The subjects will be treated with HLX10 + HLX04 combined with chemotherapy (carboplatin-pemetrexed).<br>Approximately 6–12 subjects with advanced non-squamous NSCLC will be enrolled in this stage. Approximately 6 subjects will be enrolled for the first time. After all subjects complete the first cycle of study treatment, the safety and tolerability will be confirmed by the Steering Committee (SC) to determine whether to proceed to the second stage of phase III randomized, double-blind, multicenter study. If, in the opinion of the committee, safety and tolerability cannot be determined, approximately 6 additional subjects will be enrolled for safety and tolerability evaluation.<br>The safety evaluation will be conducted in accordance with the following criteria, and any of the following events shall be recorded as "safety events":<br>✓ Grade ≥ 4 hamatological toxicity:<br>✧ Grade ≥ 4 neutropenia;<br>✧ Grade ≥ 4 thrombocytopenia;<br>✧ Other grade ≥ 4 hematological toxicities.<br>✓ Grade ≥ 3 non-hematological toxicity:<br>✧ Grade ≥ 3 nausea, vomiting, fatigue, rash, and diarrhea, not recovered to grade ≤ 2 within 7 days;<br>✧ Grade ≥ 3 injection site AE;<br>✧ Grade ≥ 3 cardiac dysfunction, not recovered to grade ≤ 2 within 7 days;<br>✧ Grade ≥ 3 acute immunotherapy-related AE or organ damage;<br>✧ Other grade ≥ 3 non-hematological toxicities (except alopecia, electrolyte imbalance, and other reactions with no clinical significance), not recovered to grade ≤ 2 within 7 days.<br>✓ Pneumonia (any grade ≥ 2 pneumonia, not recovered to grade ≤ 1 within 3 days).

	If "safety events" occur in ≤ 1 of 6 subjects, it will be considered as good safety, and the second stage can be proceeded to. If "safety events" occur in ≥ 2 of 6 subjects, 6–12 subjects will be enrolled additionally. If "safety events" occur in < 4 of the 12 subjects, the second stage can be proceeded to; if "safety events" occur in ≥ 4 of the 12 subjects, the study on the current dose will be terminated, and the SC will decide whether to reduce the dose for exploration.<br><br>If progressive disease occurs (the first PD, confirmed by the IRRC as per RECIST v1.1) in a subject, the investigator will determine whether to **continue the treatment**: after the occurrence of the first PD, **continue the treatment as per the original regimen** for a maximum of 6 weeks and then perform tumor assessment again; if tumor progression occurs again (the second PD), end the study treatment for the subject and proceed to the follow-up period; if no progression occurs, continue the treatment as per the **original regimen**.<br><br>***The second stage is a three-arm, randomized, double-blind, multicenter, phase III clinical study.***<br><br>Eligible subjects in this study will be randomized in a 1:1:1 ratio to 3 groups as follows:<br>- **Group A (HLX10 + HLX04)**: HLX10 + HLX04 combined with chemotherapy (carboplatin-pemetrexed)<br>- **Group B (HLX10)**: HLX10 + HLX04 placebo combined with chemotherapy (carboplatin-pemetrexed)<br>- **Group C (control group)**: HLX04 placebo + HLX04 placebo combined with chemotherapy (carboplatin-pemetrexed)<br><br>Randomization is stratified by: PD-L1 expression level (negative, positive, or not evaluable), smoking history (yes versus no), and brain metastasis (yes versus no).<br><br>After screening, subjects meet the inclusion criteria and not meeting the exclusion criteria will be enrolled. Each included subject will be treated with study drugs once every 3 weeks until loss of clinical benefit, death, intolerable toxicity, withdrawal of informed consent, or occurrence of other reasons specified in the protocol (whichever occurs first).<br><br>During the study, if a subject experiences progressive disease (the first PD, confirmed by the IRRC as per RECIST v1.1), the investigator will unblind the subject and decide whether to **continue the treatment, as detailed below**:<br>➢ Group A: After the occurrence of the first PD, **continue the treatment as per the original regimen** for a maximum of 6 weeks and then perform tumor assessment again; if tumor progression occurs again (the second PD), end the study treatment for the subject and proceed to the follow-up period; if no progression occurs, continue the treatment as per the **original regimen**.<br>➢ Group B: After the occurrence of the first PD, **continue the treatment as per the original regimen** for a maximum of 6 weeks and then perform tumor assessment again; if tumor progression occurs again (the second PD), end the study treatment for the subject and proceed to the follow-up period; if no progression occurs, continue the treatment as per the **original regimen**.

	➢ Group C: After the occurrence of the first PD, discontinue chemotherapy, and continue the treatment **with HLX10 + HLX04**. If tumor progression occurs again (the second PD) after treatment, the investigator will determine whether to **continue the treatment with HLX10 + HLX04**. If the treatment is continued, perform tumor assessment again after a maximum of 6 weeks; if tumor progression occurs the third time (the third PD), end the study treatment for the subject and proceed to the follow-up period.<br>If a subject **decides to continue the treatment** after PD, **then the subject must meet the following criteria (applicable to the first stage and the second stage):**<br>(1) Absence of clinical symptoms and signs of significant disease progression (including worsening laboratory results).<br>(2) A stable ECOG PS score.<br>(3) With no rapid disease progression or tumor progression requiring urgent alternative medical intervention at critical anatomical sites (e.g., spinal cord compression).<br>(4) The major organ function meets the inclusion and exclusion criteria of this study.<br>**(5) The subject should sign an informed consent form.**<br>The primary objective of this study is PFS assessed by IRRC as per RECIST v1.1, which will be compared between group B and group C. For subjects receiving the first evaluation of PD, regardless of the continuation of study treatment after progression, the first PD date as assessed by the IRRC will be used for all statistical analyses containing progression information.
**Planned number of subjects**	Approximately 6–12 subjects in the first stage and 630 subjects in the second stage (210 subjects in each group)
**Study drugs, dosage, regimen, and route of administration**	These study drugs are administered as follows in every 3-week (21-day) treatment cycle.<br>**<u>Investigational product:</u>**<br>➢ HLX10 or placebo, 4.5 mg/kg, intravenous infusion (IV), to be completed within 30–90 min. Administering on day 1 of each 3-week (21-day) cycle. The treatment will continue until loss of clinical benefit, or until the duration of treatment reaches 2 years (up to 35 dosing cycles), and then the investigator will decide whether to continue the treatment.<br>➢ HLX04 or placebo, 15 mg/kg, intravenous infusion, the first infusion should last 90 min (± 15 min); if the subject can well tolerate the first infusion, the duration of second infusion can be shortened to 60 min (± 15 min); if the subject can well tolerate the infusion in 60 min (± 15 min), the subsequent infusions can be completed within 30 min (± 10 min). Administration on day 1 of each 3-week (21-day) cycle. The treatment will be continued until loss of clinical benefit, or until the duration of treatment reaches 2 years (up to 35 dosing cycles), and then the investigator will decide whether to continue the treatment.<br>**<u>Other study drugs: combination chemotherapy</u>**<br>➢ Pemetrexed: 500 mg/m^2, intravenous infusion, the duration of infusion should exceed 10 min. Administering on day 1 of each 3-week (21-day) cycle. The treatment will be continued until loss of clinical benefit, or until the duration of treatment reaches 2 years (up to 35 dosing cycles), and then the investigator will decide whether to continue the treatment.

	➢ Carboplatin: AUC = 5, up to a dosage of 800 mg by intravenous infusion on Day 1 of each 3-week (21-day) cycle, for up to 4 cycles.<br>Refer to **Figure 3. Schematic of study treatment** for the regimen of each treatment group.<br>On the day of administration in each treatment cycle, subjects are given HLX10 or placebo intravenously first, followed by intravenous infusion of HLX04 or placebo, at an interval of at least 30 min, and intravenous infusions of pemetrexed + carboplatin at last. Vital signs should be closely monitored during the administration. At the second stage, HLX10 or placebo and HLX04 or placebo will be administered via a blinded infusion, and pemetrexed + carboplatin via an open-label infusion. Treatment with the study drugs will continue until disease progression, intolerable toxicity, discontinuation decided by subject or investigator, death, withdrawal of consent, pregnancy, incompliance with protocol or procedure requirements, administrative reasons, or other reasons specified in the protocol, whichever occurs first. If carboplatin is not used due to toxicity or other reasons in a certain cycle, it is not counted as the number of combined chemotherapy cycles. After completing 4 cycles of carboplatin, even if the subject does not meet the above criteria, carboplatin will not be continued.
**Prophylaxis**	Prophylactic and other supportive treatment for nausea and vomiting will be given to subjects according to local medical practice before and after carboplatin and pemetrexed administration.
**Dose modification**	Starting from the beginning of HLX10 or placebo/HLX04 or placebo infusion, subjects shall be closely monitored for anaphylaxis that may occur within a few minutes. Infusion should be stopped immediately, and proper treatment should be performed in the event of severe hypotension, bronchospasm, or generalized rash/erythema. In case of mild symptoms (such as flushing or local skin reactions), drugs may be administered at a slower speed. For a life-threatening reaction, including anaphylaxis, hypersensitivity reactions, renal failure, severe cardiopulmonary events, and severe skin reactions, the medications shall be discontinued permanently.<br>The dosing window is ± 3 days from the scheduled date of administration (from the date of the first dose). Drugs administered outside the dosing window is considered a delayed dose, and subsequent doses shall be administered according to the actual date of last administration. During combined treatment, if a delay of more than 2 weeks is expected due to the toxicity of chemotherapy, only HLX10 or placebo/HLX04 or placebo will be administered until the toxicity returns to the standard of chemotherapy administration. Chemotherapy may be continuously suspended for a maximum of 6 weeks, otherwise the chemotherapy should be discontinued. If a delay of more than 2 weeks is expected due to the toxicity of HLX10 or placebo/HLX04 or placebo, only chemotherapy will be administered until the toxicity recovers to the HLX10 or placebo/HLX04 or placebo dosing criteria. HLX10 or placebo/HLX04 or placebo therapy may be continuously suspended for a maximum of 12 weeks, otherwise the HLX10 or placebo/HLX04 or placebo will be discontinued. In case of a delay due to toxicity with equivocal association, all the study drugs shall be synchronously delayed if the event is expected to return to re-dosing standards within 2 weeks.

<u>Principles for HLX10 or placebo dose modification</u>

In the event of HLX10 or placebo-related toxicity, a delay in HLX10 or placebo is allowed **rather than dose modification**. Subjects who miss a scheduled infusion should be actively contacted to arrange another visit with the least delay for administration. Administration of HLX10 or placebo may be delayed, but the subsequent dosing interval should be no more than 12 weeks. A dosing interval of more than 12 weeks is considered intolerable, where HLX10 or placebo will be permanently discontinued, and the subject should withdraw from the trial. For a treatment delay due to intolerance to HLX10 or placebo, other study drugs should be administered as scheduled.

<u>Principles for HLX04 or placebo dose modification</u>

Dose modification of HLX04 or placebo due to adverse events is allowed based on the following basic principles:

Dose level	HLX04 or placebo dosing regimen
Initial dosage	15 mg/kg by intravenous infusion on Day 1 of each 3-week (21-day) cycle.
First dose reduction	7.5 mg/kg by intravenous infusion on Day 1 of each 3-week (21-day) cycle.

If the adverse event is relieved to grade 1 or baseline after the first dose reduction and there is no other toxicity after 6 weeks of treatment at the reduction level, it is allowed to resume the treatment at the initial dosage again.

Dosage of HLX04 or placebo below 7.5 mg/kg will not be allowed. Administration of HLX10 or placebo may be delayed. Two consecutive doses of HLX04 or placebo will be administered up to 12 weeks apart, but a dosing interval of more than 12 weeks is considered intolerable, where HLX04 or placebo will be permanently discontinued. In the event of treatment delay due to intolerance to HLX10 or placebo, other study drugs will be administered as scheduled.

If the adverse event is relieved to grade 1 or baseline after the first dose reduction and there is no other toxicity after 6 weeks of treatment at the reduction level, it is allowed to resume the treatment at the initial dosage again.

Dosage of HLX04 or placebo below 7.5 mg/kg will not be allowed. Administration of HLX10 or placebo may be delayed. Two consecutive doses of HLX04 or placebo will be administered up to 12 weeks apart, but a dosing interval of more than 12 weeks is considered intolerable, where HLX04 or placebo will be permanently discontinued. In the event of treatment delay due to intolerance to HLX10 or placebo, other study drugs will be administered as scheduled.

<u>Principles for chemotherapy dose modification</u>

In the event of intolerance to carboplatin/pemetrexed, dosages may be adjusted twice in accordance with the prescribing information of carboplatin and pemetrexed and local treatment standards.

Dose level	Pemetrexed dosing regimen	Carboplatin dosing regimen
Initial dosage	500 mg/m2 by intravenous infusion on Day 1 of each 3- week (21-day) cycle	AUC = 5, up to a dosage of 800 mg by intravenous infusion on Day 1 of each 3-week (21-day) cycle
First dose reduction	75% of starting dosage	75% of starting dosage
Second dose reduction	50% of starting dosage	50% of starting dosage

	In the event of treatment delay due to intolerance to HLX10 or placebo/HLX04 or placebo, chemotherapy will be conducted as scheduled. During the combined treatment, if a delay is expected due to the toxicity of chemotherapy, chemotherapy may be continuously suspended for a maximum of 6 weeks, otherwise, the chemotherapy should be discontinued. If the chemotherapy medications are discontinued for any reason other than disease progression and the subject has received at least 3 cycles of combination therapy, subjects with response or stable disease may continue to receive HLX10 or placebo/HLX04 or placebo therapy.
**Endpoints**	**Stage I:**<br>**Primary endpoint:**<br>- Safety and tolerability of the first cycle of study treatment;<br>**Secondary endpoints:**<br>- Incidence rates of adverse events (AEs) and serious adverse events (SAEs);<br>- Overall survival (OS);<br>- Progression-free survival (PFS, assessed by the IRRC and the investigator as per RECIST v1.1);<br>- Objective response rate (ORR, assessed by IRRC and the investigator as per RECIST v1.1);<br>- Duration of response (DOR, assessed by IRRC and the investigator as per RECIST v1.1);<br>- Pharmacokinetics (PK): serum HLX10/HLX04 concentration;<br>- Immunogenicity assessment: positive rate of anti-drug antibody (ADA);<br>- Relationship between PD-L1 expression level, MSI, TMB in tumor tissues and efficacy;<br>- Quality of life assessment.<br>**Stage II**<br>**Primary endpoint:**<br>- Progression-free survival (PFS, assessed by IRRC as per RECIST v1.1);<br>**Secondary endpoints:**<br>- Overall survival (OS), as a key secondary endpoint in this study;<br>- Progression-free survival (PFS, assessed by the investigator as per RECIST v1.1);<br>- Objective response rate (ORR, assessed by IRRC and the investigator as per RECIST v1.1);<br>- Duration of response (DOR, assessed by IRRC and the investigator as per RECIST v1.1);<br>- Incidence rates of adverse events (AEs) and serious adverse events (SAEs);<br>- Pharmacokinetics (PK): serum HLX10/HLX04 concentration;<br>- Immunogenicity assessment: positive rate of anti-drug antibody (ADA);<br>- Relationship between PD-L1 expression level, MSI, TMB in tumor tissues and efficacy;<br>- Quality of life assessment.

Inclusion/Exclusion Criteria

Inclusion criteria:

1. Voluntary participation in clinical studies; fully understand, be informed about the study and have signed the informed consent form (ICF); willingness to follow and ability to complete all trial procedures;
2. Aged ≥ 18 years and ≤ 75 years at the time of signing the ICF;
3. Histologically or cytologically diagnosed with stage IIIB, IIIC, or IV (AJCC 8th edition) non-squamous non-small cell lung cancer that cannot be treated with surgery or radiotherapy;
4. With no EGFR sensitive mutation or ALK, ROS1 gene rearrangement;

   Note: Blood test results alone are not accepted.
5. Having not previously received systemic therapy for stage IIIB, IIIC, or IV NSCLC. Patients who have received adjuvant or neoadjuvant treatment are allowed to be enrolled if the adjuvant/neoadjuvant treatment has been completed at least 6 months before the diagnosis of stage IIIB, IIIC, or IV NSCLC;
6. With at least one measurable lesion as assessed by IRRC as per RECIST v1.1 within 4 weeks prior to randomization;

   Note: Measurable lesions should not be from previously irradiated sites.
7. The subjects must provide tumor tissue to measure the PD-L1 expression level;
8. An ECOG PS score of 0 or 1 within 7 days prior to the first dose of the study drugs;
9. An expected survival of ≥ 12 weeks;
10. Normal major organ functions as defined by the following criteria (no blood transfusions, or treatment with albumin, recombinant human thrombopoietin or colony-stimulating factor (CSF) within 14 days prior to the first dose in this study):

**Hematologic System**	
Absolute neutrophil count (ANC)	$\geq 1.5 \times 10^9$/L
Platelet (PLT)	$\geq 100 \times 10^9$/L
Hemoglobin (Hb)	≥ 90 g/L
**Liver function**	
Total bilirubin (TBIL)	≤ 1.5 × upper limit of normal (ULN)
Alanine transaminase (ALT)	≤ 2.5 × ULN;<br>≤ 5.0 × ULN for patients with liver metastases
Aspartate transaminase (AST)	≤ 2.5 × ULN;<br>≤ 5.0 × ULN for patients with liver metastases
**Renal function**	
Creatinine (Cr)	≤ 1.5 × ULN;<br>In case of > 1.5 × ULN, creatinine clearance ≥ 50 mL/min<br>(calculated using Cockcroft-Gault formula)

**Coagulation function**	
Activated partial thromboplastin time (APTT)	≤ 1.5 × ULN
International normalised ratio (INR)	≤ 1.5 × ULN
**Urinalysis/24-h urine protein**	
Urine protein	Urine protein ≤ 1+;<br>In the case of ≥ 2+, a 24-hour urine protein test is required, and subjects with 24-hour urine protein of < 1 g are allowed to be enrolled

11. Female patients must meet one of the following conditions:

(1) Menopause (defined as no menses for at least 1 year with no confirmed cause other than menopause), or

(2) Surgically sterilized (removal of the ovaries and/or uterus), or

(3) With child-bearing potential, but must:

- be tested negative for serum pregnancy test within 7 days prior to the first dose, and
- agree to use contraception with an annual failure rate of < 1% or to remain abstinent (avoid heterosexual intercourse) from signing the informed consent form to at least 120 days after the last dose of the investigational drug and at least 150 days after the last dose of chemotherapy medication (contraception methods with an annual failure rate of < 1% include bilateral tubal ligation, male sterilization, correct use of hormonal contraceptives that inhibit ovulation, hormone-releasing intrauterine devices, and copper-containing intrauterine devices.) , and
- not breastfeed;
- Male patients must: agree to remain abstinent (avoid heterosexual intercourse) or take contraception measures as follows: male patients with a pregnant partner or a partner with childbearing potential must remain abstinent or use a condom for at least 150 days after treatment to prevent the drug exposure to the embryo. The reliability of sexual abstinence should be evaluated based on clinical trial duration and patient's preference and lifestyle. Periodic abstinence (e.g., contraception based on calendar day, ovulatory phase, basal body temperature, or postovulatory phase) and external ejaculation are ineligible methods of contraception.

Exclusion criteria:

1. Subjects with non-small cell lung cancer of other histopathological types, including subjects with mixed adenosquamous carcinoma and NSCLC subjects with small cell lung cancer and neuroendocrine carcinoma;
2. Subjects with other active malignancies within 5 years or at the same time. Localized tumors that have been cured such as basal cell carcinoma, squamous-cell skin cancer, superficial bladder carcinoma, prostate carcinoma in situ, cervical cancer in situ, and breast cancer in situ are acceptable;
3. Subjects who are preparing for or have received an organ or bone marrow transplant;

	4. Patients with pleural or pericardial effusions or ascites requiring clinical intervention;<br>5. Patients with known or documented active central nervous system (CNS) metastases and/or carcinomatous meningitis at screening. However, the following subjects are allowed to be enrolled: 1) Subjects with asymptomatic brain metastases (i.e., no progressive central nervous system symptoms caused by brain metastases, no requirement for corticosteroids, and lesion size ≤ 1.5 cm) may be included, but are required to receive regular brain imaging as a site of lesion. 2) Subjects with treated brain metastases which have been stable for at least 1 month, with no evidence of new or enlarging brain metastases, and with steroids discontinued 3 days prior to administration of the study drugs. Stable brain metastases here should be confirmed before the first dose of the study drugs;<br>6. Subjects with spinal cord compression that have not been radically treated with surgery and/or radiotherapy;<br>7. Patients with myocardial infarction and poorly controlled arrhythmia (including QTc intervals ≥ 450 ms for males and ≥ 470 ms for females) (QTc intervals are calculated by Fridericia's formula) within half a year prior to the first dose of the study drugs;<br>8. Class III to IV cardiac insufficiency according to NYHA classification or an LVEF (left ventricular ejection fraction) < 50% by cardiac color Doppler;<br>9. Subject with peripheral neuropathy ≥ Grade 2 by CTCAE;<br>10. With human immunodeficiency virus (HIV) infection;<br>11. With active pulmonary tuberculosis;<br>12. Subjects with previous and current interstitial pneumonia, pneumoconiosis, radiation pneumonitis, drug-related pneumonitis, and severe impaired pulmonary function that may interfere with the detection and management of suspected drug-related pulmonary toxicity as judged by the investigator;<br>13. With Hepatitis B (positive test for HBsAg or HBcAb and positive test for HBV-DNA) or Hepatitis C (positive tests for HCV antibody and HCV-RNA). With Hepatitis B and C co-infection (positive test for HBsAg or HBcAb and positive test for HCV antibody);<br>14. Patients with known active or suspected autoimmune diseases. Subjects in stable state and requiring no systemic treatment with immunosuppressive agents are included;<br>15. Have received treatment with live vaccines within 28 days prior to the first administration of the study drugs; but inactivated viral vaccines for seasonal influenza are allowed;<br>16. Subjects requiring treatment with systemic corticosteroids (> 10 mg/day prednisone efficacy dosage) or other immunosuppressive drugs within 14 days prior to the first administration of the study drugs or during the study. However, subjects are allowed to be enrolled under the following conditions: in the absence of active autoimmune disease, subjects are allowed to use topical or inhaled steroids and adrenal hormone replacement therapy at dosages equivalent to ≤ 10 mg/day of prednisone efficacy;<br>17. With any active infection requiring systemic anti-infective therapy within 14 days prior to the first administration of the study drugs;<br>18. Have received major surgery within 28 days prior to the first dose of study drugs,

	major surgery in this study is defined as: any surgery which requires at least 3 weeks of postoperative recovery time before receiving the study treatment. Patients with a history of tumor needle biopsy or lymph node incisional biopsy are included;<br>19. Having received radical radiation therapy within 3 months prior to the first dose of study drugs;<br>**Note: Palliative radiotherapy to bone or palliative radiotherapy to superficial lesions is allowed according to local standards 2 weeks prior to the first dose. Radiotherapy covering more than 30% of the bone marrow area within 28 days prior to the first dose is not allowed.**<br>20. Subjects may receive other anti-tumor therapies during the study, such as chemotherapy, targeted therapy, or radiotherapy (except palliative radiotherapy);<br>21. The subject has previously received other antibodies/drugs against immune checkpoints, such as PD-1, PD-L1, CTLA-4, etc.;<br>22. Patients who have received any treatment with bevacizumab or its biosimilars;<br>23. Be in any other ongoing clinical study, or the end of the previous clinical study treatment is less than 14 days from the planned start of this study;<br>24. With a known history of severe allergy to any monoclonal antibody;<br>25. With known hypersensitivity to any of the carboplatin or pemetrexed components;<br>26. Pregnant or lactating women;<br>27. Inadequately controlled hypertension (systolic blood pressure (BP) ≥ 150 mmHg and/or diastolic blood pressure ≥ 100 mmHg);<br>28. With a history of hypertensive crisis or hypertensive encephalopathy;<br>29. With any significant vascular disease (e.g., aortic aneurysm requiring surgical repair or with recent peripheral arterial thrombosis) within 6 months prior to the first dose of study drugs;<br>30. With manifestations of hemorrhage (including hemoptysis, abnormal vaginal bleeding, etc.), or Grade 2 hemorrhagic events within 3 months or Grade 3 or greater hemorrhagic events within 6 months prior to signing the informed consent form;<br>31. Currently use or recently have used (within 7 days prior to the first dose of study drugs) aspirin (> 325 mg/day) or dipyridamole, ticlopidine, clopidogrel, and cilostazol;<br>32. Currently use or recently have used (within 7 days prior to the first dose of study drugs) full-dose oral or injectable anticoagulant or thrombolytic agents for therapeutic purposes; prophylactic anticoagulation therapy for an open intravenous infusion system is allowed so long as the drug activity results in INR and APTT ≤ 1.5 × ULN within 14 days prior to the start of the study treatment. Prophylactic use of low molecular weight heparin (i.e., enoxaparin, 40 mg/day) is allowed;<br>33. Subjects who require long-term treatment with daily administration of nonsteroidal anti-inflammatory drugs (NSAIDs); occasional use of NSAIDs to relieve symptoms associated with medical conditions, such as headache or pyrexia, is allowed;<br>34. Subjects with the following gastrointestinal diseases:<br>• Gastrointestinal perforation, abdominal fistula, or intra-abdominal abscess within 6 months prior to the signing of the informed consent form;<br>• History of poorly controlled or recurrent inflammatory bowel disease

	(including ulcerative colitis and Crohn's disease);<br>• Presence of active peptic ulcer or esophageal varices (moderate or severe);<br>35. Subjects with known history of psychotropics abuse or drug abuse or alcoholism; patients who have stopped drinking can be enrolled;<br>36. In the judgement of the investigator, subjects who have any other factors that may lead to a premature discontinuation.
**Statistics**	**Sample Size Calculation**<br>Approximately 6–12 subjects will be enrolled in the stage I of this study.<br>At stage II, PFS is the primary endpoint, and OS as the key secondary endpoint. The subjects will be randomized in a 1:1:1 ratio, and the sample size is based on the number of events required to demonstrate efficacy with regard to both PFS and OS.<br>Assuming that the median PFS in the Group C is 6 months, and the hazard ratio (HR) of 0.69 in Group B, a total enrollment period of 24 months, the overall study period of 30 months, and the type I error rate $\alpha = 0.05$ (two-sided), at least 264 PFS events must be observed to obtain 85% power. Given a drop-out rate of 15%, a total of 400 subjects should be enrolled in the 2 groups (200 in each group).<br>A fixed-sequence testing method will be used to address the multiplicity of multiple group comparisons. If Group B is statistically different from Group C and the HR is < 1, the comparison between the Group A and the Group B will be continued; otherwise, the comparison will not be performed. Assuming that the median PFS in Group B is 8.7 months, the HR of 0.67 in Group A, and other parameters are the same as above, a total of 404 subjects should be enrolled in the 2 groups (202 in each group). In summary, about 606 subjects should be enrolled and at least 396 PFS events should be observed in Stage II.<br>For the key secondary endpoint OS, assuming that the median OS in the Group C is 10.7 months, and the HR of 0.7 in Group B is, the Lan-DeMets approximation to the O'Brien-Fleming boundary will be used to control the overall type I error rate $\alpha = 0.05$ (two-sided). Assuming that a total enrollment period of 24 months and the overall study period of 46 months, at least 288 OS events must be observed to obtain 85% power.<br>Assuming that the median OS in Group B is 15.2 months, the HR of 0.68 in Group A, and other parameters and calculation methods are the same as above, and taking into account the potential dropout and that the number of events required and the number of subjects enrolled in Group A are the same as those in other treatment groups, about 630 subjects need to be enrolled and at least 432 OS events need to be observed in Stage II.<br>Considering the sample size required for PFS and OS evaluation, a total of 630 subjects (210 in each group) will be enrolled at stage II.<br>**Statistical Analysis Methods**<br>**Analysis populations**<br>- Intent-to-treat (ITT) set: defined as all subjects randomized into the study. ITT population will be considered as the primary analysis population for efficacy analysis in this study. The analysis of ITT population will be conducted based on randomized treatment groups.<br>- Per Protocol Set (PPS): defined as a subset of ITT, including all randomized subjects who have received at least one post-treatment tumor assessment and without major protocol deviations that can significantly affect the primary efficacy. The analysis

based on the PPS will serve as a support of ITT analyses.

- Safety set (SS): defined as all subjects who have received at least one dose of investigational product. The safety population will be the primary analysis population for safety assessment, and will be analyzed based on actual treatment groups.
- Pharmacokinetic Set (PKS): defined as all subjects who have received at least one dose of HLX10 and have at least one post-dose concentration measurement at scheduled PK time points, without any major protocol deviations that can obviously affect the PK assessment. PK set will be used for PK analysis.

Efficacy analysis

Stage I: safety run-in phase

Efficacy endpoints for each subject will be listed.

Stage II: phase III study

- Primary efficacy endpoint

Progression-free survival (PFS) (assessed by IRRC according to RECIST v1.1 criteria): PFS will be compared between Group B and Group C using the stratified log-rank test. Stratification factors include PD-L1 expression level (negative, positive, or not evaluable), smoking history (yes versus no), and brain metastasis (yes versus no).

The HR and its 95% confidence interval will be estimated using the stratified COX proportional hazards model. The median and its 95% confidence interval will be estimated using the Kaplan-Meier method, and the Kaplan-Meier curve will be plotted.

- Secondary efficacy endpoints
- Overall survival (OS): as the key secondary endpoint in this study, using the same statistical method as the primary efficacy endpoint. In the interim analysis of OS, the significance level α will be adjusted according to the actual number of OS events reached at the analysis time point, and the final analysis will be carried out after reaching the target number of OS events;
- Progression-free survival (PFS) (assessed by the investigator as per RECIST v1.1): It uses the same statistical method as the primary efficacy endpoint;
- Objective response rate (ORR) (assessed by IRRC and the investigator respectively as per RECIST v1.1): The stratified Cochran-Mantel-Haenszel (CMH) test is used for the difference in ORR between the two groups, and the odds ratio and its 95% CI are estimated. Stratification factors include PD-L1 expression level (negative, positive, or not evaluable), smoking history (yes versus no), and brain metastasis (yes versus no);
- Duration of response (DOR) (assessed by IRRC and the investigator respectively as per RECIST v1.1): The median is estimated using the Kaplan Meier method and Kaplan-Meier curves are plotted;
- Treatment comparisons will be conducted sequentially by first comparing Group B versus Group C and then comparing Group A versus Group B. The efficacy endpoints PFS, OS, ORR, and DOR between the two groups (Group A versus Group B) will be analyzed in the same manner as the comparison between Group B and Group C.

Interim Analysis

	An Independent Data Monitoring Committee (IDMC) will be established in this study for interim analysis. At stage II, PFS is the primary endpoint, and OS as the key secondary endpoint. PFS will be analyzed twice, including a blinded sample size re-estimation and a final analysis; OS will be analyzed for four times, including a blinded sample size re-estimation, the first interim efficacy analysis during the final analysis of PFS when reaching approximately 33% of the expected number of OS events, the second interim analysis when reaching approximately 66% of the expected number of OS events , and the final analysis when reaching the expected number of OS events. The overall type I error rate will be controlled by the Lan-DeMets approximation to the O'Brien-Fleming boundary.<br>- The first interim analysis will be performed when about 420 subjects have been enrolled (approximately 2/3 of planned number of enrolled subjects), and its primary objective is to perform a blinded sample size re-estimation according to the actual PFS and OS data. This interim analysis will be performed in a blind state, without consuming the significance level α.<br>- Final analysis of PFS will be conducted when the target number (about 396) PFS events are observed, the significance level for the final analysis of PFS is 0.05 (two-sided).<br>- The first interim analysis of OS is performed with the final analysis of PFS, when the number of OS events is approximately 144 (33% of the total number of events). Based on the O'Brien-Fleming type α-spending function, the significance level for the analysis is 0.0002 (two-sided).<br>- The second interim analysis of OS is planned to include approximately 288 OS events (66% of the total number of events). Based on the O'Brien-Fleming type α-spending function, the significance level for the analysis is 0.012 (two-sided).<br>- The final analysis of OS is planned to be performed when 432 OS events are collected. Based on the O'Brien-Fleming type α-spending function, the significance level for the final analysis is 0.046 (two-sided).<br>- In the interim analysis of OS, the significance level α will be adjusted according to the actual number of OS events reached at the analysis time point using O'Brien-Fleming type α- spending function, the overall two-sided type I error rate will be maintained at 0.05, and the final analysis will be carried out after reaching the target number of OS events.<br>A fixed-sequence test (Arms B and C first, followed by Arms A and B; PFS first, followed by OS) will be used to address multiplicity. If the result of the interim analysis is positive, the sponsor may prematurely unblind and end the trial based on the IDMC's recommendation.<br>**Safety Analysis**<br>AEs will be coded according to MedDRA and graded per CTCAE v5.0. Treatment emergent adverse events will be summarized per CTCAE grades. TEAEs and concomitant medications in the trial will be summarized separately by treatment groups. The clinical laboratory parameters, ECOG scores, vital signs, physical examination, and 12-lead ECG will be summarized by the treatment group and study visit. Analysis will describe and present the observed values and changes from baseline by visit in the study. After all subjects have completed the first cycle of study treatment in stage I, a preliminary safety and tolerability analysis will be performed, which will be confirmed by SC, and then the stage II of the study will be initiated.

	**Pharmacokinetics and immunogenicity**<br>Serum drug concentrations, pharmacokinetic parameters, and ADA positive rates are descriptively summarized by visit.<br>**Biomarker analysis**<br>During the screening period in this study, tumor tissues of subjects will be collected for assays of PD-L1 expression level, MSI, and TMB; and blood samples of subjects will be collected for assays of MSI and TMB. The primary objective is to assess the relationship of efficacy to PD-L1 expression, MSI, and TMB.<br>**Analysis of subject-reported outcome variables**<br>Subjects' quality of life will be assessed by EQ-5D-5L rating scale, EORTC QLQ-C30, and EORTC QLQ-LC13 rating scale. The observed values of total score, sub-score, and individual score at each visit and the changes from the baseline will be summarized based on the randomized group using the corresponding score summary rule in the scale.

Figure 1 Study design schematic

Stage I: a single-arm study (safety run-in phase)

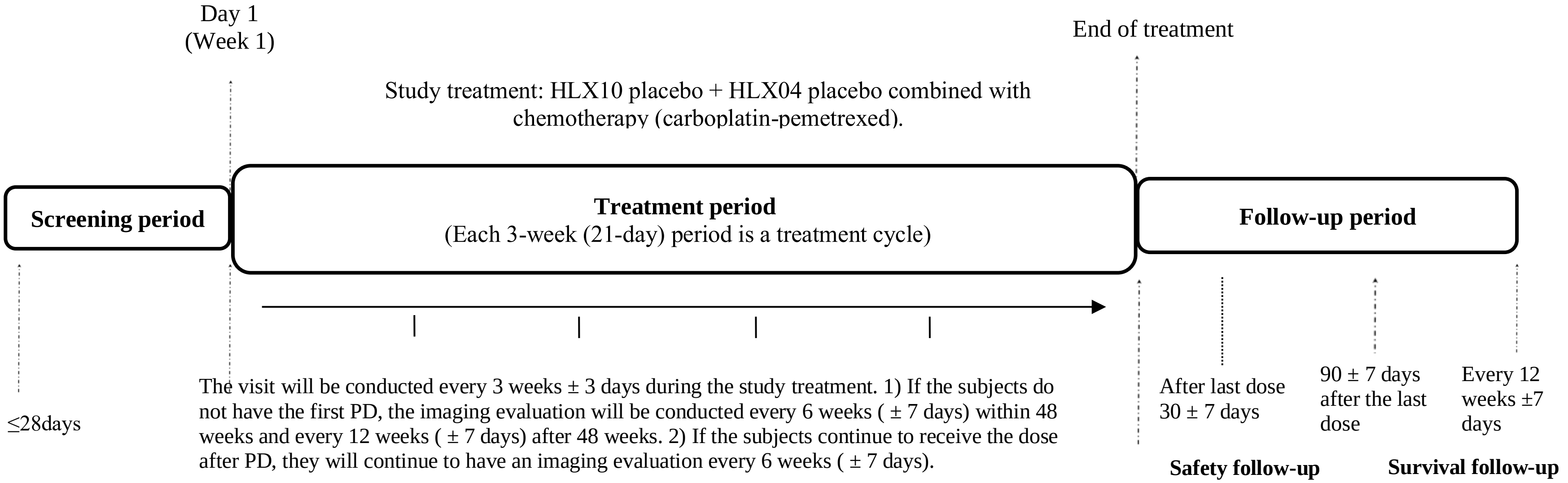

1. After the completion of the first cycle of study treatment for all subjects, a preliminary safety and tolerability analysis will be performed.
2. If a subject experiences the progressive disease (the first PD, PD confirmed by IRRC according to RECIST v1.1 criteria) during the trial, the investigator will determine whether to proceed to continue treatment. Please refer to **Figure 3 "Schematic of study treatment"** for details of study treatment arrangement for subjects.

Stage II: phase III study (three-arm, randomized,

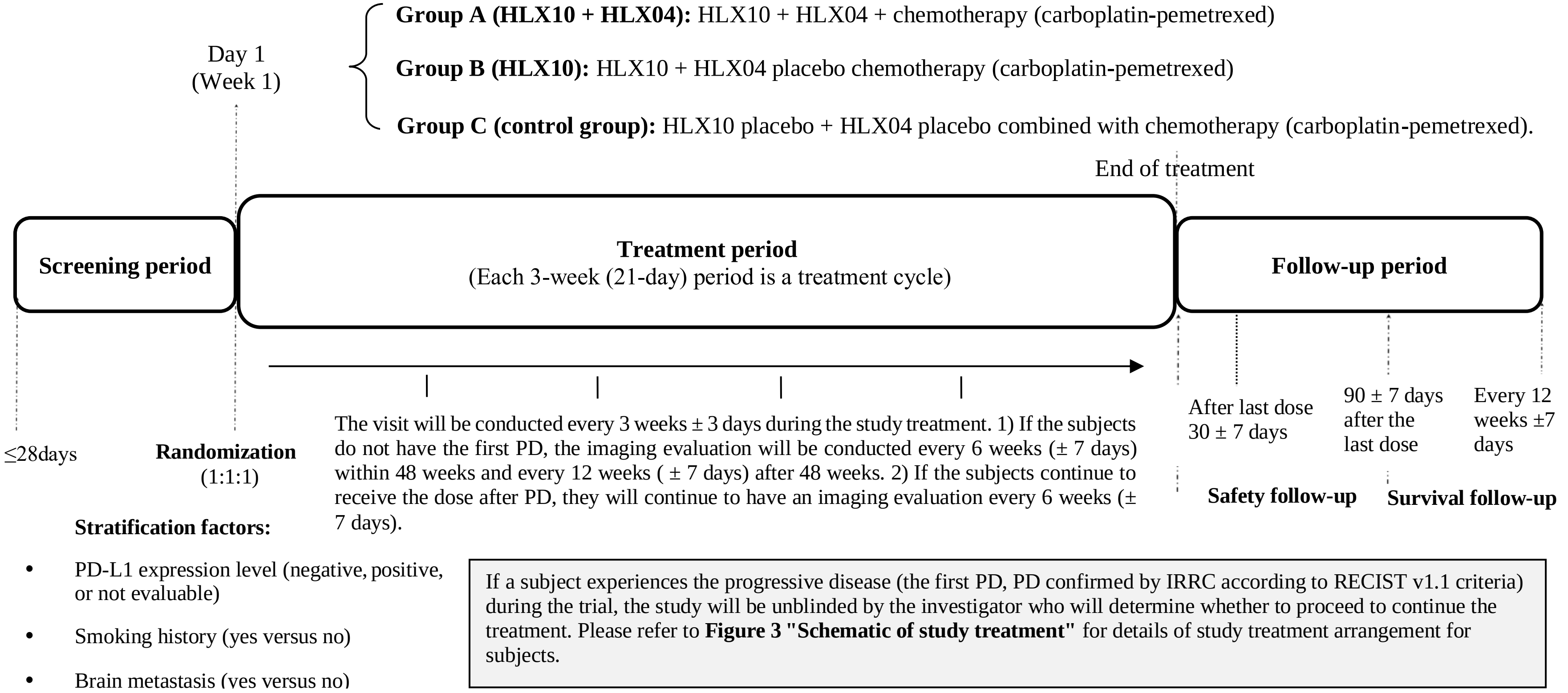

Schedule of Study Procedure

**Period**	**Screening period[1]**		**Treatment period (each 3-week period is a treatment cycle)**					**End of treatment[2]**	**Follow-up period[3]**		
**Treatment cycle/visit**	Screening period		1	2	3	4	n	End of treatment	Safety follow-up		Survival follow-up[4]
**Time of visit**									30 days after the last dose	90 days after the last dose	Every 12 weeks
**Window (day)[5]**	-28 to -8	-7 to -1		± 3	± 3	± 3	± 3	+ 7	± 7	± 7	± 7
**Management procedure**											
Informed consent form	X										
Inclusion/exclusion criteria	X										
Dispensing of subject ID card	X										
Demographics and medical history	X										
Previous and concomitant therapies [6]	X		X	X	X	X	X	X	X	X	
**Clinical operations/assessments**											
AEs[7]	X		X	X	X	X	X	X	X	X	
Quality of life[8]		X	X		X		X	X			
Echocardiography	X										
12-lead ECG	X			X	X	X	X	X	X		
Complete physical examination	X										
Symptom-directed physical examination			X	X	X	X	X	X	X		
Height, weight, and vital signs[9]	X		X	X	X	X	X	X	X		
ECOG scores		X	X	X	X	X	X	X	X		
Subsequent anti-tumor therapy									X	X	X
Survival status			X	X	X	X	X	X	X	X	X
**Study drugs**											
Random			X								
HLX10/placebo, HLX04/placebo [10]			X	X	X	X	X				
Pemetrexed			X	X	X	X	X				
Carboplatin			X	X	X	X					
**Laboratory operations/ assessments: by**											

**Period**	**Screening period**[1]		**Treatment period (each 3-week period is a treatment cycle)**					**End of treatment**[2]	**Follow-up period**[3]		
**Treatment cycle/visit**	Screening period		1	2	3	4	n	End of treatment	Safety follow-up		Survival follow-up[4]
**Time of visit**									30 days after the last dose	90 days after the last dose	Every 12 weeks
**Window (day)**[5]	-28 to -8	-7 to -1		± 3	± 3	± 3	± 3	+ 7	± 7	± 7	± 7
**study site**											
Pregnancy test[11]		**X**			**X**		**X**	**X**	**X**		
Hematology, serum chemistry, coagulation, urinalysis, and myocardial enzymes [12]		**X**		**X**	**X**	**X**	**X**	**X**	**X**		
T3 or FT3, T4 or FT4, and TSH [13]		**X**			**X**		**X**	**X**	**X**		
HBV tests, HBV DNA[14]	**X**										
● In case of HBV DNA (–) and HBsAg (+), and/or 2) HBcAb (+) at baseline. anti- HBV antibody and HBV DNA should be examined during the treatment period.					**X**		**X**	**X**	**X**		
HCV antibody and HCV RNA[14]	**X**										
● In case of 1) HCV antibody (+) and HCV RNA (–) at baseline. anti-HCV antibody and HCV RNA should be examined during the treatment period.					**X**		**X**	**X**	**X**		
HIV	**X**										
**Laboratory operations/ assessments: by central laboratory**											
PK、ADA [15]			**X**	**X**		**X**	**X**	**X**	**X**		
PD-L1 expression level, and MSI and TMB test[17]	**X**										
**Gene mutation status confirmation**											

**Period**	**Screening period[1]**		**Treatment period (each 3-week period is a treatment cycle)**					**End of treatment[2]**	**Follow-up period[3]**		
**Treatment cycle/visit**	Screening period		1	2	3	4	n	End of treatment	Safety follow-up		Survival follow-up[4]
**Time of visit**									30 days after the last dose	90 days after the last dose	Every 12 weeks
**Window (day)[5]**	-28 to -8	-7 to -1		± 3	± 3	± 3	± 3	+ 7	± 7	± 7	± 7
EGFR, ALK, and ROS1 mutation statuses[18]	**X**										
**Response assessment**											
Radiological examination[16]	**X**				**X**		**X**	**X**			

This study is divided into two stages, stage I (safety run-in period) and stage II (phase III study), with the same procedure.

1. The screening period should not exceed 28 days. In this study, **re-screening is allowed for ineligible subjects**: In case of unqualified laboratory tests, a re-test can be performed within the screening time window if determined by the investigator, without the issuance of new screening numbers; and for other conditions incompliant with the inclusion/exclusion criteria, subjects should be re-screened with a new screening number.
2. If a subject discontinues the study treatment for any reason, an end-of-treatment visit should be performed whenever possible and should be completed within 7 days after the discontinuation is learned of or confirmed (and should be completed before the subject starts a new anti-tumor therapy).
3. All subjects are required to visit the study site for safety follow-up 30 days (± 7 days) after the last dose; and if the end-of-treatment visit is delayed for any reason and occurs after the time window of 30 days (± 7 days), no further safety follow-up visit is required. A follow-up telephone call for safety follow-up 90 days (± 7 days) after the last administration is required. Only the information of AEs and AE-related concomitant drugs is collected. For subjects who discontinued for reasons other than PD, radiological assessments are to be continued as scheduled, **until PD**, initiation of new anti-tumor therapy, withdrawal of ICF, death, or end of the study, whichever occurs first.
4. Subjects should be followed up for survival by telephone every 12 weeks ± 7 days after treatment discontinuation; the frequency of survival follow-up may be increased as appropriate.
5. The time window is 28 days for screening, 3 days for treatment (7 days for tumor assessment), and 7 days for follow-up and end-of-treatment visits. For ECOG performance status, pregnancy test, hematology, serum chemistry, coagulation, urinalysis, T3 or FT3, T4 or FT4, and TSH tests during screening period, data **within 7 days prior to the randomization** should be documented, and the subjects should meet the corresponding inclusion/exclusion criteria for enrollment.
6. All prior and concomitant medications are recorded from 30 days prior to the signing of ICF through the safety follow-up visit. Concomitant medications associated with AEs are recorded up to 90 days after the last study treatment.
7. All AEs and treatment emergent AEs are recorded from the time of the ICF signing until 90 days after the last study treatment. If the patient starts a new anti-tumor therapy during AE collection period, only AE information related to the study treatment is collected after the initiation of the new anti-tumor therapy.
8. Quality of life scales including the EQ-5D-5L, the European Organization for Research and Treatment of Cancer Quality of Life Scale (EORTC QLQ- C30), and the European Organization for Research and Treatment of Cancer Lung Cancer Questionnaire Module (EORTC QLQ-LC13). Such scales are evaluated prior to the first dose and every other subsequent dosing cycle (i.e., pre-dose in Cycles 1, 3, 5, 7, etc.) until the end of treatment. A quality-of-life assessment is required at the end-of-treatment visit if no assessment is performed within the past 3 weeks. Re-assessments prior to dosing in Cycle 1 are not required for

**Period**	**Screening period[1]**		**Treatment period (each 3-week period is a treatment cycle)**					**End of treatment[2]**	**Follow-up period[3]**		
**Treatment cycle/visit**	Screening period		1	2	3	4	n	End of treatment	Safety follow-up		Survival follow-up[4]
**Time of visit**									30 days after the last dose	90 days after the last dose	Every 12 weeks
**Window (day)[5]**	-28 to -8	-7 to -1		± 3	± 3	± 3	± 3	+ 7	± 7	± 7	± 7

subjects who have a quality-of-life assessment on Day –7 to Day –1 of the screening period.

9. The height measurement is performed only at screening and vital signs include body temperature, pulse, respiratory rate, and blood pressure. Body weight is measured before each administration.
10. The investigational product is administered based on 3-week cycles after all clinical and laboratory operations/assessments are completed. **No more than 3 days should be elapsed between the date of randomization and the date of the study treatment initiation.**
11. Women of childbearing potential must have a serum pregnancy test. During the treatment period, the test should be done within 3 days pre-dose every 2 cycles. Analysis will be carried out in the local study site.
12. Routine laboratory tests include hematology, serum chemistry, coagulation, myocardial enzymes, and urinalysis. These tests will be carried out within 3 days pre-dose in each cycle; if aforementioned laboratory tests are scheduled on the same day as the study treatment, the study treatment can be arranged only after the test results are obtained. During the carboplatin therapy, routine blood tests should be performed on Day 8 (± 3 days) of each treatment cycle to closely monitor bone marrow suppression.
13. Thyroid function tests include triiodothyronine (T3 or FT3), thyroxine (T4 or FT4), and thyroid-stimulating hormone (TSH) assays. During the treatment period, the test should be done within 3 days pre-dose every 2 cycles. Analysis will be carried out in the local study site.
14. All subjects should be tested for HBV markers (HBsAg, HBsAb, HBeAg, HBeAb, and HBcAb) and HCV antibody during screening; HBsAg or HBcAb positive subjects should be further tested for HBV DNA titer; and anti-HCV antibody positive subjects should be further tested for HCV RNA. In case of HBV DNA (–) and: 1) HBsAg (+), and/or 2) HBcAb (+) during screening (baseline), HBV antibody and HBV DNA should be tested every 2 cycles in the treatment period. In case of 1) HCV antibody (+) and HCV RNA (–) at baseline, HCV antibody and HCV RNA should be tested every 2 cycles in the treatment period. The investigator will receive antiviral treatment as clinically needed.
15. PK and ADA sampling: (**Note: ADA samples will only be collected at pre-dose** and procedures are described in the laboratory manual.)
    - Blood samples will be collected at the following points: within 7 days **pre-dose** in Cycle 1; within 3 days **pre-dose** in Cycles 2, 4, 6, 8, and every4 cycles thereafter; within 2 hours after **the end of** HLX10 or placebo/HLX04 or placebo dosing in Cycles 1 and 8 of treatment period **(for PK only)**; and at end-of-treatment visit and/or safety follow-up.
16. CT or MRI should be performed at screening and after the start of study treatment on sites including brain, chest, abdomen, pelvic cavity and any other sites suspected to have tumor lesions, among which **brain MRI or CT (preferably MRI)** and bone scans are performed during baseline period, and then in treatment period are performed as determined by the investigator according to clinical needs; examination methods at the same site should be consistent as much as possible throughout the study; and if there are no contraindications, contrast agent should be used. During the treatment, for subjects without first PD, radiological assessment should be performed once every 6 weeks (± 7 days) within 48 weeks, and once every 12 weeks (± 7 days) thereafter. For any subject continuing medication after PD, radiological assessment should be repeated every 6 weeks (± 7 days). The investigator and IRRC respectively assess the tumor images according to RECIST v1.1 (the anti-tumor efficacy assessment can be performed by the investigator according to clinical needs), and the investigator

**Period**	**Screening period[1]**		**Treatment period (each 3-week period is a treatment cycle)**					**End of treatment[2]**	**Follow-up period[3]**		
**Treatment cycle/visit**	Screening period		1	2	3	4	n	End of treatment	Safety follow-up		Survival follow-up[4]
**Time of visit**									30 days after the last dose	90 days after the last dose	Every 12 weeks
**Window (day)[5]**	-28 to -8	-7 to -1		± 3	± 3	± 3	± 3	+ 7	± 7	± 7	± 7

should make subsequent treatment judgement according to the results of their own response assessment. If a tumor assessment is performed within 28 days prior to the first dose by the same method and devices in the same hospital, it may serve as the baseline tumor assessment. If the investigator evaluates as PD for the first time, the IRRC needs to evaluate the tumor imaging according to RECIST v1.1. If the IRRC confirms as PD, the investigator will unblind and decide whether to continue the treatment. At the end-of-treatment visit, if tumor imaging is performed within the last 4 weeks, a re-test is not required. **For subjects who discontinued for reasons other than PD, radiological assessments are to be continued as scheduled,** until PD, initiation of new anti-tumor therapy, withdrawal of ICF, death, or end of the study, whichever occurs first.

17. In the screening period, the subjects **must provide** formalin-fixed paraffin-embedded (FFPE) tumor samples (paraffin blocks or unstained sections) (samples within 6 months prior to the first study treatment are recommended) at non-radiotherapy sites collected at or after the diagnosis of advanced NSCLC for PD-L1 expression level determination in the central laboratory, and pathological reports of such specimens. In the absence of recent archival tumor tissue samples, a fresh biopsy of a tumor lesion at screening should be accepted to obtain the corresponding tumor samples for PD-L1 expression level determination (the number of samples depends on biopsy). If the subject agrees, tumor tissue samples and blood samples can be collected for MSI and TMB testing in the central laboratory. If pathological sampling is performed for subjects during the study treatment, it is recommended to collect their tumor samples; and these tumor tissue sections will be used for immunohistochemical analysis and PD-L1 expression level determination, to evaluate the expression level of PD-L1 in tumor cells and tumor- infiltrating immune cells, MSI, relationship between TMB and efficacy, and other purposes. Fresh sample collection, resection, core needle biopsy, and excisional, incisional, incisional, punch, or forceps biopsies are all acceptable. Fine-needle aspirations (i.e., samples that lack a complete tissue structure and provide only cell suspension and/or cell smear), brush biopsies, and cell pellet samples from pleural or peritoneal effusions are unacceptable. **For detailed requirements for tissue samples, see the Laboratory Operation Manual.**

18. The subjects are required to provide test reports on the mutation status of EGFR, ALK and ROS1 genes (using the method specified in the NCCN guidelines, and not accepting blood test results alone). Subjects should be excluded if they are known to have EGFR sensitive mutation or anaplastic lymphoma kinase (ALK) or ROS1 gene rearrangement or fusion mutation. If the status of EGFR, ALK, or ROS1 is unknown, EGFR, ALK, and ROS1 mutation will be tested in the screening period.

1. INTRODUCTION

1.1 Disease Background

Lung cancer is a common clinical malignant tumor. Its incidence and mortality are the highest among all malignant tumors, accounting for 15% with high mortality and 5-year survival rate being less than 15%. According to the data released by the National Cancer Center of China in 2018, there are about 781,000 new cases of and 626,000 deaths from lung cancer each year in China, with its occurrence and mortality ranking first among malignant tumors[1].

Non-small cell lung cancer (NSCLC) is the most common type of primary lung cancer, accounting for approximately 80% of all lung cancers[2]. Due to the insidious onset of NSCLC at the early stage, 70–80% of patients are at advanced stage at the time of clinically definite diagnosis, losing the best time for surgical treatment, and a significant proportion of patients with early-stage NSCLC receiving surgical treatment will develop distant recurrence and die due to lung cancer[3]. Despite improvements in diagnosis, imaging, staging, and treatment of NSCLC, the estimated 5-year overall survival rate of patients with phase IV NSCLC remains low worldwide. The 5-year overall survival rates of patients with phase IV NSCLC in Europe and the United States are 11% and 17%, respectively, and their counterpart in China is less than 20%[4][5].

1.2 Research Development and Basis for Dose Selection

1.2.1 Overview of HLX10 and HLX04

HLX10 is an innovative monoclonal antibody targeting PD-1 independently developed by Shanghai Henlius Biotech, Inc. HLX10 is an IgG4 humanized monoclonal antibody, and its gene sequence is screened using the hybridoma technique and genetically engineered to complete humanization. Chinese hamster ovary cells (CHO) are used as the host cell to construct a stable cell line to produce the protein characterized by a typical human immunoglobulin IgG4 structure of a Y shape, which is formed by two identical heavy chains and two identical light chains linked by an inter-chain disulfide bond. HLX10 has been approved by FDA from US, TFDA from Taiwan, China, and NMPA from China for a dose escalation phase I clinical trial. There are four dose groups (0.3, 1, 3, and 10 mg/kg, once every two weeks) in the trial, with a maximum enrollment of about 30 subjects.

HLX04, a similar product of Avastin® from Roche, is a recombinant anti-VEGF humanized monoclonal antibody developed by Shanghai Henlius Biotech, Inc. Its humanized gene sequence is designed by Henlius according to the protein sequence of Avastin® of Roche and the product is obtained by the transfection of CHO cells with the expression vector, enabling the involvement of human IgG framework region and mouse CDR sequence, and massively produced in CHO cells. As a glycosylated and covalently cross-linked tetramer, HLX04's large-scale

manufacturing is based on the wide-ranging cell culture and purification. HLX04 was approved for clinical trials in the treatment of metastatic colorectal cancer in December 2015 and non-squamous non-small cell lung cancer in May 2016, with approval numbers of 2015L05219 and 2016L04811, respectively. The phase I head-to-head clinical trial (healthy population) between HLX04 and bevacizumab has been completed and the phase III clinical trial (advanced colorectal cancer) is undergoing. The results of the phase I clinical trial showed that HLX04 enjoys adequate safety, and that the PK data of Avastin® marketed in China, the EU, and the US have no significant difference.

1.2.2 Non-clinical studies on HLX10

1.2.2.1 In vitro pharmacodynamics (PD) studies of HLX10

A series of in vitro PD studies comparing HLX10 with the active control Nivolumab showed that HLX10 can bind to the surface of activated T cells expressing PD-1 and has the ability to block the binding of PD-1 to PD-L1 or PD-L2 on the cell surface. The binding and blocking ability of HLX10 present dose-dependence. The HLX10 in vitro mixed leukocyte reaction (MLR) assay showed that HLX10 blocks immunosuppression depending on the binding of PD-1 to its ligands, thereby stimulating activated $CD4^+$ T cells, increasing T cell proliferation, and producing more IL-2 cytokines. This phenomenon was dose-dependent in both the investigational product HLX10 and the active control Nivolumab.

In addition, to investigate the occupancy ratio of HLX10 to PD-1 receptor on human T cells, different doses of HLX10 (50.0, 10.0, 2.0, 4.0×10^{-1}, 8.0×10^{-2}, 1.6×10^{-2}, 3.2×10^{-3}, and 6.4×10^{-4} μg/mL) were pre-incubated with the whole blood of six healthy subjects to simulate the injection of HLX10 into human blood. The results showed that with an increasing dose of HLX10 during pre-incubation, the PD-1 receptor occupancy (RO) on $CD3^+$ T cells increased. Once the blood concentration of HLX10 in four of the six healthy subjects reached 2 μg/mL, the PD-1 RO on $CD3^+$ T cells was more than 80%.

1.2.2.2 In vivo pharmacodynamics studies of HLX10

The anti-tumor efficacy and safety evaluation in NOD/SCID mice model subcutaneously xenografted with HT-29 human colon cancer cells showed that HLX10 had no adverse effects on the health status and body weight of the mice in each dose group, indicating that HLX10 has a high level of safety. In terms of tumor suppression, 30 mg/kg HLX10 effectively inhibited the growth of subcutaneously xenografted HT-29 human colon cancer cells in NOD/SCID mice in the presence of human peripheral blood mononuclear cells ($P < 0.0001$).

Anti-tumor efficacy and safety evaluation in NOD/SCID mice model subcutaneously xenografted with human non-small cell lung cancer cells NCI-H292 showed that the higher dose of HLX10 did not cause adverse effects on health status and body weight of mice, indicating that

HLX10 has a high level of safety. In terms of tumor suppression, HLX10 at a dose of 30 mg/kg significantly inhibited the growth of NCI-H292 tumor tissues in the presence of human peripheral blood mononuclear cells ($P < 0.001$), as compared with the placebo group in both tumor volume observation and statistical data.

In the dose-finding study (P16-106-TS), pharmacokinetics study (P16-106-YD), and chronic toxicity study (P16-106-CD) in cynomolgus monkeys, the receptor occupancy at different time points before and after the intravenous injection of HLX10 at different doses in cynomolgus monkeys was studied concomitantly, which provided a basis for the selection of clinical effective dose and initial dose.

The final results showed that the results of in vivo test were consistent with that of in vitro test in terms of the PD-1 RO in human peripheral blood, i.e., once the HLX10 concentration in the blood of subjects reached 2 μg/mL, the PD-1 RO on $CD3^+$ T cells was more than 80%. In cynomolgus monkeys, when the blood concentrations of both test animals in the 3 mg/kg group were below the lower limit of detection (2 μg/mL), the corresponding RO values were still 79% and 97%, respectively. According to all results, it can be concluded that when a single dose of 3 mg/kg HLX10 was administered to cynomolgus monkeys, the RO saturation can be maintained for more than 4 weeks and when a dose of 5 mg/kg was administered to cynomolgus monkeys for 13 consecutive weeks (once weekly), 100% RO saturation was still achieved in some animals after the 6-week recovery period. It can be inferred that HLX10 at a lower clinical dose (less than 1 mg/kg) can lead to RO saturation and have adequate efficacy.

1.2.2.3 Tissue cross reactivity of HLX10

The human tissue cross reactivity study of HLX10 showed that HLX10-Biotin (2.0 μg/mL and 0.5 μg/mL) specifically bound to normal human lymphocytes, including lymphocytes in lymph node, lung, ileum, stomach, spleen, fallopian tube, colon, and thymus tissues.

The results of cynomolgus monkey tissue cross reactivity study of HLX10 showed that HLX10-Biotin (2.0 μg/mL and 0.5 μg/mL) specifically bound to normal cynomolgus monkey lymphocytes, including lymphocytes in stomach, jejunum, colon, spleen, thymus, and mesenteric lymph nodes.

1.2.2.4 General pharmacology study of HLX10

The general pharmacology assessment test on CNS, cardiovascular system, and respiratory system was conducted in cynomolgus monkeys. This test was conducted concomitantly with the chronic toxicity study. During the test, no investigational product-related abnormalities were found in clinical symptoms of animals in each group, and no obvious abnormalities were found in their behaviors, breathing, etc. The abnormal findings of dying female animal in high-dose group euthanized on D55 were related to amoeba infection, while the abnormal findings of

female animals died on D64 were related to anaphylaxis caused by the drug. Surviving animals showed no regular changes in body temperature, systolic blood pressure, diastolic blood pressure, mean arterial blood pressure, blood oxygen saturation, heart rate, P-R, Q-T, and QTc intervals, QRS duration, and other ECG parameters. Therefore, when 5, 50, and 100 mg/kg of HLX10 were given to cynomolgus monkeys via intravenous infusion for 13 consecutive weeks (once weekly), no significant effect on the CNS, cardiovascular system, and respiratory system of cynomolgus monkeys was observed.

1.2.2.5 Acute toxicity study of HLX10

A 4-week dose-finding toxicity study was conducted in cynomolgus monkeys with repeated intravenous infusions of HLX10 at 5 mg/kg, 50 mg/kg, and 100 mg/kg, respectively. Administration was carried out on D1, D8, D15, and D22, and all animals were euthanized on D29 and subjected to gross anatomy. There were no dead or dying animals during the study. One male animal in medium-dose group had loose and soft stools on D15–20 and D21–29, respectively, and one female animal in medium-dose group also had loose stools on D11–13, D15–20, and D29, which was considered possibly related to the investigational product. No abnormal symptoms related to the investigational product were observed in the low-dose group or the high-dose group. No abnormal changes in body weight, food consumption, body temperature, ECG parameters, coagulation indicators, serum chemistry, or gross anatomy were observed in the animals in each dose group. The proportion of ADA positive animals in 5, 50, and 100 mg/kg groups were 4/4, 3/4, and 4/4, respectively. The time of first appearance of antibodies was D8, and antibody titers ranged from < 1 to 128. Based on the results of this study, HLX10 was well tolerated in cynomolgus monkeys when administered at 100 mg/kg for 4 weeks. The dose of 100 mg/kg can be used for the longer-term repeated-dose study.

1.2.2.6 Chronic toxicity study of HLX10

Toxicity and toxicokinetic studies were performed in cynomolgus monkeys with repeated intravenous infusions of HLX10 for 13 weeks, followed by a recovery period of 6 weeks. Intravenous infusions of placebo and 5 mg/kg, 50 mg/kg, and 100 mg/kg of HLX10 were given once a week for 13 consecutive weeks. During the study, no death or dying related to the investigational product was observed in animals in the low-dose or medium-dose groups. At the dosage of 100 mg/kg, one female animal was found dead from a drug-related anaphylaxis; one dying female animal was euthanized on D55 due to amoebic infection. Loose stools and/or soft stools were observed in each dose group, and the incidence rate in female animals in the high-dose group (100 mg/kg) was slightly higher with slightly longer duration. The above gastrointestinal symptoms were likely related to the drug and were consistent with the adverse reactions reported for anti-PD-1 monoclonal antibodies. Except for two dead animals in the high-dose group, the gastrointestinal reactions (loose/soft stools) of the other animals resolved

completely after the 6-week recovery period. Surviving animals in the treatment groups did not show any other abnormalities related to the investigational product during the study. Except for the female animal in the high-dose group euthanized on D55, ADA positivity was observed in all animals of treatment groups, and ADA response was the highest in the low-dose group. Antibody titers increased with the increasing dose frequency and continued to increase until the end of the recovery period (D134). Given toxicokinetic (TK) results, the presence of ADA significantly reduced systemic exposure in all dose groups. HLX10 at 5 and 50 mg/kg were repeatedly administered to cynomolgus monkeys via intravenous infusion once weekly. No significant toxicity was observed after administration for 13 consecutive weeks and no irritative reaction was seen at the administered sites. No significant effect on major functional systems such as the cardiovascular system, CNS and respiratory system was observed. The non-observed-adverse-effect level (NOAEL) of HLX10 was 50 mg/kg under the conditions of this experiment. The AUC_{last} and C_{max} of males on D85 were 597.60 h·mg/mL and 4909.72 μg/mL, and the AUC_{last} and C_{max} of females were 403.64 h·mg/mL and 3726.17 μg/mL.

1.2.2.7 Preclinical pharmacokinetic study of HLX10

The results of the PK study of single HLX10 intravenous infusion in cynomolgus monkeys showed that the serum drug concentration of HLX10 increased with the increasing dosage in animals that received intravenous infusion of HLX10 at 3, 10, and 30 mg/kg. The systemic exposure (C_{max} and AUC_{last}) increased with the increasing dosage. The median resorting time (MRT) was between 153.02–231.28 h. The elimination half-life ($t_{1/2}$) was between 137.97–256.99 h. The results showed that in the dose range of 3–30 mg/kg, the investigational product showed a basically linear pharmacokinetic profile in cynomolgus monkeys. The CL and volume of distribution (Vz) of HLX10 were similar between groups, ranging from 0.13 to 0.23 mL/h/kg and 38.05 to 53.52 mL/kg, respectively. All animals in 3, 10, and 30 mg/kg dose groups of HLX10 were positive for ADA on D8, D15, D22, and D29. The time of first appearance of antibody was on D8, and the antibody titer ranged from < 1 to 512. Except that the area under the curve (AUC_{last}, AUC_{inf}) of female animals in 10 mg/kg group was lower than that of male animals with statistical difference ($p < 0.05$), there was no significant difference in PK parameters between genders in other dose groups. According to the analysis results of antibody generation, it can be concluded that 2/3 of female animals in 10 mg/kg group developed strong antibody on D22, with antibody titers of 8–128, resulting in slightly faster elimination and lower area under curve (AUC_{last}, AUC_{inf}) in female animals than male animals.

1.2.2.8 Toxicokinetic study of HLX10

The TK study was conducted concomitantly with the 4-week dose-finding toxicity study of repeated HLX10 intravenous infusions in cynomolgus monkeys. The results showed that on D1 and D22, the systemic exposure of HLX10 (C_{max} and AUC_{last}) increased with the increasing

dosage. The generation of anti-drug antibody in some animals on D22 accelerated the elimination of blood HLX10 concentration. In ADA-negative animals, the mean C_{max} and mean AUC_{last} in each dose group on D22 were higher than those on D1, indicating that the drug accumulated to some extent. The blood HLX10 concentration in the D22 pre-dose samples also showed this feature. The accumulation factor was between 2.24–2.41. Compared with those on D1, the ADA negative group showed a smaller Vz and a slower CL of HLX10 on D22. In the ADA-positive group, the CL increased significantly.

A TK study was conducted concomitantly with the toxicity and TK studies in which repeated HLX10 intravenous infusions were given to cynomolgus monkeys for 13 weeks, followed by a 6-week recovery period. The results showed that on D1 and D85, the systemic exposure of HLX10 (Cmax and AUClast) increased with the increasing dosage, showing dose-dependent toxicokinetic characteristics. Some of the animals generated anti-drug antibodies after repeated administration. In ADA-negative animals, the mean Cmax and mean AUClast in each dose group on D85 were higher than those on D1, indicating that the drug accumulated. The blood HLX10 concentration in the D85 pre-dose samples also showed this feature. The accumulation factor was between 2.34–4.32. Compared with those on D1, the ADA negative group showed a smaller Vz and a slower CL of HLX10 on D85. In the ADA-positive group, the CL increased significantly. On D1, the male animals in the 100 mg/kg group had slightly higher systemic exposure (AUC_{last}, AUC_{inf}) and slightly lower CL than the female animals; other groups did not show any significant difference between genders in the PK parameters on D1 or D85.

1.2.2.9 HLX10 immunogenicity and immunotoxicity

Assessment test for immunogenicity and immunotoxicity in cynomolgus monkeys was carried out concomitantly with the chronic toxicity study. The results showed that when 5, 50, and 100 mg/kg of HLX10 were administered intravenously to cynomolgus monkeys once a week for 13 consecutive weeks, no immunotoxicity was observed at the dosages of 5 and 50 mg/kg. At the dosage of 100 mg/kg, 1 female died from anaphylaxis, and this animal was positive for anti-drug antibody and had an increased IL-6 after drug administration. At the dosage of 100 mg/kg, male animals had a decrease in the CD3+, CD4+, and CD4+/CD8+ ratio, and an increase in CD8+ which was associated with the pharmacological activity of the investigational product. The presence of anti-drug antibody was observed after HLX10 was administered to the animals. The incidence rate of anti-drug antibodies in the low-dose group was higher than that in the medium- and high-dose group. Antibody titer increased with the increase of administration time, and could lead to significant reduction of systemic exposure. As a humanized antibody, HLX10 is an exogenous substance to cynomolgus monkeys. Therefore, immunogenicity in the animals is reasonable. For immunogenicity assessment of antibody-based drugs, human clinical trial is a preferred method.

1.2.2.10 Other preclinical studies of HLX10

The hemolysis study of HLX10 showed that HLX10 at a concentration of 10 mg/mL had no hemolytic effect on human red blood cells in vitro and did not cause red blood cell aggregation.

The local irritation study was conducted concomitantly with the chronic toxicity study. The results showed that when HLX10 at dosages of 5, 50, and 100 mg/kg were administered to cynomolgus monkeys by repeated intravenous infusions once weekly for 13 weeks, no obvious irritative injury was observed in blood vessels and surrounding tissues at infusion sites in the dose range of 0.5–10.0 mg/mL.

No genotoxicity study of HLX10 has been conducted yet.

1.2.3 Non-clinical studies of HLX04

1.2.3.1 Pharmacodynamics study of HLX04

HLX04 significantly inhibited the growth of human colorectal adenocarcinoma cells HCT-8 transplanted subcutaneously in nude mice, human colorectal adenocarcinoma LS-174T transplanted subcutaneously in nude mice, and human colonic cancer COLO205 transplanted subcutaneously in nude mice. In the above three models, the effects of HLX04 and bevacizumab injection (Avastin) were similar at the dosage of 5 mg/kg, i.e., HLX04 had the same efficacy as the equivalent dosage of Avastin. The details were as follows:

<u>Inhibitory effect on human colorectal adenocarcinoma cells HCT-8 transplanted subcutaneously in nude mice</u>

A total of 35 nude mice with human colorectal adenocarcinoma cells HCT-8 subcutaneous xenograft models were randomly divided into 5 groups: group 1 (physiological saline), group 2 (Avastin, 5 mg/kg), group 3 (HLX04, 1.25 mg/kg), group 4 (HLX04, 2.5 mg/kg), and group 5 (HLX04, 5 mg/kg), with 7 animals in each group, administered twice a week for 4 consecutive weeks, and euthanized on D29. It was considered effective when T/C (%) ≤ 40% and RTV shows a difference compared with that of the negative control group ($P < 0.05$). The results showed that HLX04 at dosages of 1.25, 2.5, and 5 mg/kg had significant inhibitory effects on tumor growth of human colorectal adenocarcinoma cells HCT-8 transplanted subcutaneously in nude mice, and 1.25 mg/kg was the minimum effective dosage. And HLX04 and Avastin had similar effects at the dosage of 5 mg/kg.

<u>Inhibitory effect on human colorectal adenocarcinoma cells LS-174T transplanted subcutaneously in nude mice</u>

A total of 50 nude mice with human colorectal adenocarcinoma cells LS-174T subcutaneous xenograft models were randomly divided into 5 groups: group 1 (physiological saline), group 2 (Avastin, 5 mg/kg), group 3 (HLX04, 1.25 mg/kg), group 4 (HLX04, 2.5 mg/kg), and group 5 (HLX04, 5 mg/kg), with 10 animals in each group, administered twice a week for 3 consecutive weeks, and euthanized on D22. It was considered effective when T/C (%) ≤ 40% and RTV shows

a difference compared with that of the negative control group ($P < 0.05$). The results showed that HLX04 at the dosage of 5 mg/kg had a significant inhibitory effect on tumor growth of human colorectal adenocarcinoma cells LS-174T transplanted subcutaneously in nude mice, which was similar to the effect of Avastin at the dosage of 5 mg/kg.

Inhibitory effect on human colonic cancer cells COLO205 transplanted subcutaneously in nude mice

This test was to evaluate the anti-tumor effect and safety of HLX04 and the active control Avastin at different dosages in BALB/c nude mouse animal models xenografted with human colonic cancer cell line COLO205. The test includes HLX04 0.5 mg/kg, 5 mg/kg, and 25 mg/kg dose groups, active control Avastin 5 mg/kg group, and placebo group. There were 8 mice in each group, with intraperitoneal administration twice a week, for a total of 3 weeks. The results showed that HLX04 significantly inhibited tumor growth in the human colonic cancer COLO205 tumor model at the dosages of 0.5 mg/kg, 5 mg/kg, and 25 mg/kg, had a good reversal effect on cachexia in the COLO205 colonic cancer model, and was equivalent to the same dosage of Avastin in efficacy. HLX04 was well tolerated in tumor-bearing mice at the dosages tested.

1.2.3.2 Pharmacokinetic study of HLX04

In cynomolgus monkeys, single intravenous infusion of HLX04 at different dosages, multiple intravenous infusions of HLX04 at single dosage, and multiple intravenous infusions of the control Avastin at single dosage were administrated to observe the changes of blood drug concentration-time. The study showed that the pharmacokinetic behavior of HLX04 was very similar to that of Avastin. After intravenous infusion of the investigational product HLX04 in cynomolgus monkeys, a linear pharmacokinetic profile was observed within the range of 2–50 mg/kg. The details were as follows:

Single intravenous infusion of HLX04 at different dosages

After a single intravenous infusion of 2 mg/kg, 10 mg/kg, and 50 mg/kg of HLX04 in cynomolgus monkeys, the serum drug concentration peaked at 0.5 h after administration in most cynomolgus monkeys, and the serum drug concentration increased with the increase of dosage, with statistically significant differences at each time point between low-medium, medium-high, and high-medium dose groups. The systemic clearance (CLs) were 0.4 ± 0.1, 0.2 ± 0.0, and 0.3 ± 0.1 mL/h/kg in the low-, medium-, and high-dose groups, respectively. The terminal half-life $T_{1/2}$ values were 164.8 ± 72.9, 169.4 ± 69.0, and 1532.9 ± 687.3 h, respectively, without significant difference between different dose groups. The dosage ratio of each dose group was 1:5:25, the increase in AUC was 1:8.9:41.9, and the increase in C_{max} was 1:5.2:24.8, which was higher than the dosage increase ratio; among dose groups, terminal half-life $T_{1/2}$ values increased with the increasing dosage, and the systemic clearance (CL) increased with the increasing dosage, suggesting a linear pharmacokinetic profile in cynomolgus monkeys within the range of 2–50 mg/kg.

Multiple intravenous infusions of HLX04 at single dosage

Cynomolgus monkeys received intravenous infusions of HLX04 (10 mg/kg) once a week for 4 consecutive weeks. The C_{max} values after the 1st and 4th administration were 235.7 ± 46.3 $\mu g.mL^{-1}$ and 281.1 ± 69.6 $\mu g.mL^{-1}$, respectively, showing no statistical difference (P > 0.05). The terminal half life $T_{1/2}$ values were 113.0 ± 74.7 h and 177.5 ± 158.9 h, respectively, with no statistical difference (P > 0.05). The AUC $_{(0–168\ h)}$ was 16537.5 ± 2988.1 and 23169.8 ± 8135.8 $\mu g.h.mL^{-1}$ after the 1st and 4th administration, respectively, and the accumulation factor (AUC at the 4th week/AUC at the 1st week) was 1.4 ± 0.4, suggesting that after continuous intravenous infusions of 10 $mg.kg^{-1}$ of HLX04, there was no accumulation in vivo.

Multiple intravenous infusions of Avastin at single dosage

Cynomolgus monkeys received intravenous infusions of the control Avastin (10 $mg•kg^{-1}$) once a week for 4 consecutive weeks. The C_{max} values after the 1st and 4th administration were 227.0 ± 47.3 $\mu g.mL^{-1}$ and 308.6 ± 104.2 $\mu g.mL^{-1}$, respectively, showing no statistical difference (P > 0.05). The terminal half-life $T_{1/2}$ values were 100.6 ± 102.1 h and 277.5 ± 219.2 h, respectively, with no statistical difference (P > 0.05). The AUC $_{(0–168\ h)}$ was 16730.6 ± 4068.0 and 30450.4 ± 15370.4 $\mu g.h.mL^{-1}$ after the 1st and 4th administration, respectively, and the accumulation factor (AUC at the 4th week/AUC at the 1st week) was 1.7 ± 0.7, suggesting that after continuous intravenous infusions of 10 $mg.kg^{-1}$ of Avastin, there was no accumulation in vivo.

After the first and last administration, there was no statistical significance in AUC $_{(0–168\ h)}$, CL, Vss, C_{max}, $T_{1/2}$, and MRT between the investigational product HLX04 and the control Avastin (P > 0.05).

1.2.3.3 Toxicokinetic testing of HLX04

The toxicokinetic study conducted concomitantly with repeated intravenous injection of HLX04 and Avastin in cynomolgus monkeys for 4 weeks followed by a 4-week recovery period showed that HLX04 injection and Avastin administered by intravenous injection at the same dosage were basically consistent in toxicokinetic.

1.2.3.4 Toxicology study of HLX04

The effects of HLX04 on nervous system of mice and cardiovascular system and respiratory system of cynomolgus monkeys were investigated through the evaluation of two safety pharmacology indicators in mice and cynomolgus monkeys, respectively. The test results indicated that single intravenous injection of 5.0 mg/kg, 25.0 mg/kg, and 125.0 mg/kg of HLX04 had no significant effect on nervous system of mice; single intravenous injection of 2.0–50.0 mg/kg of HLX04 had no significant effect on cardiovascular system and respiratory system of cynomolgus monkeys.

Acute toxicity study

HLX04 group and vehicle control group were established with 20 rats (half male and half female) in each group and administered with HLX04 and the vehicle placebo 1000 mg/kg respectively for a 14-day observation. The results showed that after intravenous injection of HLX04 and placebo in rats, no significant abnormalities were observed. It suggested that MTD in rats receiving intravenous injection of HLX04 was ≥ 1000 mg/kg.

Six healthy cynomolgus monkeys (both male and female) were administered with single intravenous injection of HLX04 at the dosages of 74 mg/kg, 111 mg/kg, 167 mg/kg, and 250 mg/kg, respectively, by dose-escalation method (50%). A vehicle control group was also established and given the placebo. Serum chemistry, hematology, urine routine, ECG, histopathology, general symptoms and other indicators were detected on D0, D1, D7, and D14. The results showed that the NOAEL was 111 mg/kg after single intravenous injection of 74–250 mg/kg of HLX04 in cynomolgus monkeys. Primary toxicities include weight decreased, TG increased, WBC increased, NEUT increased, LUC increased, FIB increased, LYMPH decreased, MONO decreased, EOS decreased, and TT extended. Target organs of toxic action may be primarily digestive system and hematologic system.

Chronic toxicity study

The toxicities of HLX04 in cynomolgus monkeys after intravenous injection twice a week for 4 consecutive weeks and the recovery after discontinuation for 4 weeks were observed, and toxicokinetic tests were conducted concomitantly. Five groups were established, namely, vehicle control group, active control group, low-dose group, medium-dose group, and high-dose group. There were 6 cynomolgus monkeys (half male and half female) in each group. The dosages of HLX04 in the low-dose, medium-dose, and high-dose groups were 2 mg/kg, 10 mg/kg, and 50 mg/kg, respectively; 50 mg/kg of Avastin was administered to the active control group; and the placebo was administered to the vehicle control group. The drugs were administered twice a week for 4 cycles, with a 4-week recovery period. Serum chemistry, hematology, urine routine, ECG, histopathology, general symptoms and other indicators were detected. The results demonstrated that, in the case of intravenous injection of HLX04 at the dosage of 2–50 mg/kg in cynomolgus monkeys, the safe dosage was 2 mg/kg, and the toxic dosage was 10 mg/kg. Main toxicities in animals were femoral growth plate chondrocyte proliferation, calcification and osteogenesis imperfecta, and proximal vascular and perivascular injuries and inflammations at administration sites. Target organs of toxic action were mainly femoral epiphyses and administered sites. The action was the extension and amplification of the pharmacological effects of HLX04. Attention should also be paid to the possible effects of HLX04 on the digestive system (liver). Under the test conditions, HLX04 resembled the active control, bevacizumab, at an equal dosage in toxicity level and profile.

1.2.3.5 HLX04 immunogenicity and immunotoxicity

The results of the concomitant test of the chronic toxicity study in cynomolgus monkeys

intravenously injected with HLX04 or the control bevacizumab once weekly for 4 weeks followed by a recovery period of 4 weeks showed that no specific antibodies were produced against HLX04 in different individuals, which was consistent with the results of the control bevacizumab group.

The immunotoxicity of HLX04 in cynomolgus monkeys after continuous intravenous injections twice a week for 4 weeks and the recovery after discontinuation for four weeks were observed. The results showed that after intravenous injection of 2–50 mg/kg of HLX04 injection in cynomolgus monkeys, no significant immunotoxicity was observed under the test conditions.

1.2.3.6 Other preclinical studies of HLX04

The local irritative reaction of the recombinant anti-VEGF humanized monoclonal antibody injection intravenously injected in cynomolgus monkeys twice a week for four consecutive weeks and the recovery after discontinuation for four weeks were observed. The results showed that under the test conditions, after intravenous injections of 2–50 mg/kg of HLX04 injection in cynomolgus monkeys, local irritation might be present at medium and high dosages (10 mg/kg, 50 mg/kg), and such action was somewhat related to the pharmacological effects of the investigational product HLX04 itself, with level and profile of the action being similar to those of the active control bevacizumab at an equal dosage.

Immunohistochemistry method was used to detect whether HLX04 could specifically bind to normal human tissues and normal cynomolgus monkey tissues, i.e., cross reactivity, and the results were compared with those of the commercially available drug product Avastin (bevacizumab). The results showed that both the investigational product HLX04 and the active control bevacizumab could specifically bind to human colon cancer tissues highly expressing VEGF; and HLX04 and bevacizumab did not specifically bind to normal human tissues.

The results of hemolysis test of HLX04 demonstrated that the recombinant anti-VEGF humanized monoclonal antibody injection has no hemolytic effect under the test conditions.

1.2.4 Evaluation of anti-tumor efficacy of HLX10 combined with HLX04 in the model of NOD/SCID mice

In the tumor model of NOD/SCID mice, treatment of HLX04 in combination with HLX10 significantly inhibited tumor growth, and the efficacy was substantially superior to that of HLX04 or HLX10 alone (Figure 1.).

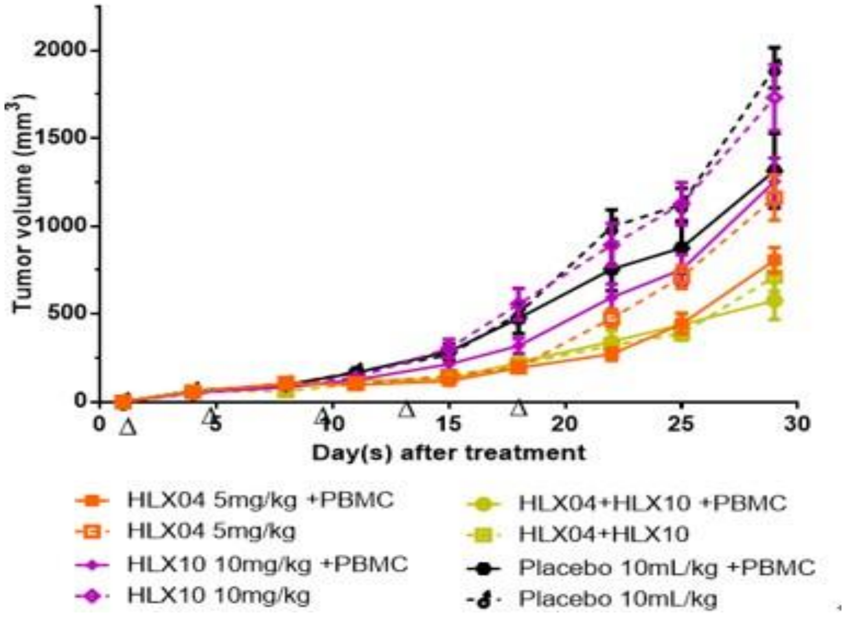

Figure 2 Efficacy of HLX04 combined with HLX10 in the mouse tumor model

1.2.5 Clinical studies on HLX10 and HLX04

HLX10 has been approved by the FDA, TFDA, and NMPA for a dose escalation phase I clinical trial. This is a prospective, open-label, and dose-escalation phase I study of HLX10 in patients with metastatic or recurrent solid tumors who have failed standard treatment. The study will use a Bayesian Optimal Interval Design (BOIN) to determine the MTD of HLX10. The safety, PK profile, biomarkers, PD markers, immunogenicity, as well as the preliminary efficacy will also be examined. Patients who meet the inclusion criteria during screening will be infused with a specified dosage of HLX10 once every 2 weeks until progressive disease, one year of treatment, withdrawal from the study, or death, whichever occurs first. Each treatment cycle consists of 2 bi-weekly doses of the investigational products. This study plans to enroll a maximum of 30 patients. Patients who withdraw from the study for reasons other than DLT prior to completion of Cycle 1 will be replaced.

There are four dose groups (0.3, 1, 3, and 10 mg/kg, once every 2 weeks) in the study, and patients of 0.3, 1, and 3 mg/kg dose groups have already been enrolled and completed safety evaluation. The safety evaluation for 10 mg/kg dose group is undergoing.

The study also measured the PD-1 receptor occupancy on T cells. The results showed that the receptor occupancy on T cells reached more than 90% in 0.3 mg/kg and 1 mg/kg dose groups.

HLX04 was approved for clinical trials in the treatment of metastatic colorectal cancer in December 2015 and non-squamous non-small cell lung cancer in May 2016, with approval numbers of 2015L05219 and 2016L04811, respectively. The phase I head-to-head clinical trial

(healthy population) between HLX04 and bevacizumab has been completed and the phase III clinical trial (advanced colorectal cancer) is undergoing. The results of the phase I clinical trial showed that HLX04 enjoys adequate safety, and that the PK data of Avastin® marketed in China, the EU, and the US have no significant difference.

The first-in-human dose-escalation trial (phase I) of HLX10 combined with HLX04 is planned to evaluate the safety and tolerance of HLX10 combined with HLX04 in the treatment of patients with advanced or metastatic solid tumors who have failed standard treatment. In addition, it also aims to determine the maximum tolerated dosage of the combined treatment, to investigate the pharmacokinetics, pharmacodynamics, and immunogenicity of HLX10 combined with HLX04, to assess the initial efficacy of the combined treatment, and to explore potential prognosis and predictive biomarkers.

The phase I dose escalation trial of HLX10 combined with HLX04 in patients with advanced digestive system tumors has been initiated. The dosages selected are 1, 3, and 10 mg/kg/2 weeks for HLX10; and a fixed dosage of 5 mg/kg/2 weeks of HLX04 was used according to the package insert of bevacizumab in treating gastrointestinal tumors. Three subjects are planned to be enrolled in each dose group, and no more than 30 subjects are planned to be enrolled in the whole trial.

Currently, patients in the HLX10 1 mg/kg/2 weeks + HLX04 5 mg/kg/2 weeks dose group have already been enrolled and completed safety evaluation. The safety evaluation for HLX10 3 mg/kg/2 weeks + HLX04 5 mg/kg/2 weeks dose group is undergoing.

1.2.6 Rationale for selection of chemotherapy as control group

The platinum-based chemotherapy provided in this study is carboplatin-pemetrexed, which is the first-line chemotherapy regimen for non-squamous NSCLC recommended by NCCN and "China Experts Consensus on the Diagnosis and Treatment of Advanced Stage Primary Lung Cancer (2016 Version)", allowing investigators and subjects to have more flexibility in conducting study and treatment according to the standard clinical practice of drugs.

1.2.7 Rationale for combined treatment

With the deepening of the study on the molecular pathogenesis of cancer, the immunological mechanism of tumorigenesis and development has gradually become a study hotspot. It is becoming increasingly clear that cancer can be recognized by the immune system, and in some cases, the immune system can control or even eliminate tumors[6]. PD-1 was originally cloned by Ishida et al.[7] as a member of the CD28 superfamily in murine T cell hybridomas. It is a monomeric glycoprotein that is mainly expressed on the surface of activated macrophages, T lymphocytes, B lymphocytes, NK cells, and some myeloid cells. Its ligands, PD-L1 (programmed cell death-ligand 1) and PD-L2, are mainly expressed on tumor cells and antigen-

presenting cells[8][9][10]. Because activation of the PD-1 gene may be involved in the classical type of programmed cell death, it is named programmed cell death protein 1 (PD-1). PD-L1 is highly expressed in a variety of cancers and is as high as 88% in some cancers. In these cancers (including lung cancer), binding of PD-1 to PD-L1 in tumor tissues will attenuate immune response of an organism, and protect tumor tissues from attack of cytotoxic T cells, resulting in tumor immune tolerance[11][12]. Therefore, anti-tumor response of T cells can be enhanced by blocking the binding of PD-1 to its ligand PD-L1[13].

In recent years, with the rapid development of tumor immunotherapy, the focus of first-line treatment for advanced NSCLC with no driver gene has gradually turned to immunotherapy, and multiple PD-1 inhibitors have been approved by the FDA for NSCLC treatment. Meanwhile, several clinical trials of PD-1/PD-L1 inhibitor in combination with chemotherapy for the treatment of NSCLC are ongoing, and the FDA has accelerated the approval of pembrolizumab in combination with pemetrexed/carboplatin as the first-line treatment for metastatic/progressive stage non-squamous NSCLC. The PD-1/PD-L1 inhibitor in combination with chemotherapy is expected to become the new standard of the first-line treatment for NSCLC.

In addition, tumor formation is closely related to the increase of vascular endothelial growth factor (VEGF) in the tumor microenvironment. The increased VEGF expression can decrease the function of T lymphocytes in the tumor microenvironment, thus inhibiting the anti-tumor immune responses. The antiangiogenic therapy increases T lymphocyte trafficking and infiltration and enhances anti-tumor immune responses in the tumor microenvironment. Therefore, the combined antiangiogenic therapy has the potential to increase the sensitivity of NSCLC patients to the first-line immunotherapy. Meanwhile, the cytotoxicity effect of chemotherapy on tumor cells will expose more tumor antigens, which is beneficial to enhance the recognition effect related to the immunotherapy. Therefore, it is reasonable to assume that the combination of immunosuppressive drugs, anti-angiogenesis drugs, and chemotherapy may have a stronger anti-tumor synergistic effect.

1.2.8 Rationale for HLX10 and HLX04 dose selection

HLX04 is a biosimilar of bevacizumab. The results of HLX04 preclinical studies showed that HLX04 exhibited a linear pharmacokinetic profile within the range of 2–50 mg/kg, which was very similar to the pharmacokinetic behavior of bevacizumab; HLX04 was equivalent to the same dosage of bevacizumab in efficacy, and these two drugs are also similar in toxicity level and profile. The package inserts recommended dosage of bevacizumab when used in combination with carboplatin/paclitaxel for the first-line treatment of non-squamous NSCLC indication is 15 mg/kg. In the IMPower150 study, subjects treated with bevacizumab 15 mg/kg plus carboplatin/paclitaxel combined with or without atezolizumab achieved good efficacy, and the bevacizumab plus carboplatin/paclitaxel plus atezolizumab group showed statistically and clinically significant benefits in PFS as well as good safety and tolerance compared with the

bevacizumab plus carboplatin/paclitaxel group. Therefore, the dosage of HLX04 was also 15 mg/kg in combination with carboplatin/pemetrexed plus HLX10 in this phase III clinical study.

Based on the results of the HLX10 preclinical and phase I clinical trials, the currently available PK and ADA data support an average body weight dosage of HLX10 of 4.5 mg/kg administered every 21 days as the recommended dosage for phase III clinical studies.

1.3 Assessment for Risks and Benefits

1.3.1 Potential benefits

A randomized, open-label, multicenter phase II clinical study of carboplatin and pemetrexed combined with or without pembrolizumab (trade name: Keytruda) in the treatment of advanced non-squamous non-small cell lung cancer was conducted in 2014 by Merck Sharp & Dohme. A total of 123 patients were included in the KEYNOTE-021[15] trial, and randomly divided into pembrolizumab plus chemotherapy group (60 patients) and chemotherapy alone group (63 patients). Results: The progression-free survival was significantly longer for pembrolizumab plus chemotherapy than for chemotherapy alone (HR = 0.53 [95% CI 0·31 –0·91]; p = 0.010). The median progression-free survival was 13.0 months (95% CI, 8.3 months to not reached) for pembrolizumab plus chemotherapy, and 8.9 months (4.4 to 10.3) for chemotherapy alone. Based on this study, the FDA accelerated the approval of pembrolizumab (Keytruda) in combination with pemetrexed and carboplatin for the treatment of previously untreated patients with advanced NSCLC (non-squamous cancer) without mutation on 10 May 2017, bringing the treatment of lung cancer with immune monoclonal antibody to a new stage, namely a new treatment mode of combined chemotherapy.

A further randomized, double-blind phase III clinical study, KEYNOTE-189 trial, was conducted later to further evaluate the efficacy of pembrolizumab in combination with chemotherapy (carboplatin/cisplatin + pemetrexed) versus chemotherapy (carboplatin/cisplatin + pemetrexed) in the first-line treatment of patients with metastatic non-squamous NSCLC. In this study, 614 subjects were enrolled and randomly assigned in a 2:1 ratio to the pembrolizumab plus chemotherapy group or placebo plus chemotherapy group. As of 8 Nov. 2017, results at the median follow-up period of 10.5 months showed that patients in the pembrolizumab plus chemotherapy group had not reached median OS compared with 11.3 months in the placebo plus chemotherapy group. Patients in the combined treatment group had a 51% reduction in the mortality risk (HR = 0.49) compared with the chemotherapy group, with a 58% reduction in mortality for patients of the high PD-L1 expression group (PD-L1 > 50%) (HR = 0.42); the median PFS was 8.8 months and 4.9 months, respectively.

Platinum-based chemotherapy in combination with the antiangiogenic inhibitor Bevacizumab has currently been approved for the first-line treatment of advanced non-squamous NSCLC.

Impower 150[15] is a randomized, open-label, controlled phase III clinical study to evaluate the efficacy and safety of PD-L1 monoclonal antibody atezolizumab + chemotherapy combined with or without bevacizumab in the treatment of stage IV non-squamous NSCLC. A total of 1202 subjects were randomly assigned in a 1:1:1 ratio to 3 treatment groups: atezolizumab + chemotherapy (group A), atezolizumab + chemotherapy + bevacizumab (group B), or chemotherapy + bevacizumab (group C, control group). The results showed that the median PFS was longer in group B than in group C (8.3 months versus 6.8 months; risk ratio for disease progression or death was 0.62; 95% CI, 0.52–0.74; P < 0.001); the response value was 11.3 months versus 6.8 months in the T cell wild-type population (hazard ratio, 0.51; 95% CI, 0.38–0.68; P < 0.001); and it showed longer progression-free survival and median overall survival in group B compared with group C (19.2 months versus 14.7 months; hazard ratio for death, 0.78; 95% CI, 0.64–0.96; P = 0.02) in the overall target treatment population and in patients with low PD-L1 expression and liver metastases; group B showed results consistent with previously reported safety risks for a single drug. In conclusion, the results of the trial suggested that combining PD- 1/PD-L1 inhibitors with antiangiogenic therapy might allow the NSCLC patients to benefit more from immunotherapy in the first-line treatment.

1.3.2 Identified and potential risks

PD-1/PD-L1 inhibitors not only enhance the anti-tumor effect of cellular immunity, but may also enhance the normal immune response, leading to immune tolerance imbalance and immune-related adverse events (irAEs). IrAEs can affect any organ in the human body, and currently nearly two-thirds of patients treated with immune checkpoint inhibitors have experienced irAEs of different degrees. In Feb. 2018, NCCN and ASCO jointly issued the Guidelines for Management of Immunotherapy-Related Toxicities, which states that the irAEs of the skin, intestine, endocrine, lung, and musculoskeletal systems are relatively common, while the irAEs of cardiovascular, hematological, renal, neurological, and ophthalmic systems are rare. Most irAEs were mild to moderate in severity; common irAEs currently known in patients treated with PD-1/PD-L1 inhibitors include skin toxicity (mainly maculopapular rash and pruritus; 30% to 40%), diarrhea and/or colitis (8% to 19%), fatigue (16% to 24%), immune-related hepatitis (5%), hypothyroidism (4% to 10%), hyperthyroidism (4%), hypophysitis (< 1%), type 1 diabetes, immune-related pneumonia, sarcoidosis, inflammatory arthritis, etc. Others such as cardiovascular adverse events, anemia, thrombocytopenia, nephritis, encephalopathy, leukodystrophy, post-reversible encephalopathy syndrome (PRES), peripheral motor and sensory neuropathy, uveitis, episcleritis, blepharitis, and acute pancreatitis occurred less frequently.

KEYNOTE-021 reported that the incidence of grade 3 or greater treatment-related adverse events was similar between the pembrolizumab plus chemotherapy group and the chemotherapy alone group, and the most common grade 3 or greater treatment-related adverse events in the pembrolizumab plus chemotherapy group were anemia, which occurred in 7 patients (12%), and decreased neutrophil count, which occurred in 3 patients (5%); acute kidney injury, decreased

lymphocyte count, fatigue, neutropenia, septicemia, and thrombocytopenia each occurring in 6 patients (3%). The most common grade 3 or greater adverse events in the chemotherapy alone group were anemia, which occurred in 9 patients (15%), as well as decreased neutrophil count, pancytopenia, and thrombocytopenia, which occurred in 2 patients each (3%). One of 59 patients (2%) in the pembrolizumab plus chemotherapy group died of sepsis, while 2 of 62 patients (3%) in the chemotherapy alone group died of sepsis, and 1 due to pancytopenia.

Impower 150 reported that the incidence of treatment-related adverse events was 94.4% for atezolizumab + chemotherapy + bevacizumab group (group B) and 95.4% for chemotherapy + bevacizumab group (group C, control group); the incidence of grade 1 or 2 treatment-related adverse events was 35.9% for atezolizumab + chemotherapy + bevacizumab group (group B) and 45.4% for chemotherapy + bevacizumab group (group C, control group); the common treatment-related grade 3 or 4 adverse events were neutropenia, decreased neutrophil count, febrile neutropenia, and hypertension. The incidence of rash, stomatitis, febrile neutropenia, and hemoptysis was less than 10% higher in patients of atezolizumab + chemotherapy + bevacizumab group (group B) than in patients of group C. Treatment-related adverse events occurred in 11 patients (2.8%) in the atezolizumab + chemotherapy + bevacizumab group (group B) and in 9 patients (2.3%) in the chemotherapy + bevacizumab group (group C, control group); and 5 deaths occurred in the atezolizumab + chemotherapy + bevacizumab group (group B), 4 of which occurred in patients with potentially high-risk features due to pulmonary hemorrhage or hemoptysis. The incidence of treatment-related serious adverse events was 25.4% in the atezolizumab + chemotherapy + bevacizumab group (group B) and 19.3% in the chemotherapy + bevacizumab group (group C, control group). The incidence of grade 1 or 2 immune-related adverse events was 77.4% in the atezolizumab + chemotherapy + bevacizumab group (group B), with no deaths. Common immune-related adverse events that occurred were rash, hepatitis, hypothyroidism, hyperthyroidism, pneumonia, and colitis.

1.3.3 Overall benefits: risk and ethics review

At present, chemotherapy for advanced NSCLC has encountered the bottleneck of efficacy, and it is urgent to explore more effective first-line treatment for NSCLC. Immune checkpoint has become a new treatment option for NSCLC, which can effectively improve the prognosis of NSCLC patients. At the same time, the adverse events of the pembrolizumab combination therapy are consistent with the known toxicity of monotherapy. Pembrolizumab in combination with chemotherapy (carboplatin-pemetrexed) has been approved for the first-line treatment of NSCLC on 10 May, 2017, and atezolizumab in combination with bevacizumab has been approved for the treatment of NSCLC. The adverse events of the combination protocol were consistent with the known toxicity of monotherapy, and no new safety signals were found. No DLT was observed based on the current first-in-human study data for HLX10, and the available safety data and pharmacokinetic data demonstrate that the safety of HLX10 in patients is

acceptable enough to support the implementation of this phase of clinical study.

2. OBJECTIVES

2.1. Stage I: A Single-Arm Study (Safety Run-In Phase)

Primary objective:

- To evaluate the safety and tolerability of HLX10 + HLX04 combined with chemotherapy as first-line treatment in patients with advanced non-squamous non-small cell lung cancer.

Secondary objective:

- To evaluate the clinical efficacy of HLX10 + HLX04 combined with chemotherapy as first-line treatment in patients with advanced non-squamous non-small cell lung cancer.

2.2. Stage II: Phase III Study

Primary objective:

- To evaluate the clinical efficacy of HLX10 combined with chemotherapy versus HLX10 + HLX04 combined with chemotherapy as first-line treatment in patients with advanced non-squamous non-small cell lung cancer.

Secondary objective:

- To evaluate the safety and tolerability of HLX10 combined with chemotherapy versus HLX10 + HLX04 combined with chemotherapy as first-line treatment in patients with advanced non-squamous non-small cell lung cancer.

3. STUDY DESIGN

3.1. Overall Study Design

This study has two stages:

The first stage is a single-arm study and a safety run-in phase. Approximately 6–12 subjects with advanced non-squamous NSCLC will be enrolled in this stage. Six subjects will be enrolled for the first time and treated with HLX10 + HLX04 combination chemotherapy (carboplatin - pemetrexed). After all subjects complete the first cycle of study treatment, the safety and tolerability will be confirmed by the Steering Committee (SC) to determine whether to proceed to the second stage of phase III randomized, double-blind, multicenter study. The specific safety assessment process is as follows:

Study Flow Chart of Stage I

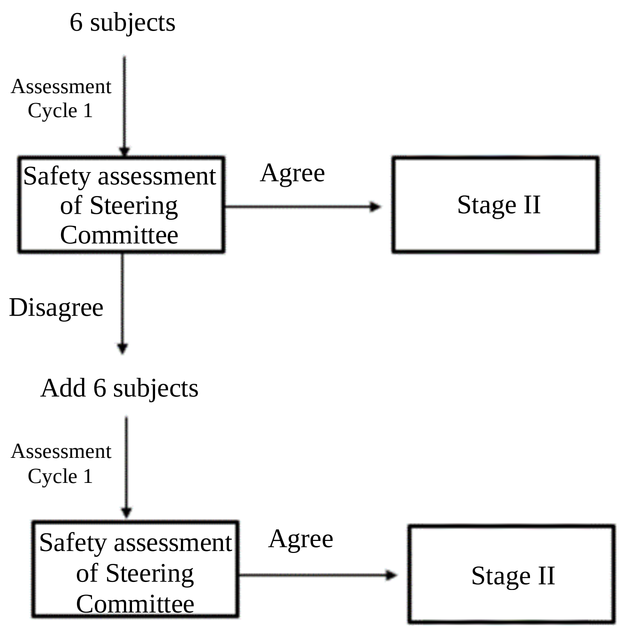

Safety evaluation will be conducted according to the following criteria, and any of the following events should be recorded as "safety events":

- ✓ Grade ≥ 4 hamatological toxicity:
  - ✧ Grade ≥ 4 neutropenia;
  - ✧ Grade ≥ 4 thrombocytopenia;
  - ✧ Other grade ≥ 4 hematological toxicities.
- ✓ Grade ≥ 3 non-hematological toxicity:
  - ✧ Grade ≥ 3 nausea, vomiting, fatigue, rash and diarrhea, which did not recover to grade ≤ 2 within 7 days;
  - ✧ Grade ≥ 3 injection site AE;
  - ✧ Grade ≥ 3 abnormal cardiac function, which did not recover to ≤ grade 2 within 7 days;
  - ✧ Grade ≥ 3 acute AE or organ injury related to immunotherapy;
  - ✧ Other grade ≥ 3 non-hematological toxicities (except alopecia, electrolyte imbalance and other reactions with no clinical significance), which did not recover to ≤ grade 2 within 7 days.
- ✓ Pneumonia (any grade ≥ 2 pneumonia, not recovered to grade ≤ 1 within 3 days).

If "safety events" occurs in ≤ 1 of the 6 subjects, it is considered that the safety is good and the second period can be entered. If "safety events" occur in ≥ 2 of 6 subjects, 6–12 subjects will be

enrolled additionally. If "safety events" occurs in < 4 of the 12 subjects, they will enter the second period. If "safety events" occurs in ≥ 4 of the 12 subjects, the current dose study will be terminated, and SC will decide whether to reduce the dose for exploration.

If progressive disease occurs (the first PD, PD confirmed by IRRC as per RECIST v1.1) in a subject, the investigator will determine whether to **continue treatment**: after the occurrence of the first PD, **continue the treatment as per the original regimen** for a maximum of 6 weeks and then perform tumor assessment again; if tumor progression occurs again (the second PD), end the study treatment for the subject and proceed to the follow-up period; if no progression occurs, continue the treatment as per the **original regimen**.

The second stage is a three-arm, randomized, double-blind, multicenter phase III clinical study to evaluate the clinical efficacy, safety, and tolerability of HLX10 combined with chemotherapy versus HLX10 + HLX04 combined with chemotherapy versus chemotherapy in subjects with non-squamous non-small cell lung cancer (NSCLC) who have not previously received systemic therapy for advanced NSCLC, collect PK parameters, and explore the biomarkers related to efficacy.

3.2. Endpoints

3.2.1 Stage I endpoints

Primary endpoint:

- Safety and tolerability of the first cycle of study treatment;

Secondary endpoints:

- Incidence rates of adverse events (AEs) and serious adverse events (SAEs);
- Overall survival (OS);
- Progression-free survival (PFS) (assessed by IRRC and the investigator as per RECIST v1.1, respectively);
- Objective response rate (ORR, assessed by IRRC and the investigator as per RECIST v1.1);
- Duration of response (DOR, assessed by IRRC and the investigator as per RECIST v1.1);
- Pharmacokinetics (PK): serum HLX10/HLX04 concentration;
- Immunogenicity assessment: positive rate of anti-drug antibody (ADA);
- Relationship between PD-L1 expression level, MSI, TMB in tumor tissues and efficacy;
- Quality of life assessment.

3.2.2 Stage II endpoints

Primary endpoint:

- Progression-free survival (PFS, assessed by IRRC as per RECIST v1.1);

Secondary endpoints:

- Overall survival (OS), as a key secondary endpoint in this study;
- Progression-free survival (PFS, assessed by the investigator as per RECIST v1.1);
- Objective response rate (ORR, assessed by IRRC and the investigator as per RECIST v1.1);
- Duration of response (DOR, assessed by IRRC and the investigator as per RECISTv1.1);
- Incidence rates of adverse events (AEs) and serious adverse events (SAEs);
- Pharmacokinetics (PK): serum HLX10/HLX04 concentration;
- Immunogenicity assessment: positive rate of anti-drug antibody (ADA);
- Relationship between PD-L1 expression level, MSI, TMB in tumor tissues and efficacy;
- Quality of life assessment.

3.3. Randomization, Blinding, and Unblinding

A randomized, double-blind design is employed in stage II of this trial. Eligible subjects are randomly allocated to the following 3 groups using an interactive web/voice response system (IWRS/IVRS) in a 1:1:1 ratio:

- **Group A (HLX10 + HLX04)**: HLX10 + HLX04 combined with chemotherapy (carboplatin-pemetrexed)
- **Group B (HLX10)**: HLX10 + HLX04 placebo combined with chemotherapy (carboplatin-pemetrexed)
- **Group C (control group): HLX10 placebo + HLX04 placebo combined with chemotherapy (carboplatin-pemetrexed)**

Randomization is stratified by: PD-L1 expression level (negative, positive, or not evaluable), smoking history (yes versus no), and brain metastasis (yes versus no).

During the study, the subjects, the investigator, the sponsor, and the designees are not aware of the randomized allocation, with the exception of the need for emergency unblinding or the initiation of treatment after disease progression.

3.3.1 Emergency unblinding

During the treatment with the investigational product, if the investigator determines that the investigational product is related to a life-threatening situation of a subject, and the investigator

considers that knowing the medication of the subject is conducive to the handling of AEs, then an emergency unblinding is allowed. The decision to unblind in an emergency is the responsibility of the investigator and will not be delayed or declined by the sponsor; however, the investigator may contact the sponsor or its designees to discuss the unblinding and the protocol that is in the best interest of the subject prior to unblinding. The investigator shall ensure that unblinding is performed in strict accordance with the protocol. The investigator shall inform the sponsor of the circumstances of and reasons for emergency unblinding as soon as possible and record these details clearly on the subject’s source document. The unblinding process will be completed on IWRS using personal identification number for emergency unblinding. If deemed necessary, the unblinding shall only apply to the affected subject.

3.3.2 Unblinding for treatment after disease progression

In the course of investigational product treatment, if a subject develops progressive disease (the first PD, PD confirmed by IRRC according to RECIST v1.1 criteria), the investigator will perform unblinding, and the subject who meets certain criteria after unblinding can continue to receive treatment. See "Study Treatment Visit after Disease Progression" for the criteria of continuing medication after unblinding.

3.4. Number of Subjects

Approximately 6–12 subjects are planned to be enrolled in the stage I of this study. Approximately 630 subjects with advanced non-squamous NSCLC will be enrolled in stage II, 210 each in group A, group B, and group C.

3.5. Selection and Withdrawal of Subjects

3.5.1 Inclusion criteria

The subject has to meet all the following criteria before enrollment into this study:

1. Voluntary participation in clinical studies; fully understand, be informed about the study and have signed the informed consent form (ICF); willingness to follow and ability to complete all trial procedures;
2. Aged ≥ 18 years and ≤ 75 years at the time of signing the ICF;
3. Histologically or cytologically diagnosed with stage IIIB, IIIC, or IV (AJCC 8th edition) non-squamous non-small cell lung cancer that cannot be treated with surgery or radiotherapy;
4. With no EGFR sensitive mutation or ALK, ROS1 gene rearrangement ;

Note: Blood test results alone are not accepted.

5. Having not previously received systemic therapy for stage IIIB, IIIC, or IV NSCLC. Patients who have received adjuvant or neoadjuvant treatment are allowed to be enrolled if the adjuvant/neoadjuvant treatment has been completed at least 6 months before the diagnosis of stage IIIB, IIIC, or IV NSCLC;

6. With at least one measurable lesion as assessed by IRRC as per RECIST v1.1 within 4 weeks prior to randomization;

Note: Measurable lesions should not be from previously irradiated sites.

7. The subjects must provide tumor tissue to measure the PD-L1 expression level;

8. An ECOG PS score of 0 or 1 within 7 days prior to the first dose of the study drugs;

9. An expected survival of ≥ 12 weeks;

10. Normal major organ functions as defined by the following criteria (no blood transfusions, or treatment with albumin, recombinant human thrombopoietin or colony-stimulating factor (CSF) within 14 days prior to the first dose in this study):

**Hamatologic system**	
Absolute neutrophil count (ANC)	≥ 1.5 × 10^9/L
Platelet (PLT)	≥ 100 × 10^9/L
Haemoglobin (Hb)	≥ 90 g/L
**Liver function**	
Total bilirubin (TBIL)	≤ 1.5 × upper limit of normal (ULN)
Alanine transaminase (ALT)	≤ 2.5 × ULN;<br>≤ 5.0 × ULN for patients with liver metastases
Aspartate transaminase (AST)	≤ 2.5 × ULN;<br>≤ 5.0 × ULN for patients with liver metastases
**Renal function**	
Creatinine (Cr)	≤ 1.5 × ULN;<br>In case of > 1.5 × ULN, creatinine clearance ≥ 50 mL/min (calculated using Cockcroft-Gault formula)
**Coagulation function**	
Activated partial thromboplastin time (APTT)	≤ 1.5 × ULN
International normalised ratio (INR)	≤ 1.5 × ULN
**Urinalysis/24-h urine protein**	
Urine protein	Urine protein ≤ 1+;<br>In the case of ≥ 2+, a 24-hour urine protein test is required, and subjects with 24-hour urine protein of < 1 g are allowed to be enrolled

11. Female patients must meet one of the following conditions:

1) Menopause (defined as no menses for at least 1 year with no confirmed cause other than menopause), or
2) Surgically sterilized (removal of the ovaries and/or uterus), or
3) With child-bearing potential, but must:

- be tested negative for serum pregnancy test within 7 days prior to the first dose, and
- agree to use contraception with an annual failure rate of < 1% or to remain abstinent (avoid heterosexual intercourse) from signing the informed consent form to at least 120 days after the last dose of the investigational product and at least 150 days after the last dose of chemotherapy medication (contraception methods with an annual failure rate of < 1% include bilateral tubal ligation, male sterilization, correct use of hormonal contraceptives that inhibit ovulation, hormone-releasing intrauterine devices, and copper-containing intrauterine devices) , and
- not breastfeed;
- Male patients must: agree to remain abstinent (avoid heterosexual intercourse) or take contraception measures as follows: male patients with a pregnant partner or a partner with childbearing potential must remain abstinent or use a condom for at least 150 days after treatment to prevent the drug exposure to the embryo. The reliability of abstinence should be evaluated based on clinical trial duration and patient's preference and lifestyle. Periodic abstinence (e.g., contraception based on calendar day, ovulatory phase, basal body temperature, or postovulatory phase) and external ejaculation are ineligible methods of contraception.

3.5.2 Exclusion criteria

The subject meeting any of the following criteria may not be included in this study:

1. Subjects with non-small cell lung cancer of other histopathological types, including subjects with mixed adenosquamous carcinoma and NSCLC subjects with small cell lung cancer and neuroendocrine carcinoma;
2. Subjects with other active malignancies within 5 years or at the same time. Localized tumors that have been cured such as basal cell carcinoma, squamous-cell skin cancer, superficial bladder carcinoma, prostate carcinoma in situ, cervical cancer in situ, and breast cancer in situ are acceptable;
3. Subjects who are preparing for or have received an organ or bone marrow transplant;
4. Patients with pleural or pericardial effusions or ascites requiring clinical intervention;
5. Patients with known or documented active central nervous system (CNS) metastases and/or

carcinomatous meningitis at screening. However, the following subjects are allowed to be enrolled: 1) Subjects with asymptomatic brain metastases (i.e., no progressive central nervous system symptoms caused by brain metastases, no requirement for corticosteroids, and lesion size ≤ 1.5 cm) may be included, but are required to receive regular brain imaging as a site of lesion. 2) Subjects with treated brain metastases which have been stable for at least 1 month, with no evidence of new or enlarging brain metastases, and with steroids discontinued 3 days prior to administration of the study drugs. Stable brain metastases here should be confirmed before the first dose of the study drugs;

6. Subjects with spinal cord compression that has not been radically treated with surgery and/or radiotherapy;
7. Patients with myocardial infarction and poorly controlled arrhythmia (including QTc intervals ≥ 450 ms for males and ≥ 470 ms for females) (QTc intervals are calculated by Fridericia's formula) within half a year prior to the first dose of the study drugs;
8. Class III to IV cardiac insufficiency according to NYHA classification or an LVEF (left ventricular ejection fraction) < 50% by cardiac color Doppler;
9. Subject with peripheral neuropathy ≥ Grade 2 by CTCAE;
10. With human immunodeficiency virus (HIV) infection;
11. With active pulmonary tuberculosis;
12. Subjects with previous and current interstitial pneumonia, pneumoconiosis, radiation pneumonitis, drug-related pneumonitis, and severe impaired pulmonary function that may interfere with the detection and management of suspected drug-related pulmonary toxicity as judged by the investigator;
13. With Hepatitis B (positive test for HBsAg or HBcAb and positive test for HBV-DNA) or Hepatitis C (positive tests for HCV antibody and HCV-RNA). With Hepatitis B and C co-infection (positive test for HBsAg or HBcAb and positive test for HCV antibody);
14. Patients with known active or suspected autoimmune diseases. Subjects in stable state and requiring no systemic treatment with immunosuppressive agents are included;
15. Have received treatment with live vaccines within 28 days prior to the first administration of the study drugs; but inactivated viral vaccines for seasonal influenza are allowed;
16. Subjects requiring treatment with systemic corticosteroids (> 10 mg/day prednisone efficacy dosage) or other immunosuppressive drugs within 14 days prior to the first administration of the study drugs or during the study. However, subjects are allowed to be enrolled under the following conditions: in the absence of active autoimmune disease, subjects are allowed to

use topical or inhaled steroids and adrenal hormone replacement therapy at dosages equivalent to ≤ 10 mg/day of prednisone efficacy;

17. With any active infection requiring systemic anti-infective therapy within 14 days prior to the first administration of the study drugs;

18. Have received major surgery within 28 days prior to the first dose of study drugs; major surgery in this study is defined as: any surgery which requires at least 3 weeks of postoperative recovery time before receiving the study treatment. Patients with a history of tumor needle biopsy or lymph node incisional biopsy are included;

19. Having received radical radiation therapy within 3 months prior to the first dose of study drugs;

Note: Palliative radiotherapy to bone or palliative radiotherapy to superficial lesions is allowed according to local standards 2 weeks prior to the first dose. Radiotherapy covering more than 30% of the bone marrow area within 28 days prior to the first dose is not allowed.

20. Subjects may receive other anti-tumor therapies during the study, such as chemotherapy, targeted therapy, or radiotherapy (except palliative radiotherapy);

21. The subject has previously received other antibodies/drugs against immune checkpoints, such as PD-1, PD-L1, CTLA-4, etc.

22. Patients who have received any treatment with bevacizumab or its biosimilars;

23. Be in any other ongoing clinical study, or the end of the previous clinical study treatment is less than 14 days from the planned start of this study;

24. With a known history of severe allergy to any monoclonal antibody;

25. With known hypersensitivity to any of the carboplatin or pemetrexed components;

26. Pregnant or lactating women;

27. Inadequately controlled hypertension (systolic blood pressure (BP) ≥ 150 mmHg and/or diastolic blood pressure ≥ 100 mmHg);

28. With a history of hypertensive crisis or hypertensive encephalopathy;

29. With any significant vascular disease (e.g., aortic aneurysm requiring surgical repair or with recent peripheral arterial thrombosis) within 6 months prior to the first dose of the study drugs;

30. With manifestations of hemorrhage (including hemoptysis, abnormal vaginal bleeding, etc.), or Grade 2 hemorrhagic events within 3 months or Grade 3 or greater hemorrhagic events within 6 months prior to signing the informed consent form;

31. Currently use or recently have used (within 7 days prior to the first dose of study drugs) aspirin (> 325 mg/day) or dipyridamole, ticlopidine, clopidogrel, and cilostazol;

32. Currently using or recently have used (within 7 days prior to the first dose of the study drugs) full-dose oral or injectable anticoagulant or thrombolytic agents for therapeutic purposes; prophylactic anticoagulation therapy for an open intravenous infusion system is allowed so long as the drug activity results in INR and APTT ≤ 1.5 × ULN within 14 days prior to the start of the study treatment. Prophylactic use of low molecular weight heparin (i.e., enoxaparin, 40 mg/day) is allowed;
33. Subjects who require long-term treatment with daily administration of nonsteroidal anti-inflammatory drugs (NSAIDs); Occasional use of NSAIDs to relieve symptoms associated with medical conditions, such as headache or pyrexia, is allowed;
34. Subjects with the following gastrointestinal diseases:
    - Gastrointestinal perforation, abdominal fistula, or intra-abdominal abscess within 6 months prior to the signing of the informed consent form;
    - History of poorly controlled or recurrent inflammatory bowel disease (including ulcerative colitis and Crohn's disease);
    - Presence of active peptic ulcer or esophageal varices (moderate or severe);
35. Subjects with known history of psychotropics abuse or drug abuse or alcoholism; patients who have stopped drinking can be enrolled;
36. In the judgement of the investigator, subjects who have any other factors that may lead to a premature discontinuation.

3.5.3 Criteria for discontinuation

3.5.3.1 Reasons for discontinuation

A discontinuation of treatment means that the subject will no longer receive a study medication of this trial. Reasons for the discontinuation of treatment may include:

1. Poor compliance that has affected the efficacy and safety evaluation per the judgement of the investigator;
2. Intolerable AEs or SAEs that are inappropriate to continue the study treatment as judged by the investigator;
3. There is evidence of definite disease progression or deterioration, and it is judged by the investigator that it is not suitable to continue receiving investigational product treatment;
4. A delayed dosing of the investigational product meeting the criteria in Section 3.9.3;
5. Loss to follow-up or death;
6. Withdrawal of informed consent;

7. Treatment discontinuation requested by subjects;

8. Other reasons of discontinuation as determined by the investigator in the best interest of the subject.

9. After the duration of treatment reaches 2 years (35 cycles), the investigator will determine whether the subject should discontinue the study treatment.

3.5.3.2 Management of discontinuations

The reasons for discontinuations shall be documented in the eCRF.

If a subject's study treatment is discontinued for any reason, an end-of-treatment visit should be performed whenever possible.

For subjects whose treatment is discontinued for reasons other than withdrawal of informed consent, loss to follow-up, or death, safety follow-up visits and survival follow-ups are required, and assessments during the follow-up period are shown in Section 4.5; If a subject discontinues the study treatment due to reasons other than PD, then radiologic assessment should be further performed according to an established schedule, where possible, until PD, initiation of a new anti-tumor therapy, withdrawal of ICF, death, or end of study (whichever occurs first). All AEs present at the time of withdrawal will be followed up until the outcomes of such AEs.

In case of an enrolled subject's withdrawal for any reasons, no subject replacement is permitted.

3.6. Loss to follow-up

A subject will be considered lost to follow-up if the subject repeatedly fails to return for scheduled visits as scheduled and is unable to be contacted by the study site.

The following actions must be taken if a subject fails to return to the study site for a scheduled study visit:

- The study site must attempt to contact the subject to reschedule the visit as soon as possible (within the specified time window of visit), to counsel the subject on the importance of maintaining the visit schedule, and to ascertain whether the subject wishes to or should continue in the study.
- If a subject is judged as loss to follow-up, the investigator or the designated personnel must contact the subject again whenever possible (the investigator should make three phone calls if possible and send a registered letter to the mailing address last provided by the subject or use other local available methods to contact the subject when necessary). These attempts should be recorded in the subject's medical record/case report form (CRF).
- Should the subject continue to be unreachable, he or she will be considered to have withdrawn from the study.

3.7. End of Study

The end of study is defined as the observation of the number of deaths required for the final analysis of OS (see Section 4.10).

3.8. Early Termination of Study/Closure of Study Site

The study may be terminated prematurely due to the following reasons. Written permissions of both the principal investigator and the sponsor are required for the early termination of the study, and results of the study shall be reported in accordance with the requirements of the protocol.

1. The study is unlikely to be completed within an acceptable time frame due to difficulties in subject enrollment;
2. The investigator questions the safety of the drug during the study and concludes that further study would pose serious risks to the subjects;
3. The principal investigator and the sponsor believe that the number and severity of adverse events suggest a premature termination;
4. The efficacy fails to meet expectations, and it is not necessary to continue the clinical trial;
5. The study is revoked by regulatory authorities;
6. The sponsor has the right to terminate the study at a certain study site in the event of:
   - serious violations of ICH-GCP by the study site;
   - repeated serious protocol violations by the study site;

Upon the termination of the study, all related records shall be kept for future reference.

3.9. Study Treatment

3.9.1 Study drugs

Investigational product HLX10

Name	Recombinant humanized anti-PD-1 monoclonal antibody injection (HLX10)
Strength	100 mg (10 mL)/vial
Storage condition	Store at 2–8 °C away from light.
Manufacturer	Shanghai Henlius Biopharmaceuticals Co.,Ltd.
Supplier	Shanghai Henlius Biotech, Inc.

Investigational product HLX04

Name	Recombinant anti-VEGF humanized monoclonal antibody injection (HLX04)

Strength	100 mg (4 mL)/vial
Storage condition	Store at 2–8 °C away from light.
Manufacturer	Shanghai Henlius Biopharmaceuticals Co.,Ltd.
Supplier	Shanghai Henlius Biotech, Inc.

Control Placebo

An injection that is visually indistinguishable and does not contain any active ingredient of HLX10 or HLX04, supplied by Shanghai Henlius Biotech, Inc.

Other study drugs

Commercially available carboplatin and pemetrexed supplied by Shanghai Henlius Biotech, Inc. Please refer to the currently approved prescribing information of carboplatin and pemetrexed for information on formulation, preparation, storage, and administration.

3.9.2 Route of administration and dosage

Study drugs are administered as follows in every 3-week (21-day) cycle.

Investigational product:

- HLX10 or placebo, 4.5 mg/kg, IV infusion on day 1 of each cycle, infused over 30–90 min. Administer every 3 weeks (21 days). The treatment will be continued until loss of clinical benefit, or until the duration of treatment reaches 2 years (up to 35 dosing cycles), and then the investigator will decide whether to continue the treatment.
- HLX04 or placebo, 15 mg/kg, via intravenous infusion, and the first infusion lasts for 90 min (± 15 min), if the subject has good tolerability to the first infusion, the second infusion can be shortened to 60 min (± 15 min), and if the subject has good tolerability to 60 min (± 15 min), the subsequent infusion can be completed over 30 min (± 10 min). Administration on day 1 of each 3-week (21-day) cycle. The treatment will continue until loss of clinical benefit, or until the duration of treatment reaches 2 years (up to 35 dosing cycles), and then the investigator will decide whether to continue the treatment.

The administered dose of HLX10 or placebo, HLX04 or placebo is calculated according to the body weight of the subject. Before each dose, investigators should measure the body weight of the subjects to determine the administered dose. If the subject's weight changes ≤ 10% compared to the corresponding weight reference value at baseline, there is no need to recalculate the administered dose; if the weight change is > 10%, the administered dose should be calculated according to the new weight, at which point the new weight is used as the baseline value for subsequent weight measurements.

Other study drugs: Combination chemotherapy

- Pemetrexed: 500 mg/m^2, via intravenous infusion, and the infusion duration should exceed 10 min. Administering on day 1 of each 3-week (21-day) cycle. The treatment will continue until loss of clinical benefit, or until the duration of treatment reaches 2 years (up to 35 dosing cycles), and then the investigator will decide whether to continue the treatment.

The dose of pemetrexed is calculated according to the body surface area [body surface area (m^2) = 0.0061 × height (cm) + 0.0128 × body weight (kg) − 0.1529].

- Carboplatin: AUC = 5, up to a dosage of 800 mg, by intravenous infusion on Day 1 of each 3-week (21-day) cycle, for up to 4 cycles.

  Dosage of carboplatin shall be calculated according to the following Calvert formula:

  - Dosage of carboplatin (mg) = target AUC x [(CrCl (mL/min) + 25)]
  - Creatinine clearance (CrCl) is calculated according to the Corkroft-Gault formula based on the subject's most recent serum creatinine and body weight. Note: If CrCl calculated by the Corkroft-Gault formula is > 125 mL/min, CrCl shall be calculated using an alternative formula in accordance to the standards of the study site, or capped at 125 mL/min.

The creatinine clearance is calculated using the measured value of serum creatinine (mg/dL):

$$\text{Male: } \frac{(140-\text{Age}) \times \text{Weight (kg)}}{\text{Serum creatinine (mg/dL)} \times 72}$$

$$\text{Female: } \frac{(140-\text{Age}) \times \text{Weight (kg)}}{\text{Serum creatinine (mg/dL)} \times 72} \times 0.85$$

The creatinine clearance is calculated using the measured value of serum creatinine (μmol/ L):

$$\text{Male: } \frac{(140-\text{Age}) \times \text{Weight (kg)} \times 1.23}{\text{Creatinine } (\mu\text{mol/L})}$$

$$\text{Female: } \frac{(140-\text{Age}) \times \text{Weight (kg)} \times 1.23 \times 0.85}{\text{Creatinine } (\mu\text{mol/L})}$$

Refer to **Figure 3** "Schematic of study treatment" for the regimen of each treatment group.

The dose of chemotherapy drugs is calculated according to the body weight of the subjects. Investigators should measure the body weight of subjects before each dose. The administered dose of chemotherapy drugs can be calculated according to the body weight before administration or confirmed with reference to the dose calculation principle for investigational product HLX10 or HLX04.

On the day of administration in each treatment cycle, subjects are given HLX10 or placebo intravenously first, followed by intravenous infusion of HLX04 or placebo, with interval of at least 30 min; intravenous infusions of pemetrexed + carboplatin at last. Vital signs should be closely monitored during the administration. During the second period, HLX10 or placebo and HLX04 or placebo will be administered via a blinded infusion, and pemetrexed + carboplatin via

an open-label infusion. Treatment with the study drugs will continue until disease progression, intolerable toxicity, discontinuation decided by subject or investigator, death, withdrawal of consent, pregnancy, incompliance with protocol or procedure requirements, administrative reasons, or other reasons specified in the protocol, whichever occurs first. If carboplatin is not used due to toxicity or other reasons in a certain cycle, it is not counted as the number of combined chemotherapy cycles. After completing 4 cycles of carboplatin, even if the subject does not meet the above criteria, carboplatin will not be continued.

3.9.3 Dose modification

Starting from the beginning of HLX10 or placebo/HLX04 or placebo intravenous infusion, subjects shall be closely monitored for anaphylaxis that may occur within a few minutes. Infusion should be stopped immediately, and proper treatment should be performed in the event of severe hypotension, bronchospasm, or generalized rash/erythema. In case of mild symptoms (such as flushing or local skin reactions), drugs may be administered at a slower speed. For a life-threatening reaction, including anaphylaxis, hypersensitivity reactions, renal failure, severe cardiopulmonary events, and severe skin reactions, the medications shall be discontinued permanently.

The dosing window is ± 3 days from the scheduled date of administration (from the date of the first dose). Drugs administered outside the dosing window is considered a delayed dose, and subsequent doses shall be administered according to the actual date of last administration. During combined treatment, if a delay of more than 2 weeks is expected due to the toxicity of chemotherapy, only HLX10 or placebo/HLX04 or placebo will be administered until the toxicity returns to the standard of chemotherapy administration. Chemotherapy may be continuously suspended for a maximum of 6 weeks, otherwise the chemotherapy should be discontinued. If a delay of more than 2 weeks is expected due to the toxicity of HLX10 or placebo/HLX04 or placebo, only chemotherapy will be administered until the toxicity recovers to the HLX10 or placebo/HLX04 or placebo dosing criteria. HLX10 or placebo/HLX04 or placebo therapy may be continuously suspended for a maximum of 12 weeks, otherwise the HLX10 or placebo/HLX04 or placebo will be discontinued. In case of a delay due to toxicity with equivocal association, all the study drugs shall be synchronously delayed if the event is expected to return to re-dosing standards within 2 weeks.

3.9.4 Principles for HLX10 or placebo dose modification

In the event of HLX10 or placebo-related toxicity, a delay in HLX10 or placebo is allowed **rather than dose modification**. Subjects who miss a scheduled infusion should be actively contacted to arrange another visit with the least delay for administration. Administration of HLX10 or placebo may be delayed, but the subsequent dosing interval should be no more than 12 weeks. A dosing interval of more than 12 weeks is considered intolerable, where HLX10 or placebo will be permanently discontinued, and the subject should discontinue the treatment. If

treatment is delayed due to intolerance to HLX10 or placebo, the other study drugs should be administered as scheduled.

If an irAE occurs, it should be treated in accordance with the latest version of "NCCN Guidelines for Dose Modification and Treatment for Immune-Related Toxicity" or "CSCO Guidelines for Toxicity Management Related to Immune Checkpoint Inhibitors". The basic principles are suggested as follows:

Table 1 Basic principles for HLX10 or placebo dose modification

Adverse event severity grade	Dose modification
Grade 1	No dose modification is required
Grade 2	Suspend the administration of the investigational product/study protocol medication until grade 2 response is relieved to ≤ grade 1.<br>If the toxicity is aggravated, treatment is performed according to those suitable for grade 3 or grade 4 responses.<br>Once the dose of steroids is gradually reduced and the event is stabilized to ≤ grade 1, the investigational product can be resumed.<br>For patients with endocrine disorders who may require long-term or sustained steroid replacement therapy, they may resume the treatment with the investigational product under the following conditions:<br>1. The event has become stabilized and controlled.<br>2. The subject is clinically stable according to the judgement of the investigator or attending physician.<br>3. The dosage of prednisone or an equivalent dosage of other medications is ≤ 10 mg/day.
Grade 3	Based on individual toxicity, the investigational product may be permanently discontinued. Please refer to the following guideline.
Grade 4	Permanent discontinuation of the investigational product/study protocol.

3.9.5 Principles for HLX04 or placebo dose modification

Dose modification of HLX04 or placebo due to adverse events is allowed based on the following basic principles:

Table 2 Basic principles for HLX04 or placebo dose modification

**Dose level**	**HLX04 or placebo dosing regimen**
Initial dosage	15 mg/kg by intravenous infusion on Day 1 of each 3-week (21-day) cycle.
First dose reduction	7.5 mg/kg by intravenous infusion on Day 1 of each 3-week (21-day) cycle.

If the adverse event is relieved to grade 1 or baseline after the first dose reduction and there is no other toxicity after 6 weeks of study treatment at the reduction level, it is allowed to resume the treatment at the initial dosage again.

Dosage of HLX04 or placebo below 7.5 mg/kg will not be allowed, and HLX04 or placebo dosing can be delayed. Two consecutive doses of HLX04 or placebo will be administered up to 12 weeks apart, but a dosing interval of more than 12 weeks is considered intolerable, where HLX04 or placebo will be permanently discontinued. In the event of treatment delay due to

intolerance to HLX10 or placebo, other study drugs will be administered as scheduled.

In general, AEs suspected to be related to HLX04 or placebo (as graded by CTCAE V5.0) that are observed in the enrolled subjects may be handled by referring to the package insert of bevacizumab or by **following the standard practice at the study site**. Permanently discontinue HLX04 or placebo for:

- Gastrointestinal perforation (gastrointestinal perforation, gastrointestinal fistula, intra-abdominal abscess), fistula involving internal organs
- Wound dehiscence and wound healing complications requiring medical intervention
- Serious hemorrhage (i.e., requiring medical intervention)
- Severe arterial thromboembolic events
- Hypertensive crisis or hypertensive encephalopathy
- Reversible posterior leukoencephalopathy syndrome
- Nephrotic syndrome

Temporarily suspend HLX04 or placebo for:

- After at least 4 weeks when elective surgery is required, or it cannot be used within at least 4 weeks after surgery and before the wound is completely recovered
- Severe hypertension (blood pressure > 160 [systolic pressure] or 100 [diastolic pressure] for three consecutive days) not under the control by medical management
- Moderate (24-h urine protein 2.0-3.4 g) to severe (24-h urine protein ≥ 3.5 g) proteinuria pending further evaluation
- Severe infusion-related reactions (life-threatening infusion reactions such as air embolism, acute heart failure, pulmonary edema and anaphylactic shock)

3.9.6 Principles for chemotherapy dose modifications

In the event of intolerance to carboplatin/pemetrexed, dosages may be modified twice in accordance with the prescribing information of carboplatin and pemetrexed and local treatment standards, as detailed in **Table 3**

Table 3 Basic principles for dose modifications of carboplatin and pemetrexed

Dose level	Pemetrexed dosing regimen	Carboplatin dosing regimen
Initial dosage	500 mg/m^2 by intravenous infusion on Day 1 of each 3-week (21-day) cycle	AUC = 5, up to a dosage of 800 mg, Intravenous infusion on Day 1 of each 3-week (21-day) cycle

First dose reduction	75% of starting dosage	75% of starting dosage
Second dose reduction	50% of starting dosage	50% of starting dosage

In the event of treatment delay due to intolerance to HLX10 or placebo/HLX04 or placebo, chemotherapy will be conducted as scheduled. During the combined treatment, if a delay is expected due to the toxicity of chemotherapy, chemotherapy may be continuously suspended for a maximum of 6 weeks, otherwise, the chemotherapy should be discontinued. If the chemotherapy medications are discontinued for any reason other than disease progression and the subject has received at least 3 cycles of combination therapy, subjects with response or stable disease may continue to receive HLX10 or placebo/HLX04 or placebo therapy. The following are **recommended carboplatin and pemetrexed dose modifications for hematological and non-hematological toxicities.**

Hematological toxicities

At the start of each cycle, the neutrophil count must be ≥ 1.5 × 10^9/L and platelet count must be ≥ 100 × 10^9/L. Otherwise the treatment should be postponed for up to 6 weeks to provide a sufficient period for recovery. According to the guidelines of the American Society of Clinical Oncology (ASCO) and National Comprehensive Cancer Network (NCCN), growth factors can be given. At the beginning of subsequent cycles after recovery, dosages shall be modified based on the PLT and ANC nadirs of the previous cycle (for recommendations, refer to **Table 4**).

Table 4 Principles for dose modifications of carboplatin and pemetrexed for hematological toxicities

Toxicity[a]	Dosage of carboplatin	Dosage of pemetrexed
ANC < 0.5 × 10^9/L and PLT ≥ 50 × 10^9/L	75% of starting dosage	75% of starting dosage
PLT < 50 × 10^9/L, ANC not considered	75% of starting dosage	75% of starting dosage
PLT < 50 × 10^9/L with grade 2 hemorrhage, ANC not considered	50% of starting dosage	50% of starting dosage
ANC < 1 × 10^9/L with fever ≥ 38.5 °C	75% of starting dosage	75% of starting dosage

[a] Nadir of the last cycle.

Dosages shall be permanently reduced at the first onset of neutropenic fever or thrombocytopenia (platelet count < 25 × 10^9/L or < 50 × 10^9/L with signs of bleeding or need for blood transfusion). For the need for dosage reduction at the second onset of neutropenic fever or thrombocytopenia, dosages of carboplatin and pemetrexed shall be reduced in accordance with the physician's judgement and local standard medical practices. In case of neutropenic fever or grade 4 neutropenia, colony-stimulating factors (such as granulocyte colony-stimulating factor) may be administered in place of reduced dosages in accordance with local standard medical practices and ASCO guidelines. For subjects who require a third dose reduction, chemotherapy should be discontinued immediately.

Subjects who require dose modification due to both ANC and PLT shall receive a lower dosage.

Treatments may be postponed for up to 42 days until the first day when neutrophil count is ≥ 1.5 × 10^9/ L and platelet count is ≥ 100 × 10^9/L. However, if the counts fail to recover within 3 weeks, the chemotherapy shall be reduced or suspended in accordance with the physician's judgement and local standard medical practices before neutrophil count recovery.

The investigator should pay attention and remain alert to early and significant signs of myelosuppression, infections and neutropenic fever, to ensure a prompt and appropriate management of such complications. The investigator shall remind the subjects of the signs of such complications and encourage them to seek medical attention as soon as possible.

If chemotherapy is to be suspended due to hematological toxicities, a full blood count (including WBC differential counts) shall be performed once a week until such counts return to the lower limit specified for the treatment. The treatment shall be completed as planned thereafter.

No dose reduction is required for anemia. Subjects shall be supported in accordance with the guidelines of the institution where the attending physician is located.

<u>Non-hematological toxicities</u>

In the event of grade 3 or 4 non-hematological toxicities, treatment shall be delayed until the measurement falls below or is equivalent to the subject's baseline value. At the beginning of the subsequent cycles, the dosage shall be reduced based on the dosage of the last cycle leading to non-hematological toxicities. The following **Table 5**. and **Table 6**. include related recommendations for dose modifications of carboplatin and pemetrexed for non-hematological toxicities.

Table 5 Carboplatin dose modifications based on non-hematological toxicities in previous cycles

**Toxicity**		**Modified carboplatin dosage by % of original scheduled dosage**[a]
Diarrhea	Grade 3 or 4[b]	100%
Oral mucositis	Grade 3 or 4	75%
Nausea/vomiting	Grade 3 or 4	75%
Neurotoxicity (motor or sensory)	Grade 2	100%
	Grade 3 or 4	75%
Transaminitis	Grade 3	75%
	Grade 4	Terminate the treatment
Others	Grade 3 or 4	75%

[a] **Modify carboplatin dosage to a specific percentage of the previous AUC, if deemed appropriate by the attending physician.**
[b] **Or any severity of diarrhea that requires hospitalization.**

Table 6 Pemetrexed dose modifications based on non-hematological toxicities in previous cycles

Toxicity		Modified pemetrexed dosage by % of previous dosage
Any non-hematological toxicity other than mucositis[a]	Grade 3 or 4	75%
Grade 3 or 4 mucositis	Grade 3 or 4	50%
Diarrhea	Grade 3 or 4[b]	75%
Neurotoxicity (motor or sensory)	Grade 1 or 2	100%
	Grade 3 or 4	Terminate the treatment

[a] Excluding neurotoxicity and grade 3 transaminitis
[b] Or any severity of diarrhea that requires hospitalization.

Nausea and/or vomiting shall be treated with appropriate antiemetics. If grade 3 or 4 nausea/ vomiting continues despite the use of antiemetic, reduce subsequent dosage by 25%. If the dose is tolerated, restore the dosage to 100% as soon as possible.

If a subject develops oral mucositis on day 1 of any cycle, suspend the treatment until the oral mucositis subsides. If oral mucositis/stomatitis does not subside within 3 weeks, discontinue carboplatin. For the subject developing grade 3 acute oral mucositis at any time, carboplatin and pemetrexed should be administered at 75% of the dosage after the oral mucositis has completely subsided, which is a permanent dose reduction.

Treatment with carboplatin and pemetrexed (the reason for discontinuation of pemetrexed does not include grade 3 transaminitis) should be discontinued if the subject experiences recurrent grade 3 or 4 hematological or non-hematological toxicity after 2 dose modifications.

3.9.7 Packaging and labelling

HLX10/placebo, HLX04/placebo, carboplatin, and pemetrexed vials shall be labelled by a third party designated by the sponsor. See the drug management manual for drug labels.

3.9.8 Storage, management, and dispensing

The study drugs will be dispensed by the sponsor, or a third party designated by the sponsor. The study site shall establish a comprehensive procedure for study drug reception. A designee is required to receive the study drugs and sign for the receipt. Study drugs are only used in trials specified in this study protocol. Only authorized personnel may have access to these drugs.

The study site shall establish a strict and designated drug management system for the storage and dispensing of study drugs, as well as a registration system. The study site should ensure that the storage conditions of the study drugs are in compliance with the regulations and shall document such conditions and keep the records.

Only the investigator or assigned personnel may administer the medications to the subject and manage such medications. The dispensing and retrieval of every dose shall be documented on a specified log in a timely manner. Any loss, missing, or misuse of study drugs shall be documented in detail.

The study drugs will be recovered and destructed by the sponsor, or a third party designated by the sponsor, and shall not come into the market.

For detailed operations, refer to the drug management manual.

3.9.9 Concomitant and prohibited therapies

The investigator can, at his/her discretion, give all the drugs that he/she deems necessary for the safety of the subjects and expected not to interfere with the assessment of the investigational product (i.e. best supportive therapy). Prophylactic and other supportive treatment for nausea and vomiting may be given to subjects according to local medical practice before and after carboplatin and pemetrexed administration.

All concomitant medications (including start/end dates, total daily dosages, and indications) must be documented in the subject's source document and in the corresponding section of eCRF.

Prohibited medications/ therapies

Medications and therapies which are prohibited during study treatment include:

- Any other therapy with anti-tumor effects, including but not limited to systemic chemotherapy, radiotherapy, hormonal therapy, immunotherapy, biotherapy, molecular targeted therapy, or NMPA-approved anti-tumor modern Chinese medical formulations for marketing (refer to Appendix 7), immunomodulating adjuvants with anti-tumor effects (such as thymosin, lentinan, interleukin-12, etc.); localized treatment of isolated lesions (other than the target lesion) may be accepted (e.g., local surgery or radiotherapy for bone metastases);
- Any other clinical trial treatment, including drugs, instruments or other therapies (such as radiotherapy);
- Immunosuppressants include, but are not limited to, prednisone of over 10 mg/day, or equivalent systemic corticosteroids, methotrexate, azathioprine, and TNF-α blockers, with the exceptions of:
  - ✓ management of study treatment-related AEs with immunosuppressants;
  - ✓ short-term prophylactic use in a subject who is scheduled to receive chemotherapy, when the prescribing information requests for corticosteroids to be administered to patients with known hypersensitivity;
  - ✓ use in subjects who are allergic to contrast agents;
  - ✓ use of inhaled, topical and intranasal corticosteroids;
  - ✓ Short-term use of corticosteroids will be allowed when clinical indications are

present and it is deemed necessary for disease management (e.g., for chronic obstructive pulmonary disease, radiation therapy, nausea).

- Live vaccines within 4 weeks prior to the first study dose and throughout the trial, including but not limited to: measles, mumps, rubella, chickenpox, yellow fever, rabies, Bacillus Calmette-Guérin, and typhoid vaccines. Subjects may receive inactivated viral vaccines by injection for seasonal flu or COVID-19 but may not receive live attenuated vaccines.
- Denosumab. Patients receiving denosumab prior to randomization must be willing and eligible to receive bisphosphonate replacement therapy during the study.

Permitted medications/therapies

Medications and therapies permitted during the study include:

- Treatment for complications, adverse events or symptoms (including blood products, blood transfusions, infusions, antibiotics, anti-diarrheal medications, etc.), with the exception of medications/therapies which are expected to interfere (or interact) with the evaluation of the study;
- Antiemetics;
- Nutritional support;
- Necessary medications or therapies for pre-existing conditions.

3.9.10 Treatment compliance

During the study and follow-up periods, details of study medications should be recorded in the eCRF. Any medication deviating from the protocol should be recorded in the eCRF, including date of and reason for such deviations. The clinical research associate (CRA) shall review the medication compliance during his/her visits to the study site and at the end of the study.

4. STUDY PROCEDURES AND VISITS

4.1. Study Procedures

4.1.1 Demographics and medical history

Demographics contain information on date of birth, gender, ethnicity, etc.

At screening, lung cancer history of each subject must be collected, including clinical phase, pathological diagnosis, diagnosis method, diagnosis date and prior medications (surgical history, radiotherapy/chemotherapy history, etc.). Subjects' personal histories are also collected, including allergy history, drug dependence history, smoking and drinking; in addition, histories of other important diseases within one year prior to signing the ICF must be collected.

4.1.2 Prior and concomitant medications

All previous and concomitant treatments are recorded from 30 days before signing ICF to 90 days after the last study treatment. Concomitant medications associated with AEs are recorded up to 90 days after the last study treatment.

4.1.3 Adverse event

All AEs and treatment emergent AEs are recorded from the time of the ICF signing until 90 days after the last study treatment. If a subject started a new antineoplastic therapy during the AE collection period, only information on AEs related to study treatment is collected after the new antineoplastic therapy.

4.1.4 Quality of life assessment

In this study, subjects will be assessed for quality of life according to EQ-5D-5L, EORTC QLQ-C30 and EORTC QLQ-LC13.

4.1.5 EQ-5D-5L

EQ-5D is a standardized measure of health states developed by the EuroQol group that allows a simple and general rating of health status from clinical and economic perspectives (EuroQol Group 1990). Applicable to a wide range of health conditions and treatments, it provides a simple descriptive profile and a single index value for health status and can be used in the clinical and economic evaluation of health care as well as in population health surveys. The questionnaire assesses five dimensions: mobility, self-care, daily activities, pain or discomfort, anxiety or depression. Each dimension has 5 answer options reflecting the increased difficulty.

Since 2009, the EuroQol group has developed a more sensitive EQ-5D version (EQ-5D-5L), in which the range of answers for each dimension was expanded, namely the severity levels increased from three to five. Preliminary studies have shown that, compared to the nature of measuring parameters in the 3-level version, the 5-level version improved in the following aspects: reduced the ceiling effect, increased robustness, and enhanced ability to distinguish between different health levels.

During the study, subjects are asked to select the most appropriate level in each of the five dimensions described above, indicating their current health state. The questionnaire also includes a visual analogue scale in which subjects will be asked to rate their current health state on a scale from 0 to 100, with 0 indicating the worst health state (see Appendix 3).

4.1.6 EORTC QLQ-C30

EORTC QLQ-C30 Version 3 is an established instrument to assess the health-related quality of life (HRQoL) and is usually used as an endpoint in clinical trials of tumors. The questionnaire

assesses HRQoL/health status through 9 multi-item rating scales: 5 functional rating scales (physical, role, cognitive, emotional, and social), 3 symptom rating scales (fatigue, pain, nausea and vomiting), and 1 global health and QoL (quality of life) rating scale. The 6 individual symptom measures include: dyspnea, sleeplessness, loss of appetite, constipation, diarrhea, and financial difficulties (see Appendix 3). For the 15 domains described above, the total score is standardized to a range from 0 to 100, where higher scores indicate stronger functioning, higher HRQoL or higher symptom levels.

4.1.7 EORTC QLQ-LC13

QLQ-LC13 is a 13-item self-administered questionnaire for lung cancer disease that will be used along with EORTC QLQ-C30. The scale includes both multiple and single lung cancer-related symptom parameters (i.e., cough, hemoptysis, dyspnea, and pain), as well as side effects of conventional chemotherapy and radiotherapy (i.e., alopecia, neurological disorders, oral pain and dysphagia). Similar to EORTC QLQ-C30, all questions (except one question) are on a 4-point scale. The answer option of a question (Question 43: "Have you taken any painkillers?") is "Yes" or "No". QLQ-LC13 is scored similarly to EORTC QLQ-C30.

4.1.8 Echocardiography

Echocardiography must be performed for all subjects at screening, and the results of LVEF are recorded.

During the study treatment, if the subject has clinical symptoms such as shortness of breath, tachycardia, cough, jugular vein distention and hepatomegaly, relevant examinations must be performed timely following the evaluation by the investigator.

4.1.9 12-ECG

Subjects will rest for 5 minutes before each 12-lead ECG. In case of any clinically significant ECG abnormalities at any visits, a re-examination is recommended within 24 hours.

4.1.10 Complete physical examination

At screening, the subjects shall receive a complete physical examination covering his/her head and neck (including thyroid gland), chest (including heart and lung), abdomen (liver, gallbladder, spleen, and kidney), limbs, skin, lymph nodes, nervous system as well as the general conditions, with the examination results recorded; special attention should be paid to the symptoms and signs in respiratory system.

4.1.11 Symptom-directed physical examination

A symptom-oriented physical examination will be performed by the investigator during study treatment based on clinical observations and symptoms. Clinically significant physical examination abnormalities that are judged by the investigator to be significantly worse than the

screening period or newly developed should be recorded as adverse events.

4.1.12 Height, weight and vital signs

Height is measured only at screening. Vital signs should be assessed after the subject has rested for at least 5 minutes, including blood pressure (mmHg), pulse (beats/min), respiratory rate (breaths/min) and body temperature (°C), and body weight should be recorded.

Body weight and vital signs should be measured prior to each dose during study treatment.

4.1.13 ECOG score

Evaluation of ECOG PS by the same investigator is recommended throughout the study. The first ECOG score should be completed within 7 days prior to randomization.

4.1.14 Local laboratory tests

Local laboratory tests performed at the study site include routine blood test, serum chemistry, coagulation, urinalysis, thyroid function, virological test, myocardial enzyme test and pregnancy test. Routine blood test, serum chemistry, coagulation, urinalysis and myocardial enzyme test will be performed within 3 days pre-dose in each cycle; when these laboratory tests scheduled on the same day as the study treatment, the study drugs can be administered only after the test results are obtained. For During carboplatin therapy, routine blood tests should be performed on Day 8 (± 3 days) of each treatment cycle to closely monitor bone marrow suppression. During the treatment period, thyroid function and blood pregnancy (for females of childbearing age only) tests are performed 3 days pre-dose every 2 treatments.

Table 7 Local laboratory tests

Routine blood test	Serum chemistry	Urinalysis[a]	Others
Red blood cells<br>Haemoglobin<br>Platelet<br>White blood cell counts<br>WBC differential counts and percentages<br>• Basophils<br>• Eosinophils<br>• Lymphocytes<br>• Monocytes<br>• Absolute neutrophil count	Urea/urea nitrogen<br>Creatinine<br>Blood glucose<br>Total bilirubin<br>Direct bilirubin<br>Alanine aminotransferase<br>Aspartate aminotransferase<br>Alkaline phosphatase<br>Lactate dehydrogenase<br>Total cholesterol<br>Total protein<br>Albumin<br>Sodium<br>Potassium<br>Magnesium<br>Chlorine<br>Calcium<br>Phosphorus	Urine specific gravity<br>Urine pH<br>Urine protein<br>Urine glucose<br>Urine ketones<br>Urine occult blood<br>Urine white blood cells<br>In case of abnormalities, microscopically examine:<br>White blood cells<br>Red blood cells	**Coagulation function**<br>International normalized ratio<br>Activated partial thromboplastin time<br>**Thyroid function tests**<br>Triiodothyronine<br>Thyroxine<br>Thyroid-stimulating hormone<br>**Virology[b]**<br>Hepatitis B surface antigen Anti-HBs<br>Hepatitis B E antigen<br>Anti-HBe<br>Anti HBc<br>Hepatitis C virus (HCV) antibody<br>HBV-DNA (optional)<br>HCV-RNA (optional)<br>Anti-HIV

			**Myocardial enzymes**[c]<br>Creatine kinase and its isoenzyme<br>Troponin<br>Brain natriuretic peptide
			**Pregnancy test**[d]

a. If a subject has two consecutive 2++ or one ≥ 3+++ urine protein result during the study, a 24-hour urinary protein test should be performed;
b. All subjects are tested for HBsAg, HBsAb, HBeAg, HBeAb, HBcAb or HCV antibody at screening; HBsAg or HBcAb positive subjects should be further tested for HBV DNA titer; and HCV antibody positive subjects should be further tested for HCV RNA. In case of HBV DNA (–) and: 1) HBsAg (+), or/and 2) HBcAb (+) during screening (baseline), anti-HBV antibody and HBV DNA should be tested every 2 cycles during the treatment period. In case of 1) HCV antibody (+) and HCV RNA (–) at baseline, HCV antibody and HCV RNA should be tested every 2 cycles in the treatment period. The investigator will receive antiviral treatment as clinically needed.
c. Troponin/hypersensitive troponin (high-sensitivity troponin), etc. all meet the requirements of troponin detection in the protocol; brain natriuretic peptide/brain natriuretic peptide precursors all meet the requirements of brain natriuretic peptide detection in the protocol.
d. Women of childbearing potential should have a blood pregnancy test within 7 days prior to randomization and must have a negative result for enrollment; this item is also tested within 3 days pre-dose every 2 cycles.

4.1.15 PK and ADA blood sampling

PK and ADA samples of HLX10 or placebo/HLX04 or placebo are collected and sent to the central laboratory for evaluation.

- Blood samples will be collected at following time points: within 7 days pre-dose in Cycle 1; within 3 days pre-dose in Cycles 2, 4, 6, 8 and every 4 cycles thereafter; within 2 hours after the end of HLX10 or placebo/HLX04 or placebo dosing in Cycles 1 and 8 of treatment period (for PK only); at end-of-treatment visit and/or safety follow-up.

Table 8 PK and ADA blood sampling

**Study visits**		**Blood sampling**	**PK**[a]	**ADA**[b]
Treatment period	Cycle 1	7 days pre-dose	X	X
		2 hours post-dose	X	-
	Cycle 2	3 days pre-dose	X	X
	Cycle 4	3 days pre-dose	X	X
	Cycle 6	3 days pre-dose	X	X
	Cycle 8	3 days pre-dose	X	X
		2 hours post-dose	X	-
	Every 4 cycles thereafter	3 days pre-dose	X	X
**End of treatment**		-	X	X
**Safety follow-up**		-	X	X

a. PK samples will be collected within 2 hours after the end of HLX10 or placebo and

HLX04 or placebo dosing in Cycles 1 and 8 of treatment period.

b. **ADA samples will only be collected at pre-dose** and procedures are described in the laboratory manual.

4.1.16 EGFR/ALK/ROS1 DNA mutation status confirmation

The subjects should provide the detection report of EGFR, ALK and ROS1 gene mutation status (the method specified in NCCN guidelines, blood test results are not acceptable). If EGFR sensitive mutation or rearrangement or fusion mutation of anaplastic lymphoma kinase (ALK) or ROS1 gene is known, subjects should be excluded. If the status of EGFR, ALK or ROS1 is unknown, the mutation status of EGFR, ALK and ROS1 genes should be detected during screening.

4.1.17 Radiological examination

CT or MRI should be performed at screening, after the start of study treatment on sites including brain, chest, abdomen, pelvic cavity and any other sites suspected to have tumor lesions, among which brain MRI or CT (preferably MRI) and bone scans are performed during baseline period, and then in treatment period are performed as determined by the investigator according to clinical needs; examination methods at the same site should be consistent as much as possible throughout the study; if there are no contraindications, contrast agent should be used. During the treatment, the subjects who did not have first PD should have an imaging examination every 6 weeks (± 7 days) within 48 weeks and every 12 weeks (± 7 days) after 48 weeks. For patients who continue to receive the dose after PD, they should continue to have an imaging examination every 6 weeks (± 7 days). The investigator and IRRC respectively assess the tumor images according to RECIST v1.1 (the anti-tumor efficacy assessment can be performed by the investigator according to clinical needs), and the investigator should make subsequent treatment judgement according to the results of their own response assessment. If a tumor assessment is performed within 28 days prior to the first dose by the same method and devices in the same hospital, it may serve as the baseline tumor assessment.

At the EOT visit, if tumor imaging has been performed within the last 4 weeks, a re-test is not required. For subjects who discontinued for reasons other than disease progression, radiological assessments are to be continued as scheduled, until disease progression, initiation of new antineoplastic therapy, withdrawal of ICF, death, or end of study, whichever occurs first.

4.1.18 Biomarker test

Subjects in screening stage must provide samples collected at the non-radiotherapy sites at or after the diagnosis of advanced NSCLC. It is recommended to provide tumor samples (paraffin

blocks or unstained sections) treated with formalin fixation and paraffin embedding (FFPE) within 6 months before the first dose for PD-L1 expression level detection in central laboratory and provide relevant pathological reports of the above samples; if the subject does not have archived tumor tissue samples, fresh tumor biopsy should be performed during the screening period to obtain corresponding tumor samples for PD-L1 expression level detection (the number of samples obtained is determined according to biopsy); if the subject agrees, tumor tissue samples and blood samples can be collected for MSI and TMB detection in the central laboratory. If pathological sampling is performed for subjects during the study treatment, it is recommended to collect their tumor samples; and these tumor tissue sections will be used for immunohistochemical analysis to evaluate the expression level of PD-L1 in tumor cells and tumor-infiltrating immune cells and other purposes, and the relationship between MSI, TMB and response. Fresh sample collection, resection, core needle biopsy, and excisional, incisional, punch, or forceps biopsies are all acceptable. Fine-needle aspirations (i.e., samples that lack a complete tissue structure and provide only cell suspension and/or cell smear), brush biopsies, and cell pellet samples from pleural or peritoneal effusions are unacceptable. For detailed requirements for tissue samples, see the Laboratory Operation Manual.

4.2. Screening Period (Day -28 to -1)

The ICF must be signed and dated by the subject or his/her legal representative prior to conducting study-related procedures.

The screening period should not exceed 28 days, starting from the subject signing and dating the informed consent form and ending when the subject is randomized or fails screening. In this study, **re-test is allowed** for ineligible subjects: in case of unqualified laboratory tests, re-test can be performed within the screening time window as decided by the investigator, without giving a new screening number.

Subjects must complete the following study procedures or evaluations during screening:

1) Signing informed consent form
2) Demographics and medical history
3) Prior and concomitant medications
4) Adverse event
5) Quality of life assessment (Day -7 to -1)
6) Echocardiography
7) 12-ECG
8) Complete physical examination

9) Height, weight and vital signs

10) ECOG scores (Day -7 to -1)

11) Local laboratory tests

    Routine blood test, serum chemistry, coagulation, urinalysis, thyroid function, pregnancy test (for females of childbearing age only), virology and myocardial enzyme test. Tests other than virology should be completed within 7 days before randomization.

12) Gene mutation status confirmation

    There is no EGFR sensitive mutation or ALK and ROS1 gene rearrangement.

13) Radiological examination

    The subject has to undergo CT or MRI during screening (at sites including brain, chest, abdomen, pelvic cavity, and any other site suspected of tumor lesion); bone scans are also required for all subjects at screening. If tumor assessment has been performed within 28 days prior to first dose in the same hospital using the same method and machine, then this tumor assessment can be taken as baseline tumor assessment.

14) Biomarker detection: During the screening period, the subjects must provide tumor tissue samples for PD-L1 expression level detection in the central laboratory; if the subject agrees, tumor tissue samples and blood samples can be collected for MSI and TMB detection in the central laboratory.

4.3. Treatment Period

The treatment period begins with the subject's enrollment, and the first dose should be administered within 3 days after randomization. Each subject will receive study treatment once every 3 weeks until loss of clinical benefit, death, unacceptable toxicity, withdrawal of informed consent, or any other cause specified in the protocol (whichever occurs first). See **Figure 3** "Schematic of study treatment" for details.

4.3.1 Treatment visits prior to progressive disease (first PD)

The following study procedures must be completed at each visit during treatment period:

1) Concomitant therapy

2) Adverse event

3) Quality of life assessment: prior to the first dose and every other subsequent dosing cycle (i.e., pre-dose in cycles 1, 3, 5, 7, etc.) until EOT. Re-assessments are not required for subjects who had a quality-of-life assessment on Day -7 to Day -1 of the screening period.

4) 12-ECG

5) Symptom-directed physical examination

6) Weight and vital signs

7) ECOG scores

8) Survival status

Figure 3 Schematic of study treatment

Stage I: a single-arm study (safety run-in phase)

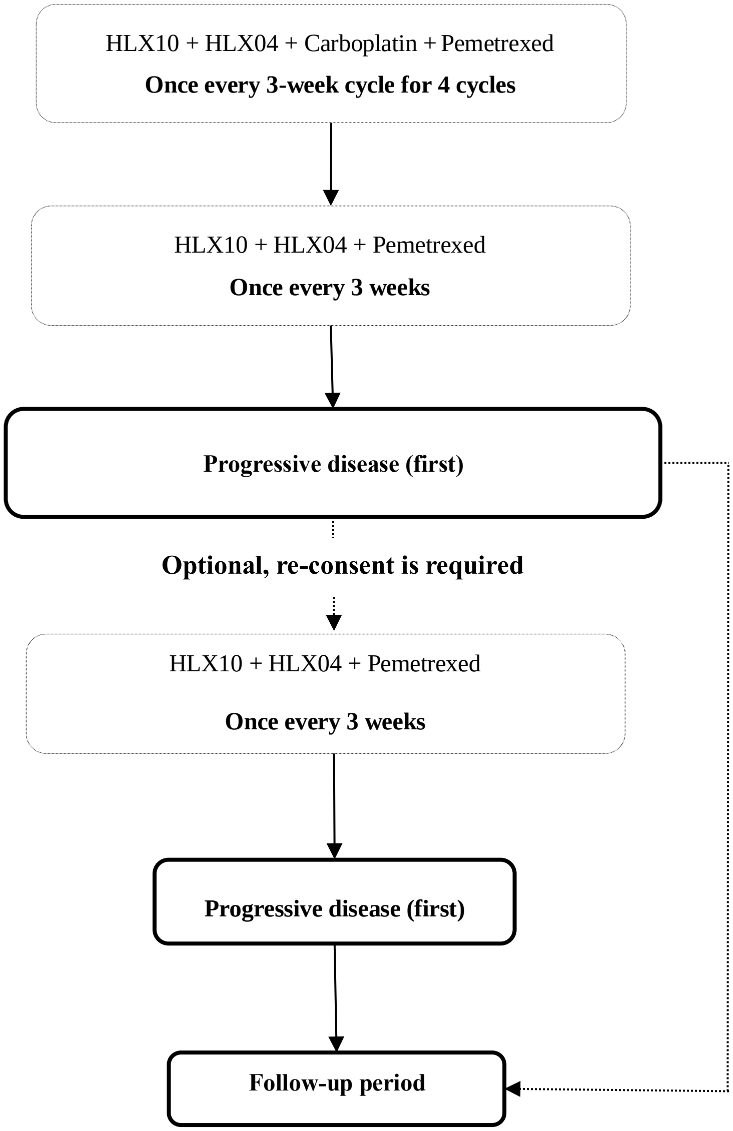

Stage II: phase III study (three-arm, randomized, double-blind)

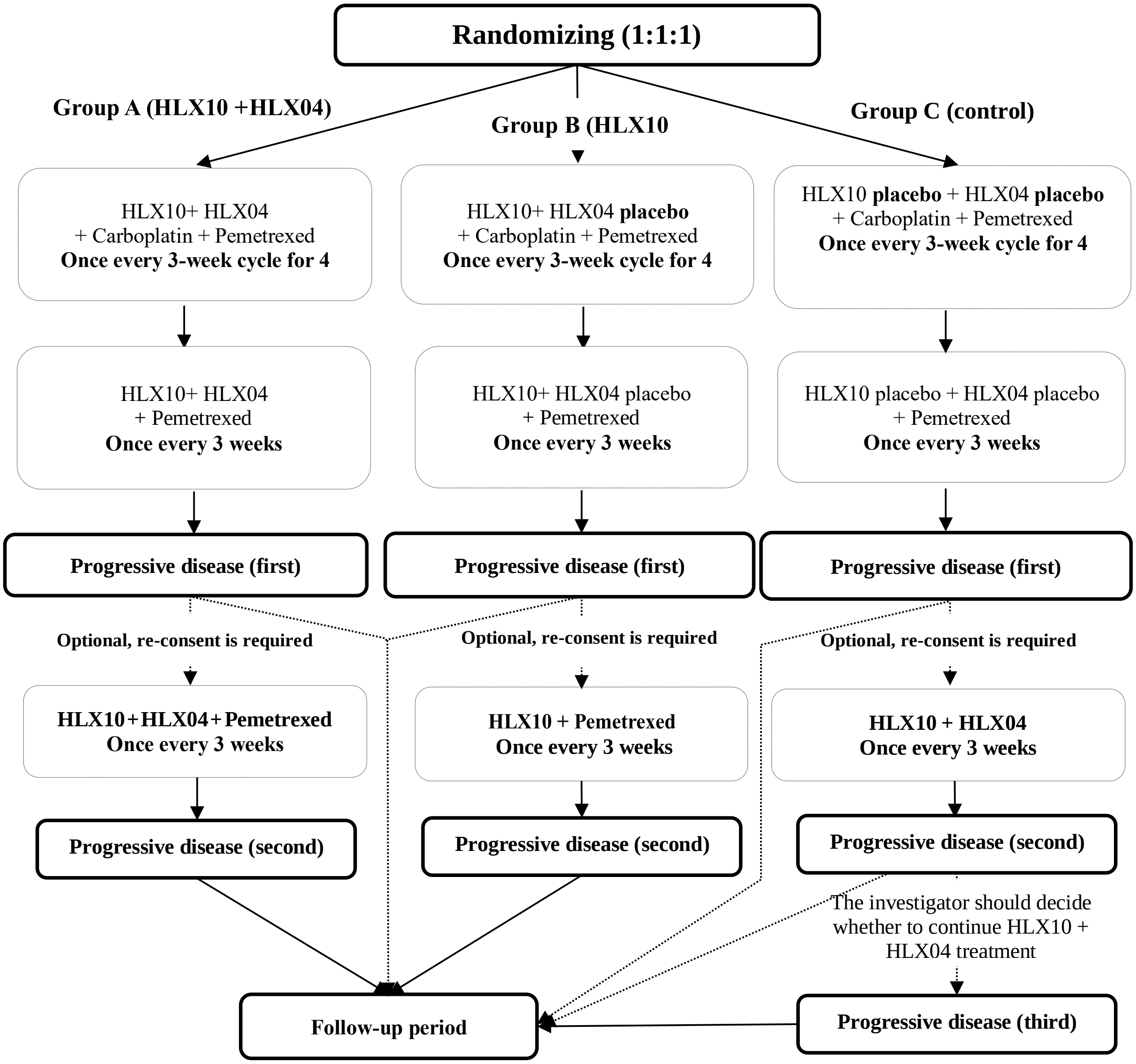

Stage I is the same as stage II. Starting dose:
HLX10 (or placebo), 4.5 mg/kg, once every 3 weeks. The treatment will be continued until loss of clinical benefit, or until the duration of treatment reaches 2 years (up to 35 dosing cycles), and then the investigator will decide whether to continue the treatment. HLX04 (or placebo), 15 mg/kg, once every 3 weeks. The treatment will be continued until loss of clinical benefit, or until the duration of treatment reaches 2 years (up to 35 dosing cycles), and then the investigator will decide whether to continue the treatment. Pemetrexed, 500 mg/m^2, once every 3 weeks. The treatment will be continued until loss of clinical benefit, or until the duration of treatment reaches 2 years (up to 35 dosing cycles), and then the investigator will decide whether to continue the treatment.
Carboplatin: AUC = 5, up to a dose of 800 mg. Once every 3-week cycle for up to 4 cycles.
If a subject decides to continue treatment after PD, then the subject must meet the following criteria:

(1) With no clinical signs and symptoms (including worsening of laboratory findings) indicating a significant disease progression.

(2) A stable Eastern Cooperative Oncology Group (ECOG) performance status score.

9) Local laboratory tests

Tests concerning hematology, serum chemistry, coagulation function, urinalysis and myocardial enzymes must be performed at the local study site within 3 days prior to each dose. Thyroid function has to be tested at the local study site within 3 days prior to every 2 doses. Serum pregnancy test must be performed for women of childbearing potential at the local study site within 3 days pre-dose every 2 cycles during the treatment period.

In case of HBV DNA (–) and: 1) HBsAg (+), and/or 2) HBcAb (+) during screening (at baseline), HBV antibody and HBV DNA should be tested every 2 cycles during the treatment period. In case of 1) HCV antibody (+) and HCV RNA (–) at baseline, HCV antibody and HCV RNA should be tested every 2 cycles in the treatment period. The investigator will receive antiviral treatment as clinically needed.

For aforementioned tests scheduled on the same day as study treatment, the study treatment can be arranged only after the test results are obtained.

10) PK and ADA blood sampling

Blood samples will be collected at following time points: within 7 days pre-dose in Cycle 1; within 3 days pre-dose in Cycles 2, 4, 6, 8 and every 4 cycles thereafter; within 2 hours after the end of HLX10 or placebo/HLX04 or placebo dosing in Cycles 1 and 8 of treatment period (for PK only).

11) study treatment

Study drugs, including HLX10/placebo, HLX04/placebo, carboplatin (up to 4 cycles) and pemetrexed, are administered on Day 1 of each cycle after all clinical and laboratory operations/assessments are completed.

12) Imaging examination

CT or MRI should be performed every 6 weeks (± 7 days) during the first 48 weeks after the start of study treatment, and every 12 weeks (± 7 days) after week 48 on sites including chest, abdomen, pelvic cavity and any other sites suspected to have tumor lesions; examination methods at the same site should be consistent as much as possible throughout the study; if there are no contraindications, contrast agent should be used. The investigator and IRRC respectively assess the tumor images according to RECIST v1.1 (the anti-tumor efficacy assessment can be performed by the investigator according to clinical needs), and the investigator should make subsequent treatment judgement according to the results of their own response assessment.

4.3.2 Treatment visits after progressive disease (first PD)

During the study, if a subject experiences progressive disease (first PD, PD confirmed by IRRC

according to RECIST v1.1 criteria), the investigator will unblind the subject and decide whether to **continue the treatment (the study treatment visits scheduled for subjects in safety run-in period of Stage I is the same as that for subjects in Group A of Stage II), the details are as follows:**

- Group A: After the occurrence of the first PD, **continue the treatment as per the original regimen** for a maximum of 6 weeks and then perform tumor assessment again; if tumor progression occurs again (the second PD), end the study treatment for the subject and proceed to the follow-up period; if no progression occurs, continue the treatment as per the **original regimen**.
- Group B: After the occurrence of the first PD, **continue the treatment as per the original regimen** for a maximum of 6 weeks and then perform tumor assessment again; if tumor progression occurs again (the second PD), end the study treatment for the subject and proceed to the follow-up period; if no progression occurs, continue the treatment as per the **original regimen**.
- Group C: After the occurrence of the first PD, discontinue chemotherapy, and continue the treatment with HLX10 + HLX04. If tumor progression occurs again (the second PD) after continued treatment, the investigator will determine whether to **continue the treatment with HLX10 + HLX04**. If the treatment is continued, perform tumor assessment again after a maximum of 6 weeks; if tumor progression occurs the third time (the third PD), end the study treatment for the subject and proceed to the follow-up period.

If a subject decides to **continue medication** after PD, **the following criteria must be met (applicable to both the first and second stages):**

(1) Absence of clinical symptoms and signs of significant disease progression (including worsening laboratory results).

(2) A stable ECOG PS score.

(3) With no rapid disease progression or tumor progression requiring urgent alternative medical intervention at critical anatomical sites (e.g., spinal cord compression).

(4) The major organ function meets the inclusion and exclusion criteria of this study.

(5) The subject should sign an informed consent form.

Patients who continue treatment after progressive disease should be closely monitored clinically and followed up as soon as possible if symptomatic deterioration occurs. Treatment should be discontinued at any time if clinical worsening due to disease progression is noted or if continuous disease progression is confirmed by follow-up imaging assessments.

Subjects who re-consent after disease progression should complete the disease progression

baseline assessment for continuation of treatment within 28 days after confirmation of disease progression (in case of a need for recovery due to AEs, a maximum of 42 days from the end of previous study treatment is allowed). The imaging test confirming the subject's progressive disease can serve as the baseline for further treatment if the following two conditions are met: (1) an interval of no more than 28 days from the imaging test to the start of further treatment; (2) no study treatment has been done after that imaging test; otherwise, another baseline imaging assessment should be performed before further treatment. Subjects who continue to receive dose after PD should continue to have an imaging examination every 6 weeks (± 7 days).

Treatment visits will be continued every 3 weeks after the start of further treatment, and the visits will be scheduled as those prior to progressive disease (first PD). If the test results (including laboratory procedures and clinical procedures) in the prior 7 days have been obtained at first dose after the disease progresses, the tests may not be repeated. After the disease progresses, the study treatment visit is continued every 3 weeks (± 3 days) with the time of first dose after PD as the baseline, and the collection of blood samples for PK and ADA follow the original protocol. The tumor evaluation after PD is continued every 6 weeks (± 7 days) with the time of first dose after PD as the baseline, and follows the imaging follow-up requirements of the protocol.

4.4. End-of-Treatment Visit

If a subject's study treatment is discontinued for any reason, an end-of-treatment visit should be performed whenever possible. The visit should be completed within 7 days after the subject is informed or confirmed of discontinuation (and should be completed before the subject starts a new anti-tumor therapy). During the visit, the investigator shall collect the following information:

1) Concomitant therapy
2) Adverse event
3) Quality of life assessment

   Quality-of-life assessment: One quality-of-life assessment should be performed at this visit if such assessment has not been done in the past 3 weeks.
4) 12-ECG
5) Symptom-directed physical examination
6) Weight and vital signs
7) ECOG scores
8) Survival status

9) Local laboratory tests

Local laboratory tests: routine blood test, serum chemistry, coagulation, urinalysis, thyroid function test, myocardial enzyme test, pregnancy test (for females of childbearing age only) and virology (if necessary).

Retest may be waived if it is within 3 weeks from the last thyroid function test.

If HBV DNA(–) and 1) HBsAg(+) and/or 2) HBcAb(+) during the screening period (at baseline), and it is more than 3 weeks from the last test, anti-HBV antibody and HBV DNA should be tested at the EOT visit. If 1) anti-HCV antibody(+) and HCV RNA(–) at baseline, and it is more than 3 weeks from the last test, anti-HCV antibody and HCV RNA should be tested at the EOT visit. The investigator will receive antiviral treatment as clinically needed.

10) Central laboratory assessment

Samples of PK and ADA should be collected at termination visit.

11) Tumor imaging assessments

At the EOT visit, if tumor imaging has been performed within the last 4 weeks, a re-test is not required.

4.5. Follow-Up Period

After the EOT visit, subjects will be followed up. If a subject terminates the treatment not because of progressive disease (PD), then radiologic assessment should be further performed according to an established schedule, where possible, until PD, initiation of a new anti-tumor therapy, ICF withdrawal, death or study completion (whichever occurs first).

4.5.1 Safety follow-up period

All subjects are required to visit the study site for safety follow-up 30 days (± 7 days) after the last dose; and if the end-of-treatment visit is delayed for any reasons and occurs after the time window of 30 days (± 7 days), no further safety follow-up visit is required. A follow-up telephone call for safety follow-up 90 days (± 7 days) after the last administration is required. Only the information of AEs and AE-related concomitant drugs is collected. Safety information should be acquired by phone with a window period of ± 7 days. Safety follow-up assessment 30 (± 7) days after the final dose includes:

1) Concomitant therapy

2) Adverse event

3) Quality of life assessment

4) 12-ECG
5) Symptom-directed physical examination
6) Weight and vital signs
7) ECOG scores
8) Documenting subsequent anti-tumor therapies
9) Survival status
10) Local laboratory tests

    Local laboratory tests: routine blood test, serum chemistry, coagulation, urinalysis, thyroid function test, myocardial enzyme test, pregnancy test (for females of childbearing age only) and virological test (if necessary).

    Retest may be waived if it is within 3 weeks from the last thyroid function test.

    If HBV DNA (–) and 1) HBsAg(+) and/or 2) HBcAb(+) during screening period (at baseline), and it is more than 3 weeks from the last test, then HBV antibody assay and HBV DNA assay should be performed at the safety follow-up visit. In case of 1) anti-HCV antibody (+) and HCV RNA (–) at baseline, and it is more than 3 weeks from the last test, anti-HCV antibody and HCV RNA should be tested at the safety follow-up visit.

11) Central laboratory assessment

    Samples of PK and ADA should be collected.

4.5.2 Survival follow-up period

During the survival follow-up period, subjects without PD and not receiving any other anti-tumor therapy should return to the hospital according to the established schedule for radiologic assessment follow-up, until PD, initiation of a new anti-tumor therapy, ICF withdrawal, death or study completion (whichever occurs first); while subjects experiencing PD or undergoing any other anti-tumor therapy just need to be followed up for survival status by telephone call (TC) once every 12 weeks (± 7 days).

The following assessments shall be done at each follow-up visit:

12) Documenting survival status;
13) Documenting subsequent anti-tumor therapies.

4.6. Study Assessments

4.6.1 Efficacy assessment

Except overall survival, other efficacy endpoints are evaluated based on tumor response as per RECIST 1.1. Tumor assessment will be performed by qualified personnel at the site and the IRRC. Treatment decision will be based on tumor response assessed by the investigator and these results will be reported in eCRF.

Tumor assessment schedule is not influenced by treatment interruption or any other event leading to imbalance in disease assessment time between treatment groups.

PFS is defined as the time from randomization to the first documentation of PD or death due to any reason (whichever occurs first). PFS will always be obtained based on scan/assessment date instead of visit date.

Overall survival (OS) is defined as the time from randomization through death due to any reason.

Objective response rate (ORR) is defined as the percentage of subjects whose best overall response is evaluated as CR or PR.

Duration of response (DOR) is defined as the time from the first documentation of response (CR or PR) to the first documentation of PD or death due to any reason(whichever occurs first). Response termination date should be consistent with the date of PD or death due to any reason for evaluation of PFS endpoint as per RECIST 1.1.

4.6.2 Safety assessment

Safety assessment consists of monitoring and documenting all adverse events (including serious adverse events), laboratory tests (routine blood test, serum chemistry, coagulation test, urinalysis, and thyroid function test, etc.), 12-lead ECG, vital signs, and physical examination.

4.7. Adverse Event

4.7.1 Definition of AE

AE is defined as untoward medical occurrence in a patient or a subject of a clinical study administered a pharmaceutical product. AE does not necessarily have to have a causal relationship with the treatment. An AE may be worsening or more frequent occurrence of the pre-existing symptoms, signs, or laboratory abnormalities, a newly diagnosed disease, or abnormal laboratory finding, etc..

AEs include, but are not limited to:

- Abnormal test result;
- Signs and symptoms of clinical significance;
- Physical examination results;

- Hypersensitivity;
- Drug abuse;
- Drug dependence.

In addition, AEs may include signs and symptoms caused by:

- Overdose;
- Drug misuse;
- Drug interaction;
- Dosing error.

Not all vital signs measurement abnormalities or laboratory test abnormalities need to be reported as an AE. A vital signs measurement result or laboratory test result that meets any of the following criteria should be reported as an AE:

- The test result is accompanied by symptom, and (or)
- The test result requires drug/surgery intervention, and (or);
- The test result leads to study dosing adjustments (outside of protocol stipulated dose adjustment) or
- The test result constitutes an AE in the opinion of the investigator or the sponsor.

If none of the above criteria met, merely repeating abnormal examination result does not constitute an adverse event. The test results that the investigator judges to be erroneous do not need to be reported as an adverse event.

The investigators are responsible for reviewing all vital signs and laboratory test results. Medical and scientific assessments should be performed when determining whether a vital sign abnormality alone or a laboratory test abnormality alone is classified as an AE.

4.7.2 Adverse event of special interest (AESI)

Adverse events of special interest (AESI) are investigational product related events attracting scientific and medical interests which may require close monitoring and prompt communication between the investigators and the sponsor. AESIs can be serious or non-serious adverse event. Expedited reporting enables continuous monitoring of AESIs in order to describe and understand their association with use of the investigational product.

AESI in this study includes infusion related reaction (IRR) and other immune-related adverse events (irAE).

Acute infusion reaction (including cytokine release syndrome, angioedema or allergic reaction). Infusion reaction often occurs during or shortly after drug infusion, and generally relieve within

24 h after completion of the infusion. Symptoms and signs of infusion reactions include allergic reaction /hypersensitivity, drug fever, arthralgia, bronchial spasm, cough, vertigo, and dyspnea. Serious allergic reaction may require epinephrine treatment. Infusion reaction should be graded according to NCI CTCAE criteria. Treatment recommendations are as follows:

Table 9 Treatment of infusion reaction

CTCAE Grade	Management	Post-infusion prophylactic drugs
**Grade 1:**<br>Mild reaction, no indications for termination of infusion and no indications for intervention	Strengthening monitoring of vital signs until the subject is considered medically stable by investigators.	No
**Grade 2:**<br>Require infusion interruption but responds promptly to symptomatic therapy (e.g., antihistamines, NSAIDs, narcotics, intravenous fluids). prophylactic medications indicated for ≤ 24 h	**Stop the infusion and monitor the symptoms.**<br>Additional treatments, such as intravenous fluids, antihistamines, acetaminophen, or narcotics.<br>If the symptoms subside within one hour after drug discontinuation, restart the infusion at 50% of the original infusion rate.<br>If symptoms do not subside, continuous monitoring will be required, and the patient can be hospitalized for further treatment.<br>Although adequate prophylactic drugs have been administered, permanent discontinuation of HLX10 infusion should be performed for the patients experiencing grade 2 toxicity.	Prophylactic use of antihistamines (such as diphenhydramine) and acetaminophen or determined according to the clinical practice of the study site.
**Grade 3:**<br>Extended symptoms (which cannot rapidly alleviate even after symptomatic treatment and/or suspension of infusion); symptoms recur after initial alleviation; indications of hospitalization for other clinical sequelae (such as kidney injury, pulmonary infiltration).<br>**Grade 4:**<br>Life-threatening; indications for vasopressors and ventilator support	**Stop infusion**<br>Additional treatment is required: Intravenous fluids, antihistamines, NSAIDs, acetaminophen, anaesthetics, oxygen therapy, vasopressors, glucocorticoids, and epinephrine.<br>Hospitalization for further treatment is required.<br>The patient should immediately stop receiving treatment and permanently stop receiving HLX10.	No subsequent infusion

irAEs are defined as AEs that are associated with drug exposure and demonstrate immune-mediated mechanisms with no other unequivocal etiology. Serological, immunological, and histological (biopsy) data should be used to support the diagnosis of irAEs where appropriate. Appropriate methods should be used to exclude pathological factors of irAE such as tumor, infection, metabolism, toxin, etc. More specific guidance on the assessment and treatment for irAEs is detailed in "Investigator's Brochure".

For any suspected irAE, related system functions need to be closely observed, and adequate assessment shall be carried out to identify the cause and exclude other potential causes. Overall, HLX10 should be suspended or permanently discontinued and/or symptomatic treatment, such as glucocorticoids, should be given depending on the severity of events. If the AE is not improved or is worsened after glucocorticoid therapy, one can consider increasing the dose of glucocorticoid and/or using any other systemic immunosuppressant. When the grade of an AE is < 1, dose of glucocorticoid can be reduced gradually and the treatment has to persist for at least 1 month. When an AE is relieved and less than Grade 1 and the dose of glucocorticoid is reduced to a daily dose of less than 10 mg of prednisone (or other drugs with equal potency), HLX10 infusion may continue. In the event of recurrence of any grade 3 or above irAE (except for endocrine system disorders), permanent discontinuation and study withdrawal have to be done immediately. An irAE should be treated in accordance with the latest version of "NCCN Guidelines for Dose Modification and Treatment for Immune-Related Toxicity" or "CSCO Guidelines for Toxicity Management Related to Immune Checkpoint Inhibitors" and refer to Section 3.9.4 for basic principles and suggestions.

AESI that meets the SAE criteria should be handled in accordance with the relevant procedures of SAE reporting. Any of the following AE should be documented into the "Adverse Event of Special Interest Form" and reported to the sponsor within 24 hours after awareness by the investigator even if it does not meet the SAE criteria (with no need to report to the relevant regulatory ethics department):

- ≥ grade 3 of infusion reactions;
- ≥ grade 2 of colitis, uveitis, interstitial pneumonia, and myocarditis;
- ≥ grade 3 of other immune-related adverse events.

4.7.3 Definition of serious adverse event (SAE)

A serious adverse event (SAE) is defined as any untoward medical occurrence at any dose during a clinical trial and meets any one or more of the following criteria:

1) Results in death
2) Life-threatening (AE occurrence leads to an immediate life-threatening risk to the subject, not including AEs that may lead to death after PD, e.g., drug-induced hepatitis without liver failure)
3) Leading to hospitalization or prolonged hospitalization;
4) Leading to permanent or significant disability/ incapacity
5) Leading to any congenital anomaly/birth defect (of the offspring of the subjects)

6) Other important medical events: Such events may not immediately be life-threatening or result in death or hospitalization but may jeopardize the subject or may require intervention to prevent any one of other outcomes constituting SAEs stated above. Medical and scientific judgment must be made to determine whether such events are reported as an SAE. Important medical events may include allergic bronchospasm necessitating intensive therapy in the emergency department or at home, blood dyscrasias or convulsions not leading to hospitalization, and drug dependence or drug abuse).

Note: Hospitalization or prolonged hospitalization on account of any non due to AE/for convenience purely for the purpose of clinical trial purpose does not meet the criteria for a medical event and thus should not be considered as an SAE.

Hospitalization also includes in-hospital transfers to emergency/ICU ward (such as referral from pediatrics department to internal medicine department, from internal medicine department to coronary care unit, from neurology department to tuberculosis unit).

Hospitalization does not include a stay in any of the following premises:

- Rehabilitation facilities;
- Hospice care centres;
- Short-term care facilities (e.g., nursing assistants);
- Skilled nursing facilities;
- Nursing home;
- Same Day surgery (outpatient/daytime surgery/daytime operation).

Hospitalization or prolonged hospitalization due to any of the following causes is not classified as an SAE:

- Hospitalization or prolonged hospitalization not related to any AE or worsening of any original adverse disease (for example, the workup of persistent laboratory outliers existing prior to treatment);
- Hospitalization for non-medical reasons (e.g. homeless subject or other administrative reasons);
- Hospitalization for administrative reasons (e.g., routine annual checkup);
- Hospitalization stipulated in the study protocol (e.g., procedures required to carry out the study protocol);
- Selective hospitalization not associated with the worsening of AE (e.g. for elective plastic surgery);

- Hospitalization or surgery scheduled by the subject;
- The treatment or surgery scheduled before signing the informed consent should be recorded in the whole trial protocol and/or the baseline data of the individual subject (If a subject develops a discomfort or disease prior to the study enrollment and is planned to be admitted for treatment and/or surgery before or during the study but does not present with exacerbation in an unexpected manner during the study, this event will not be categorized as an SAE
- Hospitalization for non-medical reasons (e.g. lack of housing, financial insufficiency, leave of caregiver, family environment, and administrative reasons).

4.7.4 Liver function test outliers meeting Hy's Law

Any cases which confirmed by re-examination to meet the criteria listed in the table below and have no other reason at the time of abnormal liver function test(LFT) shall be deemed as a potential Hy's Law (HL) case, regardless of whether all the examination results which should determine the cause of abnormal LFT have been obtained or not. An event that meets the criteria in the table below should also be reported as an SAE.

Table 10 Liver function test outliers that should be reported as an SAE

Baseline period	Normal (AST/ALT and total bilirubin)	Abnormal (AST or ALT or total bilirubin)
Treatment period	✧ ALT or AST ≥ 3 × ULN<br>✧ concurrent with total bilirubin ≥ 2 × ULN<br>✧ And alkaline phosphatase ≤ 2 × ULN or unknown<br>✧ And no hemolysis	✧ AST or ALT ≥ 2 × baseline level and ≥ 3 × ULN or AST or ALT ≥ 8 × ULN (whichever is smaller)<br>✧ concurrent with total bilirubin ≥ 2 × ULN and an increase by one upper limit of normal over baseline or or > 3 × ULN (whichever is smaller)<br>✧ And alkaline phosphatase ≤ 2 × ULN or unknown<br>✧ And no of hemolysis

4.7.5 Recording of AE

4.7.5.1 Collection Time of adverse event

AE that occurs from the signing of the informed consent form to 90 days after the last dose of the investigational product (HLX10/placebo or HLX04/placebo) or the initiation of a new anti- tumor treatment (whichever occurs first) will be recorded in the corresponding AE page of EDC. Only SAEs related to the investigational product (HLX10/ placebo or HLX04/ placebo) will be recorded and reported afterwards.

4.7.5.2 Follow-up of unresolved adverse events and causality between the adverse event and investigational product

All AEs and SAEs of each subject should be actively followed up by the investigator throughout the study. Determine the follow-up time and frequency based on the medical conditions, and implement necessary treatment and therapeutic actions during the follow-up to ensure that the

damages to the subjects are minimized, the subject safety is fully guaranteed, and the whole follow-up process and the corresponding treatment results are recorded in detail. If the event is ongoing after end of treatment or study, it should be followed by the investigator as possible until all events meet any of the followings:

1) The event is recovered (or returns to the baseline level);
2) The event is stable (as predicted by the investigator, the AE will not be further improved or exacerbated);
3) Failing to acquire more information (the subject refuses to provide more information, or it is evidenced that the subject is lost to follow-up even after the maximum effort has been made).

The sponsor reserves the right to request additional information (if necessary) about the ongoing AE/SAE from any subject through the investigator at the end of the study.

4.7.5.3 Information collection

For each AE, the following information will be collected:

- AE Term
- Onset date and end date of the AE
- CTCAE grade change
- Whether it is an SAE
- Causality between the AE and the study drugs as assessed by the investigator
- Actions taken on the study drugs
- Treatment measures taken the AE
- Outcome
- irAE or not

In addition, the following information will be collected for each SAE:

- Date when the AE meets SAE criteria
- Date when the investigator be aware of the SAE
- Reason for determining the event as a SAE
- Date of discharge (if applicable)
- Possible cause of death (if applicable)
- Date of death (if applicable)

- Autopsy results (if applicable)
- Assessment of causality between the AE and study procedure
- Assessment of causality between the AE and other drugs
- Description of the AE

Note that when recording the adverse event term, the event term should be recorded in eCRF (and SAE report, wherever is applicable) as the name of the diagnosis rather than a single symptom and sign if the term of the diagnosis is known at the time of reporting (for example, the recording should be "hepatitis", not "increased transaminases/bilirubin" or "jaundice"). However, each sign or symptom should be respectively recorded in eCRF (and SAE report, if applicable) as an AE or SAE if a series of signs and/or symptoms cannot be medically characterized as a single diagnosis and/or syndrome when reporting. If the diagnosis is confirmed later, the individual sign and/or symptom in eCRF and SAE report (wherever is applicable) should be replaced by the diagnosis term of AE or SAE.

A diagnostic or therapeutic invasive (such as surgery) and non-invasive operation should not be reported as an adverse event. But if the operation is resulted from a medical condition that meets the definition of an AE, it should be reported. For example, acute appendicitis that flares during AE reporting should be reported as an AE and the resultant appendectomy should be documented as the treatment for this AE.

In case of a subject who is discontinued from the drug therapy while maintaining participation in the trial for any reason:

- The persistent AEs should be tracked to continue the collection of follow-up information;
- Before the follow-up is concluded, SAEs related to the investigational product/procedure should be continuously collected while following the standard time frame and process of SAE reporting.

4.7.6 Severity evaluation

The investigator shall assess the severity of each AE and SAE reported during the study. In case of an observed AE exceeding the range specified in such criteria, investigators must classify the severity of each type of AE based on their clinical judgment. AEs and SAEs should be assessed and graded based upon US National Cancer Institute (NCI) CTCAE v5.0. The severity definitions in CTCAE v5.0 are as follows:

Grade	CTCAE Description
1	Mild: Asymptomatic or mild symptoms; clinical or diagnostic observations only; intervention not indicated.

**2**	Moderate: Minimal, local or non-invasive intervention indicated; limiting age-appropriate instrumental activities of daily living*.
**3**	Severe or medically significant but not immediately life-threatening; hospitalization or prolongation of hospitalization indicated; disabling; limiting self-care ADL**.
**4**	Life-threatening consequences: Urgent intervention indicated
**5**	AE-related death

CTCAE = Common Terminology Criteria for Adverse Events
*: Instrumental ADL refers to preparing meals, shopping for groceries or clothes, using the telephone, managing money, etc.
**: Self-care ADL refers to bathing, dressing and undressing, feeding self, using the toilet, taking medications, and not bedridden.
Attention must be paid to distinguish severity from intensity of an AE. "Severe" is used to describe intensity, so a severe AE is not definitely an SAE. For example, a headache may be severe in intensity but not necessarily meets the criteria to be listed as SAE unless it meets the SAE criteria.

4.7.7 Causality assessment

The investigator must provide a causality assessment of all AEs (both serious and non-serious). The investigators must record causality, i.e., " related", "possibly related", "unlikely related", "not related", or "unknown". " related", "possibly related", and "unknown" will be considered a causal relationship between the AE and the drug, i.e., adverse drug reactions. When determining the relationship between an AE/SAE and the investigational product, the factors that need to be considered are as follows:

a) Whether the start time of administration and the occurrence time of an adverse event are in chronological order;

b) Whether the suspected AE conforms to a known AE type of the study drugs;

c) Whether the suspected adverse event can be explained by the effects of concomitant administration, clinical status of the patients, or the effects of other therapies.

d) Whether the AE disappeared or relieves after discontinuation or dose reduction;

e) Whether the same reaction occurs after re-dosing.

Table 11 Scale for assessment of causality between AE and investigational product

	**Related**	**Possibly related**	**Unlikely related**	**Not related**	**Unknown**
Reasonable time relation with the investigational product	+	+	-	—	Essential data for assessment is unavailable
Known type of drug reactions	+	± ?	—	—	
Reaction is attenuated or absent after discontinuation	+	± ?	± ?	—	
Reaction reoccurs after repeated medication	+	?	?	—	

There is a probability of any other cause leading to this AE	-	± ?	± ?	+	
Note: + Yes – No ± Likely ? Unknown					

4.7.8 Expectedness Judgement

An unexpected adverse event refers to an event not consistent with the corresponding reference safety information (RSI) of the drug in nature or in terms severity. For IPs, the expectedness of AEs will be determined by the sponsor based on whether it is listed in the Investigator's Brochure.

4.7.9 Disease progression

PD refers to the deterioration of a subject's condition caused by the disease under study. Deterioration of symptoms and/or signs associated with PD should not be recorded as an AE/SAE. Both occurrence of new metastases relative to the primary tumor and progressions of pre-existing metastases are considered to be disease progression. PD leading to hospitalization is not required to be reported as SAE but PD leading to death required.

4.7.10 Newly developed primary tumor

The occurrence of any new tumor should be regarded as an SAE. Newly developed primary tumors refer to tumors found after the subject is enrolled into this study and that are not primarily tumor being treated with the study drugs.

4.7.11 Death

All deaths that occur during the study or during the 90-day safety follow-up following the last dose of the investigational product (HLX10/placebo or HLX04/placebo) as defined in the protocol or before the initiation of a new anti-tumor therapy (whichever occurs first) must be reported as follows:

- In the event of death (including one due to PD), the AE that causes the death should be reported as an SAE to the CRA, sponsor or its representative within 24 hours, and the leading cause of death should be provided.
- If the cause of death is unclear, report the SAE as "Death of Unknown Cause" The cause of death should be further explained during follow-up. An autopsy may help assess the cause of death. If an autopsy is performed, the autopsy report should be submitted to the sponsor's PV team or its representative as soon as possible.

4.7.12 Reporting of AE

The investigator should report all SAEs that occur during the clinical study within 24 hours after awareness to the sponsor or a representative designated by the sponsor. The time frame also applies to additional information (follow-up information) of previously released SAE report, and

initial report and follow-up report of pregnant cases. The sponsor's representative and the investigator share the responsibility of ensuring that all essential information is submitted within the above time frame.

For all SAEs, the investigator is obliged to acquire relevant information and submit it to the sponsor within the above-mentioned time frame of reporting. In addition, the sponsor may request the investigator to acquire more follow-up information quickly. Such information may be more detailed than the information shown in the AE report. In general, such information should include sufficiently detailed description of AEs so as to facilitate comprehensive medical assessment of the cases and independent assessment of causality. Moreover, the investigator must provide information about other potential causes of AEs, such as concomitant medications and complications.

A hard copy of completed and signed Henlius SAE report should be sent by the investigators to the sponsor and the designated representative by fax/mail. In rare cases when no fax device is available, a notification by phone can be accepted, followed by a hard copy of Henlius SAE report sent by mail. After the investigator sends a notification by phone, it is still necessary for the investigator to complete and sign a hard copy of Henlius SAE report in 24 hours after being informed of the event.

The sponsor or its representatives should submit the drug safety report as required by regulatory authorities and regulations.

4.7.13 Overdose

Drug overdose means that a subject receives (intentionally or accidentally) a drug dose in excess of the dose specified in the protocol. In the event of drug overdose, appropriate symptomatic and supportive therapies may be given to the subject. Any overdose-induced adverse reaction should be reported to CRA and included in a standard AE report.

Any drug overdose associate with SAE, it should be reported based on the standard report method and the timeline of the SAE.

4.7.14 Pregnancy

Subjects and women with child-bearing potential shall take appropriate contraceptive measures in a period from signing the ICF through at least 120 days after the final dose of the investigational product and in at least 150 days after the final dose of chemotherapy drug.

During the study, female subjects should stop the study treatment as soon as they become pregnant and inform the investigator. The investigator should report any pregnancy to the sponsor (or an authorized representative) within 24 hours after being informed of the event. The subject will then be monitored until 30 days after the end of pregnancy.

If a female subject or the female partner of a male subject is found pregnant within 6 months after the last dose of the investigational product, it should be immediately reported to the investigator; the investigator should complete the 'Pregnancy Report Form' within 24 hours after awareness of the pregnancy, report it to the sponsor (or authorized representative), and record it in the eCRF for follow-up of the outcome. Any pregnancy event should be followed up until 30 days after the end of pregnancy.

Any AE/SAE occurring in mothers or newborns during pregnancy, such as spontaneous abortion or the termination of pregnancy for medical reasons, birth defects or congenital abnormalities of newborns, malformation and anomalies of stillborn fetuses, and complications of mothers and newborns, should be documented and reported according to "Recording of adverse events" and "Reporting of serious adverse events".

4.8. Sample Size Estimation

Approximately 6–12 subjects are planned to be enrolled in the stage I of this study. Approximately 6 subjects will be enrolled for the first time. After all subjects complete the first cycle of study treatment, the safety and tolerability will be confirmed by the Steering Committee to determine whether to proceed to the second stage of phase III randomized, double-blind, multicenter study. If, in the opinion of the committee, the safety and tolerability cannot be determined, approximately 6 additional subjects will be enrolled for safety and tolerability evaluation.

At stage II, PFS is the primary endpoint, and OS as the key secondary endpoint. The subjects will be randomized in a 1:1:1 ratio, and the sample size is based on the number of events required to demonstrate efficacy with regard to both PFS and OS.

Assuming that the median PFS in the Group C is 6 months, and the hazard ratio (HR) of 0.69 in Group B, a total enrollment period of 24 months, the overall study period of 30 months, and the type I error rate $\alpha = 0.05$ (two-sided), at least 264 PFS events must be observed to obtain 85% power. Given a drop-out rate of 15%, a total of 400 subjects should be enrolled in the 2 groups (200 in each group).

A fixed-sequence testing method will be used to address the multiplicity of multiple group comparisons. If Group B is statistically different from Group C and the HR is < 1, the comparison between the Group A and the Group B will be continued; otherwise, the comparison will not be performed. Assuming that the median PFS in Group B is 8.7 months, the HR of 0.67 in Group A, and other parameters are the same as above, a total of 404 subjects should be enrolled in the 2 groups (202 in each group). In summary, about 606 subjects should be enrolled and at least 396 PFS events should be observed in Stage II.

For the key secondary endpoint OS, assuming that the median OS in the Group C is 10.7 months, and the HR of 0.7 in Group B is, the Lan-DeMets approximation to the O'Brien-Fleming boundary will be used to control the overall type I error rate $\alpha = 0.05$ (two-sided). Assuming that a total enrollment period of 24 months and the overall study period of 46 months, at least 288 OS events must be observed to obtain 85% power.

Assuming that the median OS in Group B is 15.2 months, the HR of 0.68 in Group A, and other parameters and calculation methods are the same as above, and taking into account the potential dropout and that the number of events required and the number of subjects enrolled in Group A are the same as those in other treatment groups, about 630 subjects need to be enrolled and at least 432 OS events need to be observed in Stage II.

Considering the sample size required for PFS and OS evaluation, a total of 630 subjects(210 in each group) will be enrolled at stage II.

4.9. Statistical Analysis Sets

4.9.1 Intent-to-treat (ITT) set

All subjects randomized into the study and will be the primary analysis set for the efficacy analysis in this study. ITT set will be analyzed based on randomized groups.

4.9.2 Per protocol set (PPS)

As a subset of the ITT set, the per protocol set (PPS) consists of all randomized subjects who have received at least one post-treatment tumor assessment without any major protocol deviation that can significantly affect the primary efficacy. The analysis based on the PPS will serve as a support of ITT analyses.

4.9.3 Safety set (SS)

All subjects who have received at least one dose of investigational product. The safety population will be the primary analysis population for safety assessment, and will be analyzed based on actual treatment groups.

4.9.4 Pharmacokinetic set (PKS)

All subjects who have received at least one dose of HLX10 and have at least one post-dose concentration measurement at scheduled PK time points, without any major protocol deviations that can obviously affect the PK assessment. PK set will be used for PK analysis.

4.10. Interim Analysis

An Independent Data Monitoring Committee (IDMC) will be established in this study for interim analysis. At stage II, PFS is the primary endpoint, and OS as the key secondary endpoint. PFS will be analyzed twice, including a blinded sample size re-estimation and a final analysis; OS will be analyzed for four times, including a blinded sample size re-estimation, the first interim efficacy analysis during the final analysis of PFS when reaching approximately 33% of the expected number of OS events, the second interim analysis when reaching approximately 66% of the expected number of OS events, and the final analysis when reaching the expected number of OS events. The overall type I error rate will be controlled by the Lan-DeMets approximation to the O’Brien-Fleming boundary.

The first interim analysis is planned to be conducted when about 420 subjects are enrolled (accounting for about 2/3 of the planned number of enrolled subjects). The primary objective is to perform a blinded sample size re-estimation according to the actual PFS and OS data. This interim analysis will be performed in a blind state, without consuming the significance level α.

Final analysis of PFS is planned to be conducted when the target number (about 396 PFS events) is observed. The significance level for the final analysis of PFS of Group B versus Group C is 0.05 (two-sided).

PFS analysis will be tested in the following sequence:

1. HLX10 in combination with chemotherapy (Group B) versus control group (Group C)
2. HLX10 + HLX04 in combination with chemotherapy (Group A) versus HLX10 in combination with chemotherapy (Group B)

The first interim analysis of OS is planned to be performed with the final analysis of PFS, when the number of OS events is approximately 144 (33% of the total number of events). Based on the O'Brien-Fleming type α-spending function, the significance level for the analysis is 0.0002 (two-sided).

The second interim analysis of OS is planned to include approximately 288 OS events (66% of the total number of events). Based on the O'Brien-Fleming type α-spending function, the significance level for the analysis is 0.012 (two-sided).

The final analysis of OS is planned to be performed when 432 OS events are collected. Based on the O'Brien-Fleming type α-spending function, the significance level for the final analysis is 0.046 (two-sided).If any previous interim analysis in the sequence is not statistically significant, the alpha controlled at 0.05 (two-sided) will not be passed on to subsequent analyses.

Based on the actual number of OS events reached at the interim analysis time points, the significance level for treatment group comparison will be adjusted using the Lan-DeMets approximation to the O’Brien-Fleming boundary, the overall two-sided type I error rate will be maintained at 0.05, and the final analysis will be carried out after reaching the target number of OS events. If the result of the interim analysis is positive, the sponsor may prematurely unblind and end the trial based on the IDMC's recommendation.

4.11. Statistical Analysis Methods

SAS9.2 (or above versions) statistical analysis software will be used for all statistical analyses. Continuous variables, including number of subjects, mean, standard deviation, median, maximum, and minimum, are summarized using descriptive statistics. Categorical variables, including number of subjects, percentage and/or number of events, are summarized using descriptive statistics.

The detailed statistical analysis plan and methodology shall be elaborated in the Statistical Analysis Plan (SAP).

4.11.1 Demographics, medical history, and baseline characteristics

Demographics, baseline characteristics, medical history, and concomitant medications of all randomized subjects will be summarized according to randomization using descriptive statistics.

4.11.2 Medication compliance

The medication compliance of the study drugs will be summarized using descriptive statistics by study groups.

4.11.3 Efficacy analysis

4.11.4 Stage I: safety run-in phase

Efficacy endpoints for each subject will be listed.

4.11.5 Stage II: phase III study

Analysis of primary efficacy endpoint:

Progression-free survival (PFS, assessed by IRRC according to RECIST v1.1): Defined as the time from randomization to the first documentation PD or death due to any reason (whichever occurs first). For subjects with neither PD nor death will be censored on the day of the last evaluable tumor assessment. For subjects who have no tumor assessment during the study and alive, PFS will be censored on the day of randomization. For subjects who have no PD reported and initiate any other anti-tumor therapy that not specified in the protocol, PFS will be censored on the day of the last evaluable tumor assessment prior to the initiation of subsequent anti-tumor treatment. Comparison of PFS between the two groups (Group B versus Group C) will be made by hierarchical log-rank test, and the stratification factors are: PD-L1 expression level (negative, positive and not evaluable), smoking history (yes versus no), brain metastasis (yes versus no). HR and its 95% confidence interval are estimated by stratified COX proportional hazards model; the median and its 95% confidence interval are estimated by Kaplan Meier method, and Kaplan-Meier curve will be ploted.

Analysis of secondary efficacy endpoints

Overall survival (OS): Defined as the time from randomization to death due to any reason. For subjects without death record, OS will be censored on the last known survival date. For subjects not providing any follow-up information, OS will be censored on the day of randomization. Overall survival (OS) is the key secondary endpoint in the stage II, and the statistical analysis method is the same as the primary efficacy endpoint. In the OS efficacy interim analysis, the

significance level α will be adjusted according to the actual number of OS events reached at the analysis time point, and the final analysis will be carried out after reaching the target number of OS events.

PFS assessed by the investigator as per RECIST v1.1 will be statistically analyzed using the same method as that for primary efficacy endpoint.

Objective response rate (ORR) is assessed by the IRRC and the investigator as per RECIST v1.1 respectively. ORR is defined as the percentage of subjects whose best overall response is complete response (CR) or partial response (PR). The stratified CMH method is used to estimate the odds ratio and its 95% CI. Stratification factors include PD-L1 expression level (negative, positive, or not evaluable), smoking history (yes versus no), and brain metastasis (yes versus no).

Duration of response (DOR) is assessed by the IRRC and the investigator as per RECIST v1.1 respectively. DOR is defined as the time from the first documentation of response (CR or PR) to the first documentation of PD or death due to any reasons(whichever occurs first). The DOR will be analyzed only for subjects whose best overall response is evaluated as CR or PR. For subject not experiencing PD or death after achieving response, DOR will be censored on the day of the last evaluable tumor assessment; if no tumor assessment is performed after response achievement, then DOR will be censored on the day of tumor assessment when response is achieved. The median and its 95% confidence interval (CI) will be estimated using the Kaplan-Meier method, and the Kaplan-Meier curve will be plotted.

Treatment comparisons will be conducted sequentially by first comparing Group B versus Group C and then comparing Group A versus Group B. The efficacy endpoints PFS, OS, ORR, and DOR between the two groups (Group A versus Group B) will be analyzed in the same manner as the comparison between Group B and Group C.

4.11.6 Safety Analysis

AEs will be coded according to MedDRA and graded per CTCAE v5.0. Treatment emergent adverse event will be summarized by CTCAE grades. TEAEs and concomitant medications in the trial will be summarized separately by treatment groups. The clinical laboratory parameters, ECOG scores, vital signs, physical examination, and 12-lead ECG will be summarized by the treatment group and study visit. Analysis will describe and present the observed values and changes from baseline by visit in the study. After all subjects have completed the first cycle of study treatment in stage I, a preliminary safety and tolerability analysis will be performed, which will be confirmed by SC, and then the stage II of the study will be initiated.

4.11.7 Pharmacokinetics and immunogenicity analysis

Descriptive statistics of serum concentrations and PK parameters at various visit will be

provided.

ADA-positive rates at various visit will be summarized.

The statistical method will be detailed in the SAP.

If applicable, relations between exposure of HLX10/HLX04 and safety, immunogenicity, and/or efficacy data will be evaluated separately. If this analysis is done, then a separate analysis plan will be written, and the results will be reported separately (not provided in the Clinical Study Report).

4.11.8 Biomarker analysis

During the screening period in this study, tumor tissues of subjects will be collected for assays of PD-L1 expression level, MSI, and TMB; and blood samples of subjects will be collected for assays of MSI and TMB. The primary objective is to assess the relationships between PD-L1 expression and MSI, TMB and efficacy.

4.11.9 Analysis of patient-reported outcomes

The health status and self-perceived health of each patient will be documented in the form of EQ-5D-5L scale, EORTC QLQ-C30 scale, and EORTC QLQ-LC13 scale.

The observed values of total score, sub-score, and individual score at each visit and the changes from the baseline will be described statistically using scale corresponding score summary rules based on the treatment groups. The analytical methods will be detailed in the SAP. All PRO analyses will be performed based on the intention to treat set unless otherwise spefified.

5. ETHICS

5.1. Ethical Requirements

This trial shall be implemented in accordance with the GCP, "Declaration of Helsinki", relevant regulations, and review comments of the IRB.

The investigator shall ensure that this trial is reviewed and approved by a qualified IRB in compliance with GCP. Prior to the trial, the investigator shall submit the trial protocol, ICFs, and other essential documents to the IRB for review and approval. The sponsor can provide the study drugs only after receiving approval from the EC. Meanwhile, the IRB shall be informed of SAEs that occur during the study and subsequent protocol amendments that may impact the safety of subjects and their participation in the study. The investigator is obligated to report the trial progress to the IRB. In addition, the investigator must promptly submit copies of all communications with the EC to the sponsor. When reviewing and approving the trial protocol, the IRB must verify the protocol title and number, indicate and date the reviewed protocol documents. In the event of any additional amendment to the trial protocol and the ICF during the

trial, an additional written approval shall be obtained from relevant authorities according to applicable regulations.

5.2. Informed Consent

The investigator must inform the subjects of the information about this trial in both oral and written manners. Subjects have the right to know the detailed information on the trial.

The ICF (along with the trial protocol) must be reviewed and approved by the IRB. If necessary, the investigator is obligated to explain the content of the ICF to subjects in a manner and wording understandable to the subject. The subject shall have enough time to read the ICF before signing it.

The final text of the ICF shall contain: the trial objective, processes and time frame of the trial, testing procedures, expected benefits and potential risks on the subjects, informing the subjects of being probably assigned to any groups in the trial; treatment and corresponding compensation received by the subjects in the event of any trial-related injury; principle of confidentiality for the personal data of the subjects, etc.

The ICF must be signed and dated by the subject, and the investigator who implements the informed consent shall also sign and date the ICF. The ICF shall be made in duplicates, with the investigator and the subject each holding one copy. Should any important new information related to the investigational product be found, the ICF has to be revised in writing and submitted to the IRB for approval before being approved by the subject again.

5.3. Subject Confidentiality

The investigator is obligated to keep the subjects anonymous. In the CRF or other documents, the subjects can be only identified with capitalized letters, numbers and/or codes, instead of names. The investigator must properly keep the Subject Enrollment Log documenting the codes, names, and residential addresses of the subjects. The investigator must keep strictly confidential any document that may reveal the identity of the subject.

6. DATA MANAGEMENT

6.1. Database Setup

Data of this clinical trial will be collected through remote data entry in the eCRF. The data manager shall design a database and test it with simulated data or real eCRF data to ensure that the database works accurately and correctly.

6.2. Data Check

During data management, data checks include the data logic check, manual check, medical check, and statistical pre-analysis check. All the data queries will be displayed in the EDC

system in the form of electronic queries for the study site to answer. A query will be closed if its answer is acceptable. If any data query is not resolved or a new query arises after the database is updated based on the previous answer, then the investigator or CRC shall answer the query again. The above process will be repeated until all data in the database is checked to be correct.

6.3. Database Lock

The data manager shall draft a data review report pursuant to the trial protocol, data review criteria, and the database. The project manager should convene a data review meeting attended by sponsor, principal investigator, statistician, and data manager to review the data, and a data review resolution should be jointly signed by the representatives of the participants. The data manager shall implement data locking upon approval by all the participants. The locked data will be submitted to the statistician for statistical analysis.

The data can be locked when the following conditions are satisfied.

1) All data has been collected and entered into the database.
2) All codes have been checked and verified.
3) All data queries have been resolved (including queries proposed in data review).
4) Data review has been completed.
5) Source data verification has been completed.
6) Verification of SAEs has been completed.
7) Signatures of all investigators have been obtained.
8) Analyzable cases have been defined and stored in the final analysis database.
9) The SAP has been signed.

7. STUDY MANAGEMENT

7.1. Quality Control and Quality Assurance

Prior to the formal initiation of the trial, the sponsor (or CRO authorized by the sponsor) and the investigator shall discuss and develop a clinical study plan to guarantee the trial quality. The study personnel involved in this trial shall be trained on GCP.

Study drugs must be managed in each study site as per relevant SOPs, involving the receipt, storage, dispensing, and return of the drugs.

In accordance with the GCP guideline, necessary steps must be taken during the design and implementation of this study to ensure the accuracy, consistency, integrity, and credibility of the collected data. All observations and outliers in the clinical trial shall be promptly and carefully

verified and recorded to ensure data reliability. All instruments, devices, reagents, and reference standards used in this clinical trial must be ensured to be in strict compliance with corresponding quality specifications and operated in normal state.

The investigator will enter information required by the protocol into the Case Report Form, and the monitor shall check if it is completely and accurately filled out and instruct the personnel in the study site to make amendments and supplements when necessary.

The drug regulatory authorities and the sponsor may entrust the auditor to perform systematic audit for study-related activities and documents to evaluate whether the study is performed in accordance with the protocol, SOPs, and applicable laws and regulations, as well as whether the study data is recorded timely, truthfully, accurately, and completely. The audit shall be performed by personnel not directly involved in this clinical trial.

7.1.1 Training

According to GCP guidelines, the CRA should have qualifications recognized by the sponsor. Prior to initiation of the clinical study, the investigator should be trained by the study site director to familiarize and understand the protocol, comprehend GCP guidelines, use consistent recording methods and interpretation criteria, and to strictly carry out the study as specified in the protocol.

7.1.2 Clinical monitoring

The CRA acts as a primary liaison between sponsor and investigator. The CRA will fulfil all monitoring responsibilities to monitor this clinical study in accordance with GCP. The CRA will establish and maintain regular contact between the investigator and the sponsor.

The CRA will conduct regular clinical monitoring according to all relevant regulatory requirements and standards, or visit the study site depending on actual situation, supervise the progress of the clinical trial, check and verify if all data records, reports, and eCRF entries are correct, complete, and consistent with the source data, and ensure that the clinical trial is performed according to the clinical trial protocol; the investigator shall actively assist the CRA in these processes.

7.1.3 Audit

During the study, the sponsor may conduct a QA audit for the study site, the study database, and the study documentation. The audit includes drug supply, required trial documents, documentation of the informed consent process, and consistency between the CRF and source documents. The content and scope of the audit can be expanded when needed. Upon reasonable notice, the investigator shall allow study-related audits by the auditor commissioned by the sponsor and inspections from the regulatory authorities.

In addition, the NMPA may inspect the study as well.

7.1.4 Data Management/Coding

Electronic Data Capture (EDC) system will be used in this study, indicating that all eCRF data will be entered in the electronic format at the study site.

The departments of data management and biostatistics will process data generated in this clinical study according to relevant SOPs.

Data acquisition is performed by the personnel designated and authorized by the investigator at the study site. Before the study is initiated and any data of any subject in the study is entered into the EDC system, the investigator and all the authorized personnel at the study site must be properly trained, and appropriate safety measures shall be taken.

The CRA will compare the eCRFs with source documents to ensure that there is no deviation in critical data. All items, corrections, and changes have to be completed by the investigator or the personnel designated by the investigator. Queries will be raised by relevant personnel and sent to the investigator. In this regard, the EDC system will have audit trails, which means the names of the study personnel, as well as the time and date will be documented.

The investigator is responsible for maintaining source documents. These documents shall be checked by the CRA during each monitoring visit. The investigator must submit one copy of the complete eCRF including data of all subjects receiving study medication, regardless of the treatment duration. The study and subject numbers shall be used to indicate explicitly all supporting documents submitted along with the eCRF, e.g., laboratory or hospital records. Any personal information including subject names shall be deleted or made illegible so as to keep the subject information confidential.

7.1.5 Missing and useless data

The handling of missing and useless data is detailed in the SAP.

7.2. Documentation and Retention of Study Data

The investigator is obligated to maintain essential documents of the study (the protocol and protocol amendment, completed eCRFs, signed ICFs, important correspondence files, and all other supporting documents). The study site shall establish a plan to store these documents for at least 5 years after the investigational product is approved for marketing. The study site shall store these documents until at least 5 years after the investigational product is finally approved for marketing, and until at least 5 years after there is no pending approval or marketing authorization application for the investigational product, or after clinical development of this investigational product is formally terminated. These documents shall be retained for a longer time upon the request of any corresponding regulatory authority, or any hospital, institution, or private clinic involved in this study. Subject codes (subject names and corresponding study

numbers) should also be retained for the same time period. As agreed by the sponsor, these documents can be transferred to another responsible party who must observe the document retention policy. The sponsor must be notified in writing of any transfer of documents. The investigator must contact the sponsor before disposing any study records.

7.3. Follow-ups and Medical Measures After Study Completion

AEs/SAEs (including laboratory abnormalities) unresolved at the end of the study or when a subject withdraws from the study have to be followed up.

After the end of study treatment, the investigator should offer necessary and reasonable medical interventions to the subjects to protect their safety, rights, and interests.

8. RESPONSIBILITIES

8.1. Responsibilities of the Investigator

The investigator's responsibilities mainly include but are not limited to:

1) The investigator shall hold a joint discussion with the sponsor to finalize and sign the protocol, which will be implemented after approval by the EC.
2) Scrutinize, understand, and strictly implement the protocol.
3) Be knowledgeable about and familiar with the nature, function, efficacy, and safety of the investigational product (including information about the preclinical study of the product), as well as all new information related to the product discovered during the clinical trial.
4) Conduct the clinical trial in medical institutions equipped with adequate medical facilities, laboratory equipment, and staff, as well as all facilities needed to handle emergencies, so as to ensure the safety of the subjects. Ensure the accuracy and reliability of laboratory results.
5) Obtain the consent of the medical institution or the competent authority, and ensure that sufficient time is left to complete the clinical trial within the time limit set by the protocol. Explain the data, regulations, and responsibilities concerning the trial to the staff participating in the clinical trial to ensure that a sufficient number of subjects meeting the criteria of the protocol are enrolled in the clinical trial.
6) Explain to the subjects the details of the trial approved by the IRB and obtain ICFs from the subjects.
7) The investigator will be responsible for making clinical study-related medical decisions to ensure that subjects will receive proper treatment if AEs occur during the study.
8) Take necessary measures to ensure the safety of the subjects and record such measures. If SAEs occur during the clinical study, the investigator should immediately give appropriate

treatment to the subject, and at the same time report to the drug regulatory authority, health administrative department, sponsor, and EC with signature and date on the report.

9) Ensure that data is recorded in the case history and the CRF in a true, accurate, complete, timely, and legitimate manner.

10) The investigator should accept the monitoring and auditing by the CRA or auditor designated by the sponsor, as well as the auditing and inspection by drug regulatory authority to ensure the quality of the clinical study.

11) Agreements will be made between the investigator and sponsor for the cost of the clinical study, and which should be written in the contract. Refrain from charging the subjects for the cost of the study drug during the clinical trial.

12) After completion of the clinical study, the investigator must write a summary report with signature and date, and send it to the sponsor.

8.2. Responsibilities of the Sponsor

Sponsor's responsibilities mainly include but are not limited to:

1) The sponsor should obtain approval from NMPA.

2) The sponsor is responsible for initiating and applying for a clinical study, and providing study funds.

3) The sponsor should provide investigator's brochure, which shall include the chemical, pharmaceutical, toxicological, pharmacological, and clinical (including previous and ongoing studies) information and data of the investigational product.

4) The clinical protocol should be designed jointly by the sponsor and investigator. Sign the trial protocol and contract agreed upon by both parties.

5) The sponsor should provide investigational product and control drug that are identified easily, encoded correctly and labelled with special label for the investigator, and ensure that the quality is qualified. Package and store the study drugs properly as required by the trial protocol. The sponsor should establish management and documentation systems for the study drugs.

6) The sponsor should appoint qualified CRAs who are accepted by the investigator.

7) The sponsor should establish quality control and quality assurance systems for the clinical study, and organize audit of the clinical study to ensure quality.

8) The sponsor should promptly scrutinize the SAEs with the investigator, take necessary actions to ensure the safety, rights, and interests of the subjects, and report in a timely manner to the drug regulatory authority and health administrative department.

9) The sponsor is responsible for submitting the study summary report to the NMPA.

10) The sponsor should purchase the drug clinical study liability insurance for this study. The sponsor should provide comprehensive medical coverage for the subjects participating in the clinical study. For subjects who suffer study-related damage or death, the sponsor is responsible for affording the cost of treatment and providing appropriate financial compensation. The sponsor should provide the investigator with legal and economic guarantees, except for those caused by medical malpractice.

9. CONFIDENTIALITY AND PUBLICATION OF TRIAL RESULTS

All information related to this study (not limited to the study protocol and the Investigator's Brochure) have to be kept in strict confidentiality. The investigator must recognize that the scientific or medical information derived from this study may be of commercial value to the sponsor. The investigator should keep the information and data related to this study confidential. If the investigator intends to publicly publish information related to this study or the conclusions drawn from this study, he/she shall consult with the sponsor in advance and obtain the written consent of the sponsor. The sponsor may require the investigator not to publish information about the study before the investigational product is approved for marketing, in order to protect the sponsor's rights and interests.

The sponsor has the right to issue or publish information or data related to this study, or submit them to the drug regulatory authority. If the sponsor needs to display the name of the investigator in the content published, released, or advertised, the prior consent from the investigator must be obtained.

References

[1] National Cancer Centre: 2018 National Cancer Report

[2] Ning Ruiling. Clinical observation of comparing pemetrexed with docetaxel as second-line treatment of non-small cell lung cancer. [J]. Chongqing Medical Journal, 2013, 42 (3): 292.

[3] Sun Yan, Shi Yuankai. Handbook of Clinical Oncology [m]. Version 5. Beijing: People's Medical Publishing House, 2007: 133–156

[4] D’Addario G, Fruh M, Reck M, Baumann P, Klepetko W, Felip E, et al.Metastatic non-small- cell lung cancer:ESMO clinical practice guidelines for diagnosis, treatment and follow-up.Ann Oncol 2010;21(Suppl 5):116-9.

[5] Sharma P,Wagner K,Wolchok JD,et a1.Novel cancer immunotherapy agents with survival benefit: recent successes and next steps [J] .Nat Rev Cancer,2011,11(11) : 805-812.

[6] Ishida Y, Agata Y, Shibahara K, et al. Induced expression of PD-1, a novel member of the immunoglobulin gene superfamily, upon programmed cell death. [J]. Embo Journal, 1992, 11 (11): 3887-3895.

[7] Stadler S, Weina K, Gebhardt C, et al. New therapeutic options for advanced non-resectable malignant melanoma. [J]. Adv Med Sci, 2015, 60 (1): 83-88.

[8] Basso D, Fogar P, Falconi M, et al. Pancreatic tumors and immature immunosuppressive myeloid cells in blood and spleen: role of inhibitorv co-stimulatorv molecules PDLl and CTLA4. An in vivo and in vitro study. [J]. PLOSONE, 2013, 8 (1): e54824.

[9] Chen S, Liu H, Su N, et al. Myeloid-derived suppressor cells promote age-related increase of lung cancer growth via B7-H1. [J]. Experimental Gerontology, 2015, 61:84-91.

[10] Nishizumi H . Development of lupus-like autoimmune diseases by disruption of the PD-1 gene encoding an ITIM motif-carrying immunoreceptor. [J]. Immunity, 1999, 11 (2): 141-151.

[11] Okazaki T, Honjo T. PD-1 and PD-1 ligands: from discovery to clinical application. [J]. International Immunology, 2007, 19 (7): 813-824.

[12] Nishizumi H . Development of lupus-like autoimmune diseases by disruption of the PD-1 gene encoding an ITIM motif-carrying immunoreceptor. [J]. Immunity, 1999, 11 (2): 141-151.

[13] Okazaki T, Honjo T. PD-1 and PD-1 ligands: from discovery to clinical application. [J]. International Immunology, 2007, 19 (7): 813-824.

[14] Langer CJ, Gadgeel SM, Borghaei H, Papadimitrakopoulou VA, Patnaik A, Powell SF, Gentzler RD, Martins RG, Stevenson JP, Jalal SI, Panwalkar A, Yang JC, Gubens M,

Sequist LV, Awad MM, Fiore J, Ge Y, Raftopoulos H, Gandhi L; KEYNOTE-021 investigators. Carboplatin and pemetrexed with or without pembrolizumab for advanced, non-squamous non-small-cell lung cancer: a randomized, phase 2 cohort of the open-label KEYNOTE-021 study. Lancet Oncol. 2016 Nov;17(11):1497-1508. doi: 10.1016/S1470- 2045(16)30498-3. Epub 2016 Oct 10.

[15] Socinski MA, Jotte RM, Cappuzzo F, Orlandi F, Stroyakovskiy D, Nogami N, Rodríguez - Abreu D, Moro-Sibilot D, Thomas CA, Barlesi F, Finley G, Kelsch C, Lee A, Coleman S, Deng Y, Shen Y, Kowanetz M, Lopez-Chavez A, Sandler A, Reck M; IMpower150 Study Group. Atezolizumab for First-Line Treatment of Metastatic Nonsquamous NSCLC. N Engl J Med. 2018 Jun 14;378(24):2288-2301. doi: 10.1056/NEJMoa1716948. Epub 2018 Jun 4.

10. APPENDICES

Appendix 1: Common Terminology Criteria for Adverse Events

AEs in this study will be reported using CTCAE V5.0. CTCAE v5.0 can be downloaded from the home page of Cancer Therapy Evaluation Project (CTEP). CTCAE v5.0 should be applied in all relevant treatment centres. The URL is as follows:

https://ctep.cancer.gov/protocolDevelopment/electronic_applications/docs/CTCAE_v5_Quick_Reference_5x7.pdf

Appendix 2: Response Evaluation Criteria in Solid Tumors (RECIST 1.1)

Here below are Response Evaluation Criteria in Solid Tumors RECIST Version 1.1, and for more details, please refer to its English versionhttp://ctep.cancer.gov/protocolDevelopment/docs/recist_guideline.pdf.

Method

- Currently, CT and MRI are the best reproducible methods used for assessing the response of selected target lesions. The lesion on the CT scan is measured according to the following assumption: CT slice thickness is 5 mm or less. When CT scans have slice thickness greater than 5 mm, the minimum size for a measurable lesion should be at least 10 mm or the double of slice thickness. The use of MRI to assess diseases throughout the entire study is acceptable.

- Through the entire trial, the same assessment method and the same technique are used to characterize each identified and reported lesion.

- Ultrasound diagnosis (US) shall not be used to measure objective tumor response or progressive disease. For this study protocol, cross sectional imaging techniques (CT or MRI) are used to assess complete responses, partial response, or stable disease. FDG-PET examination is not suitable for assessing tumor response. It is sometimes reasonable to incorporate the use of FDG-PET scanning with complement CT scanning in assessment of progressive disease. To determine progressive disease (PD), new lesions can be identified using FDG-PET imaging according to the following rules:

    1) Negative FDG-PET at baseline with a positive FDG-PET at follow-up is a sign of PD based on a new lesion.

    2) No FDG-PET at baseline and a positive FDG-PET at follow-up (if the positive FDG-PET at follow-up corresponds to a new lesion confirmed by CT) is considered as PD. If the positive FDG-PET at follow-up is not confirmed as a new lesion by CT, an additional follow-up CT scan is needed to determine if there is progression at that lesion (if so, the date of PD will be the date of the first abnormal FDG-PET scan). If the positive FDG-PET at follow-up corresponds to a pre-existing site of disease on CT that is not progressing based on the anatomic images, this is not considered as PD.

If the investigator decides to use combined PET-CT, that CT shall not be substituted for dedicated CT examination required by this study protocol to complete the RECIST measurement, unless the research institute may confirm that the CT performed as part of a PET-CT is of identical diagnostic quality to a diagnostic CT (with IV and oral contrast).

Cytology and histology can be used to differentiate between PR and CR in rare cases if required

by protocol (for example, to distinguish residual benign lesions from residual malignant lesions after treatment). When effusions are known to be a potential adverse effect of treatment (e.g. with certain taxane compounds or angiogenesis inhibitors), the cytological confirmation of the neoplastic origin of any effusion that appears or worsens during treatment can be considered if the measurable tumor has met criteria for response or stable disease, in order to differentiate between response (or stable disease) and PD.

The definitions of "measurable" and "non-measurable" tumors:

All measurements should be recorded in metric notation, using rulers or calipers. Measurement results shall be recorded in a single dimension. At baseline, tumor lesions/pathological lymph nodes will be categorized as measurable or non-measurable according to the following definitions:

- **Measurable**: The tumor lesions must be accurately measured in at least one dimension (longest diameter in the plane of measurement is to be recorded) with a minimum size of 10 mm by CT scan (CT scan slice thickness no greater than 5 mm). When the CT scan slice thickness is greater than 5 mm, the longest diameter of the measurable lesion shall be at least 10 mm or twice of the slice thickness. Malignant lymph nodes: To be considered pathologically enlarged and measurable, a lymph node must be ≥ 15 mm in short axis when assessed by CT scan.
- **Non-measurable**: All other lesions, including small lesions (longest diameter < 10 mm or pathological lymph nodes with ≥ 10 to < 15 mm short axis), as well as truly non-measurable lesions, that are characterized as non-target lesions. Lesions considered truly non-measurable include: bone lesions, leptomeningeal disease, ascites, pleural or pericardial effusion, inflammatory breast disease, lymphangitic involvement of skin or lung, abdominal masses/abdominal organomegaly identified by physical exam that is not measurable by reproducible imaging techniques. Nodes that have a short axis < 10 mm are considered non-pathological and need not to be recorded or followed.

  Clinical lesions will only be considered measurable when they are superficial and ≥ 10 mm diameter as assessed using calipers (e.g. skin nodules and tangible lymph nodes). For the case of skin lesions, documentation by color photography, including a ruler to estimate the size of the lesion, is suggested. Lesions which cannot be accurately measured with calipers should be recorded as non-measurable.

Special considerations regarding lesion measurability:

Lytic bone lesions or mixed lytic-blastic lesions, with identifiable soft tissue components (that can be evaluated by cross sectional imaging techniques such as CT or MRI) can be considered as measurable lesions if the soft tissue component meets the definition of measurability described above. Blastic bone lesions are non-measurable.

Cystic lesions: Lesions that meet the criteria for radiographically defined simple cysts should not be considered as malignant lesions. "Cystic lesions" thought to represent cystic metastases that can be considered as measurable lesions, if they meet the definition of measurability described above. However, if noncystic lesions are present in the same patient, they are preferred for selection as target lesions.

Tumor lesions situated in a previously irradiated area, or in an area subjected to other loco-regional therapy, are usually not considered measurable unless there has been demonstrated progression in the lesion.

Baseline (i.e., before treatment) documentation of "target" and "non-target" lesions

During treatment, a maximum of five target lesions are selected for measurement (a maximum of two lesions per organ). Target lesions should be selected based on their size and suitability for accurate repeated measurements (either by imaging techniques or clinically).

A sum of the diameters (longest for non-nodal lesions, short axis for nodal lesions) for all target lesions will be calculated and reported as the baseline sum diameters. The baseline sum diameters will be used as reference to further characterize any objective tumor regression in the measurable dimension of the disease.

- All other lesions (or affected sites; including any measurable lesions or pathological lymph nodes not selected as target lesions) shall be identified as non-target lesions. Non-target lesions shall be recorded and qualitatively assessed during treatment. Measurements are not required and these lesions should be followed as "present", "absent", or in rare cases "unequivocal progression".
- Bone lesions: Bone scan, PET scan, or plain films are not considered adequate imaging techniques to measure bone lesions. If a sign or symptom indicative of bone metastases is present, a bone scan, MRI, CT, PET, PET/CT, or X-ray scan shall be performed. For subjects who are positive for bone scans or PET scans, another imaging technique (e.g., X-ray, CT, or MRI) must be used to confirm bone metastasis.

<u>Response criteria</u>

A subject's tumor response is assessed based on the response for target and non-target lesions, as well as the appearance of new lesions and disappearance of old lesions.

Evaluation of target lesions

*Complete Response (CR):	Disappearance of all target lesions. Any pathological lymph nodes (whether target or non-target) must have a reduction in short axis to < 10 mm.
*Partial Response (PR):	At least a 30% decrease in the sum of diameters of target lesions, taking as reference the baseline sum diameters.

*Progressive Disease (PD):	At least a 20% increase in the sum of diameters of target lesions, taking as reference the smallest sum in study. In addition to the relative increase of 20%, the sum must also demonstrate an absolute increase of at least 5 mm. (Note: the appearance of one or more new lesions is also considered progression).
*Stable Disease (SD):	Neither sufficient shrinkage to qualify for PR nor sufficient increase to qualify for PD, taking as reference the smallest sum diameters while on study.
Not Applicable (NA):	No target lesions are identified at baseline.
Not Evaluable (NE):	The scan is not completed, the scan result is incomplete, or the scan is not evaluated due to poor quality of the scan at the time point chosen for the evaluation of target lesions.

*** Diameter to be used:**

For lymph node lesions: the shortest axis

For non-lymph node lesions: the sum of the longest diameters

Once the study is started, the following rule will be adopted: If the lesion is believed to be present and is faintly seen but too small to measure, a default value of 5 mm should be assigned. If the size of the lesion increases to 5 mm or more in one dimension, their actual diameter shall be recorded. Lymph nodes identified as target lesions should always have the actual short axis measurement recorded even if the nodes regress to below 10 mm during the study. This means that when lymph nodes are included as target lesions, the "sum" of lesions may not be zero even if complete response criteria are met, since a normal lymph node is defined as having a short axis of < 10 mm. In order to qualify for CR, each node must achieve a short axis < 10 mm. For PR, SD, and PD, the actual short axis measurement of the nodes is to be included in the sum of target lesions.

Evaluation of non-target lesions

Complete Response (CR):	Disappearance of all non-target lesions. All lymph nodes must be non-pathological in size (short axis < 10 mm)
Non-CR/Non-PD:	Persistence of one or more non-target lesion(s)
Progressive Disease (PD):	Appearance of one or more new lesions, or if the original non-target lesions show suspicious progression.
Not Applicable (NA):	No non-target lesions are identified at baseline.
Not Evaluable (NE):	The scan is not completed, the scan result is incomplete, or the scan is not evaluated due to poor quality of the scan at the time point chosen for the evaluation of non-target lesions.

When the patient also has measurable lesions, in this setting, to achieve "unequivocal progression" based on the non-target disease, there must be an overall level of substantial worsening in non-target disease. Even in presence of SD or PR in target disease, the overall tumor burden has increased sufficiently to merit discontinuation of therapy. When the patient has only non-measurable disease, the increase in overall disease burden based on the change in

non-measurable disease is comparable in magnitude to the increase that would be required to declare PD for measurable disease (which is equivalent to a 20% increase in the sum of diameters in all measurable lesions).

Evaluation of best overall response

The status of the overall response of subjects at various time points is calculated as follows:

Target lesions	Non-target lesions	New lesions	Overall response
CR	CR	None	CR
CR	Non-CR/non-PD	None	PR
CR	NE	None	PR
PR	Non-PD or N/E	None	PR
SD	Non-PD or N/E	None	SD
NE	Non-PD	None	NE
PD	Any	Yes or No	PD
Any	PD	Yes or No	PD
Any	Any	Yes	PD

CR = complete response, PR = partial response, SD = stable disease, PD = progressive disease, and NE = not evaluable

Time point response of patients with non-target disease only

Non-target lesions	New lesions	Overall response
CR	None	CR
Non-CR/non-PD	None	Non-CR/non-PD
NE	None	NE
Unequivocal PD	Yes or No	PD
Any	Yes	PD
A "Non-CR/Non-PD" is preferred over "stable disease" for non-target disease since SD is increasingly used as an endpoint for assessment of efficacy in some trials so to assign this category when no lesions can be measured is not advised.		

<u>Special notes on response assessment</u>

Patients with a global deterioration of health status requiring discontinuation of treatment without objective evidence of progressive disease at that time should be reported as "symptomatic progression". In this case, it is not possible at this time to use "progressive disease" as an overall objective response of the tumor. Every effort should be made to document objective progression even after discontinuation of treatment.

In some circumstances, it may be difficult to distinguish residual disease from normal tissue. When the evaluation of complete response depends upon such determination, it is recommended that the residual lesion be further investigated (via fine needle aspirate/biopsy) before assigning a

status of complete response.

For equivocal findings of progression (e.g. very small and uncertain new lesions; cystic changes or necrosis in existing lesions), treatment may continue until the next scheduled assessment. If progression is confirmed at the next scheduled assessment, the date of progression should be recorded as the date when progression was suspected.

Duration of overall response

The duration of overall response is measured from the time measurement criteria are first met for CR/PR (whichever is recorded earlier) until the first date that recurrent or progressive disease is objectively documented (taking as reference for progressive disease the smallest measurement recorded in study).

Appendix 3: Quality of Life Scale EORTC QLQ-C30, EQ-5D-5L, EORTC QLQ-LC13

EORTC QLQ-C30 (3rd Edition)

We would like to know something about you and your health. Please answer all the questions below in person. There is no "right" or "wrong" in the answers here. Only circle the figure that best reflects your situation. The information you provide will be kept strictly confidential.

	Not at all	Somewhat	Quite	Very much
1. Is it difficult for you to engage in some laborious activities, such as carrying a heavy shopping bag or suitcase?	1	2	3	4
2. Is it difficult for you to walk long distances?	1	2	3	4
3. Is it difficult for you to walk for short distances outdoors?	1	2	3	4
4. Do you need to stay in bed or chair during the day?	1	2	3	4
5. Do you need help from others when eating, dressing, bathing or going to the bathroom?	1	2	3	4
**During the past week:**				
6. Did you feel restricted in your work and daily activities?	1	2	3	4
7. Did you feel restricted when engaging in your hobbies or leisure activities?	1	2	3	4
8. Were you short of breath?	1	2	3	4
9. Did you have pain?	1	2	3	4
10. Did you need to rest?	1	2	3	4
11. Did you have trouble sleeping?	1	2	3	4
12. Did you feel weak?	1	2	3	4
13. Was your appetite weak (Did you have no appetite)?	1	2	3	4
14. Did you feel sick?	1	2	3	4
15. Did you vomit?	1	2	3	4
16. Did you have constipation?	1	2	3	4
**During the past week:**	Not at all	Somewhat	Quite	Very much
17. Did you have diarrhea?	1	2	3	4
18. Did you feel tired?	1	2	3	4
19. Did pain affect your daily activities?	1	2	3	4
20. Did you have difficulty concentrating on doing things, such as reading a newspaper or watching TV?	1	2	3	4
21. Did you feel nervous?	1	2	3	4
22. Did you feel worried?	1	2	3	4
23. Did you have a bad temper?	1	2	3	4
24. Did you feel depressed (low-spirited)?	1	2	3	4
25. Did you have a bad memory?	1	2	3	4
26. Did your physical condition or treatment affect your family life?	1	2	3	4
27. Did your physical condition or treatment affect your social activities?	1	2	3	4
28. Was your physical condition or treatment making you financially difficult?	1	2	3	4

For the following questions, please circle the number among 1-7 that best reflects your situation

29. How would you rate your overall health during the past week?

1	2	3	4	5	6	7
Very poor						Very good

30. How would you rate your overall quality of life during the past week?

1	2	3	4	5	6	7
Very poor						Very good

EORTC QLQ- LC13

Patients sometimes report that they have the following symptoms or problems. Please indicate the extent of these symptoms or problems you have experienced during the past week and circle the figure that best suits you.

**During the past week:**		**Not at all**	**A little**	**Quite a bit**	**Very much**
31.	How bad is your cough?	1	2	3	4
32.	Did you cough up blood (with blood in your sputum)?	1	2	3	4
33.	Did you feel short of breath while resting?	1	2	3	4
34.	Did you feel shortness of breath when walking?	1	2	3	4
35.	Did you feel short of breath while climbing stairs?	1	2	3	4
36.	Did you experience pain in your mouth or tongue?	1	2	3	4
37.	Did you experience difficulty swallowing?	1	2	3	4
38.	Did you experience tingling in your hands or feet?	1	2	3	4
39.	Did you lose hair?	1	2	3	4
40.	Did you experience chest pain?	1	2	3	4
41.	Did you experience pain in your arms or shoulders?	1	2	3	4
42.	Did you experience pain in other parts of your body?	1	2	3	4
	If any, which part (please write down):				
43.	Did you take painkillers?				
	1 No 2 Yes				
	If you took painkillers, did the painkillers work well?	1	2	3	4

EQ-5D-5L

Under each heading, please tick the ONE box that best describes your health TODAY.

Mobility

I have no problems in walking about ☐

I have slight problems in walking about ☐

I have moderate problems in walking about ☐

I have severe problems in walking about ☐

I am unable to walk about ☐

Self-care

I have no problems washing or dressing myself ☐

I have slight problems washing or dressing myself ☐

I have moderate problems washing or dressing myself ☐

I have severe problems washing or dressing myself ☐

I am unable to wash or dress myself ☐

Daily activities *(e.g., work, study, housework, family or leisure activities)*

I have no problems doing my usual activities ☐

I have slight problems doing my usual activities ☐

I have moderate problems doing my usual activities ☐

I have severe problems doing my usual activities ☐

I am unable to do my usual activities ☐

Pain/discomfort

I have no pain or discomfort ☐

I have slight pain or discomfort ☐

I have moderate pain or discomfort ☐

I have severe pain or discomfort ☐

I have extreme pain or discomfort ☐

Anxiety or depression

I am not anxious or depressed ☐

I am slightly anxious or depressed ☐

I am moderately anxious or depressed ☐

I am severely anxious or depressed ☐

I am extremely anxious or depressed ☐

- We would like to know how good or bad your health is TODAY.
- This scale has numbers from 0 to 100 on it.
- 100 means the best health you can imagine.
- 0 means the worst health you can imagine.
- Please mark an " × " on the scale to indicate your health today.
- Now, please write down the number you marked on the scale in the space below.

Your health state today = ☐

The best health you can imagine

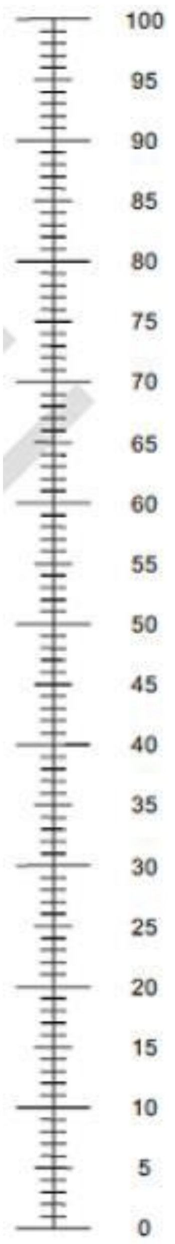

0 means the worst health you can imagine

Appendix 4: Eastern Cooperative Oncology Group (ECOG) - Performance Status Scale

Score	ECOG Status
0	Fully active, able to carry on all pre-disease performance without restriction.
1	Restricted in physically strenuous activity but ambulatory and able to carry out work of a light or sedentary nature, e.g., light house work, office work.
2	Ambulatory and capable of all self care but unable to carry out any work activities. Up and about more than 50% of waking hours
3	Capable of only limited self care, confined to bed or chair more than 50% of waking hours.
4	Completely disabled. Cannot carry on any self care. Completely confined to bed or chair.
5	Death

Appendix 5: Fridericia's Correction Formula

Fridericia's formula: $QTc = QT/RR^{0.33}$

Appendix 6: New York Heart Association Classification

Grade	Symptoms
**Class I**	No limitation of physical activity. Ordinary physical activity does not cause undue fatigue, palpitation, dyspnea (shortness of breath).
**Class II**	Slight limitation of physical activity. Comfortable at rest. Ordinary physical activity results in fatigue, palpitation, dyspnea (shortness of breath).
**Class III**	Marked limitation of physical activity. Comfortable at rest. Less than ordinary activity causes fatigue, palpitation, or dyspnea.
**Class IV**	Unable to carry on any physical activity with discomfort. Symptoms of heart failure at rest. If any physical activity is undertaken, discomfort increases.

Appendix 7: Prohibited Traditional Chinese Medicines during the Trial

**Prohibited Traditional Chinese Medicines**	
Hua Zheng Hui Sheng Tablet	Anticancer Ping Pill
Brucea Javanica Oil Soft Capsule/Brucea Javanica Oil Injection	Fu Kang Capsule
Zhe Mu Syrup	Xiao Ai Ping
Cantharidin/Cantharidin Injection/Cantharidin Capsule	Ping Xiao Capsule
Hua Chan Su	Ping Xiao Tablet
Toad Venom	Shen Dan San Jie Capsule
Kang Ai Injection	An Kang Xin Capsule
Kang Lai Te	Bo Sheng Ai Ning
Herba Sarcandrae Injection	Zedoary Turmeric Oil Glucose Injection
Ai Di Injection	Kang Li Xin Capsule
A Wei Hua Pi Cream	Ci Dan Capsule
Shenmai	Lightyellow Sophora Root

Note: Traditional Chinese medicines prohibited during the trial include but are not limited to the above drugs.
