# Supplementary material for: Serplulimab Plus Chemotherapy, with or without HLX04, versus Chemotherapy as First-Line Treatment for Nonsquamous NSCLC: Final Survival Analysis of the Phase III ASTRUM-002 Study
Source: Cancer Commun (Lond). 2026 Jun 10;46:0034. doi: 10.34133/cancomm.0034 (PMC13250280; doi:10.34133/cancomm.0034)
Supplement: Supplementary 1 — Figs. S1 and S2 Tables S1 to S7 Data S1 and S2 [file cancomm.0034.f1.zip › Supplementary Data S2-Final.pdf]

A THREE-ARM, RANDOMIZED, DOUBLE-BLIND, MULTICENTER, PHASE III CLINICAL STUDY TO EVALUATE HLX10 (RECOMBINANT HUMANIZED ANTI-PD-1 MONOCLONAL ANTIBODY INJECTION) IN COMBINATION WITH CHEMOTHERAPY (CARBOPLATIN-PEMETREXED) VERSUS HLX10 + HLX04 (RECOMBINANT ANTI-VEGF HUMANIZED MONOCLONAL ANTIBODY INJECTION) IN COMBINATION WITH CHEMOTHERAPY (CARBOPLATIN-PEMETREXED) VERSUS CHEMOTHERAPY (CARBOPLATIN-PEMETREXED) AS d TREATMENT OF ADVANCED NON-SQUAMOUS NON-SMALL CELL LUNG CANCER (NSCLC)

Protocol Number: HLX10-002-NSCLC301

STATISTICAL ANALYSIS PLAN

Version No.:1.0

Date: 31 Jul. 2023

Statistical Analysis Plan Signature Page

A Three-Arm, Randomized, Double-Blind, Multicenter, Phase III Clinical Study to Evaluate HLX10 (Recombinant Humanized Anti-PD-1 Monoclonal Antibody Injection) in Combination with Chemotherapy (Carboplatin-Pemetrexed) Versus HLX10 + HLX04 (Recombinant Anti-VEGF Humanized Monoclonal Antibody Injection) in Combination with Chemotherapy (Carboplatin-Pemetrexed) Versus Chemotherapy (Carboplatin-Pemetrexed) as First-Line Treatment of Advanced Non-Squamous Non-Small Cell Lung Cancer (NSCLC)

Protocol Number: HLX10-002-NSCLC301

Statistical Analysis Plan Version No.: 1.0

蔡志玉	31 Jul. 2023
Statistician: Zhiyu Cai	Date<br>Shanghai Henlius Biotech, Inc.
朱怡	31 Jul. 2023
Statistician Lead: Yi Zhu	Date<br>Shanghai Henlius Biotech, Inc.
魏雪	31 Jul. 2023
Medical Manager: Xue Wei	Date<br>Shanghai Henlius Biotech, Inc.
沈圆圆	31 Jul. 2023
Manager for Clinical Pharmacology and Toxicology: Yuanyuan Shen	Date<br>Shanghai Henlius Biotech, Inc.

Revision

Version No.	Date of revision	Revised by	Description of change
0.1	2019-10-12	Li Li	First version
0.2	2020-08-20	Xiao Qi	Revised based on Protocol V4.0
0.3	2021-01-14	Daowei Yang	Revised based on Protocol V5.0
0.4	2021-08-24	Daowei Yang	Updated the summary text in the AE section and deleted the summary of AEs during the screening period. Deleted the KM analysis of follow-up time and changed it to the descriptive analysis of follow-up time for OS.
0.5	2021-09-06	Daowei Yang	Updated the content of analysis of dose interruption and dose modification in the AE section. Updated the content of immunogenicity analysis. Updated the PD-L1 subgroup analysis. Updated the analysis content in the Quality of Life Assessment section. Simplified the definitions of prior medication, concomitant medication, etc. Deleted the time from prior anti-tumor therapy to informed consent.
0.6	2023-03-7	Zhiyu Cai	1. Added the efficacy hypothesis for Group A and Group B in the Sample Size section.<br>2. Updated the design of interim analysis: canceled the interim analysis for PFS.<br>3. Added the definition of estimand in accordance with the E9R1 Guideline.<br>4. Deleted the statistical analysis of PFS2 endpoint according to Protocol V7.0.
Final 1.0	2023-07-31	Zhiyu Cai	1. Clarified the number of interim analyses for OS, analysis time points, and preset efficacy boundary.

TABLE OF CONTENTS

TABLE OF CONTENTS 4
ABBREVIATIONS AND TERMINOLOGY 7
1 INTRODUCTION 9
2 OBJECTIVES 9
2.1 Primary Objectives 9
2.1.1 Stage I: a single-arm study (safety run-in phase) 9
2.1.2 Stage II: phase III study 9
2.2 Secondary Objectives 10
2.2.1 Stage I: a single-arm study (safety run-in phase) 10
2.2.2 Stage II: phase III study 10
3 STUDY DESIGN 10
3.1 Overall Design 10
3.2 Endpoints 17
3.2.1 Efficacy endpoints and estimands 18
3.2.2 Safety endpoints 20
3.2.3 Quality of life endpoints 21
3.2.4 Pharmacokinetics endpoints 22
3.2.5 Immunogenicity endpoints 22
3.2.6 Biomarker 22
3.3 Sample size 22
3.4 Statistical Hypothesis 23
3.5 Randomization and Blinding 23
3.6 Safety Data Monitoring and Interim Analysis 24
4 ANALYSIS POPULATIONS 26
4.1 Intention-To-Treat (ITT) Set 26
4.2 Per Protocol Set (PPS) 26
4.3 Safety Set (SS) 26
4.4 Pharmacokinetic Set (PKS) 26
5 STATISTICAL METHODS 26
5.1 General Principles 26
5.1.1 Statistical analysis principles 26
5.1.2 Data processing principles 28

5.1.3 Multiplicity comparison 32
5.2 Subjects 32
5.2.1 Subject disposition 32
5.2.2 Major protocol deviations 33
5.3 Demographics and Baseline Characteristics 33
5.3.1 Demographic data 33
5.3.2 Baseline tumor diagnosis 34
5.3.3 Medical history 35
5.3.4 Prior and concomitant medications 35
5.3.5 Prior and concomitant non-drug procedures 35
5.3.6 History of smoking, alcohol use, allergies, and drug dependence 36
5.3.7 Prior anti-tumor therapy 37
5.3.8 Biomarker and mutation tests at screening 38
5.3.9 Other examinations at screening 39
5.3.10 Treatment for NSCLC during the study 39
5.4 Efficacy Analysis 41
5.4.1 Analysis of primary efficacy endpoint 41
5.4.2 Analysis of key secondary efficacy endpoint 44
5.4.3 Secondary efficacy endpoints 46
5.4.4 Subgroup analysis 48
5.4.5 Other analyses 49
5.5 Safety Analysis 50
5.5.1 Drug exposure and compliance 50
5.5.2 Adverse event 51
5.5.3 Laboratory tests 55
5.5.4 Vital signs 55
5.5.5 Physical examination 56
5.5.6 12-Lead ECG 56
5.5.7 ECOG performance scores 56
5.6 Immunogenicity Analysis 57
5.7 Pharmacokinetic Analysis 57
5.8 Quality of Life Analysis 58
5.8.1 EQ-5D-5L 58
5.8.2 EORTC QLQ-C30 59

5.8.3 EORTC QLQ-LC13 60

6 CHANGES TO PROTOCOL/STATISTICAL ANALYSIS PLAN 61

7 REFERENCES 64

ABBREVIATIONS AND TERMINOLOGY

Abbreviations and Terminology	Explanation
ADA	Anti-drug antibody
AE	Adverse event
ATC	Anatomical Therapeutic Chemical
BMI	Body Mass Index
BOR	Best overall response
CI	Confidence Interval
C_{max}	Maximum concentration
CMH	Cochran-Mantel-Haenszel test
CR	Complete response
CRF	Case report form
CT	Computerized tomography
CTCAE	Common Terminology Criteria for Adverse Events
C_{trough}	Trough concentration
DOR	Duration of response
ECOG	Eastern Cooperative Oncology Group
EORTC	European Organization for Research on Treatment of Cancer
QLQ-C30	QLQ-C30
QLQ-LC13	QLQ-LC13
EQ-5D-5L	EQ-5D-5L
FT3	Free Triiodothyronine
FT4	Free thyroxine
H	High
HBcAb	Hepatitis B core antibody
HBsAg	Hepatitis B surface antigen
HBV	Hepatitis B virus
HCV	Hepatitis C virus
HIV	Human immunodeficiency virus
HR	Hazard ratio
ICF	Informed consent form
ICH	International Council for Harmonisation
IDMC	Independent Data Monitoring Committee
IRRC	Independent Radiological Review Committee
ITT	Intention-to-treat

Abbreviations and Terminology	Explanation
L	Low
LVEF	Left Ventricular Ejection Fraction
MedDRA	Medical Dictionary for Regulatory Activities
MRI	Magnetic resonance imaging
MSI	Microsatellite instability
NE	Not evaluated
NSCLC	Non-small cell lung carcinoma
ORR	Objective response rate
OS	Overall Survival
PD	Progressive disease
PFS	Progression-free survival
PK	Pharmacokinetic(s)
PKS	Pharmacokinetics set
PPS	Per protocol set
PR	Partial response
PT	Preferred Term
R_{ac}	Accumulation ratio
RECIST	Response Evaluation Criteria in Solid Tumor
SAE	Serious Adverse Event
SAP	Statistical Analysis Plan
SAS	Statistical Analysis System
SC	Steering Committee
SD	Stable disease
SOC	System Organ Class
SS	Safety set
T3	Triiodothyronine
T4	Thyroxine
TEAE	Treatment-emergent adverse event
TFL	Table, Figure and Listing
TMB	Tumor mutation burden
TSH	Thyroid stimulating hormone
VEGF	Vascular endothelial growth factor
WHODrug	World Health Organization Drug Dictionary

1 INTRODUCTION

Lung cancer is a common clinical malignant tumor. Its incidence and mortality are the highest among all malignant tumors, accounting for 15% with 5-year survival rate being less than 15%. Non-small cell lung cancer (NSCLC) is the most common type of primary lung cancer, accounting for approximately 80% of all lung cancers. Due to the insidious onset of NSCLC at the early stage, 70%–80% of patients are at advanced stage at the time of clinically definite diagnosis, losing the best time for surgical treatment, and a significant proportion of patients with early-stage NSCLC receiving surgical treatment will develop distant recurrence and die due to lung cancer.

HLX10 is an innovative monoclonal antibody targeting PD-1 independently developed by Shanghai Henlius Biotech, Inc. HLX04, a similar product of Avastin® from Roche, is a recombinant anti-VEGF humanized monoclonal antibody developed by Shanghai Henlius Biotech, Inc. Its humanized gene sequence is designed by Henlius according to the protein sequence of Avastin® of Roche.

This study has two stages: The first stage is a single-arm study and a safety run-in phase. The second stage is a three-arm, randomized, double-blind, multicenter phase III clinical study. The safety/PK/efficacy data in the first stage will be presented in listings only. This statistical analysis plan (SAP) mainly provides the statistical analysis methods and data processing principles for the analysis and reporting of the study-related data for the randomized controlled phase III study in the second stage. The SAP is generated based on the Clinical Study Protocol V7.0 (28 Nov., 2022).

2 OBJECTIVES

2.1 Primary Objectives

2.1.1 Stage I: a single-arm study (safety run-in phase)

To evaluate the safety and tolerability of HLX10 + HLX04 combined with chemotherapy as first-line treatment in patients with advanced non-squamous non-small cell lung cancer.

2.1.2 Stage II: phase III study

To evaluate the clinical efficacy of HLX10 combined with chemotherapy versus HLX10 + HLX04 combined with chemotherapy as first-line treatment in patients with advanced non-squamous non-small cell lung cancer.

2.2 Secondary Objectives

2.2.1 Stage I: a single-arm study (safety run-in phase)

To evaluate the clinical efficacy of HLX10 + HLX04 combined with chemotherapy as first-line treatment in patients with advanced non-squamous non-small cell lung cancer.

2.2.2 Stage II: phase III study

To evaluate the safety and tolerability of HLX10 combined with chemotherapy versus HLX10 + HLX04 combined with chemotherapy as first-line treatment in patients with advanced non-squamous non-small cell lung cancer.

3 STUDY DESIGN

3.1 Overall Design

This study has two stages: The first stage is a single-arm study and a safety run-in phase. Approximately 6–12 subjects with advanced non-squamous NSCLC will be enrolled in this stage. Six subjects will be enrolled for the first time to receive HLX10 + HLX04 combined with chemotherapy (carboplatin-pemetrexed) once every 3 weeks. After all subjects complete the first cycle of study treatment, the safety and tolerability will be confirmed by the Steering Committee (SC) to determine whether to proceed to the second stage of phase III randomized, double-blind, multicenter study. If "safety events" occur in ≤ 1 of 6 subjects (see "3.1 Overall Study Design" in the study protocol for the definition of safety events), it will be considered as good safety, and the second stage can be proceeded to. If "safety events" occur in ≥ 2 of 6 subjects, 6–12 subjects will be enrolled additionally. If "safety events" occur in < 4 of the 12 subjects, the second stage can be proceeded to; if "safety events" occur in ≥ 4 of the 12 subjects, the study on the current dose will be terminated, and the SC will decide whether to reduce the dose for exploration.

The second stage is a three-arm, randomized, double-blind, multicenter phase III clinical study to evaluate the clinical efficacy, safety, and tolerability of HLX10 combined with chemotherapy versus HLX10 + HLX04 combined with chemotherapy versus chemotherapy in subjects with non-squamous non-small cell lung cancer (NSCLC) who have not previously received systemic therapy for advanced NSCLC using the same dosing frequency as that in the safety run-in phase, collect pharmacokinetic (PK) parameters, and explore the biomarkers related to efficacy. Subjects will be randomized into the following groups:

- Group A (HLX10 + HLX04): HLX10 + HLX04 combined with chemotherapy (carboplatin-pemetrexed)

- Group B (HLX10): HLX10 + HLX04 placebo combined with chemotherapy (carboplatin-pemetrexed)
- Group C (control group): HLX10 placebo + HLX04 placebo combined with chemotherapy (carboplatin-pemetrexed)

This study includes the following periods: screening period (28 days); treatment period: the treatment period begins with the subject's enrollment, and the first dose should be administered within 3 days after randomization. Each subject will receive study treatment once every 3 weeks until loss of clinical benefit, or until the duration of treatment reaches 2 years (up to 35 dosing cycles), and then the investigator will decide whether to continue the treatment; and follow-up period (including safety follow-up period and survival follow-up period). If progressive disease occurs (the first PD, confirmed by the IRRC as per RECIST v1.1) in a subject, the investigator will determine whether to continue the treatment: after the occurrence of the first PD, continue the treatment as per the original regimen (safety run-in phase) or perform unblinding by the investigator and continue the treatment (second stage, with the treatment regimen detailed in section 4.3.2 of the protocol) for a maximum of 6 weeks and then perform tumor assessment again; if tumor progression occurs again (the second PD), end the study treatment for the subject and proceed to the follow-up period; if no progression occurs, continue the treatment. During the treatment, the subjects who did not have first PD should have an imaging examination every 6 weeks (± 7 days) within 48 weeks and every 12 weeks (± 7 days) after 48 weeks. For subjects who continue to receive the dose after PD, they should continue to have an imaging examination every 6 weeks (± 7 days). The schedule of study procedure is shown in Table 1.

Table 1. Schedule of activities

Period	Screening period[1]		Treatment period (each 3-week period is a treatment cycle)					End of treatment[2]	Follow-up period[3]		
**Treatment cycle/visit**	Screening period		1	2	3	4	n	End of treatment	Safety follow-up		Survival follow-up[4]
**Time of visit**									30 days after the last dose	90 days after the last dose	Every 12 weeks
**Window (day)[5]**	Days −28 to −8	Days −7 to −1		± 3	± 3	± 3	± 3	+ 7	± 7	± 7	± 7
**Management procedure**											
Informed Consent Form	X										
Eligibility criteria	X										
Dispensing of subject ID card	X										
Demographics and medical history	X										
Previous and concomitant therapies[6]	X		X	X	X	X	X	X	X	X	
**Clinical procedures/assessments**											
AEs[7]	X		X	X	X	X	X	X	X	X	
Quality of life[8]		X	X		X		X	X			
Echocardiogram	X										
12-Lead ECG	X			X	X	X	X	X	X		
Complete physical examination	X										
Symptom-oriented physical examination			X	X	X	X	X	X	X		
Height, weight, and vital signs[9]	X		X	X	X	X	X	X	X		

ECOG scores		X	X	X	X	X	X	X	X		
Subsequent anti-tumor therapy									X	X	X
Survival status			X	X	X	X	X	X	X	X	X
**Investigational product**											
Random			X								
HLX10/placebo, HLX04/placebo[10]			X	X	X	X	X				
Pemetrexed			X	X	X	X	X				
Carboplatin			X	X	X	X					
**Laboratory operations/ assessments: performed by study sites**											
Pregnancy test[11]		X			X		X	X	X		
Hematology, serum chemistry, coagulation, urinalysis, and myocardial enzymes[12]		X		X	X	X	X	X	X		
T3 or FT3, T4 or FT4, and TSH[13]		X			X		X	X	X		
HBV tests, HBV DNA[14]	X										
● In case of HBV DNA (−) and HBsAg (+), and/or HBcAb (+) at baseline, anti-HBV antibody and HBV DNA should be examined during the treatment period.					X		X	X	X		
HCV antibody, HCV RNA[14]	X										
● In case of HCV antibody (+) and HCV RNA (−) at baseline, anti-HCV antibody and HCV RNA should be examined during the treatment period.					X		X	X	X		

HIV	X									
**Laboratory operations/ assessments: performed at the central laboratory**										
PK, ADA [15]		X	X		X	X	X	X		
PD-L1 expression level, and MSI and TMB test[17]	X									
**Gene mutation status confirmation**										
EGFR, ALK, and ROS1 mutation statuses[18]	X									
**Response assessment**										
Radiological examination[16]	X			X		X	X			

This study is divided into two stages, stage I (safety run-in period) and stage II (phase III study), with the same procedure.

1. The screening period should not exceed 28 days. In this study, re-screening is allowed for ineligible subjects: In case of unqualified laboratory tests, a re-test can be performed within the screening time window if determined by the investigator, without the issuance of new screening numbers; and for other conditions incompliant with the inclusion/exclusion criteria, subjects should be re-screened with a new screening number.
2. If a subject discontinues the study treatment for any reason, an end-of-treatment visit should be performed whenever possible and should be completed within 7 days after the discontinuation is learned of or confirmed (and should be completed before the subject starts a new anti-tumor therapy).
3. All subjects are required to visit the study site for safety follow-up 30 days (± 7 days) after the last dose; and if the end-of-treatment visit is delayed for any reason and occurs after the time window of 30 days (± 7 days), no further safety follow-up visit is required. A follow-up telephone call for safety follow-up 90 days (± 7 days) after the last administration is required. Only the information of AEs and AE-related concomitant drugs is collected. For subjects who discontinued for reasons other than PD, radiological assessments are to be continued as scheduled, until PD, initiation of new anti-tumor therapy, withdrawal of ICF, death, or end of the study, whichever occurs first.
4. Subjects should be followed up for survival by telephone every 12 weeks ± 7 days after treatment discontinuation; the frequency of survival follow-up may be increased as appropriate.
5. The time window is 28 days for screening, 3 days for treatment (7 days for tumor assessment), and 7 days for follow-up and end-of-treatment visits. For ECOG performance status, pregnancy test, hematology, serum chemistry, coagulation, urinalysis, T3 or FT3, T4 or FT4, and TSH tests during screening period, data within 7 days prior to the randomization should be documented, and the subjects should meet the corresponding inclusion/exclusion criteria for enrollment.

6. All prior and concomitant medications are recorded from 30 days prior to the signing of ICF through the safety follow-up visit. Concomitant medications associated with AEs are recorded up to 90 days after the last study treatment.
7. All AEs and treatment emergent AEs are recorded from the time of the ICF signing until 90 days after the last study treatment. If the patient starts a new anti-tumor therapy during AE collection period, only AE information related to the study treatment is collected after the initiation of the new anti-tumor therapy.
8. Quality of life scales include the EQ-5D-5L, the European Organization for Research and Treatment of Cancer Quality of Life Scale (EORTC QLQ-C30), and the European Organization for Research and Treatment of Cancer Lung Cancer Questionnaire Module (EORTC QLQ-LC13). Such scales are evaluated prior to the first dose and every other subsequent dosing cycle (i.e., pre-dose in Cycles 1, 3, 5, 7, etc.) until the end of treatment. A quality-of-life assessment is required at the end-of-treatment visit if no assessment is performed within the past 3 weeks. Re-assessments prior to dosing in Cycle 1 are not required for subjects who have a quality-of-life assessment on Day −7 to Day −1 of the screening period.
9. The height measurement is performed only at screening and vital signs include body temperature, pulse, respiratory rate, and blood pressure. Body weight is measured before each administration.
10. The investigational product is administered based on 3-week cycles after all clinical and laboratory operations/assessments are completed. No more than 3 days should be elapsed between the date of randomization and the date of the study treatment initiation.
11. Women of childbearing potential must have a serum pregnancy test. During the treatment period, the test should be done within 3 days pre-dose every 2 cycles. Analysis will be carried out in the local study site.
12. Routine laboratory tests include hematology, serum chemistry, coagulation, myocardial enzymes, and urinalysis. These tests will be carried out within 3 days pre-dose in each cycle; if aforementioned laboratory tests are scheduled on the same day as the study treatment, the study treatment can be arranged only after the test results are obtained. During the carboplatin therapy, routine blood tests should be performed on Day 8 (± 3 days) of each treatment cycle to closely monitor bone marrow suppression.
13. Thyroid function tests include triiodothyronine (T3 or FT3), thyroxine (T4 or FT4), and thyroid-stimulating hormone (TSH) assays. During the treatment period, the test should be done within 3 days pre-dose every 2 cycles. Analysis will be carried out in the local study site.
14. All subjects should be tested for HBV markers (HBsAg, HBsAb, HBeAg, HBeAb, and HBcAb) and HCV antibody during screening; HBsAg or HBcAb positive subjects should be further tested for HBV DNA titer; and anti-HCV antibody positive subjects should be further tested for HCV RNA. In case of HBV DNA (−) and 1) HBsAg (+), and/or 2) HBcAb (+) during screening (baseline), HBV antibody and HBV DNA should be tested every 2 cycles in the treatment period. In case of 1) HCV antibody (+) and HCV RNA (−) at baseline, HCV antibody and HCV RNA should be tested every 2 cycles in the treatment period. The investigator will receive antiviral treatment as clinically needed.
15. PK and ADA sampling: (Note: ADA samples will only be collected at pre-dose and procedures are described in the laboratory manual.)
    - Blood samples will be collected at the following points: within 7 days pre-dose in Cycle 1; within 3 days pre-dose in Cycles 2, 4, 6, 8, and every 4 cycles thereafter; within 2 hours after the end of HLX10 or placebo/HLX04 or placebo dosing in Cycles 1 and 8 of treatment period (for PK only); and at end-of-treatment visit and/or safety follow-up.

16. CT or MRI should be performed at screening and after the start of study treatment on sites including brain, chest, abdomen, pelvic cavity and any other sites suspected to have tumor lesions, among which brain MRI or CT (preferably MRI) and bone scans are performed during baseline period, and then in treatment period are performed as determined by the investigator according to clinical needs; examination methods at the same site should be consistent as much as possible throughout the study; and if there are no contraindications, contrast agent should be used. During the treatment, for subjects without first PD, radiological assessment should be performed once every 6 weeks (± 7 days) within 48 weeks, and once every 12 weeks (± 7 days) thereafter. For any subject continuing medication after PD, radiological assessment should be repeated every 6 weeks (± 7 days). The investigator and IRRC respectively assess the tumor images according to RECIST v1.1 (the anti-tumor efficacy assessment can be performed by the investigator according to clinical needs), and the investigator should make subsequent treatment judgment according to the results of their own response assessment. If a tumor assessment is performed within 28 days prior to the first dose by the same method and devices in the same hospital, it may serve as the baseline tumor assessment. If the investigator evaluates as PD for the first time, the IRRC needs to evaluate the tumor imaging according to RECIST v1.1. If the IRRC confirms as PD, the investigator will unblind and decide whether to continue the treatment. At the end-of-treatment visit, if tumor imaging is performed within the last 4 weeks, a re-test is not required. For subjects who discontinued for reasons other than PD, radiological assessments are to be continued as scheduled, until PD, initiation of new anti-tumor therapy, withdrawal of ICF, death, or end of the study, whichever occurs first.
17. In the screening period, the subjects must provide formalin-fixed paraffin-embedded (FFPE) tumor samples (paraffin blocks or unstained sections) (samples within 6 months prior to the first study treatment are recommended) at non-radiotherapy sites collected at or after the diagnosis of advanced NSCLC for PD-L1 expression level determination in the central laboratory, and pathological reports of such specimens. In the absence of recent archival tumor tissue samples, a fresh biopsy of a tumor lesion at screening should be accepted to obtain the corresponding tumor samples for PD-L1 expression level determination (the number of samples depends on biopsy). If the subject agrees, tumor tissue samples and blood samples can be collected for MSI and TMB testing in the central laboratory. If pathological sampling is performed for subjects during the study treatment, it is recommended to collect their tumor samples; and these tumor tissue sections will be used for immunohistochemical PD-L1 expression level determination, to evaluate the relationship between PD-L1 expression level in tumor cells and tumor-infiltrating immune cells/MSI/TMB and efficacy, and other purposes. Specimens obtained through fresh collection, excision, core needle biopsy, resection, incision, punch biopsy, or forceps biopsy are all acceptable. Needle aspiration samples (i.e., samples that lack complete tissue structure and provide only a cell suspension and/or cell smear), brush samples, and cytosol samples from pleural or peritoneal fluid are not acceptable. See "Laboratory Operation Manual" for detailed requirements for tissue samples.
18. The subjects are required to provide test reports on the mutation status of EGFR, ALK and ROS1 genes (using the method specified in the NCCN guidelines, and not accepting blood test results alone). Subjects should be excluded if they are known to have EGFR sensitive mutation or anaplastic lymphoma kinase (ALK) or ROS1 gene rearrangement or fusion mutation. If the status of EGFR, ALK, or ROS1 is unknown, EGFR, ALK, and ROS1 mutation will be tested in the screening period.

3.2 Endpoints

Stage I: a single-arm study (safety run-in phase)

Primary endpoint:

- Safety and tolerability of the first cycle of study treatment;

Secondary endpoints:

- Incidence rates of adverse events (AEs) and serious adverse events (SAEs);
- Overall survival (OS);
- Progression-free survival (PFS, assessed by the IRRC and the investigator as per RECIST v1.1);
- Objective response rate (ORR, assessed by the IRRC and the investigator as per RECIST v1.1);
- Duration of response (DOR, assessed by the IRRC and the investigator as per RECIST v1.1);
- Pharmacokinetics (PK): serum HLX10/HLX04 concentration;
- Immunogenicity assessment: positive rate of anti-drug antibody (ADA);
- Relationship between PD-L1 expression level, MSI, TMB in tumor tissues and efficacy;
- Quality of life assessment.

Stage II: phase III study

Primary endpoint:

- Progression-free survival (PFS, assessed by IRRC as per RECIST v1.1);

Secondary endpoints:

- Overall survival (OS), as a key secondary endpoint in this study;
- Progression-free survival (PFS, assessed by the investigator as per RECIST v1.1);
- Objective response rate (ORR, assessed by the IRRC and the investigator as per RECIST v1.1);
- Duration of response (DOR, assessed by the IRRC and the investigator as per RECIST v1.1);

- Incidence rates of adverse events (AEs) and serious adverse events (SAEs);
- Pharmacokinetics (PK): serum HLX10/HLX04 concentration;
- Immunogenicity assessment: positive rate of anti-drug antibody (ADA);
- Relationship between PD-L1 expression level, MSI, TMB in tumor tissues and efficacy;
- Quality of life assessment.

3.2.1 Efficacy endpoints and estimands

3.2.1.1 Primary efficacy endpoint and estimand

Progression-free survival (PFS, assessed by IRRC as per RECIST v1.1): defined as the time from randomization to the first documentation of PD or death due to any reason (whichever occurs first).

Population: Patients receiving first-line treatment for advanced non-squamous non-small cell lung cancer (NSCLC) who meet the inclusion and exclusion criteria

Treatment: HLX10/placebo + HLX04/placebo combined with chemotherapy (carboplatin-pemetrexed), with every 3 weeks (21 days) as a treatment cycle

Variable: Progression-free survival (PFS) assessed by IRRC as per RECIST v1.1

Intercurrent events and handling strategies:

#	Intercurrent events	Handling strategies and description
1	Initiation of new anti-tumor therapy before PD or death	**Hypothetical strategy**: Assuming that the risk of PD or death in subjects who start a new anti-tumor therapy is the same as that in subjects who do not start a new anti-tumor therapy, censor to the date of the last tumor imaging assessment before the start of new anti-tumor therapy.
2	Death	**Composite strategy**: Death and PD are jointly used as the target endpoints.
3	Interruption or dose modification of some drugs	**Treatment policy strategy**: Continue to collect tumor imaging assessment data until the target endpoint is observed

Population-level summary: hazard ratio

3.2.1.2 Key secondary efficacy endpoint and estimand

Overall survival (OS): defined as the time from randomization to death due to any reason.

Population: Patients receiving first-line treatment for advanced non-squamous non-small cell lung cancer (NSCLC) who meet the inclusion and exclusion criteria

Treatment: HLX10/placebo + HLX04/placebo combined with chemotherapy (carboplatin-pemetrexed), with every 3 weeks (21 days) as a treatment cycle

Variable: Overall survival (OS)

Intercurrent events and handling strategies:

#	Intercurrent events	Handling strategies and description
1	Initiation of new anti-tumor therapy	**Treatment policy strategy**: Continue to collect survival information
2	Interruption or dose modification of some drugs	**Treatment policy strategy**: Continue to collect survival information
3	The chemotherapy group receives HLX10 + HLX04 after the occurrence of the first PD	**Treatment policy strategy**: Continue to collect survival information

Population-level summary: hazard ratio

3.2.1.3 Other secondary efficacy endpoints and estimands

(1) **Objective response rate (ORR)**: defined as the percentage of subjects whose best overall response is complete response (CR) or partial response (PR), assessed by the investigator and the IRRC as per RECIST v1.1, respectively.

Population: Patients receiving first-line treatment for advanced non-squamous non-small cell lung cancer (NSCLC) who meet the inclusion and exclusion criteria

Treatment: HLX10/placebo + HLX04/placebo combined with chemotherapy (carboplatin-pemetrexed), with every 3 weeks (21 days) as a treatment cycle

Variable: Percentage of subjects whose best overall response is CR/PR

Intercurrent events and handling strategies:

#	Intercurrent events	Handling strategies and description
1	Initiation of new anti-tumor therapy	**While on treatment strategy**: The outcome after the occurrence of the intercurrent event is considered to be unrelated to the study treatment, and only the data obtained before the occurrence of the intercurrent event is considered
2	Interruption or dose modification of some drugs	**Treatment policy strategy**: The intercurrent event is part of clinical practice. Continue to collect tumor assessment data

Population-level summary: odds ratio

(2) **Duration of response (DOR)**: defined as the time from the first documentation of response (CR or PR) to the first documentation of PD or death (whichever occurs first), assessed by the investigator and the IRRC as per RECIST v1.1, respectively.

Population: Patients receiving first-line treatment for advanced non-squamous non-small cell lung cancer (NSCLC) who meet the inclusion and exclusion criteria and have a best overall response of CR or PR

Treatment: HLX10/placebo + HLX04/placebo combined with chemotherapy (carboplatin-pemetrexed), with every 3 weeks (21 days) as a treatment cycle

Variable: Time from the first documentation of response (CR or PR) to the first documentation of PD or death (whichever occurs first)

Intercurrent event and handling strategy: Same as the PFS

Population-level summary: hazard ratio

3.2.2 Safety endpoints

- Adverse event

AE is defined as untoward medical occurrence in a patient or a subject of a clinical study administered a pharmaceutical product. AE does not necessarily have to have a causal relationship with the treatment. An AE may be worsening or more frequent occurrence of the pre-existing symptoms, signs, or laboratory abnormalities, a newly diagnosed disease, or abnormal laboratory finding, etc.

- Laboratory tests
    - Hematology (red blood cell count, hemoglobin, platelet, white blood cell count, differential white blood cell count and percentage (basophils, eosinophils, lymphocytes, monocytes, and neutrophils))
    - Serum chemistry (urea/urea nitrogen, creatinine, blood glucose, total bilirubin, direct bilirubin, alanine aminotransferase, aspartate aminotransferase, alkaline phosphatase, lactate dehydrogenase, total cholesterol, total protein, albumin, sodium, potassium, magnesium, chlorine, calcium, and phosphorus)
    - Urinalysis (urine specific gravity, urine pH, urine protein, urine glucose, urine ketones, urine occult blood, urine white blood cells, microscopic white blood cells, microscopic red blood cells, and 24 h urine protein)
    - Coagulation function (international normalized ratio and activated partial thromboplastin time)
    - Thyroid function (triiodothyronine, thyroxine, and thyroid-stimulating hormone)

- Virology (hepatitis B surface antigen, anti-HBs, hepatitis B E antigen, anti-HBe, anti-HBc, hepatitis C virus (HCV) antibody, HBV-DNA (optional), HCV-RNA (optional), and anti-HIV)
- Myocardial enzymes (creatine kinase and its isoenzyme, troponin, and brain natriuretic peptide)
- Pregnancy test

- Vital signs
  - Body temperature (°C)
  - Pulse (beats/min)
  - Respiratory rate (breaths/min)
  - Blood pressure (mmHg)
  - Body weight (kg)
- Physical examination
- Echocardiogram
- 12-Lead ECG
  - Heart rate (HR, beats/min)
  - QT interval (msec)
  - PR interval (msec)
  - QRS duration (msec)
  - QTc interval (msec)
- ECOG scores

3.2.3 Quality of life endpoints

- EQ-5D-5L
- EORTC QLQ-C30
- EORTC QLQ-LC13

3.2.4 Pharmacokinetics endpoints

Blood samples will be collected within 7 days pre-dose and within 2 h after the end of dosing in Cycle 1, within 3 days pre-dose in Cycles 2, 4, and 6, within 3 days pre-dose and within 2 h after the end of dosing in Cycle 8, within 3 days pre-dose every 4 cycles thereafter, at the end-of-treatment visit and/or safety follow-up to determine the serum concentration data of HLX10 and/or HLX04 in subjects.

3.2.5 Immunogenicity endpoints

- Anti-drug antibody (ADA)

3.2.6 Biomarker

- Programmed cell death-ligand 1 (PD-L1) expression level
- Microsatellite instability (MSI)
- Tumor mutation burden (TMB)

3.3 Sample size

Approximately 6–12 subjects are planned to be enrolled in the stage I of this study.

At stage II, PFS is the primary endpoint, and OS is the key secondary endpoint. The subjects will be randomized in a 1:1:1 ratio, and the sample size is based on the number of events required to demonstrate efficacy with regard to both PFS and OS. Assuming that the median PFS in the Group C is 6 months, and the hazard ratio (HR) of 0.69 in Group B, a total enrollment period of 24 months, the overall study period of 30 months, and the type I error rate $\alpha = 0.05$ (two-sided), at least 264 PFS events must be observed to obtain 85% power. Given a drop-out rate of 15%, a total of 400 subjects should be enrolled in the 2 groups (200 in each group).

A fixed-sequence testing method will be used to address the multiplicity of multiple group comparisons. If Group B is statistically different from Group C and the HR is < 1, the comparison between the Group A and the Group B will be continued; otherwise, the comparison will not be performed. Assuming that the median PFS in Group B is 8.7 months, the HR of 0.67 in Group A, and other parameters are the same as above, a total of 404 subjects should be enrolled in the 2 groups (202 in each group). In summary, about 606 subjects should be enrolled and at least 396 PFS events should be observed in Stage II.

For the key secondary endpoint OS, assuming that the median OS in the Group C is 10.7 months, the HR of 0.7 in Group B, and a group sequential design is adopted, the Lan-DeMets approximation to the O'Brien-Fleming boundary will be used to control the overall type I error rate $\alpha = 0.05$ (two-sided). Assuming a total enrollment period of 24 months and the overall study period of 46 months, at least 288 OS events must be observed to obtain 85% power.

Assuming that the median OS in Group B is 15.2 months, the HR of 0.68 in Group A, and other parameters and calculation methods are the same as above, and taking into account the potential dropout and that the number of events required and the number of subjects enrolled in Group A are the same as those in other treatment groups, about 630 subjects need to be enrolled and at least 432 OS events need to be observed in Stage II.

Considering the sample size required for PFS and OS evaluation, a total of 630 subjects (210 in each group) will be enrolled at stage II.

3.4 Statistical Hypothesis

The following hypothesis tests will be performed for the primary efficacy endpoint and key secondary efficacy endpoints:

The hypothesis for the probability of PFS in Group B (HLX10 combined with chemotherapy) and Group C (placebo combined with chemotherapy) is as follows:

$H_{10}: S_B(t) - S_C(t) = 0$; $H_{11}: S_B(t) - S_C(t) \neq 0$

The hypothesis for the probability of OS in Group B (HLX10 combined with chemotherapy) and Group C (placebo combined with chemotherapy) is as follows:

$H_{20}: S_B'(t) - S_C'(t) = 0$; $H_{21}: S_B'(t) - S_C'(t) \neq 0$

The hypothesis for the probability of PFS in Group A (HLX10 + HLX04 combined with chemotherapy) and Group B (HLX10 combined with chemotherapy) is as follows:

$H_{30}: S_A(t) - S_B(t) = 0$; $H_{31}: S_A(t) - S_B(t) \neq 0$

The hypothesis for the probability of OS in Group A (HLX10 + HLX04 combined with chemotherapy) and Group B (HLX10 combined with chemotherapy) is as follows:

$H_{40}: S_A'(t) - S_B'(t) = 0$; $H_{41}: S_A'(t) - S_B'(t) \neq 0$

Where $\mathrm{S(t)}$ represents the probability function of PFS and $\mathrm{S'(t)}$ represents the probability function of OS.

3.5 Randomization and Blinding

The second stage of this study is a three-arm, randomized, double-blind, multicenter, phase III clinical study. Eligible subjects are randomly allocated to the following 3 groups using an interactive web/voice response system (IWRS/IVRS) in a 1:1:1 ratio:

- **Group A (HLX10 + HLX04 combined with chemotherapy)**: HLX10 + HLX04 combined with chemotherapy (carboplatin-pemetrexed)

- **Group B (HLX10 combined with chemotherapy)**: HLX10 + HLX04 placebo combined with chemotherapy (carboplatin-pemetrexed)
- **Group C (placebo combined with chemotherapy)**: HLX10 placebo + HLX04 placebo combined with chemotherapy (carboplatin-pemetrexed)

Randomization is stratified by: PD-L1 expression level (negative, positive, or not evaluable), smoking history (yes versus no), and brain metastasis (yes versus no).

During the study, the subjects, the investigator, the sponsor, and the designees are not aware of the randomized allocation, with the exception of the need for emergency unblinding or the initiation of treatment after disease progression.

3.6 Safety Data Monitoring and Interim Analysis

An Independent Data Monitoring Committee (IDMC) will be established in this study to perform safety data monitoring once every 6 months and interim analysis at the time points specified in the protocol.

At stage II, PFS is the primary endpoint, and OS is the key secondary endpoint.

PFS will be analyzed twice, including a blinded sample size re-estimation and a final analysis.

OS will be analyzed for three times, including a blinded sample size re-estimation, the first interim efficacy analysis of OS during the final efficacy analysis of PFS, and the final analysis when reaching the expected number of OS events.

- The first interim analysis will be performed when about 420 subjects have been enrolled (approximately 2/3 of planned number of enrolled subjects), and its primary objective is to perform a blinded sample size re-estimation according to the actual PFS and OS data. This interim analysis will be performed in a blind state, without consuming the significance level a.
- Final analysis of PFS is planned to be conducted when the target number (about 396 PFS events) is observed. The significance level for the final analysis of PFS is 0.05 (two-sided).
- The first interim analysis of OS is planned to be performed with the final analysis of PFS, when the number of OS events is approximately 311 (72% of the total number of events). The estimated analysis time points and efficacy termination boundaries for the interim analysis and final analysis of OS in Group B (HLX10 combined with chemotherapy) and Group C (placebo combined with chemotherapy) are shown in Table 2, and the analysis time points and efficacy termination boundaries for the interim analysis and final analysis of OS in Group A (HLX10 + HLX04 combined with chemotherapy) and Group B (HLX10

combined with chemotherapy) are similar to those in Group B and Group C. Based on the actual number of OS events reached in Groups BC and AB at the analysis time points, the alpha significance level at the interim analysis will be adjusted using the Lan-DeMets approximation to the O'Brien-Fleming boundary.

- The final analysis of OS is planned to be performed when 432 OS events are collected. The estimated analysis time points and termination boundaries are shown in Table 2. Based on the actual number of OS events reached in Groups BC and AB at the analysis time points, the alpha significance level at the final analysis will be adjusted using the Lan-DeMets approximation to the O'Brien-Fleming boundary.

Table 2. Efficacy termination boundaries for the interim analysis and final analysis of OS in Group B (HLX10 combined with chemotherapy) and Group C (placebo combined with chemotherapy)

Analysis	Number of events (% information ratio)	Hazard ratio	Z value	p-value
Interim analysis	208 (72%) [planned]	0.7163	2.39751	0.0165
Final analysis	288 (100%) [planned]	0.7892	2.00449	0.04502

Note: [1] The analysis time points and efficacy termination boundaries for the interim analysis and final analysis of OS in Group A (HLX10 + HLX04 combined with chemotherapy) and Group B (HLX10 combined with chemotherapy) are similar to those in Group B and Group C.

[2] Based on the actual number of OS events reached in Groups BC and AB at the analysis time points, the alpha significance level at the interim analysis and final analysis will be adjusted using the Lan-DeMets approximation to the O'Brien-Fleming boundary.

A fixed-sequence test (the hypothesis tests are performed sequentially in the order of PFS in Group B (HLX10 combined with chemotherapy) and Group C (placebo combined with chemotherapy), OS in Group B and Group C, PFS in Group A (HLX10 + HLX04 combined with chemotherapy) and Group B (HLX10 combined with chemotherapy), OS in Group A and Group B) will be used to address multiplicity. Only when the null hypothesis is rejected for the previous hypothesis test, can the hypothesis test of the next endpoint be performed; if the null hypothesis is not rejected for the previous hypothesis test, the tests of all subsequent endpoints are stopped. At this time, the significance level of each hypothesis test is 0.05, and the overall type I error rate is still controlled within 0.05.

If the result of the interim analysis is positive, the sponsor may prematurely unblind and end the trial based on the IDMC's recommendation.

4 ANALYSIS POPULATIONS

4.1 Intention-To-Treat (ITT) Set

All subjects randomized into the study. ITT population will be considered as the primary analysis population for efficacy analysis in this study. The analysis of the ITT will be based on randomized groups.

4.2 Per Protocol Set (PPS)

As a subset of the ITT set, the per protocol set (PPS) consists of all randomized subjects who have received at least one post-treatment tumor assessment without any major protocol deviation that can significantly affect the primary efficacy. The analysis based on the PPS will serve as a support of ITT analyses. The population of the PPS will be determined in the blinded data review meeting prior to database lock.

4.3 Safety Set (SS)

All subjects who have received at least one dose of investigational product. The safety population will be the primary analysis population for safety assessment, and will be analyzed based on actual treatment groups.

4.4 Pharmacokinetic Set (PKS)

All subjects who have received at least one dose of HLX10 or HLX04 and have at least one post-dose concentration measurement at scheduled PK time points, without any major protocol deviations that can obviously affect the PK assessment. PKS will be used for PK analysis.

5 STATISTICAL METHODS

5.1 General Principles

5.1.1 Statistical analysis principles

(1) All statistical analyses and report of this study are in compliance with ICH E3 and E9 guidelines;

(2) Unless otherwise specified, the continuous variables will be summarized using descriptive statistics including non-missing number of subjects (N), arithmetic mean (Mean), standard deviation (SD), median, minimum (Min), and maximum (Max). The descriptive statistics for PK data will also include coefficient of variation (CV%), geometric mean, and geometric coefficient of variation (GCV%). The categorical or rank variables will be summarized using descriptive statistics including number and percentage of subjects and/or number of events, and the percentage will not be calculated if the number of subjects is 0.

Unless otherwise specified, the denominator of the calculated percentage is the total number of subjects in the corresponding group of the analysis population;

(3) For the statistical description of continuous variables, the minimum and maximum will be rounded to the same number of decimal places as the source data, the mean, geometric mean, and median will be rounded to 1 more decimal place than the source data, the standard deviation will be rounded to 2 more decimal places than the source data, the coefficient of variation and geometric coefficient of variation will be rounded to 1 decimal place, and the number of decimal places of descriptive statistics should not exceed 4. For the statistical description of categorical variables, the percentage will be rounded to 1 decimal place;

(4) Unless otherwise specified, all statistical tests in the study will be performed using the two-sided test, with a significance level of $\alpha = 0.05$. In general, in the statistical test, if the P value is greater than or equal to 0.0001, it will be rounded to 4 decimal places; if the P value is less than 0.0001, it will be expressed as "$P \leq 0.0001$"; if the P value is greater than 0.9999, it will be expressed as "$P \geq 0.9999$". The confidence interval (CI) will be rounded to 1 more decimal place than the point estimate;

(5) Due to the small number of subjects expected to be randomized in each study site and the large number of sites participating in the study, the study data of each study site will be pooled for summary analysis to obtain the overall estimate of the treatment efficacy of the investigational product. The "site" factor will not be considered in the statistical model or subgroup analysis;

(6) The changes in categorical data from baseline will be statistically summarized by shift tables. If there are untested and unassessed items, only the completed examinations and assessed visit results will be tabulated;

(7) Unscheduled assessments will only be presented in listings and not included in the statistical summary. If these examinations/assessments affect the baseline judgment, unscheduled examinations/assessments should be considered. If there are multiple test results for a laboratory parameter at a visit, except at baseline in the screening period, only the first valid test result will be included in the statistical summary, and other test results will be tabulated only. Invalid laboratory test results will not be included in the statistical summary but tabulated only;

(8) The safety/PK/efficacy data in the first stage (safety run-in phase) of this study will be presented in a separate listing. The efficacy event-time variables will be processed according to the rules of second stage, starting from the date of the first dose.

(9) If a subject is re-screened, only the results at the last screening will be analyzed.

(10) Unless otherwise specified, the data listings will be sorted by treatment group, subject number, visit, assessment date/time (if applicable), and parameter;

(11) The format of tables, figures, and listings will be specified in a separate TFL Mockup Shell file.

5.1.2 Data processing principles

5.1.2.1 Definition of time points

Unless otherwise specified, the baseline of this study is defined as the last accurate measurement obtained before the first dose (i.e., before the administration on the day of the first dose), and the change from baseline is defined as the difference between the measurement obtained at a scheduled examination time point and the baseline value. If the subject does not take the drug after randomization, the date of randomization will be used as the reference date for baseline definition.

The reference date is defined as the date of randomization, and the number of study days for an assessment or an event occurrence relative to the reference date is defined as follows:

- If the date of assessment or event is on or later than the reference date, then

Number of study days = Date of assessment/event occurrence − Reference date + 1

- If the date of assessment or event is earlier than the reference date, then

Number of study days = Date of assessment/event occurrence − Reference date

A time variable in "days" can be converted to a time variable in "months" as (12 × Day(s) / 365.25 or Day(s) / 30.4375), or to a time variable in "weeks" as (Day(s) / 7).

5.1.2.2 Handling of missing data

Unless otherwise specified, the missing data of efficacy, safety, quality of life, PK, and immunogenicity indicators will not be imputed in this study. For time-to-event efficacy data, the target event censoring rules are shown in Section 5.4.

Laboratory test values

For laboratory test indicators, the test values that are recorded as being below (and equal to) or above (and equal to) the test range value (e.g., $< x$, $\leq x$, $> x$, $\geq x$) should be handled as the test range value (i.e., $= x$) when summarizing the descriptive statistics; however, such values should still be listed as the results recorded in CRF when tabulating the data, i.e., "$< x$", "$\leq x$", "$> x$", or "$\geq x$".

For quantity of consumed cigarettes or alcohol consumption, the values that are recorded as being below (and equal to) or above (and equal to) the test range value (e.g., < x, ≤ x, > x, ≥ x) should be handled as the test range value (i.e., = x) when summarizing the descriptive statistics. If the test range value (e.g., xx–xx) is recorded, it should be handled as the mean range value when summarizing the descriptive statistics. The values should still be listed as the results recorded in CRF when tabulating the data, i.e., "xx–xx", "< x", "≤ x", "> x", or "≥ x".

For the missing date of an AE or concomitant medication/concomitant non-drug procedure, if it is necessary to compare it with the date of the first administration or the data analysis cut-off date (COD) (e.g., to determine whether it is an AE post-dose or concomitant medication), the missing date should be imputed as per the following methods before making a determination unless otherwise specified:

The missing date of an AE should be imputed as follows:

- Missing start date

If the start date of an AE is completely or partially missing, it will be assumed that the start date occurs as early as possible in the treatment period, unless other data can prove that the AE does not occur in the treatment period (e.g., the AE end date is earlier than the date of first dose).

1) If the year and month are known and the year and month are not the same as the year and month of the first dose, the missing date will be imputed with the first day of the known month (i.e., "01-xx-xxxx");
2) If the year and month are known and the year and month are the same as the year and month of the first dose, the AE start date will be set to be the same as the date of first dose (the date is "xx-xx-xxxx");
3) If only the year is known and the year is not the same as the year of the first dose, the missing date will be imputed with "01-01-xxxx";
4) If only the year is known and the year is the same as the year of the first dose, the AE start date will be set to be the same as the date of first dose (the date is "xx-xx-xxxx");
5) If the year, month, and day are all missing, the AE start date will be imputed with the date of first dose;
6) Other circumstances are considered as missing.

- Missing AE end date

If the end date of an AE is completely or partially missing, the end date should be estimated to maximize the duration of the AE. The imputation of end date is only applicable to AEs that are

not ongoing. AEs that are not ongoing include AEs with an outcome of "recovered/resolved without sequelae", "death", "recovered/resolved with sequelae", or "aggravating/worsening".

1) If the year and month are known, and the year and month are earlier than the data analysis cut-off date (e.g., database lock date), the missing date will be imputed with the last day of the known month (i.e., "28/29/30/31-xx-xxxx"); otherwise, the cut-off date will be used as the end date;

2) If only the year is known and the year is earlier than the cut-off date, the missing date will be imputed with the last day of that year (i.e., "31-12-xxxx"); otherwise, the cut-off date will be used as the end date;

   The minimum of the data analysis cut-off date (e.g., database lock date) and the imputed end date will be used for calculating the number of days of AEs;

3) If the imputed start date is later than the end date, the end date will be the corresponding start date;

4) If the year, month, and day are all missing, the data analysis cut-off date (e.g., database lock date) will be used as the AE end date.

5) If the end date of an AE with the outcome of "aggravating/worsening" is imputed, the imputed date should not be later than the start date of the same AE recorded subsequently (or imputed).

The missing date of concomitant medication/concomitant non-drug procedure should be imputed as follows:

- **Missing start date**

1) If only the year and month are known, the missing date will be imputed with the first day of the known month (i.e., "01-xx-xxxx");

2) If only the year is known, the missing date will be imputed with the first day of January of the known year (i.e., "01-01-xxxx");

3) If the year, month, and day are all missing, the date will not be imputed, and only the end date will be used to determine whether the date is before or after the first dose.

- **Missing end date**

If the end date of a concomitant medication is completely or partially missing, the end date should be estimated to maximize the duration of the concomitant medication/concomitant non-drug procedure. The imputation of end date is only applicable to concomitant medications/concomitant non-drug procedures that are not ongoing.

1) If the year and month are known, and the year and month are earlier than the data analysis cut-off date (e.g., database lock date), the missing date will be imputed with the last day of the known month (i.e., "28/29/30/31-xx-xxxx"); otherwise, the cut-off date will be used as the end date;

2) If only the year is known and the year is earlier than the cut-off date, the missing date will be imputed with the last day of that year (i.e., "31-12-xxxx"); otherwise, the cut-off date will be used for imputation. The minimum of the data analysis cut-off date (e.g., database lock date) and the imputed end date will be used for calculating the number of days of concomitant medication;

3) If the year, month, and day are all missing and the concomitant medication is not ongoing, the start date will be used as the end date;

4) If the imputed end date is later than the date of last known alive, the date of last known alive will be used as the corresponding end date.

The missing dates of primary diagnosis/diagnosis of stage IIIB/IIIC or stage IV/medical history, start and end of smoking history, and start and end of alcohol use should be imputed as follows:

1) If only the year is known, the missing date will be imputed with 1 Jan. (i.e., "01-01-xxxx");

2) If only the year and month are known, the missing date will be imputed with the first day (i.e., "01-xx-xxxx");

3) If the year, month, and day are all missing, the date will not be imputed.

No imputation will be performed for the other missing values. At data tabulation, the values are still listed as the original data collected.

5.1.2.3 Handling of outliers

Any outlier found will be checked. If necessary, it will be corrected or identified as a confirmed outlier after confirmation with the investigator.

During PK analysis, if there is obvious out-of-window PK blood sampling, dose interruption, or dose reduction resulting in significant abnormal PK concentrations, the data at the corresponding time points will not be included in the summary analysis.

5.1.2.4 Analysis/reporting software

Unless otherwise specified, all statistical analyses and reports will be completed using the statistical analysis system package (SAS Institute Inc., USA) version 9.4 or later. All reports will be provided in Microsoft Word document/RTF/PDF format.

5.1.3 Multiplicity comparison

This study involves two comparisons (Group B and Group C, Group A and Group B) among the three treatment groups, and the comparison between the two groups involves both interim analysis and final analysis.

This study adopts a group sequential design. The comparison between the two groups is performed using the Gate Keeping and Lan-DeMets approximation to the O'Brien-Fleming boundary to control the overall type I error α for the interim analysis and final analysis of the two groups to be below 0.05.

See "3.6 Safety Data Monitoring and Interim Analysis" for the specific α spending and the allocation of significance level.

5.2 Subjects

5.2.1 Subject disposition

The subject disposition will be described by treatment group and in total using the number and percentage of subjects, and will be plotted. The followings are mainly included:

- Number of subjects screened;
- Number and percentage of screen failure, and number and percentage of subjects for each screen failure reason;
- Number of subjects in the safety run-in phase of the Stage I single-arm study;
- The disposition of subjects in the Stage II phase III study is as follows:
  - Number and percentage of randomized subjects who have received at least one dose of the investigational product;
  - Number and percentage of subjects who have completed the treatment and prematurely discontinued the treatment, and number and percentage of subjects for each reason for premature treatment discontinuation;
  - Number and percentage of subjects who prematurely discontinued the study, and number and percentage of subjects for each reason for premature study discontinuation;
  - Number and percentage of subjects still in the follow-up of the study;
  - Number and percentage of subjects in each analysis population (ITT, PPS, SS, and PKS).

Subjects who have completed the treatment refer to the subjects who discontinue the treatment after PD confirmed by the IRRC, subject death, or subjects who discontinue the study treatment as assessed by the investigator after 2 years of treatment (35 cycles).

The primary reasons for screen failure, treatment discontinuation, end of study, and not being included in each analysis population will be tabulated by treatment group.

5.2.2 Major protocol deviations

The number and percentage of subjects with various categories of major protocol deviations will be described by treatment group and in total, including:

- Number and percentage of subjects with at least one major protocol deviation;
- Number and percentage of subjects in each major protocol deviation category.

Details of subjects' major protocol deviations will be tabulated by treatment group.

5.3 Demographics and Baseline Characteristics

Demographics and baseline characteristics will be statistically summarized and described based on the ITT. The information on demographics and baseline characteristics of subjects will be described by treatment group and in total.

5.3.1 Demographic data

The continuous variables will be summarized using descriptive statistics including non-missing number of subjects, arithmetic mean, standard deviation, median, minimum, and maximum, mainly including:

- Age (years)
- Height (cm)
- Body weight (kg)
- BMI (kg/m^2, calculated as: weight/$(height/100)^2$)
- Left ventricular ejection fraction (LVEF) by echocardiography and clinical significance of the examination result

According to the categories collected on the CRF, descriptive statistics including the number and percentage of subjects will be used for summary of categorical variables, mainly including:

- Sex
- Ethnicity

- ECOG performance scores
- PD-L1 expression level (positive vs. negative vs. not evaluable)
- Smoking history (yes vs. no)
- Brain metastasis (yes vs. no)

Detailed information on demographics of subjects will be tabulated by treatment group.

5.3.2 Baseline tumor diagnosis

The diagnosis and clinical stage of non-small cell lung cancer (NSCLC) of subjects at screening will be described by treatment group and in total.

The continuous variables will be summarized using descriptive statistics including non-missing number of subjects, arithmetic mean, standard deviation, median, minimum, and maximum, mainly including:

- Time from initial diagnosis of NSCLC (months, calculated as: (Date of informed consent − Date of initial diagnosis of NSCLC + 1)/30.4375)
- Time from diagnosis of stage IIIB/IIIC or stage IV NSCLC (months, calculated as: (Date of informed consent − Date of diagnosis of stage IIIB/IIIC or stage IV NSCLC + 1)/30.4375)
- Time of last pathological diagnosis (months, calculated as: (Date of informed consent − Date of last pathological diagnosis + 1)/30.4375)

The incomplete dates above will be handled according to the rules in "5.1.2.2 Handling of missing data".

According to the categories collected on the CRF, descriptive statistics including the number and percentage of subjects will be used for summary of categorical variables, mainly including:

- Pathological diagnosis method
- Pathological classification
- TNM classification (primary tumor (T), N regional lymph nodes, and M distant metastasis) at the time of informed consent
- Clinical stage
- Tumor status

Detailed information on NSCLC tumor diagnosis of subjects at screening will be tabulated by treatment group, including the date of initial diagnosis of NSCLC and the above indicators.

5.3.3 Medical history

The prior or existing medical conditions of subjects will be coded using the ICH Medical Dictionary for Regulatory Activities (MedDRA) V26.0. The number and percentage of subjects with prior or existing medical conditions in each system organ class (SOC) and preferred term (PT) will be summarized by treatment group and in total.

The presence/absence of medical history/existing medical conditions, name of disease/symptom, system organ class (SOC), preferred term (PT), start date, ongoing or not, and end date will be tabulated by treatment group.

5.3.4 Prior and concomitant medications

Prior and concomitant medications are defined as all prior and concomitant medications from 30 days before signing ICF to 90 days after the last study treatment. Concomitant medications associated with AEs are recorded up to 90 days after the last study treatment. Wherein:

- The medications with an end date before the first dose of the study treatment are defined as "prior medications";
- The medications with an end date after the first dose of the study treatment or with no recorded end date but recorded as ongoing until 90 days after the last study treatment are defined as "concomitant medications";

Prior and concomitant medications will be coded using the therapeutic classification (ATC-Level 2) and generic term in the World Health Organization Drug Dictionary (WHODrug-Global-B3-202303). The number and percentage of subjects who have received at least one prior or concomitant medication in the study period will be described by treatment group and in total, and the number and percentage of subjects who have received prior or concomitant medications will be summarized by therapeutic classification (ATC-Level 2) and generic term.

The presence/absence of prior/concomitant medications, preferred name, therapeutic classification (ATC-Level 2), generic term, indication, single dose, dose unit, dosing frequency, route of administration, start date, ongoing or not, and end date will be tabulated by treatment group, and marked as "prior medication" or "concomitant medication".

5.3.5 Prior and concomitant non-drug procedures

Prior and concomitant non-drug procedures are defined as all prior and concomitant non-drug procedures from 30 days before signing ICF to 90 days after the last study treatment. Concomitant non-drug procedures associated with AEs are recorded up to 90 days after the last study treatment. Wherein:

- The non-drug procedures with an end date before the first dose of the study treatment are defined as "prior non-drug procedures";
- The non-drug procedures with an end date after the first dose of the study treatment or with no recorded end date but recorded as ongoing until 90 days after the last study treatment are defined as "concomitant non-drug procedures";

The prior/concomitant non-drug procedures of subjects will be coded using the ICH Medical Dictionary for Regulatory Activities (MedDRA) V26.0.

The number and percentage of subjects with prior/concomitant non-drug procedures in each system organ class (SOC) and preferred term (PT) will be summarized by treatment group and in total. The presence/absence of prior/concomitant non-drug procedures, treatment name, system organ class (SOC), preferred term (PT), indication, start date, ongoing or not, and end date will be tabulated by treatment group.

5.3.6 History of smoking, alcohol use, allergies, and drug dependence

The continuous variables will be summarized using descriptive statistics including non-missing number of subjects, arithmetic mean, standard deviation, median, minimum, and maximum, mainly including:

- Smoking history
  - Duration of smoking history (months, calculated as: Min (end date (if quitted), date of informed consent (currently smoking) − start date + 1)/30.4375; the missing start/end date will be handled according to the rules in "5.1.2.2 Handling of missing data")
  - Quantity of consumed cigarettes (cigarette/day)
- Alcohol use history
  - Duration of alcohol use history (months, calculated as: Min (end date (quitted), date of informed consent (currently drinking) − start date + 1)/30.4375; the missing start/end date will be handled according to the rules in "5.1.2.2 Handling of missing data")
  - Alcohol consumption (mL/day)

According to the categories collected on the CRF, descriptive statistics including the number and percentage of subjects will be used for summary of categorical variables, mainly including:

- Smoking status
- Alcohol status

- Any histories of drug allergy
- Any histories of other allergies
- Drug dependence history

Smoking history, alcohol use history, allergy history, and drug dependence history of subjects will be tabulated by treatment group.

5.3.7 Prior anti-tumor therapy

5.3.7.1 Prior history of radiotherapy for NSCLC

According to the categories collected on the CRF, descriptive statistics including the number and percentage of subjects will be used for summary of categorical variables, mainly including:

- Presence/absence of prior radiotherapy for NSCLC
- Radiotherapy location

Detailed information on prior radiotherapy for NSCLC of subjects will be tabulated by treatment group, including presence/absence of prior radiotherapy for NSCLC, radiotherapy site, radiotherapy dose, dose unit, start date, and end date.

5.3.7.2 Prior history of surgery for NSCLC

According to the categories collected on the CRF, descriptive statistics including the number and percentage of subjects will be used for summary of categorical variables, mainly including:

- Presence/absence of prior surgery for NSCLC
- Surgery type

The name of prior surgery for NSCLC will be coded using the ICH Medical Dictionary for Regulatory Activities (MedDRA) V26.0. The number and percentage of subjects who have received prior surgery for NSCLC in each system organ class (SOC) and preferred term (PT) will be described by treatment group and in total. The presence/absence of prior surgery for NSCLC, surgery name, system organ class (SOC), preferred term (PT), surgery type, and date of surgery of each subject will be tabulated by treatment group.

5.3.7.3 Prior drug therapy for NSCLC

The continuous variables will be summarized using descriptive statistics including non-missing number of subjects, arithmetic mean, standard deviation, median, minimum, and maximum, mainly including:

- Duration of drug treatment (days; end date − start date + 1; the missing start date/end date will be handled according to the rules in "5.1.2.2 Handling of missing data")

According to the categories collected on the CRF, descriptive statistics including the number and percentage of subjects will be used for summary of categorical variables, mainly including:

- Presence/absence of prior drug therapy for NSCLC
- Treatment regimen type
- Treatment type
- Best response

The name of prior drug therapy for NSCLC will be coded using the Anatomical Therapeutic Chemical Classification System (ATC) in the World Health Organization Drug Dictionary (WHODrug-Global-B3-202303). The number and percentage of subjects who have received prior drug therapy for NSCLC will be summarized based on therapeutic classification (ATC-Level 2) and generic term by treatment group and in total.

The presence/absence of prior drug therapy for NSCLC, treatment regimen type, preferred name, therapeutic classification (ATC-Level 2), generic term, treatment type, dose, dose unit, dosing frequency, best overall response, start date, and end date will be tabulated by treatment group.

5.3.8 Biomarker and mutation tests at screening

The continuous variables will be summarized using descriptive statistics including non-missing number of subjects, arithmetic mean, standard deviation, median, minimum, and maximum, mainly including: tumor tissue biomarkers (CPS, TPS (%), IC Score (%), and TMB (mutations/Mb))

According to the categories collected on the CRF, descriptive statistics including the number and percentage of subjects will be used for summary of categorical variables, mainly including:

- Location of sample collection, PD-L1 ($\text{CPS} < 1$ vs. $1 \leq \text{CPS} < 10$ vs. $\text{CPS} \geq 10$）, TPS (TPS < 1%, 1% ≤ TPS <50%, TPS ≥ 50%), TMB (< 10 vs. ≥ 10), MSI (MSS vs. MSS/MSI-L vs. MSI-H)
- Mutation test (EGFR sensitive mutation, ALK gene rearrangement, and ROS1 gene rearrangement)

Detailed information on biomarker and mutation tests of subjects will be tabulated by treatment group.

For multiple PD-L1 samples, the handling rules are as follows:

- If a subject has multiple positive PD-L1 results but different sampling dates, the sample data closer to the date of randomization will be taken.
- If a subject has multiple positive PD-L1 results and the sampling dates are the same, the sample data with larger CPS, TPS, and IC values will be taken.

5.3.9 Other examinations at screening

The number and percentage of subjects with the following test results will be summarized by category, mainly including:

- Clinical significance of virology (HBV tests (HBsAg, HBsAb, HBeAg, HBeAb, and HBcAb), HCV antibody, and HIV)
- HBV DNA and HCV RNA will be tabulated only.

The virology test results and clinical significance of subjects will be tabulated by treatment group.

5.3.10 Treatment for NSCLC during the study

Treatment for NSCLC during the study includes anti-tumor treatment during the study treatment and subsequent anti-tumor treatment (including systemic therapy or non-systemic therapy) after the end of the study treatment.

5.3.10.1 Radiotherapy for NSCLC during the study

According to the categories collected on the CRF, descriptive statistics including the number and percentage of subjects will be used for summary of categorical variables, mainly including:

- Presence/absence of radiotherapy for NSCLC during the study
- Radiotherapy location

Detailed information on radiotherapy for NSCLC during the study will be tabulated by treatment group, including presence/absence of radiotherapy for NSCLC during the study, radiotherapy site, radiotherapy dose, dose unit, start date, and end date.

5.3.10.2 Surgery for NSCLC during the study

According to the categories collected on the CRF, descriptive statistics including the number and percentage of subjects will be used for summary of categorical variables, mainly including:

- Presence/absence of surgery for NSCLC during the study

- Surgery type

The name of surgery for NSCLC during the study will be coded using the ICH Medical Dictionary for Regulatory Activities (MedDRA) V26.0. The number and percentage of subjects who have received surgery for NSCLC during the study in each system organ class (SOC) and preferred term (PT) will be described by treatment group and in total. The presence/absence of surgery for NSCLC during the study, surgery name, system organ class (SOC), preferred term (PT), surgery type, and date of surgery of each subject will be tabulated by treatment group.

5.3.10.3 Drug therapy for NSCLC during the study

According to the categories collected on the CRF, descriptive statistics including the number and percentage of subjects will be used for summary of categorical variables, mainly including:

- Presence/absence of drug therapy for NSCLC during the study
- Treatment regimen type
- Treatment type

The names of drug therapy for NSCLC during the study will be coded using the Anatomical Therapeutic Chemical Classification System (ATC) in the World Health Organization Drug Dictionary (WHODrug-Global-B3-202303). The number and percentage of subjects who have received drug therapy for NSCLC during the study will be summarized based on therapeutic classification (ATC-Level 2) and generic term by treatment group and in total.

The presence/absence of drug therapy for NSCLC during the study, treatment regimen type, preferred name, therapeutic classification (ATC-Level 2), generic term, treatment type, dose, dose unit, dosing frequency, start date, ongoing or not, and end date will be tabulated by treatment group.

5.3.10.4 Subsequent systemic therapy for NSCLC

Subsequent systemic therapy for NSCLC, i.e., new anti-tumor therapy, is defined as other systemic chemotherapy, radiotherapy, hormone therapy, immunotherapy, biotherapy, or molecular targeted therapy with an anti-tumor effect approved by NMPA.

Subsequent systemic therapy for NSCLC will also be recorded on the page of radiotherapy, surgery, and drug therapy for NSCLC during the study in CRF, and subsequent systemic therapy for NSCLC will be determined by the medical and investigator based on the definition.

For subsequent systemic therapy for NSCLC, i.e., new anti-tumor therapy, if the start date is incomplete and only the day is missing, the day will be imputed with 1; if only the month and day are missing, the date will be imputed with 1 Jan.; if the year, month, and day are all missing,

the date will be imputed with the date of the first study dose. In addition, if there is clear evidence that the start date of the new anti-tumor therapy is after the end of study treatment, the end date of study treatment + 1 will be used for imputation (whichever occurs later).

The analysis method is the same as that for treatment for NSCLC during the study.

5.4 Efficacy Analysis

Stage I is the safety run-in phase of the single-arm study, and all efficacy results will be presented in listing. This section mainly describes the analytical methods for the randomized, double-blind, phase III study in stage II.

5.4.1 Analysis of primary efficacy endpoint

5.4.1.1 Primary endpoint and estimand

Progression-free survival (PFS) assessed by IRRC as per RECIST v1.1: defined as the time from randomization to the first documentation of PD or death due to any reason (whichever occurs first). PFS is calculated as follows: PFS (months) = (min (date of first documented PD, date of death) − date of randomization + 1)/30.4375. PFS will always be obtained based on the date of image scan instead of visit date.

Imaging data from subjects who re-signed the informed consent form after the first PD or started a new anti-tumor therapy will not be included in the analysis of primary endpoint.

Population: Patients receiving first-line treatment for advanced non-squamous non-small cell lung cancer (NSCLC) who meet the inclusion and exclusion criteria

Variable: Progression-free survival (PFS) assessed by IRRC as per RECIST v1.1

Treatment: HLX10/placebo + HLX04/placebo combined with chemotherapy (carboplatin-pemetrexed), with every 3 weeks (21 days) as a treatment cycle

Intercurrent events and handling strategies:

#	Intercurrent events	Handling strategies and description
1	Initiation of new anti-tumor therapy (subsequent systemic therapy for NSCLC) before PD or death[1]	**Hypothetical strategy**: Assuming that the risk of PD or death in subjects who start a new anti-tumor therapy is the same as that in subjects who do not start a new anti-tumor therapy, censor to the date of the last tumor imaging assessment before the start of new anti-tumor therapy.
2	Death	**Composite strategy**: Death and PD are jointly used as the target endpoints.
3	Interruption or dose modification of some drugs	**Treatment policy strategy**: Continue to collect tumor imaging assessment data until the target endpoint is observed

[1] New anti-tumor therapy (subsequent systemic therapy for NSCLC) is defined as other systemic chemotherapy, radiotherapy, hormone therapy, immunotherapy, biotherapy, or molecular targeted therapy with an anti-tumor effect approved by NMPA.

Population-level summary: hazard ratio

5.4.1.2 Main statistical analysis methods

The inter-group comparison of PFS will be performed using the stratified log-rank test and the randomization stratification factors are used as the stratification factors, i.e., PD-L1 expression level (negative, positive, and not evaluable), smoking history (yes vs. no), and brain metastasis (yes vs. no). The values of stratification factors collected by IWRS will be used in the analysis, with treatment group as the only fixed effect, and the two-sided *P*-value will be reported for the statistical results. The median and its 95% CI (Brookmeyer-Crowley method based on log-log transformation) will be estimated using the Kaplan-Meier method, and the Kaplan-Meier curve will be plotted. The HR and its 95% CI (Efron method) will be estimated using the stratified Cox proportional hazards model.

The censoring definition for the primary endpoint is shown in Table 3.

Table 3. Censoring rules for PFS

#	Circumstance	Date of event or censoring	Censored?
1	No baseline tumor assessment (regardless of death)	Date of randomization	Yes
2	No post-baseline tumor assessment and no death	Date of randomization	Yes
3	No post-baseline tumor assessment but died, no new anti-tumor therapy before death	Date of death	No
4	No post-baseline tumor assessment but died, receiving new anti-tumor therapy before death	Date of randomization	Yes
5	No PD or death before the end of follow-up (no new anti-tumor therapy)	Date of last tumor imaging assessment	Yes
6	No PD before the start of a new anti-tumor therapy, and PD or death after the start of a new anti-tumor therapy	Date of last tumor assessment before the use of new anti-tumor drug (including the same day)	Yes
7	PD or death during the study (including PD before the start of a new anti-tumor therapy, and missing a tumor assessment before PD or death)	Minimum of date of death and date of first PD	No

5.4.1.3 Sensitivity analysis

Sensitivity analysis 1: PD or death occurring after 2 or more consecutive missing tumor assessments or PD observed after unblinding is not considered as an event but censored to the date of the last tumor imaging assessment before the missing imaging or the date of the last tumor imaging assessment before unblinding, respectively.

The specific censoring rules are as follows:

Table 4. Censoring rules for PFS

#	Circumstance	Date of event or censoring	Censored?
1	No baseline tumor assessment (regardless of death)	Date of randomization	Yes
2	No post-baseline tumor assessment and no death	Date of randomization	Yes
3	No post-baseline tumor assessment but died, no new anti-tumor therapy before death, with the date of death no more than 98 days from the date of randomization	Date of death	No
4	No post-baseline tumor assessment but died, receiving new anti-tumor therapy before death	Date of randomization	Yes
5	No post-baseline tumor assessment but died, with the date of death more than 98 days from the date of randomization	Date of randomization	Yes
6	No PD or death before the end of follow-up (no new anti-tumor therapy)	Date of last tumor imaging assessment	Yes
7	No PD before the start of a new anti-tumor therapy, and PD or death after the start of a new anti-tumor therapy	Date of last tumor imaging assessment before the use of new anti-tumor drug (including the same day)	Yes
8	No PD before unblinding, with PD occurring after unblinding	Date of last tumor imaging assessment before unblinding (including the same day)	Yes
9	No PD before unblinding but died after unblinding, receiving a new anti-tumor therapy before death	Date of last tumor imaging assessment before the date of unblinding and new anti-tumor therapy (whichever occurs first) (including the same day)	Yes
10	No PD before unblinding but died after unblinding (no new anti-tumor therapy, with up to one tumor assessment missed before death)	Date of death	No
11	Missing two or more consecutive tumor assessments before PD or death (no new anti-tumor therapy). Missing two or more consecutive tumor assessments means that the interval between PD or death and the previous tumor assessment is more than 98 days (within 48 weeks) or 182 days (after 48 weeks). Note: 98 days = 12 weeks × 7 + 14 days; 182 days = 24 weeks × 7 + 14 days.	Date of last tumor imaging assessment before missing	Yes
12	PD or death during the study (including PD before the start of a new anti-tumor therapy, and missing a tumor assessment before PD or death)	Minimum of date of death and date of first PD	No

Sensitivity analysis 2: With actual stratification factors as adjustment variables for inter-group comparisons or statistical models, the robustness of efficacy outcomes is investigated with the presence of randomization stratification factors typo errors. The censoring rules for PFS are the same as those in the primary analysis.

Sensitivity analysis 3: Not considering the stratification factors in inter-group comparison or statistical models. The censoring rules for PFS are the same as those in the primary analysis.

Sensitivity analysis 4: The PFS assessed by the investigator as per RECIST v1.1 will be used for sensitivity analysis to evaluate the robustness of the IRRC assessment results. The censoring rules for PFS are the same as those in the primary analysis.

Sensitivity analysis 5: The PFS assessed by the investigator as per RECIST v1.1 will be used for sensitivity analysis to evaluate the robustness of the IRRC assessment results. The censoring rules for PFS are the same as those in the sensitivity analysis 1.

5.4.1.4 Supplementary analysis

Supplementary analysis 1: The supplementary analysis will be performed based on the PPS. For the efficacy results assessed by the IRRC and the investigator as per RECIST v1.1, the definitions of PFS event and censoring rules are the same as those for the main statistical analysis methods.

5.4.2 Analysis of key secondary efficacy endpoint

5.4.2.1 Key secondary endpoint and estimand

The key secondary efficacy endpoint of this study is OS, defined as the time from randomization to death due to any reason, calculated as follows: OS (months) = (date of death – date of randomization + 1)/30.4375.

Population: Patients receiving first-line treatment for advanced non-squamous non-small cell lung cancer (NSCLC) who meet the inclusion and exclusion criteria

Variable: Overall survival (OS)

Treatment: HLX10/placebo + HLX04/placebo combined with chemotherapy (carboplatin-pemetrexed), with every 3 weeks (21 days) as a treatment cycle. After the occurrence of the first PD, determine whether to continue the treatment or modify the treatment regimen until the study is discontinued.

Intercurrent events and handling strategies:

#	**Intercurrent events**	**Handling strategies and description**
1	Initiation of new anti-tumor therapy	**Treatment policy strategy**: Continue to collect survival information
2	Interruption or dose modification of some drugs	**Treatment policy strategy**: Continue to collect survival information
3	The chemotherapy group receives HLX10 + HLX04 after the occurrence of the first PD	**Treatment policy strategy**: Continue to collect survival information

Population-level summary: hazard ratio

5.4.2.2 Main statistical analysis methods

Based on the ITT, the statistical analysis method for OS as the endpoint is the same as that for the primary efficacy endpoint.

If the year and month of death are available but the day is missing, the date of death will be imputed with the first day of the month or the date of last known survival, whichever is later. If both month and day of the date of death are missing, the date of death will be imputed with 1 Jan. or the date of last known survival, whichever is later. If the date of death is completely missing, it will be imputed with the date of last known survival.

For the date of last known survival of a subject, select the latest known survival date of the subject in the EDC data. If the date of last known survival is incomplete or later than the cut-off date, proceed as follows:

1) If only the year is known, the date will be imputed with 1 Jan. (i.e., "01-01-xxxx");
2) If only the year and month are known, the missing date will be imputed with the first day (i.e., "01-xx-xxxx");
3) If the date of last known survival is later than the cutoff date, the cutoff date will be used as the latest date of last known survival.

The censoring definition for OS is shown in Table 5.

Table 5. Censoring rules for OS

	Event	Handling instruction	Censored?
1	Loss to follow-up	Date of last known survival	Yes
2	Death	Date of death	No

5.4.2.3 Sensitivity analysis

Sensitivity analysis 1: Group C (placebo combined with chemotherapy) receives HLX10 + HLX04 after PD. The rank-preserving structural failure time model (RPSFTM) method is used to adjust the deviation by treatment switching.

Sensitivity analysis 2: Group C receives HLX10 + HLX04 or other PD-1 drugs or other drugs in the same class after PD. The rank-preserving structural failure time model (RPSFTM) method is used to adjust the deviation by treatment switching.

Sensitivity analysis 3: With actual stratification factors as adjustment variables for inter-group comparisons or statistical models, the robustness of efficacy outcomes is investigated with the presence of randomization stratification factors typo errors.

Sensitivity analysis 4: Not considering the stratification factors in inter-group comparison or statistical models.

5.4.2.4 Supplementary analysis

Supplementary analysis 1: The supplementary analysis will be performed based on the PPS. The definitions of OS event and censoring rules are the same as those for the main statistical analysis methods.

5.4.3 Secondary efficacy endpoints

The analyses of secondary efficacy endpoints will be performed based on the ITT. The secondary efficacy endpoints of this study include the following.

5.4.3.1 Objective response rate (ORR)

Objective response rate (ORR) is defined as the percentage of subjects whose best overall response (BOR) is complete response (CR) or partial response (PR). Subjects without post-baseline tumor assessments will be considered non-responders.

BOR is defined as the best result of overall tumor response assessment during study treatment (respectively assessed by the IRRC and the investigator as per RECIST v1.1) as per the following priority levels: 1. complete response (CR) > 2. partial response (PR) > 3. stable disease (SD) > 4. progressive disease (PD) > 5. not evaluable (NE). The interval between the date of randomization and SD should be no less than 6 weeks (42 days). If the time from randomization to the best overall response of SD is less than 6 weeks, it will not be counted as SD.

Unconfirmed ORR and confirmed ORR will be analyzed separately, and subjects with confirmed BOR of complete response (CR) or partial response (PR) must be re-assessed at least 4 weeks later for confirmation.

Population: Patients receiving first-line treatment for advanced non-squamous non-small cell lung cancer (NSCLC) who meet the inclusion and exclusion criteria

Treatment: HLX10/placebo + HLX04/placebo combined with chemotherapy (carboplatin-pemetrexed), with every 3 weeks (21 days) as a treatment cycle

Variable: Whether the best overall response reaches CR/PR

Intercurrent events and handling strategies:

#	**Intercurrent events**	**Handling strategies and description**
1	Initiation of new anti-tumor therapy	**While on treatment strategy**: The outcome after the occurrence of the intercurrent event is considered to be unrelated to the study treatment, and only the data obtained before the occurrence of the intercurrent event is considered
2	Interruption or dose modification of some drugs	**Treatment policy strategy**: The intercurrent event is part of clinical practice. Continue to collect tumor assessment data

Population-level summary: response rate

Statistical analysis methods:

For unconfirmed ORR and confirmed ORR, the stratified Cochran-Mantel-Haenszel (CMH) method will be used to test the difference in ORR between the two groups, and the odds ratio and its 95% CI will be estimated. The stratification factors are the same as the randomization stratification factors, i.e., PD-L1 expression level (negative, positive, and not evaluable), smoking history (yes vs. no), and brain metastasis (yes vs. no). 95% CI in an individual treatment group is calculated using the Clopper-Pearson method. The number and percentage of subjects who achieve best overall response as assessed by the IRRC and the investigator will be summarized.

Sensitivity analysis:

Sensitivity analysis 1: Not considering the stratification factors in inter-group comparison or statistical models.

Supplementary analysis:

Supplementary analysis 1: The supplementary analysis will be performed based on the PPS.

5.4.3.2 Duration of response (DOR)

Respectively assessed by the IRRC and the investigator as per RECIST v1.1, defined as the time from the first documentation of response (CR or PR) to the first documentation of PD or death (whichever occurs first). Unconfirmed DOR and confirmed DOR will be analyzed separately, and subjects with confirmed DOR who have achieved complete response (CR) or partial response (PR) must be re-assessed at least 4 weeks later for confirmation.

Population: Patients receiving first-line treatment for advanced non-squamous non-small cell lung cancer (NSCLC) who meet the inclusion and exclusion criteria and have a best overall response of CR or PR

Treatment: HLX10/placebo + HLX04/placebo combined with chemotherapy (carboplatin-pemetrexed), with every 3 weeks (21 days) as a treatment cycle

Variable: Time from the first documentation of response (CR or PR) to the first documentation of PD or death (whichever occurs first)

Intercurrent event and handling strategy: Same as the PFS

Population-level summary: hazard ratio

Statistical analysis methods:

The DOR will be analyzed only for subjects whose best overall response is evaluated as CR or PR. The median and its 95% CI will be estimated using the Kaplan-Meier method, and the Kaplan-Meier curve will be plotted. Censoring rules for DOR are shown in Table 6 below.

Table 6. Censoring rules for DOR

#	Parameter	Circumstance	Date of event or censoring	Censored
1	DOR	No PD or death after the first documented CR or PR and before the end of follow-up	Date of last tumor imaging assessment	Yes
2	DOR	No tumor assessment and no death after the first documented CR or PR	Date of first confirmed CR or PR	Yes
3	DOR	Initiation of new anti-tumor therapy after the first documented CR or PR and before PD or death	Date of last tumor imaging assessment before subsequent anti-tumor treatment	Yes
4	DOR	PD or death after the first documented CR or PR	Minimum of date of death and date of first PD	No

Supplementary analysis:

Supplementary analysis 1: The supplementary analysis will be performed based on the PPS. The definitions of DOR event and censoring rules are the same as those for the main statistical analysis methods.

5.4.4 Subgroup analysis

Subgroup analyses include the subgroups defined by stratification factors and the subgroups defined by biomarker (only the number of subjects and events will be summarized if the percentage of a subgroup population is less than 5% of the ITT population). The subgroup analysis results will be used to draw forest plots. Subgroup analyses will be based on the efficacy results assessed by the IRRC, and the analysis set will be based on the ITT. The planned subgroup analyses include:

- Age: < 65 vs. ≥ 65 years

- ECOG performance score: 0 vs. 1
- Sex: male vs. female
- Smoking history: yes vs. no
- Brain metastasis: yes vs. no
- PD-L1 expression level: negative vs. positive vs. not evaluable
- PD-L1 expression level: $\text{CPS} < 1$ vs. $1 \leq \text{CPS} \leq 9$ vs. $\text{CPS} \geq 10$
- PD-L1 expression level: $\text{TPS} < 1\%$ vs. $1\% \leq \text{TPS} < 50\%$ vs. $\text{TPS} \geq 50\%$
- Tumor status: locally advanced (stage IIIB/IIIC) vs. distant metastasis (stage IV)
- MSI: MSS/MSI-Low vs. MSI-High
- TMB: $<$ 10 muts/Mb vs. $\geq$ 10 muts/Mb

5.4.5 Other analyses

The median follow-up time and its 95% CI will be calculated using the Reverse Kaplan-Meier method. Censoring rules for median follow-up time are shown in Table 7 below.

Table 7. Censoring rules for median follow-up time

#	Parameter	Circumstance	Date of event or censoring	Censored
1	Median follow-up time	No death by the end of study/analysis cut-off date/dropout	Date of event occurrence: Date of end of study/analysis cut-off/dropout	No
2	Median follow-up time	Death	Date of death	Yes

The sum of diameters of target lesions at baseline and post-baseline visits, and the differences between results at each post-baseline visit and baseline will be described using non-missing number of subjects, arithmetic mean, standard deviation, median, minimum, and maximum by treatment group. A waterfall plot will be generated to illustrate the best change from baseline in the sum of target lesion diameters, based on the best overall response (BOR).

The post-treatment tumor imaging results assessed by the IRRC will be tabulated, including subject number, visit, visit date, overall tumor response assessment, BOR, ORR (objective response), date of first dose, date of first confirmed CR/PR, date of first PD or death/censoring, etc.

The post-treatment tumor imaging results assessed by the investigator will be tabulated, including target lesions, non-target lesions, and new lesions at each visit, overall response

assessment result, BOR, ORR (objective response), date of first dose, date of first confirmed CR/PR, date of first PD or death/censoring, etc.

The information on target lesion number, lesion site, site description, assessment or not, visit, examination date, assessment method, sum of diameters (mm), change from baseline, etc.; information on non-target lesion number, lesion site, site description, assessment or not, visit, examination date, assessment method, non-target lesion results, etc.; and information on presence of new lesions, new lesion number, lesion site, site description, visit, examination date, assessment method, results, etc. will be tabulated by treatment group and subject, respectively.

5.5 Safety Analysis

5.5.1 Drug exposure and compliance

The drug exposure and compliance analysis will be based on the SS. The theoretical and actual doses of study medications (HLX10, HLX04, Pemetrexed, and Carboplatin), modification or not, discontinuation or not, and compliance information of subjects will be described by cycle and treatment group.

The compliance in each cycle is defined as: actual total dose/theoretical total dose × 100%. The actual total dose is the sum of actual doses, and the planned total dose is the sum of theoretical doses.

According to the categories collected on the CRF, descriptive statistics including the number and percentage of subjects will be used for summary of categorical variables, mainly including:

- Number and percentage of subjects for dose modification or not and each primary reason for dose modification
- Number and percentage of subjects for infusion interruption or not and each primary reason for infusion interruption
- Classification of drug compliance in each cycle (< 80%, 80%–120%, > 120%)
- Continued treatment or not after PD

In addition, the descriptive statistics of drug exposure intensity will be provided, and the total treatment days, number of treatment cycles, theoretical/actual total dose, planned dose intensity, mean dose intensity, relative dose intensity, etc. of the investigational product will be summarized.

The exposure indices are defined as follows:

- **Total treatment days** is defined as the duration from the date of first dose to the date of last dose. Actual duration of exposure (weeks) is calculated as (date of last dose − date of first dose + 1)
- **Actual total dose (mg)** is defined as the sum of all actual doses administered from the date of first dose to the date of last dose.
- **Total theoretical dose (mg)** is defined as the sum of all planned doses from the date of first dose to the date of last planned dose. The planned HLX10 dose will be collected in the EDC system.
- **Theoretical/Planned dose intensity (mg/week)** = total **theoretical** dose (mg)/total treatment days/7
- **Mean dose intensity (mg/week)** = actual total dose (mg)/total treatment days/7
- **Relative dose intensity (%)** is defined as mean dose intensity (mg/week)/planned dose intensity (mg/week) × 100%

The drug administration of each subject in each cycle will be tabulated by treatment group, including treatment group, administration name, subject number, cycle, body weight category, whether drug is administered and reason for not administering drug, theoretical dose, actual dose, start date and time of infusion, end date and time of infusion, dose modification and reason, dose interruption and reason, and compliance in each cycle.

The exposure to drug infusion of each subject will be tabulated by treatment group, including subject number, date of first infusion, date of last infusion, treatment days, total number of infusions, compliance in treatment period (%), and classification (< 80%, 80%–120%, > 120%).

5.5.2 Adverse event

AEs will be statistically summarized and described based on the SS. Since subjects in the control group will receive HLX10 + HLX04 after the occurrence of first PD and signing of ICF, the summary of AEs will be presented by the following four groups.

- HLX10 + HLX04 combined with chemotherapy group: including all subjects randomized to the HLX10 + HLX04 combined with chemotherapy group.
- HLX10 combined with chemotherapy group: including all subjects randomized to the HLX10 combined with chemotherapy group.
- Placebo combined with chemotherapy group: including all subjects randomized to the control group (placebo combined with chemotherapy), but only including data without any group switching or pre-switching data.

- Placebo combined with chemotherapy group switching to HLX10 + HLX04 group: including subjects randomized to the control group and then switched to the HLX10 + HLX04 group, and only including data after switching.

AEs in the first stage will be presented in listings.

5.5.2.1 Definition of adverse event

AE is defined as untoward medical occurrence in a patient or a subject of a clinical study administered a pharmaceutical product. AE does not necessarily have to have a causal relationship with the treatment. An AE may be worsening or more frequent occurrence of the pre-existing symptoms, signs, or laboratory abnormalities, a newly diagnosed disease, or abnormal laboratory finding, etc.

In this study, all AEs that occur from the signing of ICF to 90 days after the last dose of the investigational product (HLX10/HLX04) or the initiation of a new anti-tumor therapy (whichever occurs first) will be collected.

AEs and SAEs should be assessed and graded based on National Cancer Institute Common Terminology Criteria for Adverse Events (NCI-CTCAE) v5.0. The severity is as follows: (1) mild, (2) moderate, (3) severe or medically significant but not immediately life-threatening, (4) life-threatening consequences, and (5) AE-related death.

Adverse events of special interest (AESIs) are investigational product related events attracting scientific and medical interests, which can be serious or non-serious adverse event. AESIs in this study include infusion related reaction (IRR) and other immune-related adverse events (irAEs).

SAE is defined as any untoward medical occurrence at any dose during a clinical trial and meets any one or more of the following criteria: 1) results in death; 2) life-threatening; 3) leading to hospitalization or prolonged hospitalization; 4) leading to permanent or significant disability/incapacity; 5) leading to any congenital anomaly/birth defect; 6) other important medical events.

Treatment-related is defined as that the causality between an AE and the investigational product in this study is "related", "possibly related", or "unknown", or the relationship record is missing.

AEs during the screening period and TEAEs: The analysis of AEs will be based on the treatment-emergent adverse events (TEAEs). The relevant definitions are as follows:

- AEs that occur after the signing of ICF and before the first dose of study treatment are defined as "AEs during the screening period";

- AEs that occur from the date of the first dose of study treatment to 90 days after the last dose or initiation of a new anti-tumor therapy (whichever occurs first) are defined as "treatment-emergent adverse events" (TEAEs). AEs that occur before the first dose of study treatment but worsen after the first dose are also recorded as TEAEs. In addition, SAEs related to HLX10 or HLX04 that occur after the initiation of a new anti-tumor therapy are also considered as TEAEs.

5.5.2.2 Analysis of adverse events

AEs will be coded using the ICH Medical Dictionary for Regulatory Activities (MedDRA) V26.0.

The number of subjects who experienced AEs, incidence, and number of AEs will be described by treatment group. The following events are mainly included:

Throughout the study:

- All AEs

TEAE:

- TEAE
- TEAEs related to the investigational product (HLX04/placebo, HLX10/placebo, pemetrexed/carboplatin)
- CTCAE Grade ≥ 3 TEAEs
- CTCAE Grade ≥ 3 TEAEs related to the investigational product (HLX04/placebo, HLX10/placebo, pemetrexed/carboplatin)
- SAEs in TEAEs
- Serious TEAEs related to the investigational product (HLX04/placebo, HLX10/placebo, pemetrexed/carboplatin)
- TEAEs leading to death
- TEAEs leading to death related to the investigational product (HLX04/placebo, HLX10/placebo, pemetrexed/carboplatin)
- TEAEs leading to interruption of study medication (HLX04/placebo, HLX10/placebo, pemetrexed/carboplatin)
- TEAEs leading to dose modification of HLX04 or chemotherapy drug

- TEAEs leading to permanent discontinuation of study medication (HLX04/placebo, HLX10/placebo, pemetrexed/carboplatin)
- AESIs including immune-related adverse events (irAEs) and infusion reactions
- Serious AESIs including immune-related serious adverse events (irAEs) and serious infusion reactions

Meanwhile, the above TEAEs will be summarized by treatment group using number of subjects with TEAEs and incidence based on the system organ class (SOC) and preferred term (PT).

The number of subjects with the maximum CTCAE grade TEAEs, incidence, and number of TEAEs will be summarized by treatment group and in total based on the system organ class (SOC) and preferred term (PT). The following events are mainly included:

- All TEAEs
- All drug-related TEAEs

The analysis of all AEs complies with the following general principles:

(1) The number of events will not be presented as a percentage;

(2) When statistically describing the number of subjects and incidence, if a subject experiences more than one AE under a specific system organ class (SOC) and preferred term (PT), then only 1 subject is counted under that class, and the actual number of occurrences will be counted for the calculation of number of events;

(3) When TEAEs are summarized by the greatest CTCAE grade by SOC and PT, only the AE with the greatest CTCAE grade is included when the subject has experienced the same AE multiple times;

(4) When TEAEs are summarized by their correlation to the investigational product by SOC and PT, only the AE with the closest correlation to the investigational product is included in the statistical summary when the subject has experienced the same AE multiple times;

(5) AEs with an incidence greater than a certain percentage will be selected according to any treatment group and any system organ class/preferred term (SOC/PT).

(6) Dose modification is defined as the action taken on the investigational product: "dose reduced" or "dose interrupted with dose reduced ".

The AEs, system organ class (SOC), preferred term (PT), TEAE or not, AESI or not, immune-related or not, infusion reaction or not, cycle of occurrence, start date, ongoing or not, end date, outcome, CTCAE grade, relationship with each study medication, action taken on each

study medication, SAE or not and type of SAE, action taken will be tabulated by treatment group. The drug-related AEs, SAEs, drug-related SAEs, and AEs leading to death will also be tabulated in the same manner.

A separate listing is provided to describe subjects who died. The date of death, primary cause of death, and other relevant information for deceased subjects are listed by treatment group.

5.5.3 Laboratory tests

Laboratory tests will be statistically described based on the SS. The laboratory test results of subjects will be described by treatment group and in total.

For continuous variables, descriptive statistics including non-missing number of subjects, arithmetic mean, standard deviation, median, minimum, and maximum will be used for summary of the laboratory test results at baseline and post-baseline visits, and the difference between each post-baseline visit and baseline. According to the categories collected on the CRF, descriptive statistics including the number and percentage of subjects will be used for summary of categorical variables.

Laboratory tests mainly include: hematology, serum chemistry, urinalysis, coagulation function, thyroid function, virology, myocardial enzymes, and pregnancy test.

The number and percentage of subjects with clinically significant changes in all laboratory test results from baseline to each post-baseline visit will be summarized using shift tables. For categorical variables, a shift table will be used to summarize the data based on categories.

Laboratory test results will be tabulated by parameter, treatment group, subject, and visit, including test results, reference range, clinical significance, changes from baseline, and CTCAE grade (if applicable). In the data listing, values exceeding the clinical reference range will be marked, with values below the lower limit of the clinical reference range marked as 'L' and values above the upper limit marked as 'H'.

The pregnancy test results will be tabulated separately.

5.5.4 Vital signs

Vital signs will be statistically described based on the SS. The body temperature (°C), pulse (beats/min), respiratory rate (breaths/min), systolic blood pressure (mmHg), diastolic blood pressure (mmHg), body weight (kg), and BMI (kg/m^2) of subjects will be described by treatment group and in total.

Descriptive statistics including non-missing number of subjects, arithmetic mean, standard deviation, median, minimum, and maximum will be used for summary of the vital signs

measurement results at baseline and post-baseline visits, and the difference between each post-baseline visit and baseline.

Detailed information on vital signs measurement results will be tabulated by treatment group.

5.5.5 Physical examination

Physical examinations will be statistically described based on the SS. The physical examination results of subjects will be described by treatment group and in total, including head and neck (including thyroid gland), chest (including heart and lung), abdomen (liver, gallbladder, spleen, and kidney), limbs, skin, lymph nodes, nervous system, as well as general conditions of subjects.

The number and percentage of subjects with clinically significant changes in physical examinations from baseline to each post-baseline visit will be summarized using shift tables.

The physical examination results of subjects at each visit will be tabulated by treatment group.

5.5.6 12-Lead ECG

The 12-lead ECG results will be statistically described based on the SS. The 12-lead ECG results of subjects will be described by treatment group and in total, including heart rate (beats/min), QT interval (msec), PR interval (msec), QRS time (msec), QTc interval (msec), and QTcF interval (msec).

Descriptive statistics including non-missing number of subjects, arithmetic mean, standard deviation, median, minimum, and maximum will be used for summary of the 12-lead ECG results at baseline and post-baseline visits, and the difference between each post-baseline visit and baseline. The number and percentage of subjects with clinically significant changes in 12-lead ECG results from baseline to each post-baseline visit will be summarized using shift tables.

The results of the 12-lead ECG examination and the clinical significance at each visit will be tabulated and described by treatment group.

5.5.7 ECOG performance scores

ECOG performance scores will be statistically described based on the SS.

Descriptive statistics including number and percentage of subjects will be used for summary of ECOG scores at baseline and post-baseline visits. The number and percentage of subjects with changes in ECOG from baseline to each post-baseline visit will be summarized using shift tables.

Detailed information on ECOG scores of subjects at each visit will be tabulated by treatment group.

5.6 Immunogenicity Analysis

The immunogenicity results will be statistically described based on the SS. The immunogenicity of HLX10 and/or HLX04 will be summarized and analyzed based on the actual treatment received by the subjects.

The number and percentage of subjects who are positive for anti-HLX10 antibody, anti-HLX04 antibody (ADA), and neutralizing antibody (NAb) as well as the number and percentage of subjects with at least one ADA/NAb positive sample after administration of HLX10 and/or HLX04 at each visit will be summarized by treatment group and in total.

The administration time, ADA sampling date, and test results of each subject at each visit will be tabulated by treatment group.

5.7 Pharmacokinetic Analysis

In addition to the analysis of serum concentrations of HLX10 and HLX04, the PK parameters of the two drugs in Cycles 1 and 8 will also be analyzed. The PK parameters include:

- Maximum concentration (C_{max}): the concentration within 2 h after the end of administration in Cycles 1 and 8.
- Trough concentration (C_{trough}): the concentration within 3 days before administration in Cycles 2 and 8.
- Accumulation ratio (R_{ac_Cmax}): the ratio of C_{max} after administration in Cycle 8 to that after administration in Cycle 1 (rounded to 3 decimal places)
- Accumulation ratio ($R_{ac\_Ctrough}$): the ratio of C_{trough} before administration in Cycle 8 to that before administration in Cycle 2 (rounded to 3 decimal places)

The serum drug concentrations and PK parameters will be statistically summarized based on the PKS. Concentration data below lower limit of quantification (BLQ) will be included in the statistical summary, parameter calculation, and plotting as 0.

The serum concentrations of HLX10 and HLX04 in the subjects at each time point will be summarized and described using the non-missing number of subjects, number of subjects with BLQ concentrations, arithmetic mean, standard deviation, coefficient of variation (CV%), geometric mean, geometric coefficient of variation (GCV%), median, minimum, and maximum by treatment group and planned sampling time point. If there are less than 3 valid data at a time point, only non-missing number of subjects, number of subjects with BLQ concentrations, maximum, and minimum will be presented, and the remaining statistics will be marked as NC (not calculated).

The mean serum concentration-time curves and individual serum concentration-time curves of HLX10 and HLX04 will be plotted by treatment group according to the planned sampling time points.

The PK parameters of HLX10 and HLX04, including C1-C_{max}, C2-C_{trough}, C8-C_{max}, C8-C_{trough}, R_{ac_Cmax}, and $R_{ac\_Ctrough}$, will be summarized and described by treatment group using non-missing number of subjects, arithmetic mean, standard deviation, coefficient of variation (CV%), geometric mean, geometric coefficient of variation, median, minimum, and maximum. If there are less than 3 valid data for a treatment group, only non-missing number of subjects, maximum, and minimum will be presented, and the remaining statistics will be marked as NC (not calculated).

The administration time, planned sampling time points, actual sampling time points, and serum concentrations of HLX10 and HLX04 of the subjects at each visit will be tabulated by treatment group. The PK parameters of subjects will be tabulated by treatment group.

If data allow, the PK concentrations and parameters will be summarized and analyzed according to the ADA/NAb test results of subjects. Subjects with at least one ADA/NAb positive sample after administration will be considered ADA/NAb positive.

5.8 Quality of Life Analysis

The quality of life analysis will be based on the ITT, mainly including quality of life assessments using the EQ-5D-5L, EORTC QLQ-C30 and EORTC QLQ-LC13.

The observed values of total score, sub-score, and individual score at each visit and the changes from the baseline will be described statistically using scale corresponding score summary rules based on the treatment groups. For continuous variables, descriptive statistics including non-missing number of subjects, arithmetic mean, standard deviation, median, minimum, and maximum will be used for summary of the scale assessment results at baseline and post-baseline visits, and the difference between each post-baseline visit and baseline. Categorical variables will be statistically described using the number and percentage of subjects based on the sub-items of the scale.

Subjects' quality of life scale results will be tabulated by treatment group, including the results of each item of the scale and the summary of scores of each dimension.

5.8.1 EQ-5D-5L

The EQ-5D-5L scale consists of 5 dimensions (mobility, self-care, daily activities, pain or discomfort, anxiety or depression) and a 100-point EQ-5D visual analogue scale (EQ VAS), each containing 5 levels (no problems, slight problems, moderate problems, severe problems, and

extreme problems). The percentage of each item score will be summarized by visit and dimension, and the VAS score will be summarized by visit as continuous variable. The scores of each dimension will be cross-summarized by baseline and visit. The assessment results of subjects will be tabulated by treatment group and subject number.

5.8.2 EORTC QLQ-C30

The EORTC QLQ-C30 consists of 30 items, including 5 functional scales, 3 symptom scales, 1 global health status/QoL scale, and 6 single items.

The 5 functional scales are physical functioning (Item 1 to Item 5), role functioning (Item 6, Item 7), cognitive functioning (Item 20, Item 25), emotional functioning (Item 21 to Item 24), and social functioning (Item 26, Item 27), the 3 symptom scales are fatigue (Item 10, Item 12, Item 18), nausea and vomiting (Item 14, Item 15), and pain (Item 9, Item 19), the 1 global health status/**QoL** scale (Item 29, Item 30), and the **6** symptom single items are dyspnea (Item 8), insomnia (Item 11), inappetence (Item 13), constipation (Item 16), diarrhea (Item 17), and financial difficulties (Item 28). Among them, Items 1 to 28 have 4 rank options (not at all, a little, quite a bit, very much), and Items 29 and 30 have 7 rank options ("very bad" to "very good").

To make each scale/item comparable, all scales and items are transformed into scores from 0 to 100. Higher scores on functional scale and global health status/QoL indicate higher levels of health functioning or quality of life, and higher scores on symptom scales indicate more severe symptom problems. Scale/Item scores are calculated as follows:

(1) Calculate the Raw Score (RS)

$$RS = (I_1 + I_2 + \cdots + I_\mathrm{n})/\mathrm{n}$$

(2) Standard score S obtained by linear transformation

Functional scales:

$$\mathrm{S} = \left\{1 - \frac{RS - 1}{range}\right\} \times 100$$

Symptom scales/single items:

$$\mathrm{S} = \{(RS - 1)/range\} \times 100$$

Global health/QoL scales:

$$\mathrm{S} = \{(RS - 1)/range\} \times 100$$

Where range denotes the difference between the maximum possible score and the minimum possible score for scales/items, Range 3 for Items 1 to 28, and Range 6 for Items 29 and 30.

Missing item scores in any scale will be handled as follows (note that for the scale with a single item, the scale will be considered missing if the item score is missing):

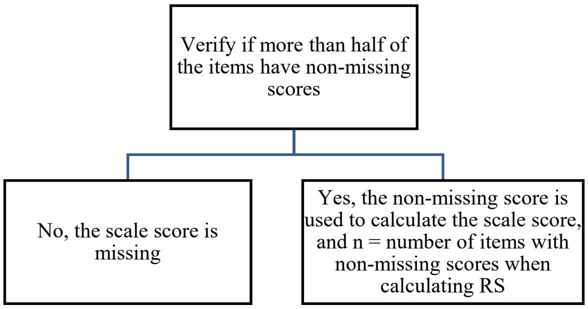

The scores of each scale will be analyzed descriptively by visit. The scores will be tabulated by treatment group and subject number.

5.8.3 EORTC QLQ-LC13

The EORTC QLQ-LC13 consists of 13 items, including 1 symptom scale, 9 symptom single items, and 1 question on the use of painkillers.

The 1 symptom scale is dyspnea (Item 33 to Item 35), the **9** symptom single items are cough (Item 31), hemoptysis (Item 32), oral pain (Item 36), dysphagia (Item 37), tingling hands or feet (Item 38), alopecia (Item 39), chest pain (Item 40), arm/shoulder pain (Item 41), and pain in other sites (Item 42), and the use of painkillers is Item 43. The symptom scale is only used if all 3 items are answered. If the answer to one item is missing, the other two items are handled as single items.

The calculation method for scale/single item scores is the same as that for EORTC QLQ-C30 symptom scale/single item scores. The scores of each scale will be analyzed descriptively by visit. The scores will be tabulated by treatment group and subject number.

6 CHANGES TO PROTOCOL/STATISTICAL ANALYSIS PLAN

No.	Category	Protocol	This SAP	Reason
1	Interim analysis	4.10. Interim analysis<br>OS will be analyzed for four times, including a blinded sample size re-estimation, the first interim efficacy analysis during the final analysis of PFS when reaching approximately 33% of the expected number of OS events, the second interim analysis when reaching approximately 66% of the expected number of OS events, and the final analysis when reaching the expected number of OS events.<br>Final analysis of PFS is planned to be conducted when the target number (about 396 PFS events) is observed.<br>The significance level for the final analysis of PFS of Group B versus Group C is 0.05 (two-sided).<br>PFS analysis will be tested in the following sequence:	3.6 Safety Data Monitoring and Interim Analysis<br>An Independent Data Monitoring Committee (IDMC) will be established in this study to perform safety data monitoring once every 6 months and interim analysis at the time points specified in the protocol.<br>At stage II, PFS is the primary endpoint, and OS is the key secondary endpoint.<br>PFS will be analyzed twice, including a blinded sample size re-estimation and a final analysis.<br>OS will be analyzed for three times, including a blinded sample size re-estimation, the first interim efficacy analysis of OS during the final efficacy analysis of PFS, and the final analysis when reaching the expected number of OS events.<br>The first interim analysis will be performed when about 420 subjects have been enrolled (approximately 2/3 of planned number of enrolled subjects), and its primary objective is to perform a blinded sample size re-estimation according to the actual PFS and OS data. This interim analysis will be performed in a blind state, without consuming the significance level α.<br>Final analysis of PFS is planned to be conducted when the target number (about 396 PFS events) is observed. The significance level for the final analysis of PFS is 0.05 (two-sided).<br>The first interim analysis of OS is planned to be performed with the final analysis of PFS, when the number of OS events is approximately 311 (72% of the total number of events). The estimated analysis time points and efficacy termination boundaries for the interim analysis and final analysis of OS in	According to the actual situation, the number of OS events has reached 72% of the total number of OS events during the final analysis of PFS, so the number of interim analyses for OS, analysis time points, and preset efficacy boundary were adjusted. The alpha significance level of the interim analysis for OS will be adjusted based on the actual number of events after subsequent unblinding.

<table>
<tr><th>No.</th><th>Category</th><th>Protocol</th><th>This SAP</th><th>Reason</th></tr>
<tr><td></td><td></td><td>1. HLX10 in combination with chemotherapy (Group B) versus control group (Group C)

2. HLX10 + HLX04 in combination with chemotherapy (Group A) versus HLX10 in combination with chemotherapy (Group B)

The first interim analysis of OS is planned to be performed with the final analysis of PFS, when the number of OS events is approximately 144

(33% of the total number of events). Based on the O'Brien-Fleming type α-spending function, the significance level for the analysis is 0.0002 (two-sided).

The second interim analysis of OS is planned to include approximately 288 OS events (66% of the total number of events). Based on the O'Brien-Fleming type α-spending function, the significance level for the</td><td>Group B (HLX10 combined with chemotherapy) and Group C (placebo combined with chemotherapy) are shown in Table 2, and the analysis time points and efficacy termination boundaries for the interim analysis and final analysis of OS in Group A (HLX10 + HLX04 combined with chemotherapy) and Group B (HLX10 combined with chemotherapy) are similar to those in Group B and Group C. Based on the actual number of OS events reached in Groups BC and AB at the analysis time points, the alpha significance level at the interim analysis will be adjusted using the Lan-DeMets approximation to the O'Brien-Fleming boundary.

The final analysis of OS is planned to be performed when 432 OS events are collected. The estimated analysis time points and termination boundaries are shown in Table 2. Based on the actual number of OS events reached in Groups BC and AB at the analysis time points, the alpha significance level at the final analysis will be adjusted using the Lan-DeMets approximation to the O'Brien-Fleming boundary.

Table 2. Efficacy termination boundaries for the interim analysis and final analysis of OS in Group B (HLX10 combined with chemotherapy) and Group C (placebo combined with chemotherapy)

<table>
<tr><th>Analysis</th><th>Number of events (% information ratio)</th><th>Hazard ratio</th><th>Z value</th><th>p-value</th></tr>
<tr><td>Interim analysis</td><td>208 (72%)
[planned]</td><td>0.7163</td><td>2.39751</td><td>0.0165</td></tr>
<tr><td>Final analysis</td><td>288 (100%)
[planned]</td><td>0.7892</td><td>2.00449</td><td>0.04502</td></tr>
</table>
Note: [1] The analysis time points and efficacy termination boundaries for the</td><td></td></tr>
</table>

No.	Category	Protocol	This SAP	Reason
		analysis is 0.012 (two-sided).<br>The final analysis of OS is planned to be performed when 432 OS events are collected. Based on the O'Brien-Fleming type α-spending function, the significance level for the final analysis is 0.046 (two-sided).<br>If any previous interim analysis in the sequence is not statistically significant, the alpha controlled at 0.05 (two-sided) will not be passed on to subsequent analyses.	interim analysis and final analysis of OS in Group A (HLX10 + HLX04 combined with chemotherapy) and Group B (HLX10 combined with chemotherapy) are similar to those in Group B and Group C.<br>[2] Based on the actual number of OS events reached in Groups BC and AB at the analysis time points, the alpha significance level at the interim analysis and final analysis will be adjusted using the Lan-DeMets approximation to the O'Brien-Fleming boundary.<br>A fixed-sequence test (the hypothesis tests are performed sequentially in the order of PFS in Group B (HLX10 combined with chemotherapy) and Group C (placebo combined with chemotherapy), OS in Group B and Group C, PFS in Group A (HLX10 + HLX04 combined with chemotherapy) and Group B (HLX10 combined with chemotherapy), OS in Group A and Group B) will be used to address multiplicity. Only when the null hypothesis is rejected for the previous hypothesis test, can the hypothesis test of the next endpoint be performed; if the null hypothesis is not rejected for the previous hypothesis test, the tests of all subsequent endpoints are stopped. At this time, the significance level of each hypothesis test is 0.05, and the overall type I error rate is still controlled within 0.05.	
2	Modified the definition of PKS	All subjects who have received at least one dose of HLX10 and have at least one post-dose concentration measurement at scheduled PK time points, without any major protocol deviations that can obviously affect the PK assessment. PKS will be used for PK analysis.	All subjects who have received at least one dose of HLX10 or HLX04 and have at least one post-dose concentration measurement at scheduled PK time points, without any major protocol deviations that can obviously affect the PK assessment. PKS will be used for PK analysis.	According to the actual situation, the serum concentrations of HLX10 and HLX04 were determined separately, and the definition of PKS was supplemented.

7 REFERENCES

1. ICH. Statistical Principles for Clinical Trials, Guideline E9, 1998. Available at http://www.emea.eu.int/pdfs/human/ich/036396en.pdf

2. CPMP. Points to Consider on Missing Data. EMEA: London, 2001. Available at http://www.emea.eu.int/pdfs/human/ewp/177699EN.pdf

3. Phillips A and Haudiquet V. ICH E9 guideline “Statistical principles for clinical trials”: a case study. Statistics in Medicine 2003; 22:1-11

4. Green S, Benedetti J and Crowley J. Clinical Trials in Oncology (2nd edition). Chapman & Hall/CRC, 2002.

5. McEntegart D. Forced randomization when using interactive voice response systems. Applied Clinical Trials October 2003; 50-58.

6. ICH. ICH E3 Guideline: Structure and Content of Clinical Study Reports Questions & Answers, 2012. Available at http://www.ich.org/fileadmin/Public_Web_Site/ICH_Products/Guidelines/Efficacy/E3/E3_QAs_R1_Step4.pdf.

7. Nan Luo, Gordon Liu Minghui Li etc. Estimating an EQ-5D-5L Value Set for China. Value in Health 20.(2017) 662-669.

8. EORTC Data Center. EORTC QLQ-C30 Scoring Manual(Third edition),2001. Available at http://www.eortc.be/home/qol/.
